# Retained colistin susceptibility in clinical *Acinetobacter baumannii* isolates with multiple mutations in *pmrCAB* and *lpxACD* operons

**DOI:** 10.3389/fcimb.2023.1229473

**Published:** 2023-08-01

**Authors:** Mai M. Zafer, Amira F. A. Hussein, Mohamed H. Al-Agamy, Hesham H. Radwan, Samira M. Hamed

**Affiliations:** ^1^ Department of Microbiology and Immunology, Faculty of Pharmacy, Ahram Canadian University, Cairo, Egypt; ^2^ Clinical and Chemical Pathology Department, Faculty of Medicine, Cairo University, Cairo, Egypt; ^3^ Faculty of Applied Health Science, Galala University, Cairo, Egypt; ^4^ Department of Pharmaceutics, College of Pharmacy, King Saud University, Riyadh, Saudi Arabia; ^5^ Department of Microbiology and Immunology, Faculty of Pharmacy, Al-Azhar University, Cairo, Egypt; ^6^ Department of Microbiology and Immunology, Faculty of Pharmacy, October University for Modern Sciences and Arts (MSA), 6th of October, Giza, Egypt

**Keywords:** healthcare-associated infections, *Acinetobacter baumannii*, extensive drug resistance, colistin resistance, whole-genome sequencing, *pmrCAB*, *lpxACD*, mutation

## Abstract

The progressive increase in the resistance rates to first- and second-line antibiotics has forced the reuse of colistin as last-line treatment for *Acinetobacter baumannii* infections, but the emergence of colistin-resistant strains is not uncommon. This has been long linked to acquired chromosomal mutations in the operons *pmrCAB* and *lpxACD*. Hence, such mutations are routinely screened in colistin-resistant strains by most studies. The current study was designed to explore the possible existence of *pmrCAB* and *lpxACD* mutations in colistin-susceptible isolates. For this purpose, the whole genome sequences of eighteen multi-/extensively drug resistant *A. baumannii* were generated by Illumina sequencing and screened for missense mutations of the operons *pmrCAB* and *lpxACD*. Most of the isolates belonged to global clones (GCs) including GC1 (n=2), GC2 (n=7), GC7 (n=2), GC9 (n=3), and GC11 (n=1). The minimum inhibitory concentrations (MICs) of colistin were determined by the broth microdilution assay. Seventeen isolates were fully susceptible to colistin with MICs ranging from (≤0.125 to 0.5 µg/ml). Interestingly, all colistin-susceptible isolates carried missense mutations in *pmrCAB* and *lpxACD* operons with reference to *A. baumannii* ATCC 19606. Overall, 34 mutations were found. Most substitutions were detected in *pmrC* (n=20) while no mutations were found in *pmrA* or *lpxA*. Notably, the mutation pattern of the two operons was almost conserved among the isolates that belonged to the same sequence type (ST) or GC. This was also confirmed by expanding the analysis to include *A. baumannii* genomes deposited in public databases. Here, we demonstrated the possible existence of missense mutations in *pmrCAB* and *lpxACD* operons in colistin-susceptible isolates, shedding light on the importance of interpreting mutations with reference to colistin-susceptible isolates of the same ST/GC to avoid the misleading impact of the ST/GC-related polymorphism. In turn, this may lead to misinterpretation of mutations and, hence, overlooking the real players in colistin resistance that are yet to be identified.

## 1 Introduction

The continual occurrence of mutations in non-susceptible pathogenic strains and the associated determinants of antimicrobial resistance within hospitals, countries, and across the world are currently the greatest threats to international Public Health (Shelenkov et al., 2021). The wide use of antibiotics in immunocompromised patients at intensive care units and the absence of antibiotic stewardship programs in hospital settings have led to the occurrence of pathogens that are multiple drug-resistant (MDR) and the rise of extensively drug-resistant (XDR) strains. Therefore, clinicians were forced to depend on colistin as the last treatment option to combat these infections (Seleim et al., 2022)*. A. baumannii*, a particularly problematic pathogen poses a significant threat to public health, by causing severe and invasive infections that are associated with high mortality rates in immunocompromised individuals and patients receiving intensive care. MDR and/or XDR *A. baumannii* have been reported to cause a significant degree of infections in Egypt during recent years (Ghaith et al., 2017; Al-Hassan et al., 2019). However, the direness of the situation is often masked in Egypt and other developing countries because of the lack of surveillance systems that can be attributed to restricted financial resources. Epidemiological surveillance is one significant tactic to assess and combat the burden exerted by problematic MDR pathogens in hospital settings. Overcoming antibiotic resistance is a critical challenge in the treatment of *Acinetobacter* infections (Wong et al., 2017), especially in view of the rise in carbapenem resistance among this species. Carbapenem resistance is generally associated with MDR and XDR phenotypes, which have been frequently demonstrated in *Acinetobacter* (Infectious Diseases Society Of, A 2012). Due to this problematic carbapenem-resistant *A. baumannii* (CRAB) clinical isolates, numerous Egyptian hospitals have reintroduced polymyxin use for therapy. Polymyxins E or colistin, the last therapeutic option for MDR Gram-negative pathogen infections, is widely used for the treatment of CRAB infections (Chamoun et al., 2021). Mechanistically, the interaction between the cationic non-ribosomal lipopeptides of colistin, and the lipid A component of lipopolysaccharide (LPS) in the outer membrane of the cell envelope destabilizes the latter. This subsequently allows the uptake of polymyxins into the periplasm and increases permeability by disrupting both outer and inner membrane integrity (Moffatt et al., 2019). Acquired colistin resistance develops primarily via drug target alteration. Substitutions or mobile genetic element insertion or deletion in the genes involved in the biosynthesis of lipid A, may result in structural or functional modifications in the lipid A moiety (Chamoun et al., 2021). Two key mechanisms for chromosomally mediated colistin resistance have been identified. The first involves LPS lipid A phosphoester group modifications, subsequent to *pmrCAB* operon mutations, which alter the affinity of lipid A to polymyxins by reducing its net negative charge. The operon *pmrCAB* comprises the *pmrC* gene that encodes a phosphoEthanolamine (pEtN) transferase, in addition to *pmrA* and *pmrB*, which encode the two component system (TCS), *PmrA/PmrB*. Overexpression of *pmrC* is induced by mutations in TCS (mainly *pmrA*) leading to colistin resistance via pEtN-mediated modification of lipid A [8,9]. Discrete genetic events are required for an adequate colistin resistance level in *A. baumannii*, which are up-regulation of *pmrAB*, *pmrB* point mutations, which leads to *pmrC* overexpression, and further addition of pEtN to lipid A (Beceiro et al., 2011). The second mechanism essentially involves termination of lipid A production due to various nucleotide substitutions, deletions, and insertions in one of its biosynthesis genes (*lpxA*, *lpxC*, and *lpxD*) that leads to frameshift mutations or produce truncated proteins that damage lipid A biosynthesis (Olaitan et al., 2014; Moffatt et al., 2019). In colistin-resistant isolates, there are non-synonymous mutations in the *lpxC* (P30L or S, N287D) and *lpxD* (E117K) genes that were previously reported from different origins (Oikonomou et al., 2015). Both colistin-susceptible and colistin-resistant isolates were found to carry the amino acid substitutions N287D (*lpxC*) and E117K (*lpxD*). It was also reported the possibility of these alterations in amino acids together with a mutation in the *pmrCAB* operon could result in synergistic effect that causes colistin resistance (Nurtop et al., 2019; Jovcic et al., 2021; Usjak et al., 2022). Several studies have focused on investigating the determinants of antibiotic resistance in colistin-resistant *A. baumannii* isolates. In this study, we investigated *pmrCAB* and *lpxACD* mutations in *A. baumannii* strains with retained susceptibility to colistin. Additionally, we made a comparative analysis of mutation patterns in colistin-susceptible isolates from distinct sequence types (STs).

## 2 Materials and methods

### 2.1 Bacterial strains

A total of 18 clinical isolates of *A. baumannii* were collected from the chemical and clinical pathology department at the Kasr Al-Ainy Hospital, Cairo University, Egypt, in the period from July to October 2020. All the isolates were obtained from patients at intensive care unit (ICU) and neonatal intensive care unit (NICU). The isolates were collected from blood, wound and sputum of adults and neonates. These were cultured on MacConkey agar medium (Oxoid, Altrincham, Cheshire, UK). Isolates were identified using Gram staining and culture morphology and were further confirmed using the VITEK 2 compact system (bioMérieux, Marcy l’Etoile, France) and amplification of *bla*_OXA-51-like_ genes using the Polymerase Chain Reaction (Turton et al., 2006).

Ethics approval and consent to participate: This work has been carried out in accordance with the relevant guidelines. The study was approved by the local ethical committee of the Department of Clinical and Chemical Pathology, Faculty of Medicine, Cairo University. All the collected isolates were recovered for routine investigations and no extra isolates were collected for the purpose of the study. Informed consent was not necessary as there was no direct contact with patients.

### 2.2 Antibiotic susceptibility testing

This test was performed on Mueller–Hinton agar (Oxoid, Altrincham, Cheshire, UK) using the standard disk diffusion method as per the Clinical and Laboratory Standards Institute guidelines (CLSI, 2020). A panel of 12 antibiotic disks, namely amikacin, cefepime, cefotaxime, cefoxitin, ceftriaxone, imipenem, levofloxacin, meropenem, piperacillin/tazobactam, tigecycline, and trimethoprim/sulfamethoxazole (Oxoid, Altrincham, Cheshire, UK) were used for the susceptibility profiling of the isolates. Minimum inhibitory concentrations (MICs) for colistin susceptibility were determined via the broth microdilution test. The results of the antimicrobial susceptibility tests were interpreted according to CLSI (2020) for all tested antimicrobial agents except tigecycline and colistin for which the breakpoints of the European Committee on Antimicrobial Susceptibility Testing (EUCAST, 2023) were used. As there is no tigecycline susceptibility breakpoints for *Acinetobacter* spp., we used the breakpoints specified for the *Enterobacterales*. While the CLSI (2020) has abolished the classification of *Acinetobacter* spp. as colistin-susceptible retaining only the intermediate (MIC ≤ 2) and resistant (MIC ≥ 4) categories, *Acinetobacter* spp. isolates having colistin MIC ≤ 2 are still categorized as colistin-susceptible by the EUCAST (2023). *Pseudomonas aeruginosa* ATCC 27853 and *Escherichia coli* ATCC 25922 were used as quality control strains.

### 2.3 Whole-genome sequencing (WGS), multilocus sequence typing (MLST), and analysis of colistin resistance genes

Draft genomes of the isolates were previously generated by us (Hamed et al., 2022) via Illumina MiSeq sequencing (Illumina Inc., San Diego, CA, USA). Extraction of DNA and library preparation were performed as previously described (Hamed et al., 2022). Illumina reads were assessed for quality using FastQC (Andrews, 2010) and low-quality reads were trimmed using Trimmomatic v0.32 (Bolger et al., 2014). SPAdes 3.14.1 was used for the de novo assembly of the trimmed reads (Bankevich et al., 2012). QUAST v5.0.2 (Gurevich et al., 2013) was used for generating the assembly metrics. Annotation of the draft genomes was achieved using the NCBI Prokaryotic Genome Annotation Pipeline (Tatusova et al., 2016). For plasmid assembly, we used PlasmidSPAdes (Antipov et al., 2016) and visualized the assembly graphs on bandage (Wick et al., 2015). The isolates were additionally analyzed for multilocus sequence types (MLST) using PubMLST server (https://pubmlst.org/abaumannii/). The phylogenetic relationships among each other, and with other international strains was analyzed using, CSI phylogeny 1.4 online tool (https://cge.cbs.dtu.dk/services/CSIPhylogeny/). Clonal complexes (CC) and global clones (GCs) were inferred via goeBURST analysis using the Phyloviz software version 2.0 (Ribeiro-Goncalves et al., 2016).

Our previous study focused on colistin resistance mechanisms in a colistin-resistant isolate, M19. In contrast, herein, we analyzed genes previously linked to colistin resistance in the colistin-susceptible isolates. The annotated files were visualized using the SnapGene v5.1.3.1, available at http://www.snapgene.com. Mutations were determined by pairwise alignment against the respective *A. baumannii* ATCC 19606 gene sequences using the Basic Local Alignment Search Tool (BLAST) available at https://blast.ncbi.nlm.nih.gov/Blast.cgi. Other colistin resistance genes were screened by the Comprehensive Antibiotic Resistance Database (CARD) server (https://card.mcmaster.ca/analyze/rgi) (Alcock et al., 2020).

### 2.4 Comparative analysis of *pmrCAB* and *lpxACD* operons in other *A. baumannii* genomes deposited in the BV-BRC database

To confirm that the mutation pattern found in our colistin-susceptible isolates is partly due to ST-related polymorphism, we compared the *pmrCAB* and *lpxACD* operons carried by our strains to strains sequenced in other studies. For this purpose, the public genome sequences of *A. baumannii* strains having the same STs and the closest genome sequences to our isolates were obtained from the Bacterial and Viral Bioinformatics Resource Center (BV-BRC), available at: https://www.bv-brc.org/, using the similar genome finder tool. Using the same platform, we extracted the predicted amino acid sequences of *pmrCAB* and *lpxACD* protein products. Multiple sequence alignments were done using Clustal Omega version 1.2.4 (https://www.ebi.ac.uk/Tools/msa/clustalo/) and visualized using MView version 1.63, available at: https://www.ebi.ac.uk/Tools/msa/mview/.

### 2.5 Zeta potential measurements

The zeta potential (ZP) of bacterial suspensions containing 1×10^9^ CFU/mL was measured after 10-fold dilution. The zeta cells were filled with bacterial cell suspensions, and the electrophoretic mobility (EPM) of the cells was recorded at 150 V and 25°C using a ZP analyzer (Malvern Zeta sizer Nano ZS, Malvern, Worcestershire, UK). To ensure reproducibility of measurements, a minimum of three runs per sample were conducted as described before (Soon et al., 2011).

### 2.6 Statistical analysis

Statistical analysis of the ZP values was performed with GraphPad Prism 8 software (GraphPad Software, San Diego, CA, USA). Comparison of the ZP of the isolates within each GC was done by the Student’s t-test and one-way analysis of variance (ANOVA), where appropriate. Pairwise comparisons were done using Tukey’s multiple comparisons test. P values less than 0.05 were considered statistically significant.

## 3 Results

**Figure 1:**
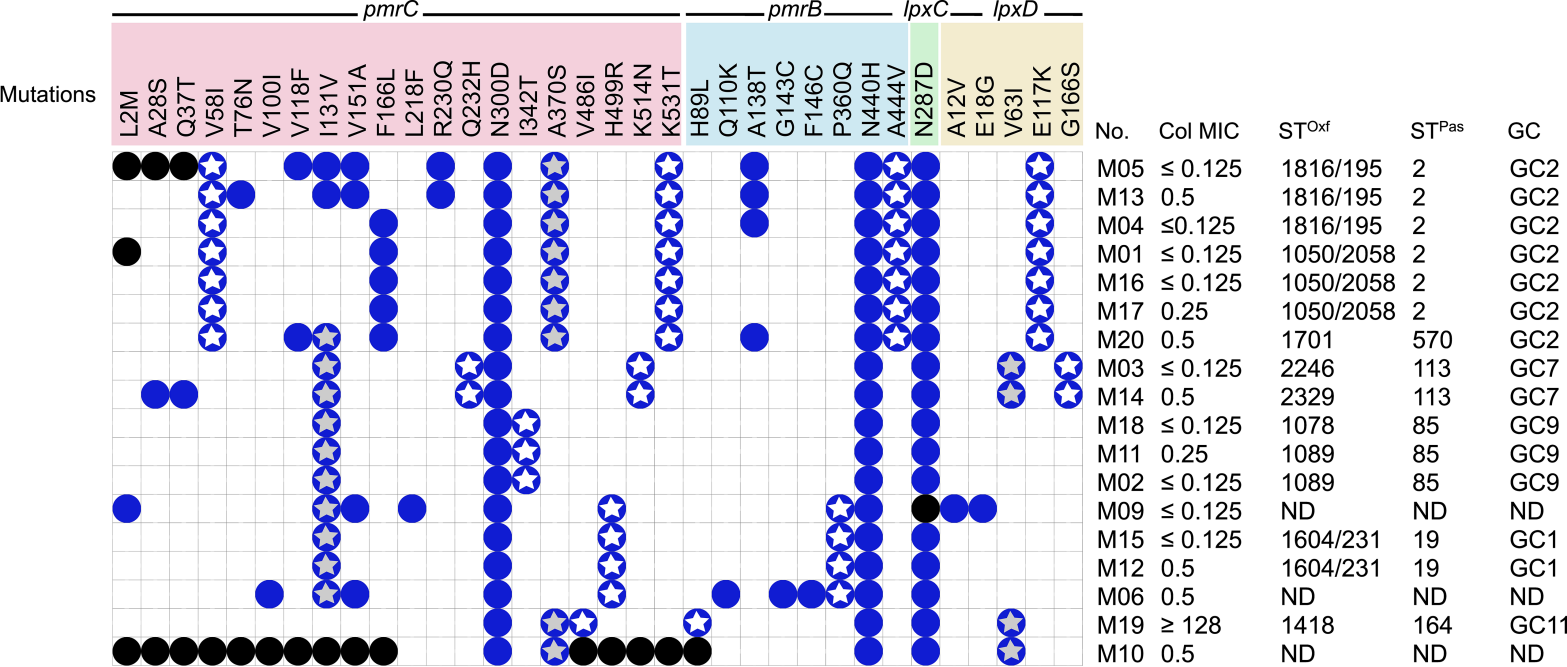
Distribution of *pmrCAB* and *lpxACD* mutations in the tested isolates and correlation to STs/GCs and colistin MIC. No missense mutations were identified in *pmrA* or *lpxA* in any of the tested isolates. Blue circles denote the presence of mutations; black circles means that the mutation could not be determined due to incomplete gene sequence; white stars denote ST-specific mutations; grey stars denote mutations shared between more than one ST; GC, global clone; MIC, minimum inhibitory concentration in µg/ml; ST^Pas^, sequence type according to Pasteur scheme; ST^Oxf^, sequence type according to Oxford scheme.

A total of 18 cultures that grew MDR/XDR *A. baumannii* were included in the study. Most patients were female and varied between neonates and older females. All the isolates were collected from ICU and NICU patients, and most cultures were obtained from patients with the greatest proportion of samples recovered from blood and wounds. Amplification of the *bla*_OXA-51-like_ gene confirmed that the collected isolates were *A. baumannii.* Most of the isolates belonged to GCs as shown in **Figure 1**.

### 3.1 Antimicrobial resistance profiles

All the isolates analyzed demonstrated high rates of resistance toward the tested antibiotics, with the exception of polymyxin and tigecycline, to which they were highly susceptible. Further, all isolates were CRAB (imipenem- and meropenem-resistant) and a total of five isolates were found to be amikacin-susceptible. Colistin susceptibility was tested by the broth microdilution method, and the MIC values obtained are shown in **Figure 1**. All the isolates were colistin-susceptible, except for M19, with an MIC ≥128 µg/ml (**Figure 1**).

### 3.2 Sequence analysis of genes related to colistin resistance

**Figure 2:**
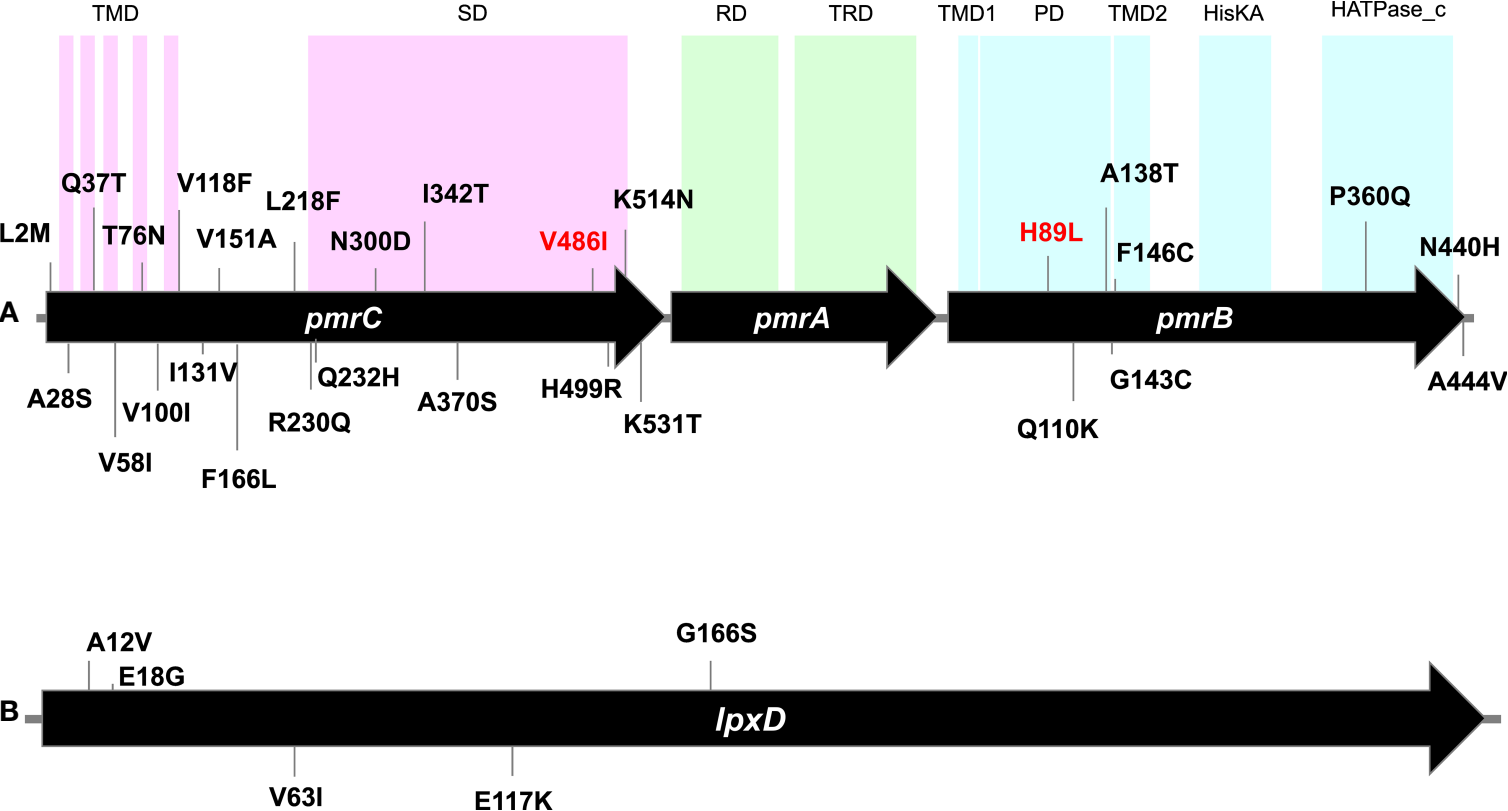
Gene maps showing point mutations identified in *pmrCAB* operon **(A)** and *lpxD* gene **(B)** carried by the tested isolates. Numbers denote codons affected by mutations. Mutations written in red are the unique mutations detected in the colistin-resistant isolate M19 but could not be determined in its phylogenetically related isolate M10. Mutations of *lpxC* were not shown as only one mutation (N278D) was carried by all isolates. TMD, transmembrane domain; SD, Sulfate domain; RD, receiver domain; TRD, transcriptional regulatory domain; PD, periplasmic domain; HisKA, histidine kinase A domain; HATPase_c, histidine kinase-like ATPase. Domain locations were adapted from Gerson et al. (2020) and Ko et al. (2017).

The assembled data of the WGS analysis of the isolates revealed the absence of mobile plasmid-mediated colistin resistance genes (*mcr1-10*). Several non-synonymous mutations were found in *pmrB*, *pmrC*, *lpx*C, and *lpx*D in the 18 isolates irrespective of their susceptibility to colistin (**Figure 2**).

#### 3.2.1 Amino acid substitutions in PmrCAB

While no mutations were found in *pmrA* among the 18 isolates, at least 20 amino acid substitutions were detected in PmrC. These included L2M, A28S, Q37T, V58l, T76N, V100l, V118F, l131V, V151A, F166L, L218F, R230Q, Q232H, N300D, l342T, A370S, V486l, H499R, K514N, and K531T (**Figure 2**). These amino acid substitutions were observed in colistin-susceptible isolates and were associated with colistin MICs that ranged from ≤0.125 µg/ml to 0.5 µg/ml. M19, the only colistin-resistant isolate with an MIC ≥128 µg/ml, harbored three mutations in *pmrC*. Of these two mutations (N300D and A370S) were also found in the colistin-susceptible strain M10 that is phylogenetically related to M19, while the third mutation (V486I) could not be determined. Notably, N300D was shared by all isolates. At least six mutations were ST/GC-specific. These included V58I that was shared by all GC2 isolates, Q232H that was carried by the two isolates that belonged to ST113^Pas^/GC7. In addition, I342T, H499R, V461I, and K531T were specific for the Pasteur STs ST85, ST19, ST164, and GC2, respectively. The mutation I131V was shared between the isolates that belonged to the STs ST113 ^Pas^, ST85^Pas^, and ST19^Pas^, while A370S was shared between ST164^Pas^ isolates and GC2 isolates.

Eight missense mutations, namely H89L, Q110K, A138T, G143C, F146C, P360Q, N440H, and A444V were detected in *pmrB*. Of these, H89L was detected only in M19, while N440H was found in all isolates. Two mutations were ST/GC-specific. These included P360Q that is specific for ST19^Pas^/GC1 isolates and A444V specific for GC2 isolates.

#### 3.2.2 Amino acid substitutions in LpxCD

Only one missense mutation was found in the gene *lpxC* and was predicted to be associated with the amino acid alteration N287D. The mutation was detected in all isolates except M09 in which the complete sequence of the gene could not be determined. Further, five amino acid substitutions in *lpxD*, namely A12V, E18G, V63I, E117K, and G166S were detected. No unique mutations were found in the colistin-resistant isolate M19 compared to the colistin-susceptible isolates.

The mutation E117K was specific for GC2 isolates, while G166S was found in the two isolates that belonged to ST113^Pas^. The mutation V63I was shared between two Pasteur STs namely ST113 and ST164. Correlations between ST and colistin MIC in the context of the detected mutations in the isolates analyzed are illustrated in **Figure 1**.

#### 3.2.3 Other colistin resistance determinants

**Figure 3:**
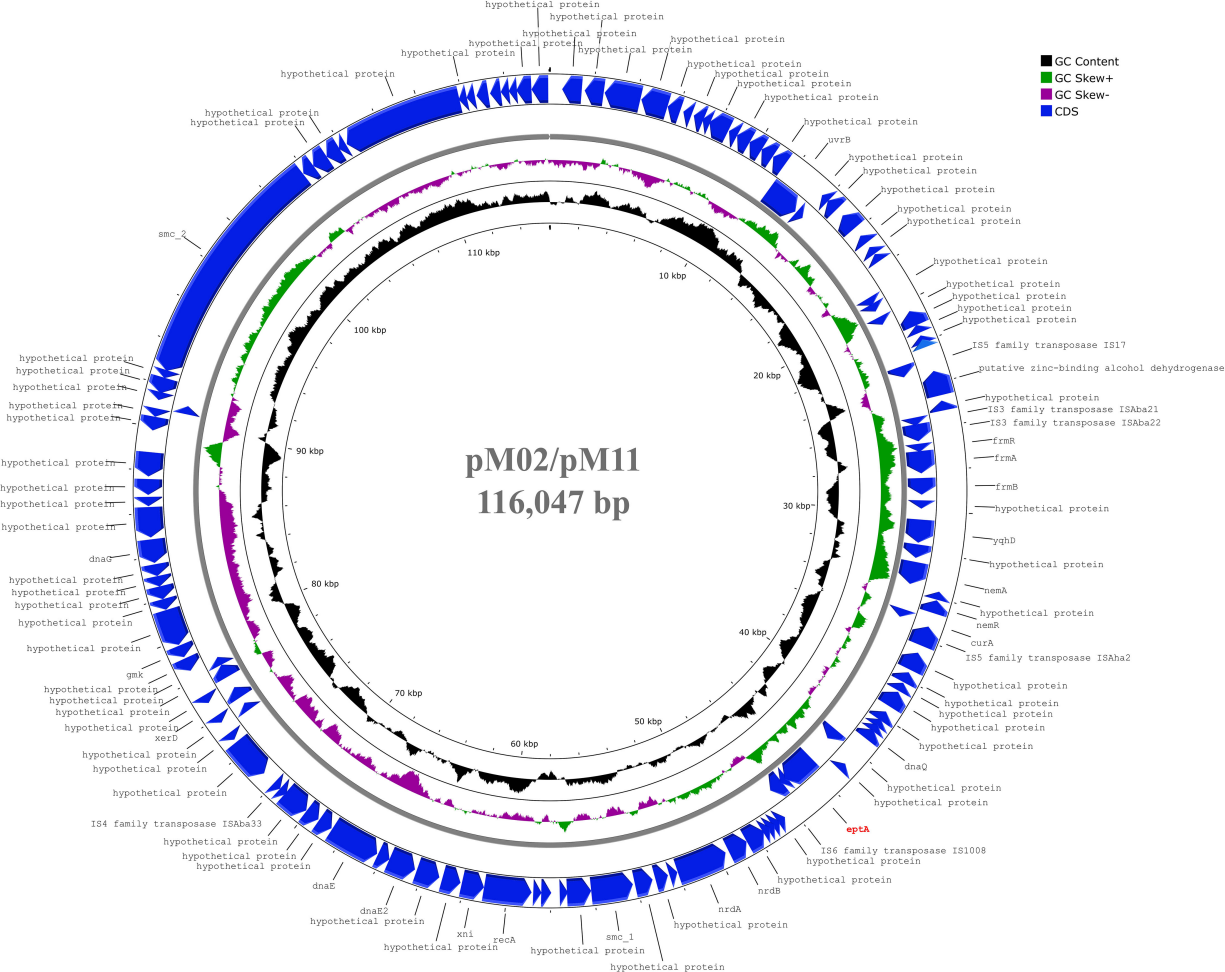
Circular map of 116,047 bp plasmid carried by M02 and M11 harbouring *eptA* gene. The plasmid showed highest similarity to another plasmid carried by *A. baumannii* strain ACN21 (GenBank: CP038645.1). The gene *eptA* was labelled in red and was not preceded by IS*Aba1* previously reported as essential for overexpression. The map was generated using the Proksee web server (https://proksee.ca/).

Genes encoding EptA (ethanolamine phosphotransferase), a homolog of PmrC, were identified in 10 of the 18 isolates. These were most commonly carried by the GC2 isolates (M01, M04, M05, M13, M16, M17, and M20), and less frequently in the GC9 isolates (M02 and M11). Only a single GC1 isolate (M12) harbored an EptA-coding gene. The EptA-coding genes carried by M01, M12, M13, M05, and M20 demonstrated similarity to *eptA-2* (GenBank accession: KC700023). Notably, none of the EptA-coding genes were preceded by an IS*Aba1* element that is known to drive *eptA* overexpression, which provides a PmrAB-independent mechanism for colistin resistance. The isolates M02 and M11 carried the EptA-coding genes on a 116,047 bp plasmid that showed 99.96% similarity to a plasmid carried by *A. baumannii*, ACN21 (GenBank accession: CP038645.1) (**Figure 3**). The genetic environment of EptA-coding genes in other strains could not be identified.

Further, an examination of IS*Aba*125 insertion into the H-NS family transcriptional regulator-coding gene, previously linked to high-level colistin resistance (Deveson Lucas et al., 2018) revealed its absence in the colistin-resistant (M19) as well as the colistin-susceptible isolates.

### 3.3 Comparison of *pmrCAB* and *lpxACD* sequences to closely-related global strains

In order to confirm the association between *pmrCAB* and *lpxACD* mutations and the STs/GCs rather than the phenotypic resistance to colistin, we made a large-scale mutation analysis on more isolates retrieved from the BV-BRC database. The operon sequences of our isolates were compared to global strains with the same STs. The multiple sequence alignments of the predicted amino acid sequences of PmrC, PmrB, LpxC, and LpxD are shown in **Supplementary Figures 1**-**19**. Our analysis confirmed the ST/GC specificity of all mutations described in our isolates carrying the same ST/GC. Some rare exceptions in which the wildtype genes were retained were also evident.

This expanded analysis also showed that the *pmrC* mutation V486I that was uniquely found in the colistin-resistant isolate M19 and could not be determined in the phylogenetically-related isolate M10 (**Figure 2**) was likely a ST-related polymorphism. This was evidenced by the existence of this mutation in all strains that belonged to the same ST. In contrast, the *pmrB* mutation H89L was only detected in M19 and three other foreign strains included in our analysis (151, 198, and GML-KP48-AB-TR). Unfortunately, the colistin susceptibility of the three strains was not available. Hence, further analysis is required to investigate the exact role of this mutation in colistin resistance.

Furthermore, we searched the metadata of the global strains included in the current study for colistin susceptibility. While the susceptibility profiles were not available for the majority of the strains, colistin susceptibility was reported for 33 strains. Only two strains namely, MS14413 (ST^Pas^ 2) and Ab-NDM-1 (ST^Pas^ 85), were reported to be colistin-resistant. In addition to the ST/GC-related polymorphism reported in the current study, MS14413 carried a unique mutation in *pmrB* that was associated by the amino acid alteration T232I. Similarly, only one unique mutation was found in Ab-NDM-1. This was found in *pmrB* gene and was associated by the amino acid alteration T187P.

### 3.4 Zeta potential alteration

**Figure 4:**
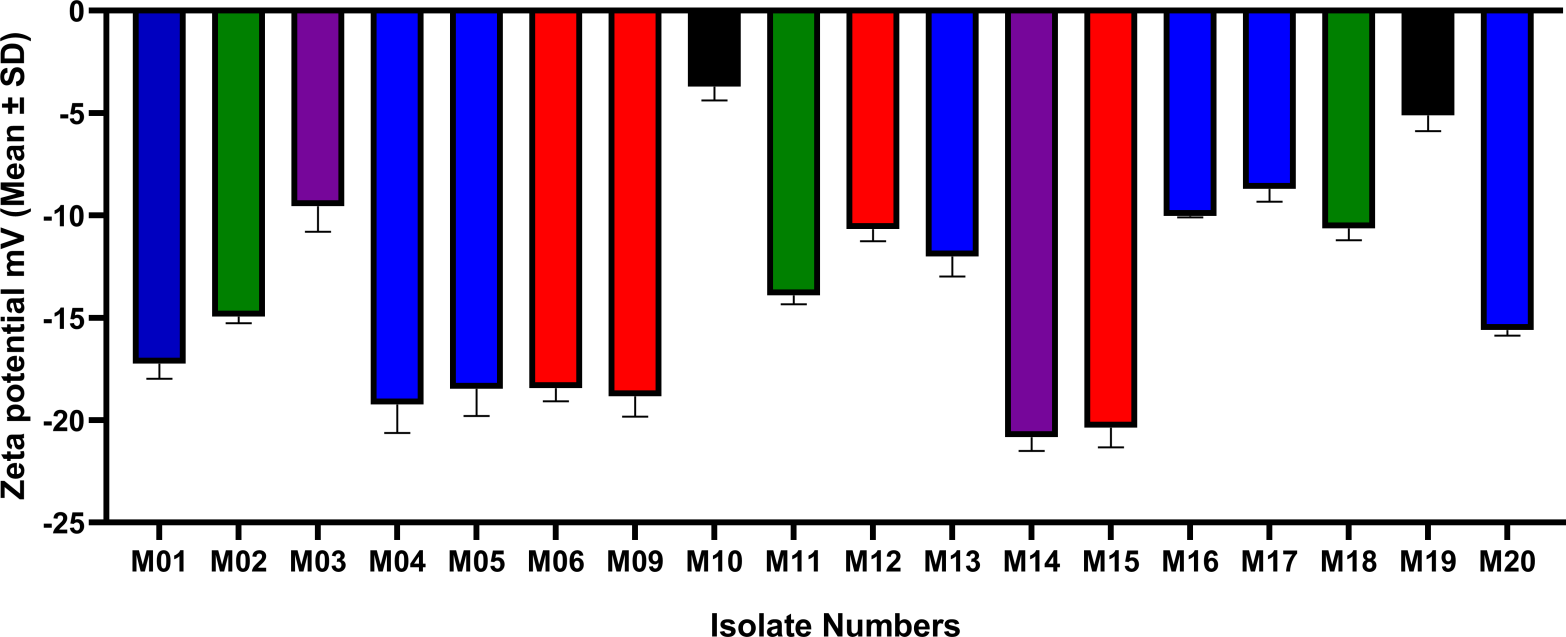
ZP values (mean±SD) of colistin-susceptible and resistant *A. baumannii* cells. Same colors refer to phylogenetically related isolates. M01, M04, M05, M13, M16, M17, and M20 all belong to GC2. M02, M11 and M18 has the same ST^Pas^85 and belong to GC9. M03 and M14 have the same ST^Pas^113 and belong to GC7. M06, M09, M12 and M15 belong to GC1. M10 is clonally related to M19 ST^Pas^164 (GC11).

A negative ZP was obtained for all tested isolates. The colistin-susceptible isolates revealed ZP values ranging from -20.8 ± 0.666 to -8.70 ± 0.627 mV. The ZP value of colistin-resistant cells (M19) was considerably less negative than that of the colistin-susceptible strains (-5.11 ± 0.77), while its phylogenetically-related colistin-susceptible isolate M10 had a less negative ZP (-3.71 ± 0.676) than all other colistin-susceptible strains and the resistant isolate M19. The ZP values of *A. baumannii* cells of all strains are shown in **Figure 4**.

Student’s *t*-test and One-way ANOVA were used for comparing the ZP values of all isolates within each GC/ST, while comparisons of the groups of isolates that belonged to different ST/GC were analyzed by one-way ANOVA. The difference between the ZP values of the isolates within each ST/GC was statistically significant (P-values <0.001). Additionally, a statistically significant difference was found between the ZP values of the isolates that belonged to different STs/GCs (P-value <0.0001) but pairwise comparisons showed that this significant difference exists only between GC11 isolates and others (P-values are shown in **Supplementary Figure 20**).

To investigate the relationship between the ZP and colistin resistance, pairwise comparisons were done between the ZP of individual isolates using the colistin-resistant strain M19 as a control. Significant difference was found between the ZP value of M19 and all other isolates except the phylogenetically-related colistin-susceptible isolate M10. P-values of the Tukey’s multiple comparison test are shown in **Supplementary Figure 21**.

## 4 Discussion

Treatment failure in *A. baumannii* is very common due to lack of understating the exact mechanisms of resistance to antibiotics together with the absence of novel therapies. In recent years, the alarming increase in the rates of CRAB strains in Egyptian hospitals have forced the reuse of colistin for treating these pathogens. Colistin is the antibiotic of choice for treating CRAB infections in healthcare facilities, a significant challenge to infection control in nosocomial settings. Elucidating the exact mechanisms that mediate and control colistin resistance is thus crucial to preserve its efficacy. To explore the relationship between chromosomally-mediated mechanisms of colistin resistance and the colistin resistance phenotype, we investigated 18 clinical isolates of *A. baumannii* collected from ICU and NICU patients by WGS. All of the isolates were previously characterized for MLST according to Pasteur and Oxford scheme. Some isolates were assigned two Oxford STs due to carrying two copies of *gdhB* locus, as described before (Gaiarsa et al., 2019). Most of the isolates belonged to high-risk GCs including GC1, GC2, GC7, GC9, and the most recently described clone, GC11 (Hansen et al., 2023).

Given the importance of studying resistance mechanisms in distinct strains, genetic investigation was carried out in colistin-susceptible strains. Detecting the MIC of colistin using the broth microdilution method is the only reliable method and remains the gold standard for assessing colistin resistance in *A. baumannii* approved by both the CLSI and by the EUCAST (CLSI, 2020; EUCAST, 2021). Of the 18 isolates analyzed, only a single strain, M19, demonstrated colistin resistance with an MIC ≥128 µg/ml. This percent of resistance was much lower than that was recently reported in Egypt in a study conducted on 17 A*. baumannii* isolates in which nine of them were colistin-resistant (Fam et al., 2020). It is noteworthy to mention that high rates of resistance toward different antibiotics were detected among our studied collection, and this could be attributed to the fact that high resistance patterns are usually observed in critically ill hospitalized patients due to existence of comorbidities and the overuse of antibiotics.

The plasmid-mediated colistin resistance determinants *mcr* genes were not detected in our studied isolates. This goes in line with most of studies conducted on colistin-resistant *A. baumannii* isolates. Moreover, the studies that reported the existence of *mcr* genes failed to sequence any *mcr* variants (Khoshnood et al., 2020; Snyman et al., 2020). Instead, *pmrAB* mutations appears to be the driving mechanism of colistin resistance in *A. baumannii* in most of published reports (Seleim et al., 2022). A total of 34 point mutations were identified in our strains (**Figure 2**) within the *pmrCAB* and *lpx*ACD operons. The association of colistin resistance in *A. baumannii* with mutations in the putative two-component regulatory system PmrAB was first reported in 2009 (Adams et al., 2009). Although previous studies have reported mutations in the response regulator-coding gene *pmrA* (Arroyo et al., 2011; Lesho et al., 2013), we observed none. Colistin resistance is primarily a consequence of mutations in the *pmrCAB* operon, especially in the *pmrB* region (Nurtop et al., 2019), and we observed the highest number of mutations in the *pmrC* region. Sequence analysis revealed eight amino acid substitutions in PmrB and 20 amino acid substitutions in PmrC, all of which were detected in colistin-susceptible isolates, except for the single colistin-resistant strain, M19. Thus, it may be inferred that not all amino acid substitutions result in elevated colistin MICs. Seleim et al. (2022) reported that the *pmrC* expression level could not differentiate between colistin-resistant and colistin-susceptible isolates, and they observed that the correlation between *pmrC* expression levels and colistin MICs was not significant (Seleim et al., 2022). Additionally, this was also reported by Gerson et al. (2020). Thus, it is therefore concluded that colistin resistance mechanisms in *A. baumannii* are much more complicated than believed. The resistant strain M19 harbored unique and distinct mutations in *pmrC* and *pmrB*, and none in *pmrA*. These included three substitutions in PmrC, including N300D, A370S, and V486I, and two substitutions, namely H89L and N440H in PmrB. Notably, V486I and H89L were only observed in this strain. The H89L substitution in PmrB (Nurtop et al., 2019) and the V486I substitution in PmrC (Srisakul et al., 2022) have been previously reported in colistin-resistant *A. baumannii* isolates. While found here in colistin-susceptible isolates that belonged to three STs, some *pmrC* mutations (I131V and H499R) and *pmrB* mutations (P360Q) were previously linked to resistance to colistin (Arroyo et al., 2011; Misic et al., 2018). In agreement with our findings, N440H and A444V mutations of *pmrB* were previously predicted not to affect colistin susceptibility (Thi Khanh Nhu et al., 2016). To completely understand lipid A modification and the resulting colistin-susceptible phenotype, the effects exerted by *pmrCAB* mutations on the expression of *pmrC* require further investigation.

A total of six diverse LpxCD amino acid substitutions in colistin-susceptible *A. baumannii* strains were evident. The colistin-resistant isolate M19 harbored the mutation N287D in *lpx*C that was also present in the colistin-susceptible isolates. A previous study reported that mutations in *lpx*D or *pmr*B alone may suffice to induce colistin resistance, thus suggesting synergism between the effects of mutations within these genes in promoting the same (Nurtop et al., 2019). The diverse range of amino acid substitutions reported in this study, some of which have been previously described, suggest that the exact colistin resistance mechanisms in *A. baumannii* requires extensive investigation. Interestingly, conserved mutation patterns were mostly found in *A. baumannii* strains that belonged to the same ST/GC, particularly those inferred by the Pasteur scheme, regardless of their susceptibility to colistin. This was further confirmed by an expanded analysis in which more strains with the same STs as those identified here were included in the mutation analysis. Hence, we here emphasize the importance of carefully analyzing colistin resistance-related mutations with reference to a colistin-susceptible strain that belongs to the same ST as the one under investigation. Otherwise, the mechanisms underlying colistin resistance may be incorrectly inferred and the real contributors to colistin resistance may be overlooked. We found many studies that investigated *pmrCAB* and *lpxACD* mutations with reference to *A. baumannii* ATCC 19606 and ATCC 17978 that may lead to overestimation of mutation-related resistance and overlooking the real mechanism of resistance (Mavroidi et al., 2015; Haeili et al., 2018; Nurtop et al., 2019; Jovcic et al., 2021; Eze et al., 2022; Usjak et al., 2022; Kabic et al., 2023).

As the susceptibility profiles of the global strains included in our study were revised, only two strains with confirmed colistin resistance were identified (MS14413 and Ab-NDM-1). Interestingly, the only unique mutations identified in the two strains were found in *pmrB*, confirming the crucial role of this gene in driving colistin resistance. Notably, other mutations identified by the authors as a contributor to colistin resistance in Ab-NDM-1 were the *pmrC* mutations I115V, N284D, and I326T (Fernandez-Cuenca et al., 2020). These correspond to the mutations I131V, N300D, and I342T (according to the numbering of *A. baumannii* ATC 19606, GenBank CP059040) identified here as polymorphism. This further emphasizes the importance of the data presented here for the correct interpretation of *pmrCAB* and *lpxACD* mutations.

The bacterial surface charge has frequently been described in terms of ZP, which is the potential at the shear plane of the electrical double layer surrounding a cell in solution (Soon et al., 2011). Our results showed that the colistin-resistant cells display less negative ZP than the colistin-susceptible cells. The less negative ZP exhibited by the colistin-resistant isolate M19 in comparison to that of the colistin-susceptible cells has been previously explained to be a consequence of alterations in the structure and composition of the outer membrane (Soon et al., 2011). Colistin-resistant cells were previously reported to have more propensity for clumping in small clusters compared to colistin-susceptible cells (Soon et al., 2009). This was justified by the higher colloid aggregate stability within the particle carrying a lower magnitude of charge due to reduced electrostatic repulsion (Klodzinska et al., 2010). Lipid A phosphates esterification with 2-aminoethanol or 4-amino-4-deoxy-L-arabinose resulting in charge shielding has been reported before in colistin-resistant *E. coli* (Gatzeva-Topalova et al., 2005) and *Pseudomonas aeruginosa* (Gooderham and Hancock, 2009). Unexpectedly, M10, a colistin-susceptible isolate, exhibited the lowest negative ZP among all the isolates. Notably, this isolate is phylogenetically related to M19 (the colistin-resistant isolate) as described previously (Hamed et al., 2022), and thus the aberrant zeta potential may either be a consequence of cell membrane alterations or a reflection of ST-related polymorphism. Statistical analysis of the ZP values of all isolates revealed a significant difference between the ZP values of the isolates that belonged to the same ST/GC. This contradicts our hypothesis that the isolates that belong to the same ST/GC may have similar ZP values. More studies are recommended to better understand the relationship between STs and the ZP of *A. baumannii*.

The existence of the alternative pEtN transferase named ethanolamine phosphotransferase A-1 (EptA) in *A. baumannii* was previously demonstrated by Lesho et al. (2013). The authors described two PmrC homologs designated EptA-1 and EptA-2 that were localized outside the *pmrABC* operon. The same study has also reported the overexpression of *eptA*-1 and *eptA*-2 in the colistin-resistant isolates. Another study conducted on a pair of MDR *A. baumannii* by Deveson Lucas et al. (2018) indicated that overexpression of the “orphan” *eptA* in a pre-colistin-treatment *A. baumannii* strain resulted in increased colistin resistance (Deveson Lucas et al., 2018). This finding provides evidence for *eptA* encoding a functional pEtN transferase that mediates colistin resistance (Deveson Lucas et al., 2018). In our collection, EptA-coding genes were harbored by the GC2 isolates (M01, M04, M05, M13, M16, M17, and M20), two GC9 isolates (M02, M11) and a single GC1 isolate, M12. None of the *eptA* genes identified here was preceded by IS*Aba1* insertion. This confirms the assumption made by Potron et al. (2019) that overexpression of *eptA* genes, and subsequently colistin resistance is a consequence of an upstream insertion of an IS*Aba1* element (Potron et al., 2019).

The insertion of IS*Aba125* within a gene encoding an H-NS family transcriptional regulator was previously linked to *eptA* overexpression and colistin resistance (Deveson Lucas et al., 2018). This was not found in any of our isolates.

## 5 Conclusion

Our study reinforces the need for extensive investigations for the elucidation of the exact mechanisms that contribute to colistin resistance in *A. baumannii*, which consequently influences possible therapeutic options. Colistin resistance mechanisms in *A. baumannii* are complex and not easily understood. Multiple mutations in *pmrCAB* and *lpx*ACD are unlikely to result in increased colistin resistance. The evaluation of mutations with reference to colistin-susceptible isolates of the same ST/GC is essential to avoid misinterpretation of ST/GC-related polymorphisms. Evidence was provided by expanding our analysis to include *A. baumannii* strains with the same STs as our isolates retrieved from a public database. Further, large scale studies including more colistin-resistant and colistin-susceptible strains are recommended to better establish the relationship between the ZP and colistin resistance and to confirm, with statistical evidence, the ST-related polymorphism of *pmrCAB* and *lpx*ACD operons that may exist in both colistin-susceptible and colistin-resistant strains.

## Data availability statement

The datasets presented in this study can be found in online repositories. The names of the repository/repositories and accession number(s) can be found in the article/**Supplementary Material**.

## Author contributions

Conceptualization, MZ, AH, MA, HR, and SH. Methodology, MZ, AH, and SH. Software, SH. Writing—original draft preparation, MZ. Writing—review and editing, MZ, AH, MA, HR, and SH. Visualization, SH. Funding acquisition, MA. All authors have read and agreed to the published version of the manuscript.
