# Supplementary material for: Retained colistin susceptibility in clinical Acinetobacter baumannii isolates with multiple mutations in pmrCAB and lpxACD operons
Source: Front Cell Infect Microbiol. 2023 Aug 1;13:1229473. doi: 10.3389/fcimb.2023.1229473 (PMC10436201; doi:10.3389/fcimb.2023.1229473)
Supplement: Supplementary file 1 [file DataSheet_1.docx]

Supplementary Material

cov pid  **1** **[ . . . . : . . .** **80**

1 ATCC19606 100.0% 100.0% **MLNFFSTLRNKQISLFMFNLIIAIWLGAILNIGFYHQVHTLTPYFGVKAILFLAATLVILVATYYAVLQILNWKWTAKIF**

2 M06 86.2% 98.9% **----------------------------------------------------------------------------AKIF**

3 M09 100.0% 98.9% **MMNFFSTLRNKQISLFMFNLIIAIWLGAILNIGFYHQVHTLTPYFGVKAILFLAATLVILVATYYAVLQILNWKWTAKIF**

4 M15 59.4% 99.4% **MLNFFSTLRNKQISLFMFNLIIAIWLGAILNIGFYHQVHTLTPYFGVKAILFLAATLVILVATYYAVLQILNWKWTAKIF**

5 M15b 39.0% 99.5% **--------------------------------------------------------------------------------**

6 M12 100.0% 99.5% **MLNFFSTLRNKQISLFMFNLIIAIWLGAILNIGFYHQVHTLTPYFGVKAILFLAATLVILVATYYAVLQILNWKWTAKIF**

7 SRR3222490 97.1% 99.4% **----------------MFNLIIAIWLGAILNIGFYHQVHTLTPYFGVKAILFLAATLVILVATYYAVLQILNWKWTAKIF**

8 SRR3227013 97.1% 99.4% **----------------MFNLIIAIWLGAILNIGFYHQVHTLTPYFGVKAILFLAATLVILVATYYAVLQILNWKWTAKIF**

9 SRR3228488 9.8% 100.0% **----------------MFNLIIAIWLGAILNIGFYHQVHTLTPYFGVKAILFLAATLVILVATYYAVLQI----------**

10 SRR3228488b 45.4% 99.6% **--------------------------------------------------------------------------------**

11 SRR3228488c 32.8% 99.4% **--------------------------------------------------------------------------------**

12 SRR3228488d 32.8% 97.8% **--------------------------------------------------------------------------------**

13 SRR3228565 97.1% 99.4% **----------------MFNLIIAIWLGAILNIGFYHQVHTLTPYFGVKAILFLAATLVILVATYYAVLQILNWKWTAKIF**

14 A18 100.0% 99.5% **MLNFFSTLRNKQISLFMFNLIIAIWLGAILNIGFYHQVHTLTPYFGVKAILFLAATLVILVATYYAVLQILNWKWTAKIF**

15 15A1042 9.5% 98.1% **--------------------------------------------------------------------------------**

16 15A1042b 80.3% 99.5% **MLNFFSTLRNKQISLFMFNLIIAIWLGAILNIGFYHQVHTLTPYFGVKAILFLAATLVILVATYYAVLQILNWKWTAKIF**

17 15A1042c 8.9% 85.7% **--------------------------------------------------------------------------------**

18 15A1042d 40.4% 98.2% **--------------------------------------------------------------------------------**

19 MRSN7133 97.1% 99.4% **----------------MFNLIIAIWLGAILNIGFYHQVHTLTPYFGVKAILFLAATLVILVATYYAVLQILNWKWTAKIF**

20 MRSN7130 97.1% 99.4% **----------------MFNLIIAIWLGAILNIGFYHQVHTLTPYFGVKAILFLAATLVILVATYYAVLQILNWKWTAKIF**

21 MRSN7202 97.1% 99.4% **----------------MFNLIIAIWLGAILNIGFYHQVHTLTPYFGVKAILFLAATLVILVATYYAVLQILNWKWTAKIF**

22 MRSN7224 97.1% 99.4% **----------------MFNLIIAIWLGAILNIGFYHQVHTLTPYFGVKAILFLAATLVILVATYYAVLQILNWKWTAKIF**

23 OIFC074 7.8% 83.7% **--------------------------------------------------------------------------------**

24 OIFC074b 8.4% 97.8% **--------------------------------------------------------------------------------**

cov pid  **81**  **. 1 . . . . : .** **160**

1 ATCC19606 100.0% 100.0% **AILLIFIGGFSSYFVNTLGVIISPDQIQNMVQTDVSEVTDLISLRFVLWTIFFVILPIFLITQVKFKQEKVSRLLLKKVF**

2 M06 86.2% 98.9% **AILLIFIGGFSSYFVNTLGIIISPDQIQNMVQTDVSEVTDLISLRFVLWTVFFVILPIFLITQVKFKQEKASRLLLKKVF**

3 M09 100.0% 98.9% **AILLIFIGGFSSYFVNTLGVIISPDQIQNMVQTDVSEVTDLISLRFVLWTVFFVILPIFLITQVKFKQEKASRLLLKKVF**

4 M15 59.4% 99.4% **AILLIFIGGFSSYFVNTLGVIISPDQIQNMVQTDVSEVTDLISLRFVLWTVFFVILPIFLITQVKFKQEKVSRLLLKKVF**

5 M15b 39.0% 99.5% **--------------------------------------------------------------------------------**

6 M12 100.0% 99.5% **AILLIFIGGFSSYFVNTLGVIISPDQIQNMVQTDVSEVTDLISLRFVLWTVFFVILPIFLITQVKFKQEKVSRLLLKKVF**

7 SRR3222490 97.1% 99.4% **AILLIFIGGFSSYFVNTLGVIISPDQIQNMVQTDVSEVTDLISLRFVLWTVFFVILPIFLITQVKFKQEKVSRLLLKKVF**

8 SRR3227013 97.1% 99.4% **AILLIFIGGFSSYFVNTLGVIISPDQIQNMVQTDVSEVTDLISLRFVLWTVFFVILPIFLITQVKFKQEKVSRLLLKKVF**

9 SRR3228488 9.8% 100.0% **--------------------------------------------------------------------------------**

10 SRR3228488b 45.4% 99.6% **--------------------------------------------------------------------------------**

11 SRR3228488c 32.8% 99.4% **------------------------DQIQNMVQTDVSEVTDLISLRFVLWTVFFVILPIFLITQVKFKQEKVSRLLLKKVF**

12 SRR3228488d 32.8% 97.8% **------------------------DQIQNMVQTDVSEFTDLISLRFVLWTVFFVILPIFLITQVKFKQEKASRLLLKKVF**

13 SRR3228565 97.1% 99.4% **AILLIFIGGFSSYFVNTLGVIISPDQIQNMVQTDVSEVTDLISLRFVLWTVFFVILPIFLITQVKFKQEKVSRLLLKKVF**

14 A18 100.0% 99.5% **AILLIFIGGFSSYFVNTLGVIISPDQIQNMVQTDVSEVTDLISLRFVLWTVFFVILPIFLITQVKFKQEKVSRLLLKKVF**

15 15A1042 9.5% 98.1% **--------------------------------------------------------------------------------**

16 15A1042b 80.3% 99.5% **AILLIFIGGFSSYFVNTLGVIISPDQIQNMVQTDVSEVTDLISLRFVLWTVFFVILPIFLITQVKFKQEKVSRLLLKKVF**

17 15A1042c 8.9% 85.7% **--------------------------------------------------------------------------------**

18 15A1042d 40.4% 98.2% **-------------------VIISPDQIQNMVQTDVSEVTDLISLRFVLWTVFFVILPIFLITQVKFKQEKASRLLLKKVF**

19 MRSN7133 97.1% 99.4% **AILLIFIGGFSSYFVNTLGVIISPDQIQNMVQTDVSEVTDLISLRFVLWTVFFVILPIFLITQVKFKQEKVSRLLLKKVF**

20 MRSN7130 97.1% 99.4% **AILLIFIGGFSSYFVNTLGVIISPDQIQNMVQTDVSEVTDLISLRFVLWTVFFVILPIFLITQVKFKQEKVSRLLLKKVF**

21 MRSN7202 97.1% 99.4% **AILLIFIGGFSSYFVNTLGVIISPDQIQNMVQTDVSEVTDLISLRFVLWTVFFVILPIFLITQVKFKQEKVSRLLLKKVF**

22 MRSN7224 97.1% 99.4% **AILLIFIGGFSSYFVNTLGVIISPDQIQNMVQTDVSEVTDLISLRFVLWTVFFVILPIFLITQVKFKQEKVSRLLLKKVF**

23 OIFC074 7.8% 83.7% **--------------------------------------------------------------------------------**

24 OIFC074b 8.4% 97.8% **--------------------------------------------------------------------------------**

cov pid **161**  **. . . 2 . . . .** **240**

1 ATCC19606 100.0% 100.0% **SLVASFAVVGVLLFTYYVDFAAIFREHRDLKGMISPQNSISSLMSYYHKKAPKKNLPLVIYGQDAHQVQRVQKNLPKLMI**

2 M06 86.2% 98.9% **SLVASFAVVGVLLFTYYVDFAAIFREHRDLKGMISPQNSISSLMSYYHKKAPKKNLPLVIYGQDAHQVQRVQKNLPKLMI**

3 M09 100.0% 98.9% **SLVASFAVVGVLLFTYYVDFAAIFREHRDLKGMISPQNSISSLMSYYHKKAPKKNLPFVIYGQDAHQVQRVQKNLPKLMI**

4 M15 59.4% 99.4% **SLVASFAVVGVLLFTYYVDFAAIFREHRDLKGMISPQNSISSLMSYYHKKAPKKNLPLVIYGQDAHQVQRVQKNLPKLMI**

5 M15b 39.0% 99.5% **--------------------------------------------------------------------------------**

6 M12 100.0% 99.5% **SLVASFAVVGVLLFTYYVDFAAIFREHRDLKGMISPQNSISSLMSYYHKKAPKKNLPLVIYGQDAHQVQRVQKNLPKLMI**

7 SRR3222490 97.1% 99.4% **SLVASFAVVGVLLFTYYVDFAAIFREHRDLKGMISPQNSISSLMSYYHKKAPKKNLPLVIYGQDAHQVQRVQKNLPKLMI**

8 SRR3227013 97.1% 99.4% **SLVASFAVVGVLLFTYYVDFAAIFREHRDLKGMISPQNSISSLMSYYHKKAPKKNLPLVIYGQDAHQVQRVQKNLPKLMI**

9 SRR3228488 9.8% 100.0% **--------------------------------------------------------------------------------**

10 SRR3228488b 45.4% 99.6% **--------------------------------------------------------------------------------**

11 SRR3228488c 32.8% 99.4% **SLVASFAVVGVLLFTYYVDFAAIFREHRDLKGMISPQNSISSLMSYYHKKAPKKNLPLVIYGQDAHQVQRVQKNLPKLMI**

12 SRR3228488d 32.8% 97.8% **SLVASFAVVGVLLFTYYVDFAAIFREHRDLKGMISPQNSISSLMSYYHKKAPKKNLPFVIYGQDAHQVQRVQKNLPKLMI**

13 SRR3228565 97.1% 99.4% **SLVASFAVVGVLLFTYYVDFAAIFREHRDLKGMISPQNSISSLMSYYHKKAPKKNLPLVIYGQDAHQVQRVQKNLPKLMI**

14 A18 100.0% 99.5% **SLVASFAVVGVLLFTYYVDFAAIFREHRDLKGMISPQNSISSLMSYYHKKAPKKNLPLVIYGQDAHQVQRVQKNLPKLMI**

15 15A1042 9.5% 98.1% **--------------------------------------------------------------------------------**

16 15A1042b 80.3% 99.5% **SLVASFAVVGVLLFTYYVDFAAIFREHRDLKGMISPQNSISSLMSYYHKKAPKKNLPLVIYGQDAHQVQRVQKNLPKLMI**

17 15A1042c 8.9% 85.7% **--------------------------------------------------------------------------------**

18 15A1042d 40.4% 98.2% **SLVASFAVVGVLLFTYYVDFAAIFREHRDLKGMISPQNSISSLMSYYHKKAPKKNLPFVIYGQDAHQVQRVQKNLPKLMI**

19 MRSN7133 97.1% 99.4% **SLVASFAVVGVLLFTYYVDFAAIFREHRDLKGMISPQNSISSLMSYYHKKAPKKNLPLVIYGQDAHQVQRVQKNLPKLMI**

20 MRSN7130 97.1% 99.4% **SLVASFAVVGVLLFTYYVDFAAIFREHRDLKGMISPQNSISSLMSYYHKKAPKKNLPLVIYGQDAHQVQRVQKNLPKLMI**

21 MRSN7202 97.1% 99.4% **SLVASFAVVGVLLFTYYVDFAAIFREHRDLKGMISPQNSISSLMSYYHKKAPKKNLPLVIYGQDAHQVQRVQKNLPKLMI**

22 MRSN7224 97.1% 99.4% **SLVASFAVVGVLLFTYYVDFAAIFREHRDLKGMISPQNSISSLMSYYHKKAPKKNLPLVIYGQDAHQVQRVQKNLPKLMI**

23 OIFC074 7.8% 83.7% **--------------------------------------------------------------------------------**

24 OIFC074b 8.4% 97.8% **--------------------------------------------------------------------------------**

cov pid **241**  **: . . . . 3 . .** **320**

1 ATCC19606 100.0% 100.0% **LVVGETARAESFSLNGYAKNTNPELSKQDIFNFSQVSSCGTATAVSVPCMFSGMPRVDYNEQLASHREGLLDIAKRAGYQ**

2 M06 86.2% 98.9% **LVVGETARAESFSLNGYAKNTNPELSKQDIFNFSQVSSCGTATAVSVPCMFSGMPRVDYDEQLASHREGLLDIAKRAGYQ**

3 M09 100.0% 98.9% **LVVGETARAESFSLNGYAKNTNPELSKQDIFNFSQVSSCGTATAVSVPCMFSGMPRVDYDEQLASHREGLLDIAKRAGYQ**

4 M15 59.4% 99.4% **LVVGETARAESFSLNGYAKNTNPELSKQDIFNFSQVSSCGTATAVSVPCMFSGMPRVDYDEQLASHREGLLDIAKRAGYQ**

5 M15b 39.0% 99.5% **--------------------------------------------VSVPCMFSGMPRVDYDEQLASHREGLLDIAKRAGYQ**

6 M12 100.0% 99.5% **LVVGETARAESFSLNGYAKNTNPELSKQDIFNFSQVSSCGTATAVSVPCMFSGMPRVDYDEQLASHREGLLDIAKRAGYQ**

7 SRR3222490 97.1% 99.4% **LVVGETARAESFSLNGYAKNTNPELSKQDIFNFSQVSSCGTATAVSVPCMFSGMPRVDYDEQLASHREGLLDIAKRAGYQ**

8 SRR3227013 97.1% 99.4% **LVVGETARAESFSLNGYAKNTNPELSKQDIFNFSQVSSCGTATAVSVPCMFSGMPRVDYDEQLASHREGLLDIAKRAGYQ**

9 SRR3228488 9.8% 100.0% **--------------------------------------------------------------------------------**

10 SRR3228488b 45.4% 99.6% **--------------------------------------------VSVPCMFSGMPRVDYDEQLASHREGLLDIAKRAGYQ**

11 SRR3228488c 32.8% 99.4% **LVVGETARAESFSLNGYAKNTNPELSKQDIFNFSQVSSCGTATA------------------------------------**

12 SRR3228488d 32.8% 97.8% **LVVGETARAESFSLNGYAKNTNPELSKQDIFNFSQVSSCGTATA------------------------------------**

13 SRR3228565 97.1% 99.4% **LVVGETARAESFSLNGYAKNTNPELSKQDIFNFSQVSSCGTATAVSVPCMFSGMPRVDYDEQLASHREGLLDIAKRAGYQ**

14 A18 100.0% 99.5% **LVVGETARAESFSLNGYAKNTNPELSKQDIFNFSQVSSCGTATAVSVPCMFSGMPRVDYDEQLASHREGLLDIAKRAGYQ**

15 15A1042 9.5% 98.1% **--------------------------------------------------------------------------------**

16 15A1042b 80.3% 99.5% **LVVGETARAESFSLNGYAKNTNPELSKQDIFNFSQVSSCGTATAVSVPCMFSGMPRVDYDEQLASHREGLLDIAKRAGYQ**

17 15A1042c 8.9% 85.7% **--------------------------------------------------------------------------------**

18 15A1042d 40.4% 98.2% **LVVGETARAESFSLNGYAKNTNPELSKQDIFNFSQVSSCGTATAVSVPCMFSGMPRVDYDEQLASHREGLLDIAKRAGYQ**

19 MRSN7133 97.1% 99.4% **LVVGETARAESFSLNGYAKNTNPELSKQDIFNFSQVSSCGTATAVSVPCMFSGMPRVDYDEQLASHREGLLDIAKRAGYQ**

20 MRSN7130 97.1% 99.4% **LVVGETARAESFSLNGYAKNTNPELSKQDIFNFSQVSSCGTATAVSVPCMFSGMPRVDYDEQLASHREGLLDIAKRAGYQ**

21 MRSN7202 97.1% 99.4% **LVVGETARAESFSLNGYAKNTNPELSKQDIFNFSQVSSCGTATAVSVPCMFSGMPRVDYDEQLASHREGLLDIAKRAGYQ**

22 MRSN7224 97.1% 99.4% **LVVGETARAESFSLNGYAKNTNPELSKQDIFNFSQVSSCGTATAVSVPCMFSGMPRVDYDEQLASHREGLLDIAKRAGYQ**

23 OIFC074 7.8% 83.7% **--------------------------------------------------------------------------------**

24 OIFC074b 8.4% 97.8% **--------------------------------------------------------------------------------**

cov pid **321**  **. . : . . . . 4** **400**

1 ATCC19606 100.0% 100.0% **VTWIDNNSGCKGACDRVEQYQIPENLKKKWCKDGECYDDILIDSLKQYLATIAKDDDRPRLIVLHQVGSHGPAYYKRAPE**

2 M06 86.2% 98.9% **VTWIDNNSGCKGACDRVEQYQIPENLKKKWCKDGECYDDILIDSLKQYLATIAKDDDRPRLIVLHQVGSHGPAYYKRAPE**

3 M09 100.0% 98.9% **VTWIDNNSGCKGACDRVEQYQIPENLKKKWCKDGECYDDILIDSLKQYLATIAKDDDRPRLIVLHQVGSHGPAYYKRAPE**

4 M15 59.4% 99.4% **VTWIDN--------------------------------------------------------------------------**

5 M15b 39.0% 99.5% **VTWIDNNSGCKGACDRVEQYQIPENLKKKWCKDGECYDDILIDSLKQYLATIAKDDDRPRLIVLHQVGSHGPAYYKRAPE**

6 M12 100.0% 99.5% **VTWIDNNSGCKGACDRVEQYQIPENLKKKWCKDGECYDDILIDSLKQYLATIAKDDDRPRLIVLHQVGSHGPAYYKRAPE**

7 SRR3222490 97.1% 99.4% **VTWIDNNSGCKGACDRVEQYQIPENLKKKWCKDGECYDDILIDSLKQYLATIAKDDDRPRLIVLHQVGSHGPAYYKRAPE**

8 SRR3227013 97.1% 99.4% **VTWIDNNSGCKGACDRVEQYQIPENLKKKWCKDGECYDDILIDSLKQYLATIAKDDDRPRLIVLHQVGSHGPAYYKRAPE**

9 SRR3228488 9.8% 100.0% **--------------------------------------------------------------------------------**

10 SRR3228488b 45.4% 99.6% **VTWIDNNSGCKGACDRVEQYQIPENLKKKWCKDGECYDDILIDSLKQYLATIAKDDDRPRLIVLHQVGSHGPAYYKRAPE**

11 SRR3228488c 32.8% 99.4% **--------------------------------------------------------------------------------**

12 SRR3228488d 32.8% 97.8% **--------------------------------------------------------------------------------**

13 SRR3228565 97.1% 99.4% **VTWIDNNSGCKGACDRVEQYQIPENLKKKWCKDGECYDDILIDSLKQYLATIAKDDDRPRLIVLHQVGSHGPAYYKRAPE**

14 A18 100.0% 99.5% **VTWIDNNSGCKGACDRVEQYQIPENLKKKWCKDGECYDDILIDSLKQYLATIAKDDDRPRLIVLHQVGSHGPAYYKRAPE**

15 15A1042 9.5% 98.1% **--------------------------------------------------------------------------------**

16 15A1042b 80.3% 99.5% **VTWIDNNSGCKGACDRVEQYQIPENLKKKWCKDGECYDDILIDSLKQYLATIAKDDDRPRLIVLHQVGSHGPAYYKRAPE**

17 15A1042c 8.9% 85.7% **--------------------------------------------------------------------------------**

18 15A1042d 40.4% 98.2% **V-------------------------------------------------------------------------------**

19 MRSN7133 97.1% 99.4% **VTWIDNNSGCKGACDRVEQYQIPENLKKKWCKDGECYDDILIDSLKQYLATIAKDDDRPRLIVLHQVGSHGPAYYKRAPE**

20 MRSN7130 97.1% 99.4% **VTWIDNNSGCKGACDRVEQYQIPENLKKKWCKDGECYDDILIDSLKQYLATIAKDDDRPRLIVLHQVGSHGPAYYKRAPE**

21 MRSN7202 97.1% 99.4% **VTWIDNNSGCKGACDRVEQYQIPENLKKKWCKDGECYDDILIDSLKQYLATIAKDDDRPRLIVLHQVGSHGPAYYKRAPE**

22 MRSN7224 97.1% 99.4% **VTWIDNNSGCKGACDRVEQYQIPENLKKKWCKDGECYDDILIDSLKQYLATIAKDDDRPRLIVLHQVGSHGPAYYKRAPE**

23 OIFC074 7.8% 83.7% **--------------------------------------------------------------------------------**

24 OIFC074b 8.4% 97.8% **--------------------------------------------------------------------------------**

cov pid **401**  **. . . . : . . .** **480**

1 ATCC19606 100.0% 100.0% **AYQPFKPTCDTNAIQGCSQTELLNSYDNTIVYTDHVLSQMINTLKEISKYQTGLWYLSDHGESTGEHGLYLHGSPYAIAP**

2 M06 86.2% 98.9% **AYQPFKPTCDTNAIQGCSQTELLNSYDNTIVYTDHVLSQMINTLKEISKYQTGLWYLSDHGESTGEHGLYLHGSPYAIAP**

3 M09 100.0% 98.9% **AYQPFKPTCDTNAIQGCSQTELLNSYDNTIVYTDHVLSQMINTLKEISKYQTGLWYLSDHGESTGEHGLYLHGSPYAIAP**

4 M15 59.4% 99.4% **--------------------------------------------------------------------------------**

5 M15b 39.0% 99.5% **AYQPFKPTCDTNAIQGCSQTELLNSYDNTIVYTDHVLSQMINTLKEISKYQTGLWYLSDHGESTGEHGLYLHGSPYAIAP**

6 M12 100.0% 99.5% **AYQPFKPTCDTNAIQGCSQTELLNSYDNTIVYTDHVLSQMINTLKEISKYQTGLWYLSDHGESTGEHGLYLHGSPYAIAP**

7 SRR3222490 97.1% 99.4% **AYQPFKPTCDTNAIQGCSQTELLNSYDNTIVYTDHVLSQMINTLKEISKYQTGLWYLSDHGESTGEHGLYLHGSPYAIAP**

8 SRR3227013 97.1% 99.4% **AYQPFKPTCDTNAIQGCSQTELLNSYDNTIVYTDHVLSQMINTLKEISKYQTGLWYLSDHGESTGEHGLYLHGSPYAIAP**

9 SRR3228488 9.8% 100.0% **--------------------------------------------------------------------------------**

10 SRR3228488b 45.4% 99.6% **AYQPFKPTCDTNAIQGCSQTELLNSYDNTIVYTDHVLSQMINTLKEISKYQTGLWYLSDHGESTGEHGLYLHGSPYAIAP**

11 SRR3228488c 32.8% 99.4% **--------------------------------------------------------------------------------**

12 SRR3228488d 32.8% 97.8% **--------------------------------------------------------------------------------**

13 SRR3228565 97.1% 99.4% **AYQPFKPTCDTNAIQGCSQTELLNSYDNTIVYTDHVLSQMINTLKEISKYQTGLWYLSDHGESTGEHGLYLHGSPYAIAP**

14 A18 100.0% 99.5% **AYQPFKPTCDTNAIQGCSQTELLNSYDNTIVYTDHVLSQMINTLKEISKYQTGLWYLSDHGESTGEHGLYLHGSPYAIAP**

15 15A1042 9.5% 98.1% **--------------------------------------------------------------------------------**

16 15A1042b 80.3% 99.5% **AYQPFKPTCDTNAIQGCSQTELLNSYDNTIVYTDHVLSQMI---------------------------------------**

17 15A1042c 8.9% 85.7% **--------------------------------------------------------------------------------**

18 15A1042d 40.4% 98.2% **--------------------------------------------------------------------------------**

19 MRSN7133 97.1% 99.4% **AYQPFKPTCDTNAIQGCSQTELLNSYDNTIVYTDHVLSQMINTLKEISKYQTGLWYLSDHGESTGEHGLYLHGSPYAIAP**

20 MRSN7130 97.1% 99.4% **AYQPFKPTCDTNAIQGCSQTELLNSYDNTIVYTDHVLSQMINTLKEISKYQTGLWYLSDHGESTGEHGLYLHGSPYAIAP**

21 MRSN7202 97.1% 99.4% **AYQPFKPTCDTNAIQGCSQTELLNSYDNTIVYTDHVLSQMINTLKEISKYQTGLWYLSDHGESTGEHGLYLHGSPYAIAP**

22 MRSN7224 97.1% 99.4% **AYQPFKPTCDTNAIQGCSQTELLNSYDNTIVYTDHVLSQMINTLKEISKYQTGLWYLSDHGESTGEHGLYLHGSPYAIAP**

23 OIFC074 7.8% 83.7% **--------------------------------------------------------------------------------**

24 OIFC074b 8.4% 97.8% **--------------------------------------------------------------------------------**

cov pid **481**  **. 5 . . . . ]** **549**

1 ATCC19606 100.0% 100.0% **SQQTHVPMIMWFSESWKQHNLAQVNCLSQQTKQKLSQDNLFPSLLSLLDVKTQVINPQLDMLHSCAHVN**

2 M06 86.2% 98.9% **SQQTHVPMIMWFSESWKQRNLAQVNCLSQQTKQKLSQDNLFPSLLSLLDVKTQVINPQLDMLHSCAHVN**

3 M09 100.0% 98.9% **SQQTHVPMIMWFSESWKQRNLAQVNCLSQQTKQKLSQDNLFPSLLSLLDVKTQVINPQLDMLHSCAHVN**

4 M15 59.4% 99.4% **---------------------------------------------------------------------**

5 M15b 39.0% 99.5% **SQQTHVPMIMWFSESWKQ---------------------------------------------------**

6 M12 100.0% 99.5% **SQQTHVPMIMWFSESWKQRNLAQVNCLSQQTKQKLSQDNLFPSLLSLLDVKTQVINPQLDMLHSCAHVN**

7 SRR3222490 97.1% 99.4% **SQQTHVPMIMWFSESWKQRNLAQVNCLSQQTKQKLSQDNLFPSLLSLLDVKTQVINPQLDMLHSCAHVN**

8 SRR3227013 97.1% 99.4% **SQQTHVPMIMWFSESWKQRNLAQVNCLSQQTKQKLSQDNLFPSLLSLLDVKTQVINPQLDMLHSCAHVN**

9 SRR3228488 9.8% 100.0% **---------------------------------------------------------------------**

10 SRR3228488b 45.4% 99.6% **SQQTHVPMIMWFSESWKQHNLAQVNCLSQQTKQKLSQDNLFPSLLSLLDVKTQ----------------**

11 SRR3228488c 32.8% 99.4% **---------------------------------------------------------------------**

12 SRR3228488d 32.8% 97.8% **---------------------------------------------------------------------**

13 SRR3228565 97.1% 99.4% **SQQTHVPMIMWFSESWKQRNLAQVNCLSQQTKQKLSQDNLFPSLLSLLDVKTQVINPQLDMLHSCAHVN**

14 A18 100.0% 99.5% **SQQTHVPMIMWFSESWKQRNLAQVNCLSQQTKQKLSQDNLFPSLLSLLDVKTQVINPQLDMLHSCAHVN**

15 15A1042 9.5% 98.1% **-----------------QRNLAQVNCLSQQTKQKLSQDNLFPSLLSLLDVKTQVINPQLDMLHSCAHVN**

16 15A1042b 80.3% 99.5% **---------------------------------------------------------------------**

17 15A1042c 8.9% 85.7% **-----------------QRNLAQVNCLSQQTKQKLSQDNLFPSLLSLLDVKTQVVNNKLDMLSQCK---**

18 15A1042d 40.4% 98.2% **---------------------------------------------------------------------**

19 MRSN7133 97.1% 99.4% **SQQTHVPMIMWFSESWKQRNLAQVNCLSQQTKQKLSQDNLFPSLLSLLDVKTQVINPQLDMLHSCAHVN**

20 MRSN7130 97.1% 99.4% **SQQTHVPMIMWFSESWKQRNLAQVNCLSQQTKQKLSQDNLFPSLLSLLDVKTQVINPQLDMLHSCAHVN**

21 MRSN7202 97.1% 99.4% **SQQTHVPMIMWFSESWKQRNLAQVNCLSQQTKQKLSQDNLFPSLLSLLDVKTQVINPQLDMLHSCAHVN**

22 MRSN7224 97.1% 99.4% **SQQTHVPMIMWFSESWKQRNLAQVNCLSQQTKQKLSQDNLFPSLLSLLDVKTQVINPQLDMLHSCAHVN**

23 OIFC074 7.8% 83.7% **-----------------------MNCLSQQTKQKLSQDNLFPSLLSLLDVKTQVVNNKLDMLSQCK---**

24 OIFC074b 8.4% 97.8% **-----------------------MNCLSQQTKQKLSQDNLFPSLLSLLDVKTQVINPQLDMLHSCAHVN**

**Supplementary Figure 1: Multiple sequence alignment (MSA) of the predicted amino acid sequence of PmrC carried by ST19^Pas^ (GC1) isolates and close genomes retrieved from the BV-BRC database compared to the respective gene in *A. baumannii* ATCC 19606.** MSA was created by the A multiple alignment viewer MView hosted by the EMBL-EBI; cov, coverage; pid, percent identity.

cov pid  **1** **[ . . . . : . . .** **80**

1 ATCC19606 100.0% 100.0% **MLNFFSTLRNKQISLFMFNLIIAIWLGAILNIGFYHQVHTLTPYFGVKAILFLAATLVILVATYYAVLQILNWKWTAKIF**

2 M01 86.7% 99.2% **---------------FMFNLIIAIWLGAILNIGFYHQVHTLTPYFGVKAILFLAATLIILVATYYAVLQILNWKWTAKIF**

3 M01b 11.1% 98.4% **--------------------------------------------------------------------------------**

4 M04 89.4% 99.2% **MLNFFSTLRNKQISLFMFNLIIAIWLGAILNIGFYHQVHTLTPYFGVKAILFLAATLIILVATYYAVLQILNWKWTAKIF**

5 M04b 11.1% 98.4% **--------------------------------------------------------------------------------**

6 M05 69.9% 99.2% **--------------------------------------------------------------------------------**

7 M05b 66.7% 98.1% **-----------------------------------------------------------------------NWKWTAKIF**

8 M13 78.5% 99.1% **-----------------------------------------TPYFGVKAILFLAATLIILVATYYAVLQILNWKWTAKIF**

9 M13b 73.0% 98.8% **-----------------------------------------------------------------------NWKWNAKIF**

10 M13c 11.1% 98.4% **--------------------------------------------------------------------------------**

11 M16 100.0% 99.1% **MLNFFSTLRNKQISLFMFNLIIAIWLGAILNIGFYHQVHTLTPYFGVKAILFLAATLIILVATYYAVLQILNWKWTAKIF**

12 M17 100.0% 99.1% **MLNFFSTLRNKQISLFMFNLIIAIWLGAILNIGFYHQVHTLTPYFGVKAILFLAATLIILVATYYAVLQILNWKWTAKIF**

13 M20 27.3% 98.0% **MLNFFSTLRNKQISLFMFNLIIAIWLGAILNIGFYHQVHTLTPYFGVKAILFLAATLIILVATYYAVLQILNWKWTAKIF**

14 M20b 80.3% 98.6% **--------------------------------------------------------------------------------**

15 AC-40 88.0% 99.2% **MLNFFSTLRNKQISLFMFNLIIAIWLGAILNIGFYHQVHTLTPYFGVKAILFLAATLIILVATYYAVLQILNWKWTAKIF**

16 MS14413 97.1% 99.1% **----------------MFNLIIAIWLGAILNIGFYHQVHTLTPYFGVKAILFLAATLIILVATYYAVLQILNWKWTAKIF**

17 SQ093 100.0% 99.1% **MLNFFSTLRNKQISLFMFNLIIAIWLGAILNIGFYHQVHTLTPYFGVKAILFLAATLIILVATYYAVLQILNWKWTAKIF**

18 KAB3 97.1% 99.1% **----------------MFNLIIAIWLGAILNIGFYHQVHTLTPYFGVKAILFLAATLIILVATYYAVLQILNWKWTAKIF**

19 AC-14 100.0% 99.1% **MLNFFSTLRNKQISLFMFNLIIAIWLGAILNIGFYHQVHTLTPYFGVKAILFLAATLIILVATYYAVLQILNWKWTAKIF**

20 AC-45 100.0% 99.1% **MLNFFSTLRNKQISLFMFNLIIAIWLGAILNIGFYHQVHTLTPYFGVKAILFLAATLIILVATYYAVLQILNWKWTAKIF**

21 AC-23 88.0% 99.2% **MLNFFSTLRNKQISLFMFNLIIAIWLGAILNIGFYHQVHTLTPYFGVKAILFLAATLIILVATYYAVLQILNWKWTAKIF**

22 SUH-26-2 100.0% 99.1% **MLNFFSTLRNKQISLFMFNLIIAIWLGAILNIGFYHQVHTLTPYFGVKAILFLAATLIILVATYYAVLQILNWKWTAKIF**

23 SUH-11-2 99.5% 97.8% **MLNFFSTLRNKQISLFMFNLIIAIWLGAILNIGFYHQVHTLTPYFGVKAILFLAATLIILVATYYAVLQILNWKWTAKIF**

24 SUH-11-1 99.5% 97.8% **MLNFFSTLRNKQISLFMFNLIIAIWLGAILNIGFYHQVHTLTPYFGVKAILFLAATLIILVATYYAVLQILNWKWTAKIF**

25 A21 97.1% 99.1% **----------------MFNLIIAIWLGAILNIGFYHQVHTLTPYFGVKAILFLAATLIILVATYYAVLQILNWKWTAKIF**

26 SK044 100.0% 99.1% **MLNFFSTLRNKQISLFMFNLIIAIWLGAILNIGFYHQVHTLTPYFGVKAILFLAATLIILVATYYAVLQILNWKWTAKIF**

27 SK011 100.0% 99.1% **MLNFFSTLRNKQISLFMFNLIIAIWLGAILNIGFYHQVHTLTPYFGVKAILFLAATLIILVATYYAVLQILNWKWTAKIF**

28 SK002 100.0% 99.1% **MLNFFSTLRNKQISLFMFNLIIAIWLGAILNIGFYHQVHTLTPYFGVKAILFLAATLIILVATYYAVLQILNWKWTAKIF**

29 PSU120 100.0% 99.1% **MLNFFSTLRNKQISLFMFNLIIAIWLGAILNIGFYHQVHTLTPYFGVKAILFLAATLIILVATYYAVLQILNWKWTAKIF**

30 KUSSH35 100.0% 99.1% **MLNFFSTLRNKQISLFMFNLIIAIWLGAILNIGFYHQVHTLTPYFGVKAILFLAATLIILVATYYAVLQILNWKWTAKIF**

31 KUSSH15 22.4% 99.2% **MLNFFSTLRNKQISLFMFNLIIAIWLGAILNIGFYHQVHTLTPYFGVKAILFLAATLIILVATYYAVLQILNWKWTAKIF**

32 KUSSH37 82.9% 99.1% **MLNFFSTLRNKQISLFMFNLIIAIWLGAILNIGFYHQVHTLTPYFGVKAILFLAATLIILVATYYAVLQILNWKWTAKIF**

33 KUSSH36 100.0% 99.1% **MLNFFSTLRNKQISLFMFNLIIAIWLGAILNIGFYHQVHTLTPYFGVKAILFLAATLIILVATYYAVLQILNWKWTAKIF**

34 130 97.1% 99.1% **----------------MFNLIIAIWLGAILNIGFYHQVHTLTPYFGVKAILFLAATLIILVATYYAVLQILNWKWTAKIF**

35 Ab34 83.1% 99.1% **MLNFFSTLRNKQISLFMFNLIIAIWLGAILNIGFYHQVHTLTPYFGVKAILFLAATLIILVATYYAVLQILNWKWTAKIF**

36 Ab35 100.0% 99.1% **MLNFFSTLRNKQISLFMFNLIIAIWLGAILNIGFYHQVHTLTPYFGVKAILFLAATLIILVATYYAVLQILNWKWTAKIF**

37 Ab36 100.0% 99.1% **MLNFFSTLRNKQISLFMFNLIIAIWLGAILNIGFYHQVHTLTPYFGVKAILFLAATLIILVATYYAVLQILNWKWTAKIF**

38 Ab38 100.0% 99.1% **MLNFFSTLRNKQISLFMFNLIIAIWLGAILNIGFYHQVHTLTPYFGVKAILFLAATLIILVATYYAVLQILNWKWTAKIF**

39 Ab40 100.0% 99.1% **MLNFFSTLRNKQISLFMFNLIIAIWLGAILNIGFYHQVHTLTPYFGVKAILFLAATLIILVATYYAVLQILNWKWTAKIF**

40 Ab41 100.0% 99.1% **MLNFFSTLRNKQISLFMFNLIIAIWLGAILNIGFYHQVHTLTPYFGVKAILFLAATLIILVATYYAVLQILNWKWTAKIF**

41 Ab15 100.0% 99.1% **MLNFFSTLRNKQISLFMFNLIIAIWLGAILNIGFYHQVHTLTPYFGVKAILFLAATLIILVATYYAVLQILNWKWTAKIF**

42 Ab65 100.0% 99.1% **MLNFFSTLRNKQISLFMFNLIIAIWLGAILNIGFYHQVHTLTPYFGVKAILFLAATLIILVATYYAVLQILNWKWTAKIF**

43 Ab64 100.0% 99.1% **MLNFFSTLRNKQISLFMFNLIIAIWLGAILNIGFYHQVHTLTPYFGVKAILFLAATLIILVATYYAVLQILNWKWTAKIF**

44 AB22 100.0% 99.1% **MLNFFSTLRNKQISLFMFNLIIAIWLGAILNIGFYHQVHTLTPYFGVKAILFLAATLIILVATYYAVLQILNWKWTAKIF**

cov pid  **81**  **. 1 . . . . : .** **160**

1 ATCC19606 100.0% 100.0% **AILLIFIGGFSSYFVNTLGVIISPDQIQNMVQTDVSEVTDLISLRFVLWTIFFVILPIFLITQVKFKQEKVSRLLLKKVF**

2 M01 86.7% 99.2% **AILLIFIGGFSSYFVNTLGVIISPDQIQNMVQTDVSEVTDLISLRFVLWTIFFVILPIFLITQVKFKQEKVSRLLLKKVF**

3 M01b 11.1% 98.4% **--------------------------------------------------------------------------------**

4 M04 89.4% 99.2% **AILLIFIGGFSSYFVNTLGVIISPDQIQNMVQTDVSEVTDLISLRFVLWTIFFVILPIFLITQVKFKQEKVSRLLLKKVF**

5 M04b 11.1% 98.4% **--------------------------------------------------------------------------------**

6 M05 69.9% 99.2% **--------------------------------------------------------------------------------**

7 M05b 66.7% 98.1% **AILLIFIGGFSSYFVNTLGVIISPDQIQNMVQTDVSEFTDLISLRFVLWTVFFVILPIFLITQVKFKQEKASRLLLKKVF**

8 M13 78.5% 99.1% **AILLIFIGGFSSYFVNTLGVIISPDQIQNMVQTDVSEVTDLISLRFVLWTIFFVILPIFLITQVKFKQEKVSRLLLKKVF**

9 M13b 73.0% 98.8% **AILLIFIGGFSSYFVNTLGVIISPDQIQNMVQTDVSEVTDLISLRFVLWTVFFVILPIFLITQVKFKQEKASRLLLKKVF**

10 M13c 11.1% 98.4% **--------------------------------------------------------------------------------**

11 M16 100.0% 99.1% **AILLIFIGGFSSYFVNTLGVIISPDQIQNMVQTDVSEVTDLISLRFVLWTIFFVILPIFLITQVKFKQEKVSRLLLKKVF**

12 M17 100.0% 99.1% **AILLIFIGGFSSYFVNTLGVIISPDQIQNMVQTDVSEVTDLISLRFVLWTIFFVILPIFLITQVKFKQEKVSRLLLKKVF**

13 M20 27.3% 98.0% **AILLIFIGGFSSYFVNTLGVIISPDQIQNMVQTDVSEFTDLISLRFVLWTVFFVILPIFLITQVKFKQEK----------**

14 M20b 80.3% 98.6% **----------------------------NMVQTDVSEFTDLISLRFVLWTVFFVILPIFLITQVKFKQEKVSRLLLKKVF**

15 AC-40 88.0% 99.2% **AILLIFIGGFSSYFVNTLGVIISPDQIQNMVQTDVSEVTDLISLRFVLWTIFFVILPIFLITQVKFKQEKVSRLLLKKVF**

16 MS14413 97.1% 99.1% **AILLIFIGGFSSYFVNTLGVIISPDQIQNMVQTDVSEVTDLISLRFVLWTIFFVILPIFLITQVKFKQEKVSRLLLKKVF**

17 SQ093 100.0% 99.1% **AILLIFIGGFSSYFVNTLGVIISPDQIQNMVQTDVSEVTDLISLRFVLWTIFFVILPIFLITQVKFKQEKVSRLLLKKVF**

18 KAB3 97.1% 99.1% **AILLIFIGGFSSYFVNTLGVIISPDQIQNMVQTDVSEVTDLISLRFVLWTIFFVILPIFLITQVKFKQEKVSRLLLKKVF**

19 AC-14 100.0% 99.1% **AILLIFIGGFSSYFVNTLGVIISPDQIQNMVQTDVSEVTDLISLRFVLWTIFFVILPIFLITQVKFKQEKVSRLLLKKVF**

20 AC-45 100.0% 99.1% **AILLIFIGGFSSYFVNTLGVIISPDQIQNMVQTDVSEVTDLISLRFVLWTIFFVILPIFLITQVKFKQEKVSRLLLKKVF**

21 AC-23 88.0% 99.2% **AILLIFIGGFSSYFVNTLGVIISPDQIQNMVQTDVSEVTDLISLRFVLWTIFFVILPIFLITQVKFKQEKVSRLLLKKVF**

22 SUH-26-2 100.0% 99.1% **AILLIFIGGFSSYFVNTLGVIISPDQIQNMVQTDVSEVTDLISLRFVLWTIFFVILPIFLITQVKFKQEKVSRLLLKKVF**

23 SUH-11-2 99.5% 97.8% **AILLIFIGGFSSYFVNTLGVIISPDQIQNMVQTDVSEVTDLISLRFVLWTIFFVILPIFLITQVKFKQEKVSRLLLKKVF**

24 SUH-11-1 99.5% 97.8% **AILLIFIGGFSSYFVNTLGVIISPDQIQNMVQTDVSEVTDLISLRFVLWTIFFVILPIFLITQVKFKQEKVSRLLLKKVF**

25 A21 97.1% 99.1% **AILLIFIGGFSSYFVNTLGVIISPDQIQNMVQTDVSEVTDLISLRFVLWTIFFVILPIFLITQVKFKQEKVSRLLLKKVF**

26 SK044 100.0% 99.1% **AILLIFIGGFSSYFVNTLGVIISPDQIQNMVQTDVSEVTDLISLRFVLWTIFFVILPIFLITQVKFKQEKVSRLLLKKVF**

27 SK011 100.0% 99.1% **AILLIFIGGFSSYFVNTLGVIISPDQIQNMVQTDVSEVTDLISLRFVLWTIFFVILPIFLITQVKFKQEKVSRLLLKKVF**

28 SK002 100.0% 99.1% **AILLIFIGGFSSYFVNTLGVIISPDQIQNMVQTDVSEVTDLISLRFVLWTIFFVILPIFLITQVKFKQEKVSRLLLKKVF**

29 PSU120 100.0% 99.1% **AILLIFIGGFSSYFVNTLGVIISPDQIQNMVQTDVSEVTDLISLRFVLWTIFFVILPIFLITQVKFKQEKVSRLLLKKVF**

30 KUSSH35 100.0% 99.1% **AILLIFIGGFSSYFVNTLGVIISPDQIQNMVQTDVSEVTDLISLRFVLWTIFFVILPIFLITQVKFKQEKVSRLLLKKVF**

31 KUSSH15 22.4% 99.2% **AILLIFIGGFSSYFVNTLGVIISPDQIQNMVQTDVSEVTDLIS-------------------------------------**

32 KUSSH37 82.9% 99.1% **AILLIFIGGFSSYFVNTLGVIISPDQIQNMVQTDVSEVTDLISLRFVLWTIFFVILPIFLITQVKFKQEKVSRLLLKKVF**

33 KUSSH36 100.0% 99.1% **AILLIFIGGFSSYFVNTLGVIISPDQIQNMVQTDVSEVTDLISLRFVLWTIFFVILPIFLITQVKFKQEKVSRLLLKKVF**

34 130 97.1% 99.1% **AILLIFIGGFSSYFVNTLGVIISPDQIQNMVQTDVSEVTDLISLRFVLWTIFFVILPIFLITQVKFKQEKVSRLLLKKVF**

35 Ab34 83.1% 99.1% **AILLIFIGGFSSYFVNTLGVIISPDQIQNMVQTDVSEVTDLISLRFVLWTIFFVILPIFLITQVKFKQEKVSRLLLKKVF**

36 Ab35 100.0% 99.1% **AILLIFIGGFSSYFVNTLGVIISPDQIQNMVQTDVSEVTDLISLRFVLWTIFFVILPIFLITQVKFKQEKVSRLLLKKVF**

37 Ab36 100.0% 99.1% **AILLIFIGGFSSYFVNTLGVIISPDQIQNMVQTDVSEVTDLISLRFVLWTIFFVILPIFLITQVKFKQEKVSRLLLKKVF**

38 Ab38 100.0% 99.1% **AILLIFIGGFSSYFVNTLGVIISPDQIQNMVQTDVSEVTDLISLRFVLWTIFFVILPIFLITQVKFKQEKVSRLLLKKVF**

39 Ab40 100.0% 99.1% **AILLIFIGGFSSYFVNTLGVIISPDQIQNMVQTDVSEVTDLISLRFVLWTIFFVILPIFLITQVKFKQEKVSRLLLKKVF**

40 Ab41 100.0% 99.1% **AILLIFIGGFSSYFVNTLGVIISPDQIQNMVQTDVSEVTDLISLRFVLWTIFFVILPIFLITQVKFKQEKVSRLLLKKVF**

41 Ab15 100.0% 99.1% **AILLIFIGGFSSYFVNTLGVIISPDQIQNMVQTDVSEVTDLISLRFVLWTIFFVILPIFLITQVKFKQEKVSRLLLKKVF**

42 Ab65 100.0% 99.1% **AILLIFIGGFSSYFVNTLGVIISPDQIQNMVQTDVSEVTDLISLRFVLWTIFFVILPIFLITQVKFKQEKVSRLLLKKVF**

43 Ab64 100.0% 99.1% **AILLIFIGGFSSYFVNTLGVIISPDQIQNMVQTDVSEVTDLISLRFVLWTIFFVILPIFLITQVKFKQEKVSRLLLKKVF**

44 AB22 100.0% 99.1% **AILLIFIGGFSSYFVNTLGVIISPDQIQNMVQTDVSEVTDLISLRFVLWTIFFVILPIFLITQVKFKQEKVSRLLLKKVF**

cov pid **161**  **. . . 2 . . . .** **240**

1 ATCC19606 100.0% 100.0% **SLVASFAVVGVLLFTYYVDFAAIFREHRDLKGMISPQNSISSLMSYYHKKAPKKNLPLVIYGQDAHQVQRVQKNLPKLMI**

2 M01 86.7% 99.2% **SLVASLAVVGVLLFTYYVDFAAIFREHRDLKGMISPQNSISSLMSYYHKKAPKKNLPLVIYGQDAHQVQRVQKNLPKLMI**

3 M01b 11.1% 98.4% **--------------------------------------------------------------------------------**

4 M04 89.4% 99.2% **SLVASLAVVGVLLFTYYVDFAAIFREHRDLKGMISPQNSISSLMSYYHKKAPKKNLPLVIYGQDAHQVQRVQKNLPKLMI**

5 M04b 11.1% 98.4% **--------------------------------------------------------------------------------**

6 M05 69.9% 99.2% **-----FAVVGVLLFTYYVDFAAIFREHRDLKGMISPQNSISSLMSYYHKKAPKKNLPLVIYGQDAHQVQRVQKNLPKLMI**

7 M05b 66.7% 98.1% **SLVASFAVVGVLLFTYYVDFAAIFREHRDLKGMISPQNSISSLMSYYHKKAPKKNLPLVIYGQDAHQVQQVQKNLPKLMI**

8 M13 78.5% 99.1% **SLVASLAVVGVLLFTYYVDFAAIFREHRDLKGMISPQNSISSLMSYYHKKAPKKNLPLVIYGQDAHQVQRVQKNLPKLMI**

9 M13b 73.0% 98.8% **SLVASFAVVGVLLFTYYVDFAAIFREHRDLKGMISPQNSISSLMSYYHKKAPKKNLPLVIYGQDAHQVQQVQKNLPKLMI**

10 M13c 11.1% 98.4% **--------------------------------------------------------------------------------**

11 M16 100.0% 99.1% **SLVASLAVVGVLLFTYYVDFAAIFREHRDLKGMISPQNSISSLMSYYHKKAPKKNLPLVIYGQDAHQVQRVQKNLPKLMI**

12 M17 100.0% 99.1% **SLVASLAVVGVLLFTYYVDFAAIFREHRDLKGMISPQNSISSLMSYYHKKAPKKNLPLVIYGQDAHQVQRVQKNLPKLMI**

13 M20 27.3% 98.0% **--------------------------------------------------------------------------------**

14 M20b 80.3% 98.6% **SLVASLAVVGVLLFTYYVDFAAIFREHRDLKGMISPQNSISSLMSYYHKKAPKKNLPLVIYGQDAHQVQRVQKNLPKLMI**

15 AC-40 88.0% 99.2% **SLVASLAVVGVLLFTYYVDFAAIFREHRDLKGMISPQNSISSLMSYYHKKAPKKNLPLVIYGQDAHQVQRVQKNLPKLMI**

16 MS14413 97.1% 99.1% **SLVASLAVVGVLLFTYYVDFAAIFREHRDLKGMISPQNSISSLMSYYHKKAPKKNLPLVIYGQDAHQVQRVQKNLPKLMI**

17 SQ093 100.0% 99.1% **SLVASLAVVGVLLFTYYVDFAAIFREHRDLKGMISPQNSISSLMSYYHKKAPKKNLPLVIYGQDAHQVQRVQKNLPKLMI**

18 KAB3 97.1% 99.1% **SLVASLAVVGVLLFTYYVDFAAIFREHRDLKGMISPQNSISSLMSYYHKKAPKKNLPLVIYGQDAHQVQRVQKNLPKLMI**

19 AC-14 100.0% 99.1% **SLVASLAVVGVLLFTYYVDFAAIFREHRDLKGMISPQNSISSLMSYYHKKAPKKNLPLVIYGQDAHQVQRVQKNLPKLMI**

20 AC-45 100.0% 99.1% **SLVASLAVVGVLLFTYYVDFAAIFREHRDLKGMISPQNSISSLMSYYHKKAPKKNLPLVIYGQDAHQVQRVQKNLPKLMI**

21 AC-23 88.0% 99.2% **SLVASLAVVGVLLFTYYVDFAAIFREHRDLKGMISPQNSISSLMSYYHKKAPKKNLPLVIYGQDAHQVQRVQKNLPKLMI**

22 SUH-26-2 100.0% 99.1% **SLVASLAVVGVLLFTYYVDFAAIFREHRDLKGMISPQNSISSLMSYYHKKAPKKNLPLVIYGQDAHQVQRVQKNLPKLMI**

23 SUH-11-2 99.5% 97.8% **SLVASLAVVGVLLFTYYVDFAAIFREHRDLKGMISPQNSISSLMSYYHKKAPKKNLPLVIYGQDAHQVQRVQKNLPKLMI**

24 SUH-11-1 99.5% 97.8% **SLVASLAVVGVLLFTYYVDFAAIFREHRDLKGMISPQNSISSLMSYYHKKAPKKNLPLVIYGQDAHQVQRVQKNLPKLMI**

25 A21 97.1% 99.1% **SLVASLAVVGVLLFTYYVDFAAIFREHRDLKGMISPQNSISSLMSYYHKKAPKKNLPLVIYGQDAHQVQRVQKNLPKLMI**

26 SK044 100.0% 99.1% **SLVASLAVVGVLLFTYYVDFAAIFREHRDLKGMISPQNSISSLMSYYHKKAPKKNLPLVIYGQDAHQVQRVQKNLPKLMI**

27 SK011 100.0% 99.1% **SLVASLAVVGVLLFTYYVDFAAIFREHRDLKGMISPQNSISSLMSYYHKKAPKKNLPLVIYGQDAHQVQRVQKNLPKLMI**

28 SK002 100.0% 99.1% **SLVASLAVVGVLLFTYYVDFAAIFREHRDLKGMISPQNSISSLMSYYHKKAPKKNLPLVIYGQDAHQVQRVQKNLPKLMI**

29 PSU120 100.0% 99.1% **SLVASLAVVGVLLFTYYVDFAAIFREHRDLKGMISPQNSISSLMSYYHKKAPKKNLPLVIYGQDAHQVQRVQKNLPKLMI**

30 KUSSH35 100.0% 99.1% **SLVASLAVVGVLLFTYYVDFAAIFREHRDLKGMISPQNSISSLMSYYHKKAPKKNLPLVIYGQDAHQVQRVQKNLPKLMI**

31 KUSSH15 22.4% 99.2% **--------------------------------------------------------------------------------**

32 KUSSH37 82.9% 99.1% **SLVASLAVVGVLLFTYYVDFAAIFREHRDLKGMISPQNSISSLMSYYHKKAPKKNLPLVIYGQDAHQVQRVQKNLPKLMI**

33 KUSSH36 100.0% 99.1% **SLVASLAVVGVLLFTYYVDFAAIFREHRDLKGMISPQNSISSLMSYYHKKAPKKNLPLVIYGQDAHQVQRVQKNLPKLMI**

34 130 97.1% 99.1% **SLVASLAVVGVLLFTYYVDFAAIFREHRDLKGMISPQNSISSLMSYYHKKAPKKNLPLVIYGQDAHQVQRVQKNLPKLMI**

35 Ab34 83.1% 99.1% **SLVASLAVVGVLLFTYYVDFAAIFREHRDLKGMISPQNSISSLMSYYHKKAPKKNLPLVIYGQDAHQVQRVQKNLPKLMI**

36 Ab35 100.0% 99.1% **SLVASLAVVGVLLFTYYVDFAAIFREHRDLKGMISPQNSISSLMSYYHKKAPKKNLPLVIYGQDAHQVQRVQKNLPKLMI**

37 Ab36 100.0% 99.1% **SLVASLAVVGVLLFTYYVDFAAIFREHRDLKGMISPQNSISSLMSYYHKKAPKKNLPLVIYGQDAHQVQRVQKNLPKLMI**

38 Ab38 100.0% 99.1% **SLVASLAVVGVLLFTYYVDFAAIFREHRDLKGMISPQNSISSLMSYYHKKAPKKNLPLVIYGQDAHQVQRVQKNLPKLMI**

39 Ab40 100.0% 99.1% **SLVASLAVVGVLLFTYYVDFAAIFREHRDLKGMISPQNSISSLMSYYHKKAPKKNLPLVIYGQDAHQVQRVQKNLPKLMI**

40 Ab41 100.0% 99.1% **SLVASLAVVGVLLFTYYVDFAAIFREHRDLKGMISPQNSISSLMSYYHKKAPKKNLPLVIYGQDAHQVQRVQKNLPKLMI**

41 Ab15 100.0% 99.1% **SLVASLAVVGVLLFTYYVDFAAIFREHRDLKGMISPQNSISSLMSYYHKKAPKKNLPLVIYGQDAHQVQRVQKNLPKLMI**

42 Ab65 100.0% 99.1% **SLVASLAVVGVLLFTYYVDFAAIFREHRDLKGMISPQNSISSLMSYYHKKAPKKNLPLVIYGQDAHQVQRVQKNLPKLMI**

43 Ab64 100.0% 99.1% **SLVASLAVVGVLLFTYYVDFAAIFREHRDLKGMISPQNSISSLMSYYHKKAPKKNLPLVIYGQDAHQVQRVQKNLPKLMI**

44 AB22 100.0% 99.1% **SLVASLAVVGVLLFTYYVDFAAIFREHRDLKGMISPQNSISSLMSYYHKKAPKKNLPLVIYGQDAHQVQRVQKNLPKLMI**

cov pid **241**  **: . . . . 3 . .** **320**

1 ATCC19606 100.0% 100.0% **LVVGETARAESFSLNGYAKNTNPELSKQDIFNFSQVSSCGTATAVSVPCMFSGMPRVDYNEQLASHREGLLDIAKRAGYQ**

2 M01 86.7% 99.2% **LVVGETARAESFSLNGYAKNTNPELSKQDIFNFSQVSSCGTATAVSVPCMFSGMPRVDYDEQLASHREGLLDIAKRAGYQ**

3 M01b 11.1% 98.4% **--------------------------------------------------------------------------------**

4 M04 89.4% 99.2% **LVVGETARAESFSLNGYAKNTNPELSKQDIFNFSQVSSCGTATAVSVPCMFSGMPRVDYDEQLASHREGLLDIAKRAGYQ**

5 M04b 11.1% 98.4% **--------------------------------------------------------------------------------**

6 M05 69.9% 99.2% **LVVGETARAESFSLNGYAKNTNPELSKQDIFNFSQVSSCGTATAVSVPCMFSGMPRVDYDEQLASHREGLLDIAKRAGYQ**

7 M05b 66.7% 98.1% **LVVGETARAESFSLNGYAKNTNPELSKQDIFNFSQVSSCGTATAVSVPCMFSGMPRVDYDEQLASHREGLLDIAKRAGYQ**

8 M13 78.5% 99.1% **LVVGETARAESFSLNGYAKNTNPELSKQDIFNFSQVSSCGTATAVSVPCMFSGMPRVDYDEQLASHREGLLDIAKRAGYQ**

9 M13b 73.0% 98.8% **LVVGETARAESFSLNGYAKNTNPELSKQDIFNFSQVSSCGTATAVSVPCMFSGMPRVDYDEQLASHREGLLDIAKRAGYQ**

10 M13c 11.1% 98.4% **--------------------------------------------------------------------------------**

11 M16 100.0% 99.1% **LVVGETARAESFSLNGYAKNTNPELSKQDIFNFSQVSSCGTATAVSVPCMFSGMPRVDYDEQLASHREGLLDIAKRAGYQ**

12 M17 100.0% 99.1% **LVVGETARAESFSLNGYAKNTNPELSKQDIFNFSQVSSCGTATAVSVPCMFSGMPRVDYDEQLASHREGLLDIAKRAGYQ**

13 M20 27.3% 98.0% **--------------------------------------------------------------------------------**

14 M20b 80.3% 98.6% **LVVGETARAESFSLNGYAKNTNPELSKQDIFNFSQVSSCGTATAVSVPCMFSGMPRVDYDEQLASHREGLLDIAKRAGYQ**

15 AC-40 88.0% 99.2% **LVVGETARAESFSLNGYAKNTNPELSKQDIFNFSQVSSCGTATAVSVPCMFSGMPRVDYDEQLASHREGLLDIAKRAGYQ**

16 MS14413 97.1% 99.1% **LVVGETARAESFSLNGYAKNTNPELSKQDIFNFSQVSSCGTATAVSVPCMFSGMPRVDYDEQLASHREGLLDIAKRAGYQ**

17 SQ093 100.0% 99.1% **LVVGETARAESFSLNGYAKNTNPELSKQDIFNFSQVSSCGTATAVSVPCMFSGMPRVDYDEQLASHREGLLDIAKRAGYQ**

18 KAB3 97.1% 99.1% **LVVGETARAESFSLNGYAKNTNPELSKQDIFNFSQVSSCGTATAVSVPCMFSGMPRVDYDEQLASHREGLLDIAKRAGYQ**

19 AC-14 100.0% 99.1% **LVVGETARAESFSLNGYAKNTNPELSKQDIFNFSQVSSCGTATAVSVPCMFSGMPRVDYDEQLASHREGLLDIAKRAGYQ**

20 AC-45 100.0% 99.1% **LVVGETARAESFSLNGYAKNTNPELSKQDIFNFSQVSSCGTATAVSVPCMFSGMPRVDYDEQLASHREGLLDIAKRAGYQ**

21 AC-23 88.0% 99.2% **LVVGETARAESFSLNGYAKNTNPELSKQDIFNFSQVSSCGTATAVSVPCMFSGMPRVDYDEQLASHREGLLDIAKRAGYQ**

22 SUH-26-2 100.0% 99.1% **LVVGETARAESFSLNGYAKNTNPELSKQDIFNFSQVSSCGTATAVSVPCMFSGMPRVDYDEQLASHREGLLDIAKRAGYQ**

23 SUH-11-2 99.5% 97.8% **LVVGETARAESFSLNGYAKNTNPELSKQDIFNFSQVSSCGTATAVSVPCMFSGMPRVDYDEQLASHREGLLDIAKRAGYQ**

24 SUH-11-1 99.5% 97.8% **LVVGETARAESFSLNGYAKNTNPELSKQDIFNFSQVSSCGTATAVSVPCMFSGMPRVDYDEQLASHREGLLDIAKRAGYQ**

25 A21 97.1% 99.1% **LVVGETARAESFSLNGYAKNTNPELSKQDIFNFSQVSSCGTATAVSVPCMFSGMPRVDYDEQLASHREGLLDIAKRAGYQ**

26 SK044 100.0% 99.1% **LVVGETARAESFSLNGYAKNTNPELSKQDIFNFSQVSSCGTATAVSVPCMFSGMPRVDYDEQLASHREGLLDIAKRAGYQ**

27 SK011 100.0% 99.1% **LVVGETARAESFSLNGYAKNTNPELSKQDIFNFSQVSSCGTATAVSVPCMFSGMPRVDYDEQLASHREGLLDIAKRAGYQ**

28 SK002 100.0% 99.1% **LVVGETARAESFSLNGYAKNTNPELSKQDIFNFSQVSSCGTATAVSVPCMFSGMPRVDYDEQLASHREGLLDIAKRAGYQ**

29 PSU120 100.0% 99.1% **LVVGETARAESFSLNGYAKNTNPELSKQDIFNFSQVSSCGTATAVSVPCMFSGMPRVDYDEQLASHREGLLDIAKRAGYQ**

30 KUSSH35 100.0% 99.1% **LVVGETARAESFSLNGYAKNTNPELSKQDIFNFSQVSSCGTATAVSVPCMFSGMPRVDYDEQLASHREGLLDIAKRAGYQ**

31 KUSSH15 22.4% 99.2% **--------------------------------------------------------------------------------**

32 KUSSH37 82.9% 99.1% **LVVGETARAESFSLNGYAKNTNPELSKQDIFNFSQVSSCGTATAVSVPCMFSGMPRVDYDEQLASHREGLLDIAKRAGYQ**

33 KUSSH36 100.0% 99.1% **LVVGETARAESFSLNGYAKNTNPELSKQDIFNFSQVSSCGTATAVSVPCMFSGMPRVDYDEQLASHREGLLDIAKRAGYQ**

34 130 97.1% 99.1% **LVVGETARAESFSLNGYAKNTNPELSKQDIFNFSQVSSCGTATAVSVPCMFSGMPRVDYDEQLASHREGLLDIAKRAGYQ**

35 Ab34 83.1% 99.1% **LVVGETARAESFSLNGYAKNTNPELSKQDIFNFSQVSSCGTATAVSVPCMFSGMPRVDYDEQLASHREGLLDIAKRAGYQ**

36 Ab35 100.0% 99.1% **LVVGETARAESFSLNGYAKNTNPELSKQDIFNFSQVSSCGTATAVSVPCMFSGMPRVDYDEQLASHREGLLDIAKRAGYQ**

37 Ab36 100.0% 99.1% **LVVGETARAESFSLNGYAKNTNPELSKQDIFNFSQVSSCGTATAVSVPCMFSGMPRVDYDEQLASHREGLLDIAKRAGYQ**

38 Ab38 100.0% 99.1% **LVVGETARAESFSLNGYAKNTNPELSKQDIFNFSQVSSCGTATAVSVPCMFSGMPRVDYDEQLASHREGLLDIAKRAGYQ**

39 Ab40 100.0% 99.1% **LVVGETARAESFSLNGYAKNTNPELSKQDIFNFSQVSSCGTATAVSVPCMFSGMPRVDYDEQLASHREGLLDIAKRAGYQ**

40 Ab41 100.0% 99.1% **LVVGETARAESFSLNGYAKNTNPELSKQDIFNFSQVSSCGTATAVSVPCMFSGMPRVDYDEQLASHREGLLDIAKRAGYQ**

41 Ab15 100.0% 99.1% **LVVGETARAESFSLNGYAKNTNPELSKQDIFNFSQVSSCGTATAVSVPCMFSGMPRVDYDEQLASHREGLLDIAKRAGYQ**

42 Ab65 100.0% 99.1% **LVVGETARAESFSLNGYAKNTNPELSKQDIFNFSQVSSCGTATAVSVPCMFSGMPRVDYDEQLASHREGLLDIAKRAGYQ**

43 Ab64 100.0% 99.1% **LVVGETARAESFSLNGYAKNTNPELSKQDIFNFSQVSSCGTATAVSVPCMFSGMPRVDYDEQLASHREGLLDIAKRAGYQ**

44 AB22 100.0% 99.1% **LVVGETARAESFSLNGYAKNTNPELSKQDIFNFSQVSSCGTATAVSVPCMFSGMPRVDYDEQLASHREGLLDIAKRAGYQ**

cov pid **321**  **. . : . . . . 4** **400**

1 ATCC19606 100.0% 100.0% **VTWIDNNSGCKGACDRVEQYQIPENLKKKWCKDGECYDDILIDSLKQYLATIAKDDDRPRLIVLHQVGSHGPAYYKRAPE**

2 M01 86.7% 99.2% **VTWIDNNSGCKGACDRVEQYQIPENLKKKWCKDGECYDDILIDSLKQYLSTIAKDDDRPRLIVLHQVGSHGPAYYKRAPE**

3 M01b 11.1% 98.4% **--------------------------------------------------------------------------------**

4 M04 89.4% 99.2% **VTWIDNNSGCKGACDRVEQYQIPENLKKKWCKDGECYDDILIDSLKQYLSTIAKDDDRPRLIVLHQVGSHGPAYYKRAPE**

5 M04b 11.1% 98.4% **--------------------------------------------------------------------------------**

6 M05 69.9% 99.2% **VTWIDNNSGCKGACDRVEQYQIPENLKKKWCKDGECYDDILIDSLKQYLSTIAKDDDRPRLIVLHQVGSHGPAYYKRAPE**

7 M05b 66.7% 98.1% **VTWIDNNSGCKGACDRVEQYQIPENLKKKWCKDGECYDDILIDSLKQYLATIAKDDDRPRLIVLHQVGSHGPAYYKRAPE**

8 M13 78.5% 99.1% **VTWIDNNSGCKGACDRVEQYQIPENLKKKWCKDGECYDDILIDSLKQYLSTIAKDDDRPRLIVLHQVGSHGPAYYKRAPE**

9 M13b 73.0% 98.8% **VTWIDNNSGCKGACDRVEQYQIPENLKKKWCKDGECYDDILIDSLKQYLATIAKDDDRPRLIVLHQVGSHGPAYYKRAPE**

10 M13c 11.1% 98.4% **--------------------------------------------------------------------------------**

11 M16 100.0% 99.1% **VTWIDNNSGCKGACDRVEQYQIPENLKKKWCKDGECYDDILIDSLKQYLSTIAKDDDRPRLIVLHQVGSHGPAYYKRAPE**

12 M17 100.0% 99.1% **VTWIDNNSGCKGACDRVEQYQIPENLKKKWCKDGECYDDILIDSLKQYLSTIAKDDDRPRLIVLHQVGSHGPAYYKRAPE**

13 M20 27.3% 98.0% **--------------------------------------------------------------------------------**

14 M20b 80.3% 98.6% **VTWIDNNSGCKGACDRVEQYQIPENLKKKWCKDGECYDDILIDSLKQYLSTIAKDDDRPRLIVLHQVGSHGPAYYKRAPE**

15 AC-40 88.0% 99.2% **VTWIDNNSGCKGACDRVEQYQIPENLKKKWCKDGECYDDILIDSLKQYLSTIAKDDDRPRLIVLHQVGSHGPAYYKRAPE**

16 MS14413 97.1% 99.1% **VTWIDNNSGCKGACDRVEQYQIPENLKKKWCKDGECYDDILIDSLKQYLSTIAKDDDRPRLIVLHQVGSHGPAYYKRAPE**

17 SQ093 100.0% 99.1% **VTWIDNNSGCKGACDRVEQYQIPENLKKKWCKDGECYDDILIDSLKQYLSTIAKDDDRPRLIVLHQVGSHGPAYYKRAPE**

18 KAB3 97.1% 99.1% **VTWIDNNSGCKGACDRVEQYQIPENLKKKWCKDGECYDDILIDSLKQYLSTIAKDDDRPRLIVLHQVGSHGPAYYKRAPE**

19 AC-14 100.0% 99.1% **VTWIDNNSGCKGACDRVEQYQIPENLKKKWCKDGECYDDILIDSLKQYLSTIAKDDDRPRLIVLHQVGSHGPAYYKRAPE**

20 AC-45 100.0% 99.1% **VTWIDNNSGCKGACDRVEQYQIPENLKKKWCKDGECYDDILIDSLKQYLSTIAKDDDRPRLIVLHQVGSHGPAYYKRAPE**

21 AC-23 88.0% 99.2% **VTWIDNNSGCKGACDRVEQYQIPENLKKKWCKDGECYDDILIDSLKQYLSTIAKDDDRPRLIVLHQVGSHGPAYYKRAPE**

22 SUH-26-2 100.0% 99.1% **VTWIDNNSGCKGACDRVEQYQIPENLKKKWCKDGECYDDILIDSLKQYLSTIAKDDDRPRLIVLHQVGSHGPAYYKRAPE**

23 SUH-11-2 99.5% 97.8% **VTWIDNNSGCKGACDRVEQYQIPENLKKKWCKDGECYDDILIDSLKQYLSTIAKDDDRPRLIVLHQVGSHGPAYYKRAPE**

24 SUH-11-1 99.5% 97.8% **VTWIDNNSGCKGACDRVEQYQIPENLKKKWCKDGECYDDILIDSLKQYLSTIAKDDDRPRLIVLHQVGSHGPAYYKRAPE**

25 A21 97.1% 99.1% **VTWIDNNSGCKGACDRVEQYQIPENLKKKWCKDGECYDDILIDSLKQYLSTIAKDDDRPRLIVLHQVGSHGPAYYKRAPE**

26 SK044 100.0% 99.1% **VTWIDNNSGCKGACDRVEQYQIPENLKKKWCKDGECYDDILIDSLKQYLSTIAKDDDRPRLIVLHQVGSHGPAYYKRAPE**

27 SK011 100.0% 99.1% **VTWIDNNSGCKGACDRVEQYQIPENLKKKWCKDGECYDDILIDSLKQYLSTIAKDDDRPRLIVLHQVGSHGPAYYKRAPE**

28 SK002 100.0% 99.1% **VTWIDNNSGCKGACDRVEQYQIPENLKKKWCKDGECYDDILIDSLKQYLSTIAKDDDRPRLIVLHQVGSHGPAYYKRAPE**

29 PSU120 100.0% 99.1% **VTWIDNNSGCKGACDRVEQYQIPENLKKKWCKDGECYDDILIDSLKQYLSTIAKDDDRPRLIVLHQVGSHGPAYYKRAPE**

30 KUSSH35 100.0% 99.1% **VTWIDNNSGCKGACDRVEQYQIPENLKKKWCKDGECYDDILIDSLKQYLSTIAKDDDRPRLIVLHQVGSHGPAYYKRAPE**

31 KUSSH15 22.4% 99.2% **--------------------------------------------------------------------------------**

32 KUSSH37 82.9% 99.1% **VTWIDNNSGCKGACDRVEQYQIPENLKKKWCKDGECYDDILIDSLKQYLSTIAKDDDRPRLIVLHQVGSHGPAYYKRAPE**

33 KUSSH36 100.0% 99.1% **VTWIDNNSGCKGACDRVEQYQIPENLKKKWCKDGECYDDILIDSLKQYLSTIAKDDDRPRLIVLHQVGSHGPAYYKRAPE**

34 130 97.1% 99.1% **VTWIDNNSGCKGACDRVEQYQIPENLKKKWCKDGECYDDILIDSLKQYLSTIAKDDDRPRLIVLHQVGSHGPAYYKRAPE**

35 Ab34 83.1% 99.1% **VTWIDNNSGCKGACDRVEQYQIPENLKKKWCKDGECYDDILIDSLKQYLSTIAKDDDRPRLIVLHQVGSHGPAYYKRAPE**

36 Ab35 100.0% 99.1% **VTWIDNNSGCKGACDRVEQYQIPENLKKKWCKDGECYDDILIDSLKQYLSTIAKDDDRPRLIVLHQVGSHGPAYYKRAPE**

37 Ab36 100.0% 99.1% **VTWIDNNSGCKGACDRVEQYQIPENLKKKWCKDGECYDDILIDSLKQYLSTIAKDDDRPRLIVLHQVGSHGPAYYKRAPE**

38 Ab38 100.0% 99.1% **VTWIDNNSGCKGACDRVEQYQIPENLKKKWCKDGECYDDILIDSLKQYLSTIAKDDDRPRLIVLHQVGSHGPAYYKRAPE**

39 Ab40 100.0% 99.1% **VTWIDNNSGCKGACDRVEQYQIPENLKKKWCKDGECYDDILIDSLKQYLSTIAKDDDRPRLIVLHQVGSHGPAYYKRAPE**

40 Ab41 100.0% 99.1% **VTWIDNNSGCKGACDRVEQYQIPENLKKKWCKDGECYDDILIDSLKQYLSTIAKDDDRPRLIVLHQVGSHGPAYYKRAPE**

41 Ab15 100.0% 99.1% **VTWIDNNSGCKGACDRVEQYQIPENLKKKWCKDGECYDDILIDSLKQYLSTIAKDDDRPRLIVLHQVGSHGPAYYKRAPE**

42 Ab65 100.0% 99.1% **VTWIDNNSGCKGACDRVEQYQIPENLKKKWCKDGECYDDILIDSLKQYLSTIAKDDDRPRLIVLHQVGSHGPAYYKRAPE**

43 Ab64 100.0% 99.1% **VTWIDNNSGCKGACDRVEQYQIPENLKKKWCKDGECYDDILIDSLKQYLSTIAKDDDRPRLIVLHQVGSHGPAYYKRAPE**

44 AB22 100.0% 99.1% **VTWIDNNSGCKGACDRVEQYQIPENLKKKWCKDGECYDDILIDSLKQYLSTIAKDDDRPRLIVLHQVGSHGPAYYKRAPE**

cov pid **401**  **. . . . : . . .** **480**

1 ATCC19606 100.0% 100.0% **AYQPFKPTCDTNAIQGCSQTELLNSYDNTIVYTDHVLSQMINTLKEISKYQTGLWYLSDHGESTGEHGLYLHGSPYAIAP**

2 M01 86.7% 99.2% **AYQPFKPTCDTNAIQGCSQTELLNSYDNTIVYTDHVLSQMINTLKEISKYQTGLWYLSDHGESTGEHGLYLHGSPYAIAP**

3 M01b 11.1% 98.4% **--------------------------------------------------------------------------------**

4 M04 89.4% 99.2% **AYQPFKPTCDTNAIQGCSQTELLNSYDNTIVYTDHVLSQMINTLKEISKYQTGLWYLSDHGESTGEHGLYLHGSPYAIAP**

5 M04b 11.1% 98.4% **--------------------------------------------------------------------------------**

6 M05 69.9% 99.2% **AYQPFKPTCDTNAIQGCSQTELLNSYDNTIVYTDHVLSQMINTLKEISKYQTGLWYLSDHGESTGEHGLYLHGSPYAIAP**

7 M05b 66.7% 98.1% **AYQPFKPTCDTNAIQGCSQPELLNSYDNTIVYTDHVV-------------------------------------------**

8 M13 78.5% 99.1% **AYQPFKPTCDTNAIQGCSQTELLNSYDNTIVYTDHVLSQMINTLKEISKYQTGLWYLSDHGESTGEHGLYLH--------**

9 M13b 73.0% 98.8% **AYQPFKPTCDTNAIQGCSQTELLNSYDNTIVYTDHVLSQMINTLKEISKYQTGLWYLSDHGESTGEHGLYLH--------**

10 M13c 11.1% 98.4% **--------------------------------------------------------------------------------**

11 M16 100.0% 99.1% **AYQPFKPTCDTNAIQGCSQTELLNSYDNTIVYTDHVLSQMINTLKEISKYQTGLWYLSDHGESTGEHGLYLHGSPYAIAP**

12 M17 100.0% 99.1% **AYQPFKPTCDTNAIQGCSQTELLNSYDNTIVYTDHVLSQMINTLKEISKYQTGLWYLSDHGESTGEHGLYLHGSPYAIAP**

13 M20 27.3% 98.0% **--------------------------------------------------------------------------------**

14 M20b 80.3% 98.6% **AYQPFKPTCDTNAIQGCSQTELLNSYDNTIVYTDHVLSQMINTLKEISKYQTGLWYLSDHGESTGEHGLYLHGSPYAIAP**

15 AC-40 88.0% 99.2% **AYQPFKPTCDTNAIQGCSQTELLNSYDNTIVYTDHVLSQMINTLKEISKYQTGLWYLSDHGESTGEHGLYLHGSPYAIAP**

16 MS14413 97.1% 99.1% **AYQPFKPTCDTNAIQGCSQTELLNSYDNTIVYTDHVLSQMINTLKEISKYQTGLWYLSDHGESTGEHGLYLHGSPYAIAP**

17 SQ093 100.0% 99.1% **AYQPFKPTCDTNAIQGCSQTELLNSYDNTIVYTDHVLSQMINTLKEISKYQTGLWYLSDHGESTGEHGLYLHGSPYAIAP**

18 KAB3 97.1% 99.1% **AYQPFKPTCDTNAIQGCSQTELLNSYDNTIVYTDHVLSQMINTLKEISKYQTGLWYLSDHGESTGEHGLYLHGSPYAIAP**

19 AC-14 100.0% 99.1% **AYQPFKPTCDTNAIQGCSQTELLNSYDNTIVYTDHVLSQMINTLKEISKYQTGLWYLSDHGESTGEHGLYLHGSPYAIAP**

20 AC-45 100.0% 99.1% **AYQPFKPTCDTNAIQGCSQTELLNSYDNTIVYTDHVLSQMINTLKEISKYQTGLWYLSDHGESTGEHGLYLHGSPYAIAP**

21 AC-23 88.0% 99.2% **AYQPFKPTCDTNAIQGCSQTELLNSYDNTIVYTDHVLSQMINTLKEISKYQTGLWYLSDHGESTGEHGLYLHGSPYAIAP**

22 SUH-26-2 100.0% 99.1% **AYQPFKPTCDTNAIQGCSQTELLNSYDNTIVYTDHVLSQMINTLKEISKYQTGLWYLSDHGESTGEHGLYLHGSPYAIAP**

23 SUH-11-2 99.5% 97.8% **AYQPFKPTCDTNAIQGCSQTELLNSYDNTIVYTDHVLSQMINTLKEISKYQTGLWYLSDHGESTGEHGLYLHGSPYAIAP**

24 SUH-11-1 99.5% 97.8% **AYQPFKPTCDTNAIQGCSQTELLNSYDNTIVYTDHVLSQMINTLKEISKYQTGLWYLSDHGESTGEHGLYLHGSPYAIAP**

25 A21 97.1% 99.1% **AYQPFKPTCDTNAIQGCSQTELLNSYDNTIVYTDHVLSQMINTLKEISKYQTGLWYLSDHGESTGEHGLYLHGSPYAIAP**

26 SK044 100.0% 99.1% **AYQPFKPTCDTNAIQGCSQTELLNSYDNTIVYTDHVLSQMINTLKEISKYQTGLWYLSDHGESTGEHGLYLHGSPYAIAP**

27 SK011 100.0% 99.1% **AYQPFKPTCDTNAIQGCSQTELLNSYDNTIVYTDHVLSQMINTLKEISKYQTGLWYLSDHGESTGEHGLYLHGSPYAIAP**

28 SK002 100.0% 99.1% **AYQPFKPTCDTNAIQGCSQTELLNSYDNTIVYTDHVLSQMINTLKEISKYQTGLWYLSDHGESTGEHGLYLHGSPYAIAP**

29 PSU120 100.0% 99.1% **AYQPFKPTCDTNAIQGCSQTELLNSYDNTIVYTDHVLSQMINTLKEISKYQTGLWYLSDHGESTGEHGLYLHGSPYAIAP**

30 KUSSH35 100.0% 99.1% **AYQPFKPTCDTNAIQGCSQTELLNSYDNTIVYTDHVLSQMINTLKEISKYQTGLWYLSDHGESTGEHGLYLHGSPYAIAP**

31 KUSSH15 22.4% 99.2% **--------------------------------------------------------------------------------**

32 KUSSH37 82.9% 99.1% **AYQPFKPTCDTNAIQGCSQTELLNSYDNTIVYTDHVLSQMINTLKEISKYQTGLW-------------------------**

33 KUSSH36 100.0% 99.1% **AYQPFKPTCDTNAIQGCSQTELLNSYDNTIVYTDHVLSQMINTLKEISKYQTGLWYLSDHGESTGEHGLYLHGSPYAIAP**

34 130 97.1% 99.1% **AYQPFKPTCDTNAIQGCSQTELLNSYDNTIVYTDHVLSQMINTLKEISKYQTGLWYLSDHGESTGEHGLYLHGSPYAIAP**

35 Ab34 83.1% 99.1% **AYQPFKPTCDTNAIQGCSQTELLNSYDNTIVYTDHVLSQMINTLKEISKYQTGLWY------------------------**

36 Ab35 100.0% 99.1% **AYQPFKPTCDTNAIQGCSQTELLNSYDNTIVYTDHVLSQMINTLKEISKYQTGLWYLSDHGESTGEHGLYLHGSPYAIAP**

37 Ab36 100.0% 99.1% **AYQPFKPTCDTNAIQGCSQTELLNSYDNTIVYTDHVLSQMINTLKEISKYQTGLWYLSDHGESTGEHGLYLHGSPYAIAP**

38 Ab38 100.0% 99.1% **AYQPFKPTCDTNAIQGCSQTELLNSYDNTIVYTDHVLSQMINTLKEISKYQTGLWYLSDHGESTGEHGLYLHGSPYAIAP**

39 Ab40 100.0% 99.1% **AYQPFKPTCDTNAIQGCSQTELLNSYDNTIVYTDHVLSQMINTLKEISKYQTGLWYLSDHGESTGEHGLYLHGSPYAIAP**

40 Ab41 100.0% 99.1% **AYQPFKPTCDTNAIQGCSQTELLNSYDNTIVYTDHVLSQMINTLKEISKYQTGLWYLSDHGESTGEHGLYLHGSPYAIAP**

41 Ab15 100.0% 99.1% **AYQPFKPTCDTNAIQGCSQTELLNSYDNTIVYTDHVLSQMINTLKEISKYQTGLWYLSDHGESTGEHGLYLHGSPYAIAP**

42 Ab65 100.0% 99.1% **AYQPFKPTCDTNAIQGCSQTELLNSYDNTIVYTDHVLSQMINTLKEISKYQTGLWYLSDHGESTGEHGLYLHGSPYAIAP**

43 Ab64 100.0% 99.1% **AYQPFKPTCDTNAIQGCSQTELLNSYDNTIVYTDHVLSQMINTLKEISKYQTGLWYLSDHGESTGEHGLYLHGSPYAIAP**

44 AB22 100.0% 99.1% **AYQPFKPTCDTNAIQGCSQTELLNSYDNTIVYTDHVLSQMINTLKEISKYQTGLWYLSDHGESTGEHGLYLHGSPYAIAP**

cov pid **481**  **. 5 . . . . ]** **549**

1 ATCC19606 100.0% 100.0% **SQQTHVPMIMWFSESWKQHNLAQVNCLSQQTKQKLSQDNLFPSLLSLLDVKTQVINPQLDMLHSCAHVN**

2 M01 86.7% 99.2% **SQQTHVPMIMW----------------------------------------------------------**

3 M01b 11.1% 98.4% **--------IMWFSESWKQHNLAQVNCLSQQTKQKLSQDNLFPSLLSLLDVTTQVINPQLDMLHSCAHVN**

4 M04 89.4% 99.2% **SQQTHVPMIMW----------------------------------------------------------**

5 M04b 11.1% 98.4% **--------IMWFSESWKQHNLAQVNCLSQQTKQKLSQDNLFPSLLSLLDVTTQVINPQLDMLHSCAHVN**

6 M05 69.9% 99.2% **SQQTHVPMIMWFSESWKQHNLAQVNCLSQQTKQKLSQDNLFPSLLSLLDVTTQVINPQLDMLHSCAHVN**

7 M05b 66.7% 98.1% **---------------------------------------------------------------------**

8 M13 78.5% 99.1% **---------------------------------------------------------------------**

9 M13b 73.0% 98.8% **---------------------------------------------------------------------**

10 M13c 11.1% 98.4% **--------IMWFSESWKQHNLAQVNCLSQQTKQKLSQDNLFPSLLSLLDVTTQVINPQLDMLHSCAHVN**

11 M16 100.0% 99.1% **SQQTHVPMIMWFSESWKQHNLAQVNCLSQQTKQKLSQDNLFPSLLSLLDVTTQVINPQLDMLHSCAHVN**

12 M17 100.0% 99.1% **SQQTHVPMIMWFSESWKQHNLAQVNCLSQQTKQKLSQDNLFPSLLSLLDVTTQVINPQLDMLHSCAHVN**

13 M20 27.3% 98.0% **---------------------------------------------------------------------**

14 M20b 80.3% 98.6% **SQQTHVPMIMWFSESWKQHNLAQVNCLSQQTKQKLSQDNLFPSLLSLLDVTTQVINPQLDMLHSCAHVN**

15 AC-40 88.0% 99.2% **SQQ------------------------------------------------------------------**

16 MS14413 97.1% 99.1% **SQQTHVPMIMWFSESWKQHNLAQVNCLSQQTKQKLSQDNLFPSLLSLLDVTTQVINPQLDMLHSCAHVN**

17 SQ093 100.0% 99.1% **SQQTHVPMIMWFSESWKQHNLAQVNCLSQQTKQKLSQDNLFPSLLSLLDVTTQVINPQLDMLHSCAHVN**

18 KAB3 97.1% 99.1% **SQQTHVPMIMWFSESWKQHNLAQVNCLSQQTKQKLSQDNLFPSLLSLLDVTTQVINPQLDMLHSCAHVN**

19 AC-14 100.0% 99.1% **SQQTHVPMIMWFSESWKQHNLAQVNCLSQQTKQKLSQDNLFPSLLSLLDVTTQVINPQLDMLHSCAHVN**

20 AC-45 100.0% 99.1% **SQQTHVPMIMWFSESWKQHNLAQVNCLSQQTKQKLSQDNLFPSLLSLLDVTTQVINPQLDMLHSCAHVN**

21 AC-23 88.0% 99.2% **SQQ------------------------------------------------------------------**

22 SUH-26-2 100.0% 99.1% **SQQTHVPMIMWFSESWKQHNLAQVNCLSQQTKQKLSQDNLFPSLLSLLDVTTQVINPQLDMLHSCAHVN**

23 SUH-11-2 99.5% 97.8% **SQQTHVPMIMWFSESWKQHNLAQVNCLSQQTKQKLSQDNLFPSLLSLLDVKTXVVNNKLNMLSQCK---**

24 SUH-11-1 99.5% 97.8% **SQQTHVPMIMWFSESWKQHNLAQVNCLSQQTKQKLSQDNLFPSLLSLLDVKTXVVNNKLNMLSQCK---**

25 A21 97.1% 99.1% **SQQTHVPMIMWFSESWKQHNLAQVNCLSQQTKQKLSQDNLFPSLLSLLDVTTQVINPQLDMLHSCAHVN**

26 SK044 100.0% 99.1% **SQQTHVPMIMWFSESWKQHNLAQVNCLSQQTKQKLSQDNLFPSLLSLLDVTTQVINPQLDMLHSCAHVN**

27 SK011 100.0% 99.1% **SQQTHVPMIMWFSESWKQHNLAQVNCLSQQTKQKLSQDNLFPSLLSLLDVTTQVINPQLDMLHSCAHVN**

28 SK002 100.0% 99.1% **SQQTHVPMIMWFSESWKQHNLAQVNCLSQQTKQKLSQDNLFPSLLSLLDVTTQVINPQLDMLHSCAHVN**

29 PSU120 100.0% 99.1% **SQQTHVPMIMWFSESWKQHNLAQVNCLSQQTKQKLSQDNLFPSLLSLLDVTTQVINPQLDMLHSCAHVN**

30 KUSSH35 100.0% 99.1% **SQQTHVPMIMWFSESWKQHNLAQVNCLSQQTKQKLSQDNLFPSLLSLLDVTTQVINPQLDMLHSCAHVN**

31 KUSSH15 22.4% 99.2% **---------------------------------------------------------------------**

32 KUSSH37 82.9% 99.1% **---------------------------------------------------------------------**

33 KUSSH36 100.0% 99.1% **SQQTHVPMIMWFSESWKQHNLAQVNCLSQQTKQKLSQDNLFPSLLSLLDVTTQVINPQLDMLHSCAHVN**

34 130 97.1% 99.1% **SQQTHVPMIMWFSESWKQHNLAQVNCLSQQTKQKLSQDNLFPSLLSLLDVTTQVINPQLDMLHSCAHVN**

35 Ab34 83.1% 99.1% **---------------------------------------------------------------------**

36 Ab35 100.0% 99.1% **SQQTHVPMIMWFSESWKQHNLAQVNCLSQQTKQKLSQDNLFPSLLSLLDVTTQVINPQLDMLHSCAHVN**

37 Ab36 100.0% 99.1% **SQQTHVPMIMWFSESWKQHNLAQVNCLSQQTKQKLSQDNLFPSLLSLLDVTTQVINPQLDMLHSCAHVN**

38 Ab38 100.0% 99.1% **SQQTHVPMIMWFSESWKQHNLAQVNCLSQQTKQKLSQDNLFPSLLSLLDVTTQVINPQLDMLHSCAHVN**

39 Ab40 100.0% 99.1% **SQQTHVPMIMWFSESWKQHNLAQVNCLSQQTKQKLSQDNLFPSLLSLLDVTTQVINPQLDMLHSCAHVN**

40 Ab41 100.0% 99.1% **SQQTHVPMIMWFSESWKQHNLAQVNCLSQQTKQKLSQDNLFPSLLSLLDVTTQVINPQLDMLHSCAHVN**

41 Ab15 100.0% 99.1% **SQQTHVPMIMWFSESWKQHNLAQVNCLSQQTKQKLSQDNLFPSLLSLLDVTTQVINPQLDMLHSCAHVN**

42 Ab65 100.0% 99.1% **SQQTHVPMIMWFSESWKQHNLAQVNCLSQQTKQKLSQDNLFPSLLSLLDVTTQVINPQLDMLHSCAHVN**

43 Ab64 100.0% 99.1% **SQQTHVPMIMWFSESWKQHNLAQVNCLSQQTKQKLSQDNLFPSLLSLLDVTTQVINPQLDMLHSCAHVN**

44 AB22 100.0% 99.1% **SQQTHVPMIMWFSESWKQHNLAQVNCLSQQTKQKLSQDNLFPSLLSLLDVTTQVINPQLDMLHSCAHVN**

**Supplementary Figure 2: Multiple sequence alignment (MSA) of the predicted amino acid sequence of PmrC carried by ST2^Pas^ and ST570^Pas^ (GC2) and close genomes retrieved from the BV-BRC database compared to the respective gene in *A. baumannii* ATCC 19606.** MSA was created by the A multiple alignment viewer MView hosted by the EMBL-EBI; cov, coverage; pid, percent identity.

cov pid  **1** **[ . . . . : . . .** **80**

1 ATCC19606 100.0% 100.0% **MLNFFSTLRNKQISLFMFNLIIAIWLGAILNIGFYHQVHTLTPYFGVKAILFLAATLVILVATYYAVLQILNWKWTAKIF**

2 M03 84.9% 99.1% **--------------------------------------------------------------------------------**

3 M14 97.1% 98.9% **----------------MFNLIIAIWLGSILNIGFYHTVHTLTPYFGVKAILFLAATLVILVATYYAVLQILNWKWTAKIF**

4 AB_1649-8 97.1% 99.2% **----------------MFNLIIAIWLGAILNIGFYHQVHTLTPYFGVKAILFLAATLVILVATYYAVLQILNWKWTAKIF**

5 AB_1650-8 97.1% 99.2% **----------------MFNLIIAIWLGAILNIGFYHQVHTLTPYFGVKAILFLAATLVILVATYYAVLQILNWKWTAKIF**

6 UV_1036 97.1% 99.2% **----------------MFNLIIAIWLGAILNIGFYHQVHTLTPYFGVKAILFLAATLVILVATYYAVLQILNWKWTAKIF**

7 259_an 97.1% 99.2% **----------------MFNLIIAIWLGAILNIGFYHQVHTLTPYFGVKAILFLAATLVILVATYYAVLQILNWKWTAKIF**

8 276_ax 97.1% 99.2% **----------------MFNLIIAIWLGAILNIGFYHQVHTLTPYFGVKAILFLAATLVILVATYYAVLQILNWKWTAKIF**

9 PT061 100.0% 99.3% **MLNFFSTLRNKQISLFMFNLIIAIWLGAILNIGFYHQVHTLTPYFGVKAILFLAATLVILVATYYAVLQILNWKWTAKIF**

10 PT003 100.0% 99.3% **MLNFFSTLRNKQISLFMFNLIIAIWLGAILNIGFYHQVHTLTPYFGVKAILFLAATLVILVATYYAVLQILNWKWTAKIF**

11 PSU068 100.0% 99.3% **MLNFFSTLRNKQISLFMFNLIIAIWLGAILNIGFYHQVHTLTPYFGVKAILFLAATLVILVATYYAVLQILNWKWTAKIF**

12 PSU073 100.0% 99.3% **MLNFFSTLRNKQISLFMFNLIIAIWLGAILNIGFYHQVHTLTPYFGVKAILFLAATLVILVATYYAVLQILNWKWTAKIF**

13 CCBH26501 97.1% 90.6% **----------------MVNLIIAIWLGASLNIGSYKKVHLLTPYLGIKATLFLAATVVIVVATYYAALQILNWKWTAKIF**

14 AB363 100.0% 99.3% **MLNFFSTLRNKQISLFMFNLIIAIWLGAILNIGFYHQVHTLTPYFGVKAILFLAATLVILVATYYAVLQILNWKWTAKIF**

15 KUSSH08 100.0% 99.3% **MLNFFSTLRNKQISLFMFNLIIAIWLGAILNIGFYHQVHTLTPYFGVKAILFLAATLVILVATYYAVLQILNWKWTAKIF**

16 KUSSH14 100.0% 99.3% **MLNFFSTLRNKQISLFMFNLIIAIWLGAILNIGFYHQVHTLTPYFGVKAILFLAATLVILVATYYAVLQILNWKWTAKIF**

17 TUMA 100.0% 99.3% **MLNFFSTLRNKQISLFMFNLIIAIWLGAILNIGFYHQVHTLTPYFGVKAILFLAATLVILVATYYAVLQILNWKWTAKIF**

18 4300STDY7045886 97.1% 99.2% **----------------MFNLIIAIWLGAILNIGFYHQVHTLTPYFGVKAILFLAATLVILVATYYAVLQILNWKWTAKIF**

19 Aci00866 100.0% 99.3% **MLNFFSTLRNKQISLFMFNLIIAIWLGAILNIGFYHQVHTLTPYFGVKAILFLAATLVILVATYYAVLQILNWKWTAKIF**

20 Aci00860 100.0% 99.3% **MLNFFSTLRNKQISLFMFNLIIAIWLGAILNIGFYHQVHTLTPYFGVKAILFLAATLVILVATYYAVLQILNWKWTAKIF**

21 Aci00848 100.0% 99.3% **MLNFFSTLRNKQISLFMFNLIIAIWLGAILNIGFYHQVHTLTPYFGVKAILFLAATLVILVATYYAVLQILNWKWTAKIF**

22 MRSN351524 100.0% 99.3% **MLNFFSTLRNKQISLFMFNLIIAIWLGAILNIGFYHQVHTLTPYFGVKAILFLAATLVILVATYYAVLQILNWKWTAKIF**

cov pid  **81**  **. 1 . . . . : .** **160**

1 ATCC19606 100.0% 100.0% **AILLIFIGGFSSYFVNTLGVIISPDQIQNMVQTDVSEVTDLISLRFVLWTIFFVILPIFLITQVKFKQEKVSRLLLKKVF**

2 M03 84.9% 99.1% **---LIFIGGFSSYFVNTLGVIISPDQIQNMVQTDVSEVTDLISLRFVLWTVFFVILPIFLITQVKFKQEKVSRLLLKKVF**

3 M14 97.1% 98.9% **AILLIFIGGFSSYFVNTLGVIISPDQIQNMVQTDVSEVTDLISLRFVLWTVFFVILPIFLITQVKFKQEKVSRLLLKKVF**

4 AB_1649-8 97.1% 99.2% **AILLIFIGGFSSYFVNTLGVIISPDQIQNMVQTDVSEVTDLISLRFVLWTVFFVILPIFLITQVKFKQEKVSRLLLKKVF**

5 AB_1650-8 97.1% 99.2% **AILLIFIGGFSSYFVNTLGVIISPDQIQNMVQTDVSEVTDLISLRFVLWTVFFVILPIFLITQVKFKQEKVSRLLLKKVF**

6 UV_1036 97.1% 99.2% **AILLIFIGGFSSYFVNTLGVIISPDQIQNMVQTDVSEVTDLISLRFVLWTVFFVILPIFLITQVKFKQEKVSRLLLKKVF**

7 259_an 97.1% 99.2% **AILLIFIGGFSSYFVNTLGVIISPDQIQNMVQTDVSEVTDLISLRFVLWTVFFVILPIFLITQVKFKQEKVSRLLLKKVF**

8 276_ax 97.1% 99.2% **AILLIFIGGFSSYFVNTLGVIISPDQIQNMVQTDVSEVTDLISLRFVLWTVFFVILPIFLITQVKFKQEKVSRLLLKKVF**

9 PT061 100.0% 99.3% **AILLIFIGGFSSYFVNTLGVIISPDQIQNMVQTDVSEVTDLISLRFVLWTVFFVILPIFLITQVKFKQEKVSRLLLKKVF**

10 PT003 100.0% 99.3% **AILLIFIGGFSSYFVNTLGVIISPDQIQNMVQTDVSEVTDLISLRFVLWTVFFVILPIFLITQVKFKQEKVSRLLLKKVF**

11 PSU068 100.0% 99.3% **AILLIFIGGFSSYFVNTLGVIISPDQIQNMVQTDVSEVTDLISLRFVLWTVFFVILPIFLITQVKFKQEKVSRLLLKKVF**

12 PSU073 100.0% 99.3% **AILLIFIGGFSSYFVNTLGVIISPDQIQNMVQTDVSEVTDLISLRFVLWTVFFVILPIFLITQVKFKQEKVSRLLLKKVF**

13 CCBH26501 97.1% 90.6% **AILLVFIGGFSSYFVNTLGVIISPDQIQNMVQTDVSEVTDLISLRFVLWTIFFVILPIFLITQVKFKQEKVSRLLLKKVF**

14 AB363 100.0% 99.3% **AILLIFIGGFSSYFVNTLGVIISPDQIQNMVQTDVSEVTDLISLRFVLWTVFFVILPIFLITQVKFKQEKVSRLLLKKVF**

15 KUSSH08 100.0% 99.3% **AILLIFIGGFSSYFVNTLGVIISPDQIQNMVQTDVSEVTDLISLRFVLWTVFFVILPIFLITQVKFKQEKVSRLLLKKVF**

16 KUSSH14 100.0% 99.3% **AILLIFIGGFSSYFVNTLGVIISPDQIQNMVQTDVSEVTDLISLRFVLWTVFFVILPIFLITQVKFKQEKVSRLLLKKVF**

17 TUMA 100.0% 99.3% **AILLIFIGGFSSYFVNTLGVIISPDQIQNMVQTDVSEVTDLISLRFVLWTVFFVILPIFLITQVKFKQEKVSRLLLKKVF**

18 4300STDY7045886 97.1% 99.2% **AILLIFIGGFSSYFVNTLGVIISPDQIQNMVQTDVSEVTDLISLRFVLWTVFFVILPIFLITQVKFKQEKVSRLLLKKVF**

19 Aci00866 100.0% 99.3% **AILLIFIGGFSSYFVNTLGVIISPDQIQNMVQTDVSEVTDLISLRFVLWTVFFVILPIFLITQVKFKQEKVSRLLLKKVF**

20 Aci00860 100.0% 99.3% **AILLIFIGGFSSYFVNTLGVIISPDQIQNMVQTDVSEVTDLISLRFVLWTVFFVILPIFLITQVKFKQEKVSRLLLKKVF**

21 Aci00848 100.0% 99.3% **AILLIFIGGFSSYFVNTLGVIISPDQIQNMVQTDVSEVTDLISLRFVLWTVFFVILPIFLITQVKFKQEKVSRLLLKKVF**

22 MRSN351524 100.0% 99.3% **AILLIFIGGFSSYFVNTLGVIISPDQIQNMVQTDVSEVTDLISLRFVLWTVFFVILPIFLITQVKFKQEKVSRLLLKKVF**

cov pid **161**  **. . . 2 . . . .** **240**

1 ATCC19606 100.0% 100.0% **SLVASFAVVGVLLFTYYVDFAAIFREHRDLKGMISPQNSISSLMSYYHKKAPKKNLPLVIYGQDAHQVQRVQKNLPKLMI**

2 M03 84.9% 99.1% **SLVASFAVVGVLLFTYYVDFAAIFREHRDLKGMISPQNSISSLMSYYHKKAPKKNLPLVIYGQDAHQVQRVHKNLPKLMI**

3 M14 97.1% 98.9% **SLVASFAVVGVLLFTYYVDFAAIFREHRDLKGMISPQNSISSLMSYYHKKAPKKNLPLVIYGQDAHQVQRVHKNLPKLMI**

4 AB_1649-8 97.1% 99.2% **SLVASFAVVGVLLFTYYVDFAAIFREHRDLKGMISPQNSISSLMSYYHKKAPKKNLPLVIYGQDAHQVQRVHKNLPKLMI**

5 AB_1650-8 97.1% 99.2% **SLVASFAVVGVLLFTYYVDFAAIFREHRDLKGMISPQNSISSLMSYYHKKAPKKNLPLVIYGQDAHQVQRVHKNLPKLMI**

6 UV_1036 97.1% 99.2% **SLVASFAVVGVLLFTYYVDFAAIFREHRDLKGMISPQNSISSLMSYYHKKAPKKNLPLVIYGQDAHQVQRVHKNLPKLMI**

7 259_an 97.1% 99.2% **SLVASFAVVGVLLFTYYVDFAAIFREHRDLKGMISPQNSISSLMSYYHKKAPKKNLPLVIYGQDAHQVQRVHKNLPKLMI**

8 276_ax 97.1% 99.2% **SLVASFAVVGVLLFTYYVDFAAIFREHRDLKGMISPQNSISSLMSYYHKKAPKKNLPLVIYGQDAHQVQRVHKNLPKLMI**

9 PT061 100.0% 99.3% **SLVASFAVVGVLLFTYYVDFAAIFREHRDLKGMISPQNSISSLMSYYHKKAPKKNLPLVIYGQDAHQVQRVHKNLPKLMI**

10 PT003 100.0% 99.3% **SLVASFAVVGVLLFTYYVDFAAIFREHRDLKGMISPQNSISSLMSYYHKKAPKKNLPLVIYGQDAHQVQRVHKNLPKLMI**

11 PSU068 100.0% 99.3% **SLVASFAVVGVLLFTYYVDFAAIFREHRDLKGMISPQNSISSLMSYYHKKAPKKNLPLVIYGQDAHQVQRVHKNLPKLMI**

12 PSU073 100.0% 99.3% **SLVASFAVVGVLLFTYYVDFAAIFREHRDLKGMISPQNSISSLMSYYHKKAPKKNLPLVIYGQDAHQVQRVHKNLPKLMI**

13 CCBH26501 97.1% 90.6% **SLVASFAVVGVLLFTYYVDFAAIFREHRDLKGMISPQNTISSVMSYYRKKAPKKNLPLVKYGEDAHQVQQTQKDLPKLMV**

14 AB363 100.0% 99.3% **SLVASFAVVGVLLFTYYVDFAAIFREHRDLKGMISPQNSISSLMSYYHKKAPKKNLPLVIYGQDAHQVQRVHKNLPKLMI**

15 KUSSH08 100.0% 99.3% **SLVASFAVVGVLLFTYYVDFAAIFREHRDLKGMISPQNSISSLMSYYHKKAPKKNLPLVIYGQDAHQVQRVHKNLPKLMI**

16 KUSSH14 100.0% 99.3% **SLVASFAVVGVLLFTYYVDFAAIFREHRDLKGMISPQNSISSLMSYYHKKAPKKNLPLVIYGQDAHQVQRVHKNLPKLMI**

17 TUMA 100.0% 99.3% **SLVASFAVVGVLLFTYYVDFAAIFREHRDLKGMISPQNSISSLMSYYHKKAPKKNLPLVIYGQDAHQVQRVHKNLPKLMI**

18 4300STDY7045886 97.1% 99.2% **SLVASFAVVGVLLFTYYVDFAAIFREHRDLKGMISPQNSISSLMSYYHKKAPKKNLPLVIYGQDAHQVQRVHKNLPKLMI**

19 Aci00866 100.0% 99.3% **SLVASFAVVGVLLFTYYVDFAAIFREHRDLKGMISPQNSISSLMSYYHKKAPKKNLPLVIYGQDAHQVQRVHKNLPKLMI**

20 Aci00860 100.0% 99.3% **SLVASFAVVGVLLFTYYVDFAAIFREHRDLKGMISPQNSISSLMSYYHKKAPKKNLPLVIYGQDAHQVQRVHKNLPKLMI**

21 Aci00848 100.0% 99.3% **SLVASFAVVGVLLFTYYVDFAAIFREHRDLKGMISPQNSISSLMSYYHKKAPKKNLPLVIYGQDAHQVQRVHKNLPKLMI**

22 MRSN351524 100.0% 99.3% **SLVASFAVVGVLLFTYYVDFAAIFREHRDLKGMISPQNSISSLMSYYHKKAPKKNLPLVIYGQDAHQVQRVHKNLPKLMI**

cov pid **241**  **: . . . . 3 . .** **320**

1 ATCC19606 100.0% 100.0% **LVVGETARAESFSLNGYAKNTNPELSKQDIFNFSQVSSCGTATAVSVPCMFSGMPRVDYNEQLASHREGLLDIAKRAGYQ**

2 M03 84.9% 99.1% **LVVGETARAESFSLNGYAKNTNPELSKQDIFNFSQVSSCGTATAVSVPCMFSGMPRVDYDEQLASHREGLLDIAKRAGYQ**

3 M14 97.1% 98.9% **LVVGETARAESFSLNGYAKNTNPELSKQDIFNFSQVSSCGTATAVSVPCMFSGMPRVDYDEQLASHREGLLDIAKRAGYQ**

4 AB_1649-8 97.1% 99.2% **LVVGETARAESFSLNGYAKNTNPELSKQDIFNFSQVSSCGTATAVSVPCMFSGMPRVDYDEQLASHREGLLDIAKRAGYQ**

5 AB_1650-8 97.1% 99.2% **LVVGETARAESFSLNGYAKNTNPELSKQDIFNFSQVSSCGTATAVSVPCMFSGMPRVDYDEQLASHREGLLDIAKRAGYQ**

6 UV_1036 97.1% 99.2% **LVVGETARAESFSLNGYAKNTNPELSKQDIFNFSQVSSCGTATAVSVPCMFSGMPRVDYDEQLASHREGLLDIAKRAGYQ**

7 259_an 97.1% 99.2% **LVVGETARAESFSLNGYAKNTNPELSKQDIFNFSQVSSCGTATAVSVPCMFSGMPRVDYDEQLASHREGLLDIAKRAGYQ**

8 276_ax 97.1% 99.2% **LVVGETARAESFSLNGYAKNTNPELSKQDIFNFSQVSSCGTATAVSVPCMFSGMPRVDYDEQLASHREGLLDIAKRAGYQ**

9 PT061 100.0% 99.3% **LVVGETARAESFSLNGYAKNTNPELSKQDIFNFSQVSSCGTATAVSVPCMFSGMPRVDYDEQLASHREGLLDIAKRAGYQ**

10 PT003 100.0% 99.3% **LVVGETARAESFSLNGYAKNTNPELSKQDIFNFSQVSSCGTATAVSVPCMFSGMPRVDYDEQLASHREGLLDIAKRAGYQ**

11 PSU068 100.0% 99.3% **LVVGETARAESFSLNGYAKNTNPELSKQDIFNFSQVSSCGTATAVSVPCMFSGMPRVDYDEQLASHREGLLDIAKRAGYQ**

12 PSU073 100.0% 99.3% **LVVGETARAESFSLNGYAKNTNPELSKQDIFNFSQVSSCGTATAVSVPCMFSGMPRVDYDEQLASHREGLLDIAKRAGYQ**

13 CCBH26501 97.1% 90.6% **LVVGETARAESFSLNGYAKNTNPELSKQNILNFSQVSSCGTATAVSVPCMFSGMPRADYDEQLASHREGLLDIAKRAGYQ**

14 AB363 100.0% 99.3% **LVVGETARAESFSLNGYAKNTNPELSKQDIFNFSQVSSCGTATAVSVPCMFSGMPRVDYDEQLASHREGLLDIAKRAGYQ**

15 KUSSH08 100.0% 99.3% **LVVGETARAESFSLNGYAKNTNPELSKQDIFNFSQVSSCGTATAVSVPCMFSGMPRVDYDEQLASHREGLLDIAKRAGYQ**

16 KUSSH14 100.0% 99.3% **LVVGETARAESFSLNGYAKNTNPELSKQDIFNFSQVSSCGTATAVSVPCMFSGMPRVDYDEQLASHREGLLDIAKRAGYQ**

17 TUMA 100.0% 99.3% **LVVGETARAESFSLNGYAKNTNPELSKQDIFNFSQVSSCGTATAVSVPCMFSGMPRVDYDEQLASHREGLLDIAKRAGYQ**

18 4300STDY7045886 97.1% 99.2% **LVVGETARAESFSLNGYAKNTNPELSKQDIFNFSQVSSCGTATAVSVPCMFSGMPRVDYDEQLASHREGLLDIAKRAGYQ**

19 Aci00866 100.0% 99.3% **LVVGETARAESFSLNGYAKNTNPELSKQDIFNFSQVSSCGTATAVSVPCMFSGMPRVDYDEQLASHREGLLDIAKRAGYQ**

20 Aci00860 100.0% 99.3% **LVVGETARAESFSLNGYAKNTNPELSKQDIFNFSQVSSCGTATAVSVPCMFSGMPRVDYDEQLASHREGLLDIAKRAGYQ**

21 Aci00848 100.0% 99.3% **LVVGETARAESFSLNGYAKNTNPELSKQDIFNFSQVSSCGTATAVSVPCMFSGMPRVDYDEQLASHREGLLDIAKRAGYQ**

22 MRSN351524 100.0% 99.3% **LVVGETARAESFSLNGYAKNTNPELSKQDIFNFSQVSSCGTATAVSVPCMFSGMPRVDYDEQLASHREGLLDIAKRAGYQ**

cov pid **321**  **. . : . . . . 4** **400**

1 ATCC19606 100.0% 100.0% **VTWIDNNSGCKGACDRVEQYQIPENLKKKWCKDGECYDDILIDSLKQYLATIAKDDDRPRLIVLHQVGSHGPAYYKRAPE**

2 M03 84.9% 99.1% **VTWIDNNSGCKGACDRVEQYQIPENLKKKWCKDGECYDDILIDSLKQYLATIAKDDDRPRLIVLHQVGSHGPAYYKRAPE**

3 M14 97.1% 98.9% **VTWIDNNSGCKGACDRVEQYQIPENLKKKWCKDGECYDDILIDSLKQYLATIAKDDDRPRLIVLHQVGSHGPAYYKRAPE**

4 AB_1649-8 97.1% 99.2% **VTWIDNNSGCKGACDRVEQYQIPENLKKKWCKDGECYDDILIDSLKQYLATIAKDDDRPRLIVLHQVGSHGPAYYKRAPE**

5 AB_1650-8 97.1% 99.2% **VTWIDNNSGCKGACDRVEQYQIPENLKKKWCKDGECYDDILIDSLKQYLATIAKDDDRPRLIVLHQVGSHGPAYYKRAPE**

6 UV_1036 97.1% 99.2% **VTWIDNNSGCKGACDRVEQYQIPENLKKKWCKDGECYDDILIDSLKQYLATIAKDDDRPRLIVLHQVGSHGPAYYKRAPE**

7 259_an 97.1% 99.2% **VTWIDNNSGCKGACDRVEQYQIPENLKKKWCKDGECYDDILIDSLKQYLATIAKDDDRPRLIVLHQVGSHGPAYYKRAPE**

8 276_ax 97.1% 99.2% **VTWIDNNSGCKGACDRVEQYQIPENLKKKWCKDGECYDDILIDSLKQYLATIAKDDDRPRLIVLHQVGSHGPAYYKRAPE**

9 PT061 100.0% 99.3% **VTWIDNNSGCKGACDRVEQYQIPENLKKKWCKDGECYDDILIDSLKQYLATIAKDDDRPRLIVLHQVGSHGPAYYKRAPE**

10 PT003 100.0% 99.3% **VTWIDNNSGCKGACDRVEQYQIPENLKKKWCKDGECYDDILIDSLKQYLATIAKDDDRPRLIVLHQVGSHGPAYYKRAPE**

11 PSU068 100.0% 99.3% **VTWIDNNSGCKGACDRVEQYQIPENLKKKWCKDGECYDDILIDSLKQYLATIAKDDDRPRLIVLHQVGSHGPAYYKRAPE**

12 PSU073 100.0% 99.3% **VTWIDNNSGCKGACDRVEQYQIPENLKKKWCKDGECYDDILIDSLKQYLATIAKDDDRPRLIVLHQVGSHGPAYYKRAPE**

13 CCBH26501 97.1% 90.6% **VTWIDNNSGCKGACDRVEQYQIPEDLKQKWCKDGECLDDILIDSLKQYLASIPKDDKRPRLVVLHQMGSHGPAYYKRAPE**

14 AB363 100.0% 99.3% **VTWIDNNSGCKGACDRVEQYQIPENLKKKWCKDGECYDDILIDSLKQYLATIAKDDDRPRLIVLHQVGSHGPAYYKRAPE**

15 KUSSH08 100.0% 99.3% **VTWIDNNSGCKGACDRVEQYQIPENLKKKWCKDGECYDDILIDSLKQYLATIAKDDDRPRLIVLHQVGSHGPAYYKRAPE**

16 KUSSH14 100.0% 99.3% **VTWIDNNSGCKGACDRVEQYQIPENLKKKWCKDGECYDDILIDSLKQYLATIAKDDDRPRLIVLHQVGSHGPAYYKRAPE**

17 TUMA 100.0% 99.3% **VTWIDNNSGCKGACDRVEQYQIPENLKKKWCKDGECYDDILIDSLKQYLATIAKDDDRPRLIVLHQVGSHGPAYYKRAPE**

18 4300STDY7045886 97.1% 99.2% **VTWIDNNSGCKGACDRVEQYQIPENLKKKWCKDGECYDDILIDSLKQYLATIAKDDDRPRLIVLHQVGSHGPAYYKRAPE**

19 Aci00866 100.0% 99.3% **VTWIDNNSGCKGACDRVEQYQIPENLKKKWCKDGECYDDILIDSLKQYLATIAKDDDRPRLIVLHQVGSHGPAYYKRAPE**

20 Aci00860 100.0% 99.3% **VTWIDNNSGCKGACDRVEQYQIPENLKKKWCKDGECYDDILIDSLKQYLATIAKDDDRPRLIVLHQVGSHGPAYYKRAPE**

21 Aci00848 100.0% 99.3% **VTWIDNNSGCKGACDRVEQYQIPENLKKKWCKDGECYDDILIDSLKQYLATIAKDDDRPRLIVLHQVGSHGPAYYKRAPE**

22 MRSN351524 100.0% 99.3% **VTWIDNNSGCKGACDRVEQYQIPENLKKKWCKDGECYDDILIDSLKQYLATIAKDDDRPRLIVLHQVGSHGPAYYKRAPE**

cov pid **401**  **. . . . : . . .** **480**

1 ATCC19606 100.0% 100.0% **AYQPFKPTCDTNAIQGCSQTELLNSYDNTIVYTDHVLSQMINTLKEISKYQTGLWYLSDHGESTGEHGLYLHGSPYAIAP**

2 M03 84.9% 99.1% **AYQPFKPTCDTNAIQGCSQTELLNSYDNTIVYTDHVLSQMINTLKEISKYQTGLWYLSDHGESTGEHGLYLHGSPYAIAP**

3 M14 97.1% 98.9% **AYQPFKPTCDTNAIQGCSQTELLNSYDNTIVYTDHVLSQMINTLKEISKYQTGLWYLSDHGESTGEHGLYLHGSPYAIAP**

4 AB_1649-8 97.1% 99.2% **AYQPFKPTCDTNAIQGCSQTELLNSYDNTIVYTDHVLSQMINTLKEISKYQTGLWYLSDHGESTGEHGLYLHGSPYAIAP**

5 AB_1650-8 97.1% 99.2% **AYQPFKPTCDTNAIQGCSQTELLNSYDNTIVYTDHVLSQMINTLKEISKYQTGLWYLSDHGESTGEHGLYLHGSPYAIAP**

6 UV_1036 97.1% 99.2% **AYQPFKPTCDTNAIQGCSQTELLNSYDNTIVYTDHVLSQMINTLKEISKYQTGLWYLSDHGESTGEHGLYLHGSPYAIAP**

7 259_an 97.1% 99.2% **AYQPFKPTCDTNAIQGCSQTELLNSYDNTIVYTDHVLSQMINTLKEISKYQTGLWYLSDHGESTGEHGLYLHGSPYAIAP**

8 276_ax 97.1% 99.2% **AYQPFKPTCDTNAIQGCSQTELLNSYDNTIVYTDHVLSQMINTLKEISKYQTGLWYLSDHGESTGEHGLYLHGSPYAIAP**

9 PT061 100.0% 99.3% **AYQPFKPTCDTNAIQGCSQTELLNSYDNTIVYTDHVLSQMINTLKEISKYQTGLWYLSDHGESTGEHGLYLHGSPYAIAP**

10 PT003 100.0% 99.3% **AYQPFKPTCDTNAIQGCSQTELLNSYDNTIVYTDHVLSQMINTLKEISKYQTGLWYLSDHGESTGEHGLYLHGSPYAIAP**

11 PSU068 100.0% 99.3% **AYQPFKPTCDTNAIQGCSQTELLNSYDNTIVYTDHVLSQMINTLKEISKYQTGLWYLSDHGESTGEHGLYLHGSPYAIAP**

12 PSU073 100.0% 99.3% **AYQPFKPTCDTNAIQGCSQTELLNSYDNTIVYTDHVLSQMINTLKEISKYQTGLWYLSDHGESTGEHGLYLHGSPYAIAP**

13 CCBH26501 97.1% 90.6% **GYQPFKPTCDTNAIQGCSPAELINSYDNTIVYTDHVLSQMINTLKEVSNYQTGFWYLSDHGESTGEHGMYLHGSPYSIAP**

14 AB363 100.0% 99.3% **AYQPFKPTCDTNAIQGCSQTELLNSYDNTIVYTDHVLSQMINTLKEISKYQTGLWYLSDHGESTGEHGLYLHGSPYAIAP**

15 KUSSH08 100.0% 99.3% **AYQPFKPTCDTNAIQGCSQTELLNSYDNTIVYTDHVLSQMINTLKEISKYQTGLWYLSDHGESTGEHGLYLHGSPYAIAP**

16 KUSSH14 100.0% 99.3% **AYQPFKPTCDTNAIQGCSQTELLNSYDNTIVYTDHVLSQMINTLKEISKYQTGLWYLSDHGESTGEHGLYLHGSPYAIAP**

17 TUMA 100.0% 99.3% **AYQPFKPTCDTNAIQGCSQTELLNSYDNTIVYTDHVLSQMINTLKEISKYQTGLWYLSDHGESTGEHGLYLHGSPYAIAP**

18 4300STDY7045886 97.1% 99.2% **AYQPFKPTCDTNAIQGCSQTELLNSYDNTIVYTDHVLSQMINTLKEISKYQTGLWYLSDHGESTGEHGLYLHGSPYAIAP**

19 Aci00866 100.0% 99.3% **AYQPFKPTCDTNAIQGCSQTELLNSYDNTIVYTDHVLSQMINTLKEISKYQTGLWYLSDHGESTGEHGLYLHGSPYAIAP**

20 Aci00860 100.0% 99.3% **AYQPFKPTCDTNAIQGCSQTELLNSYDNTIVYTDHVLSQMINTLKEISKYQTGLWYLSDHGESTGEHGLYLHGSPYAIAP**

21 Aci00848 100.0% 99.3% **AYQPFKPTCDTNAIQGCSQTELLNSYDNTIVYTDHVLSQMINTLKEISKYQTGLWYLSDHGESTGEHGLYLHGSPYAIAP**

22 MRSN351524 100.0% 99.3% **AYQPFKPTCDTNAIQGCSQTELLNSYDNTIVYTDHVLSQMINTLKEISKYQTGLWYLSDHGESTGEHGLYLHGSPYAIAP**

cov pid **481**  **. 5 . . . . ]** **549**

1 ATCC19606 100.0% 100.0% **SQQTHVPMIMWFSESWKQHNLAQVNCLSQQTKQKLSQDNLFPSLLSLLDVKTQVINPQLDMLHSCAHVN**

2 M03 84.9% 99.1% **SQQTHVPMIMWFSESWKQHNLAQVNCLSQQTKQNLSQDNLFPSLLSLLDVKTQVINPQLDMLHSCAHVN**

3 M14 97.1% 98.9% **SQQTHVPMIMWFSESWKQHNLAQVNCLSQQTKQNLSQDNLFPSLLSLLDVKTQVINPQLDMLHSCAHVN**

4 AB_1649-8 97.1% 99.2% **SQQTHVPMIMWFSESWKQHNLAQVNCLSQQTKQNLSQDNLFPSLLSLLDVKTQVINPQLDMLHSCAHVN**

5 AB_1650-8 97.1% 99.2% **SQQTHVPMIMWFSESWKQHNLAQVNCLSQQTKQNLSQDNLFPSLLSLLDVKTQVINPQLDMLHSCAHVN**

6 UV_1036 97.1% 99.2% **SQQTHVPMIMWFSESWKQHNLAQVNCLSQQTKQNLSQDNLFPSLLSLLDVKTQVINPQLDMLHSCAHVN**

7 259_an 97.1% 99.2% **SQQTHVPMIMWFSESWKQHNLAQVNCLSQQTKQNLSQDNLFPSLLSLLDVKTQVINPQLDMLHSCAHVN**

8 276_ax 97.1% 99.2% **SQQTHVPMIMWFSESWKQHNLAQVNCLSQQTKQNLSQDNLFPSLLSLLDVKTQVINPQLDMLHSCAHVN**

9 PT061 100.0% 99.3% **SQQTHVPMIMWFSESWKQHNLAQVNCLSQQTKQNLSQDNLFPSLLSLLDVKTQVINPQLDMLHSCAHVN**

10 PT003 100.0% 99.3% **SQQTHVPMIMWFSESWKQHNLAQVNCLSQQTKQNLSQDNLFPSLLSLLDVKTQVINPQLDMLHSCAHVN**

11 PSU068 100.0% 99.3% **SQQTHVPMIMWFSESWKQHNLAQVNCLSQQTKQNLSQDNLFPSLLSLLDVKTQVINPQLDMLHSCAHVN**

12 PSU073 100.0% 99.3% **SQQTHVPMIMWFSESWKQHNLAQVNCLSQQTKQNLSQDNLFPSLLSLLDVKTQVINPQLDMLHSCAHVN**

13 CCBH26501 97.1% 90.6% **SQQTHIPMIMWFSDGWKQNNLAQVNCLNQQTKQKLSQDNLFPSLLSMLDVKTQVINPQLDMLHSCANVN**

14 AB363 100.0% 99.3% **SQQTHVPMIMWFSESWKQHNLAQVNCLSQQTKQNLSQDNLFPSLLSLLDVKTQVINPQLDMLHSCAHVN**

15 KUSSH08 100.0% 99.3% **SQQTHVPMIMWFSESWKQHNLAQVNCLSQQTKQNLSQDNLFPSLLSLLDVKTQVINPQLDMLHSCAHVN**

16 KUSSH14 100.0% 99.3% **SQQTHVPMIMWFSESWKQHNLAQVNCLSQQTKQNLSQDNLFPSLLSLLDVKTQVINPQLDMLHSCAHVN**

17 TUMA 100.0% 99.3% **SQQTHVPMIMWFSESWKQHNLAQVNCLSQQTKQNLSQDNLFPSLLSLLDVKTQVINPQLDMLHSCAHVN**

18 4300STDY7045886 97.1% 99.2% **SQQTHVPMIMWFSESWKQHNLAQVNCLSQQTKQNLSQDNLFPSLLSLLDVKTQVINPQLDMLHSCAHVN**

19 Aci00866 100.0% 99.3% **SQQTHVPMIMWFSESWKQHNLAQVNCLSQQTKQNLSQDNLFPSLLSLLDVKTQVINPQLDMLHSCAHVN**

20 Aci00860 100.0% 99.3% **SQQTHVPMIMWFSESWKQHNLAQVNCLSQQTKQNLSQDNLFPSLLSLLDVKTQVINPQLDMLHSCAHVN**

21 Aci00848 100.0% 99.3% **SQQTHVPMIMWFSESWKQHNLAQVNCLSQQTKQNLSQDNLFPSLLSLLDVKTQVINPQLDMLHSCAHVN**

22 MRSN351524 100.0% 99.3% **SQQTHVPMIMWFSESWKQHNLAQVNCLSQQTKQNLSQDNLFPSLLSLLDVKTQVINPQLDMLHSCAHVN**

**Supplementary Figure 3: Multiple sequence alignment (MSA) of the predicted amino acid sequence of PmrC carried by ST113^Pas^ isolates and close genomes retrieved from the BV-BRC database compared to the respective gene in *A. baumannii* ATCC 19606.** MSA was created by the A multiple alignment viewer MView hosted by the EMBL-EBI; cov, coverage; pid, percent identity.

cov pid  **1** **[ . . . . : . . .** **80**

1 ATCC_19606 100.0% 100.0% **MLNFFSTLRNKQISLFMFNLIIAIWLGAILNIGFYHQVHTLTPYFGVKAILFLAATLVILVATYYAVLQILNWKWTAKIF**

2 M02 100.0% 99.5% **MLNFFSTLRNKQISLFMFNLIIAIWLGAILNIGFYHQVHTLTPYFGVKAILFLAATLVILVATYYAVLQILNWKWTAKIF**

3 M11 79.6% 98.4% **MLNFFSTLRNKQISLFMFNLIIAIWLGAILNIGFYHQVHTLTPYFGVKAILFLAATLVILVATYYAVLQILNWKWTAKIF**

4 M18 100.0% 99.5% **MLNFFSTLRNKQISLFMFNLIIAIWLGAILNIGFYHQVHTLTPYFGVKAILFLAATLVILVATYYAVLQILNWKWTAKIF**

5 MRSN15574 100.0% 99.5% **MLNFFSTLRNKQISLFMFNLIIAIWLGAILNIGFYHQVHTLTPYFGVKAILFLAATLVILVATYYAVLQILNWKWTAKIF**

6 AB-C 100.0% 99.5% **MLNFFSTLRNKQISLFMFNLIIAIWLGAILNIGFYHQVHTLTPYFGVKAILFLAATLVILVATYYAVLQILNWKWTAKIF**

7 ACMH-6201 97.1% 99.4% **----------------MFNLIIAIWLGAILNIGFYHQVHTLTPYFGVKAILFLAATLVILVATYYAVLQILNWKWTAKIF**

8 Survcare112 97.1% 99.4% **----------------MFNLIIAIWLGAILNIGFYHQVHTLTPYFGVKAILFLAATLVILVATYYAVLQILNWKWTAKIF**

9 15953 100.0% 99.5% **MLNFFSTLRNKQISLFMFNLIIAIWLGAILNIGFYHQVHTLTPYFGVKAILFLAATLVILVATYYAVLQILNWKWTAKIF**

10 MBL_M10 100.0% 99.5% **MLNFFSTLRNKQISLFMFNLIIAIWLGAILNIGFYHQVHTLTPYFGVKAILFLAATLVILVATYYAVLQILNWKWTAKIF**

11 AR_0033 97.1% 99.4% **----------------MFNLIIAIWLGAILNIGFYHQVHTLTPYFGVKAILFLAATLVILVATYYAVLQILNWKWTAKIF**

12 17A1955 100.0% 99.5% **MLNFFSTLRNKQISLFMFNLIIAIWLGAILNIGFYHQVHTLTPYFGVKAILFLAATLVILVATYYAVLQILNWKWTAKIF**

13 MBL_M1 100.0% 99.5% **MLNFFSTLRNKQISLFMFNLIIAIWLGAILNIGFYHQVHTLTPYFGVKAILFLAATLVILVATYYAVLQILNWKWTAKIF**

14 15946 100.0% 99.5% **MLNFFSTLRNKQISLFMFNLIIAIWLGAILNIGFYHQVHTLTPYFGVKAILFLAATLVILVATYYAVLQILNWKWTAKIF**

15 AB-B 100.0% 99.5% **MLNFFSTLRNKQISLFMFNLIIAIWLGAILNIGFYHQVHTLTPYFGVKAILFLAATLVILVATYYAVLQILNWKWTAKIF**

16 26 100.0% 99.5% **MLNFFSTLRNKQISLFMFNLIIAIWLGAILNIGFYHQVHTLTPYFGVKAILFLAATLVILVATYYAVLQILNWKWTAKIF**

17 Ab-NDM-1 97.1% 99.4% **----------------MFNLIIAIWLGAILNIGFYHQVHTLTPYFGVKAILFLAATLVILVATYYAVLQILNWKWTAKIF**

18 P116A 100.0% 99.5% **MLNFFSTLRNKQISLFMFNLIIAIWLGAILNIGFYHQVHTLTPYFGVKAILFLAATLVILVATYYAVLQILNWKWTAKIF**

19 MBL_M6 100.0% 99.5% **MLNFFSTLRNKQISLFMFNLIIAIWLGAILNIGFYHQVHTLTPYFGVKAILFLAATLVILVATYYAVLQILNWKWTAKIF**

20 AE3M 97.1% 99.4% **----------------MFNLIIAIWLGAILNIGFYHQVHTLTPYFGVKAILFLAATLVILVATYYAVLQILNWKWTAKIF**

21 AB177-VUB 100.0% 99.5% **MLNFFSTLRNKQISLFMFNLIIAIWLGAILNIGFYHQVHTLTPYFGVKAILFLAATLVILVATYYAVLQILNWKWTAKIF**

22 Cl300 100.0% 99.5% **MLNFFSTLRNKQISLFMFNLIIAIWLGAILNIGFYHQVHTLTPYFGVKAILFLAATLVILVATYYAVLQILNWKWTAKIF**

23 AR_0037 97.1% 99.4% **----------------MFNLIIAIWLGAILNIGFYHQVHTLTPYFGVKAILFLAATLVILVATYYAVLQILNWKWTAKIF**

24 AB-A 100.0% 99.5% **MLNFFSTLRNKQISLFMFNLIIAIWLGAILNIGFYHQVHTLTPYFGVKAILFLAATLVILVATYYAVLQILNWKWTAKIF**

25 MBL_M9 47.0% 99.2% **--------------------------------------------------------------------------------**

26 R11 100.0% 99.5% **MLNFFSTLRNKQISLFMFNLIIAIWLGAILNIGFYHQVHTLTPYFGVKAILFLAATLVILVATYYAVLQILNWKWTAKIF**

27 MIN-015 100.0% 99.5% **MLNFFSTLRNKQISLFMFNLIIAIWLGAILNIGFYHQVHTLTPYFGVKAILFLAATLVILVATYYAVLQILNWKWTAKIF**

cov pid  **81**  **. 1 . . . . : .** **160**

1 ATCC_19606 100.0% 100.0% **AILLIFIGGFSSYFVNTLGVIISPDQIQNMVQTDVSEVTDLISLRFVLWTIFFVILPIFLITQVKFKQEKVSRLLLKKVF**

2 M02 100.0% 99.5% **AILLIFIGGFSSYFVNTLGVIISPDQIQNMVQTDVSEVTDLISLRFVLWTVFFVILPIFLITQVKFKQEKVSRLLLKKVF**

3 M11 79.6% 98.4% **AILLIFIGGFSSYFVNTLGVIISPDQIQNMVQTDVSEVTDLISLRFVLWTVFFVILPIFLITQVKFKQEKVSRLLLKKVF**

4 M18 100.0% 99.5% **AILLIFIGGFSSYFVNTLGVIISPDQIQNMVQTDVSEVTDLISLRFVLWTVFFVILPIFLITQVKFKQEKVSRLLLKKVF**

5 MRSN15574 100.0% 99.5% **AILLIFIGGFSSYFVNTLGVIISPDQIQNMVQTDVSEVTDLISLRFVLWTVFFVILPIFLITQVKFKQEKVSRLLLKKVF**

6 AB-C 100.0% 99.5% **AILLIFIGGFSSYFVNTLGVIISPDQIQNMVQTDVSEVTDLISLRFVLWTVFFVILPIFLITQVKFKQEKVSRLLLKKVF**

7 ACMH-6201 97.1% 99.4% **AILLIFIGGFSSYFVNTLGVIISPDQIQNMVQTDVSEVTDLISLRFVLWTVFFVILPIFLITQVKFKQEKVSRLLLKKVF**

8 Survcare112 97.1% 99.4% **AILLIFIGGFSSYFVNTLGVIISPDQIQNMVQTDVSEVTDLISLRFVLWTVFFVILPIFLITQVKFKQEKVSRLLLKKVF**

9 15953 100.0% 99.5% **AILLIFIGGFSSYFVNTLGVIISPDQIQNMVQTDVSEVTDLISLRFVLWTVFFVILPIFLITQVKFKQEKVSRLLLKKVF**

10 MBL_M10 100.0% 99.5% **AILLIFIGGFSSYFVNTLGVIISPDQIQNMVQTDVSEVTDLISLRFVLWTVFFVILPIFLITQVKFKQEKVSRLLLKKVF**

11 AR_0033 97.1% 99.4% **AILLIFIGGFSSYFVNTLGVIISPDQIQNMVQTDVSEVTDLISLRFVLWTVFFVILPIFLITQVKFKQEKVSRLLLKKVF**

12 17A1955 100.0% 99.5% **AILLIFIGGFSSYFVNTLGVIISPDQIQNMVQTDVSEVTDLISLRFVLWTVFFVILPIFLITQVKFKQEKVSRLLLKKVF**

13 MBL_M1 100.0% 99.5% **AILLIFIGGFSSYFVNTLGVIISPDQIQNMVQTDVSEVTDLISLRFVLWTVFFVILPIFLITQVKFKQEKVSRLLLKKVF**

14 15946 100.0% 99.5% **AILLIFIGGFSSYFVNTLGVIISPDQIQNMVQTDVSEVTDLISLRFVLWTVFFVILPIFLITQVKFKQEKVSRLLLKKVF**

15 AB-B 100.0% 99.5% **AILLIFIGGFSSYFVNTLGVIISPDQIQNMVQTDVSEVTDLISLRFVLWTVFFVILPIFLITQVKFKQEKVSRLLLKKVF**

16 26 100.0% 99.5% **AILLIFIGGFSSYFVNTLGVIISPDQIQNMVQTDVSEVTDLISLRFVLWTVFFVILPIFLITQVKFKQEKVSRLLLKKVF**

17 Ab-NDM-1 97.1% 99.4% **AILLIFIGGFSSYFVNTLGVIISPDQIQNMVQTDVSEVTDLISLRFVLWTVFFVILPIFLITQVKFKQEKVSRLLLKKVF**

18 P116A 100.0% 99.5% **AILLIFIGGFSSYFVNTLGVIISPDQIQNMVQTDVSEVTDLISLRFVLWTVFFVILPIFLITQVKFKQEKVSRLLLKKVF**

19 MBL_M6 100.0% 99.5% **AILLIFIGGFSSYFVNTLGVIISPDQIQNMVQTDVSEVTDLISLRFVLWTVFFVILPIFLITQVKFKQEKVSRLLLKKVF**

20 AE3M 97.1% 99.4% **AILLIFIGGFSSYFVNTLGVIISPDQIQNMVQTDVSEVTDLISLRFVLWTVFFVILPIFLITQVKFKQEKVSRLLLKKVF**

21 AB177-VUB 100.0% 99.5% **AILLIFIGGFSSYFVNTLGVIISPDQIQNMVQTDVSEVTDLISLRFVLWTVFFVILPIFLITQVKFKQEKVSRLLLKKVF**

22 Cl300 100.0% 99.5% **AILLIFIGGFSSYFVNTLGVIISPDQIQNMVQTDVSEVTDLISLRFVLWTVFFVILPIFLITQVKFKQEKVSRLLLKKVF**

23 AR_0037 97.1% 99.4% **AILLIFIGGFSSYFVNTLGVIISPDQIQNMVQTDVSEVTDLISLRFVLWTVFFVILPIFLITQVKFKQEKVSRLLLKKVF**

24 AB-A 100.0% 99.5% **AILLIFIGGFSSYFVNTLGVIISPDQIQNMVQTDVSEVTDLISLRFVLWTVFFVILPIFLITQVKFKQEKVSRLLLKKVF**

25 MBL_M9 47.0% 99.2% **--------------------------------------------------------------------------------**

26 R11 100.0% 99.5% **AILLIFIGGFSSYFVNTLGVIISPDQIQNMVQTDVSEVTDLISLRFVLWTVFFVILPIFLITQVKFKQEKVSRLLLKKVF**

27 MIN-015 100.0% 99.5% **AILLIFIGGFSSYFVNTLGVIISPDQIQNMVQTDVSEVTDLISLRFVLWTVFFVILPIFLITQVKFKQEKVSRLLLKKVF**

cov pid **161**  **. . . 2 . . . .** **240**

1 ATCC_19606 100.0% 100.0% **SLVASFAVVGVLLFTYYVDFAAIFREHRDLKGMISPQNSISSLMSYYHKKAPKKNLPLVIYGQDAHQVQRVQKNLPKLMI**

2 M02 100.0% 99.5% **SLVASFAVVGVLLFTYYVDFAAIFREHRDLKGMISPQNSISSLMSYYHKKAPKKNLPLVIYGQDAHQVQRVQKNLPKLMI**

3 M11 79.6% 98.4% **SLVASFAVVGVLLFTYYVDFAAIFREHRDLKGMISPQNSISSLMSYYHKKAPKKNLPLVIYGQDAHQVQRVQKNLPKLMI**

4 M18 100.0% 99.5% **SLVASFAVVGVLLFTYYVDFAAIFREHRDLKGMISPQNSISSLMSYYHKKAPKKNLPLVIYGQDAHQVQRVQKNLPKLMI**

5 MRSN15574 100.0% 99.5% **SLVASFAVVGVLLFTYYVDFAAIFREHRDLKGMISPQNSISSLMSYYHKKAPKKNLPLVIYGQDAHQVQRVQKNLPKLMI**

6 AB-C 100.0% 99.5% **SLVASFAVVGVLLFTYYVDFAAIFREHRDLKGMISPQNSISSLMSYYHKKAPKKNLPLVIYGQDAHQVQRVQKNLPKLMI**

7 ACMH-6201 97.1% 99.4% **SLVASFAVVGVLLFTYYVDFAAIFREHRDLKGMISPQNSISSLMSYYHKKAPKKNLPLVIYGQDAHQVQRVQKNLPKLMI**

8 Survcare112 97.1% 99.4% **SLVASFAVVGVLLFTYYVDFAAIFREHRDLKGMISPQNSISSLMSYYHKKAPKKNLPLVIYGQDAHQVQRVQKNLPKLMI**

9 15953 100.0% 99.5% **SLVASFAVVGVLLFTYYVDFAAIFREHRDLKGMISPQNSISSLMSYYHKKAPKKNLPLVIYGQDAHQVQRVQKNLPKLMI**

10 MBL_M10 100.0% 99.5% **SLVASFAVVGVLLFTYYVDFAAIFREHRDLKGMISPQNSISSLMSYYHKKAPKKNLPLVIYGQDAHQVQRVQKNLPKLMI**

11 AR_0033 97.1% 99.4% **SLVASFAVVGVLLFTYYVDFAAIFREHRDLKGMISPQNSISSLMSYYHKKAPKKNLPLVIYGQDAHQVQRVQKNLPKLMI**

12 17A1955 100.0% 99.5% **SLVASFAVVGVLLFTYYVDFAAIFREHRDLKGMISPQNSISSLMSYYHKKAPKKNLPLVIYGQDAHQVQRVQKNLPKLMI**

13 MBL_M1 100.0% 99.5% **SLVASFAVVGVLLFTYYVDFAAIFREHRDLKGMISPQNSISSLMSYYHKKAPKKNLPLVIYGQDAHQVQRVQKNLPKLMI**

14 15946 100.0% 99.5% **SLVASFAVVGVLLFTYYVDFAAIFREHRDLKGMISPQNSISSLMSYYHKKAPKKNLPLVIYGQDAHQVQRVQKNLPKLMI**

15 AB-B 100.0% 99.5% **SLVASFAVVGVLLFTYYVDFAAIFREHRDLKGMISPQNSISSLMSYYHKKAPKKNLPLVIYGQDAHQVQRVQKNLPKLMI**

16 26 100.0% 99.5% **SLVASFAVVGVLLFTYYVDFAAIFREHRDLKGMISPQNSISSLMSYYHKKAPKKNLPLVIYGQDAHQVQRVQKNLPKLMI**

17 Ab-NDM-1 97.1% 99.4% **SLVASFAVVGVLLFTYYVDFAAIFREHRDLKGMISPQNSISSLMSYYHKKAPKKNLPLVIYGQDAHQVQRVQKNLPKLMI**

18 P116A 100.0% 99.5% **SLVASFAVVGVLLFTYYVDFAAIFREHRDLKGMISPQNSISSLMSYYHKKAPKKNLPLVIYGQDAHQVQRVQKNLPKLMI**

19 MBL_M6 100.0% 99.5% **SLVASFAVVGVLLFTYYVDFAAIFREHRDLKGMISPQNSISSLMSYYHKKAPKKNLPLVIYGQDAHQVQRVQKNLPKLMI**

20 AE3M 97.1% 99.4% **SLVASFAVVGVLLFTYYVDFAAIFREHRDLKGMISPQNSISSLMSYYHKKAPKKNLPLVIYGQDAHQVQRVQKNLPKLMI**

21 AB177-VUB 100.0% 99.5% **SLVASFAVVGVLLFTYYVDFAAIFREHRDLKGMISPQNSISSLMSYYHKKAPKKNLPLVIYGQDAHQVQRVQKNLPKLMI**

22 Cl300 100.0% 99.5% **SLVASFAVVGVLLFTYYVDFAAIFREHRDLKGMISPQNSISSLMSYYHKKAPKKNLPLVIYGQDAHQVQRVQKNLPKLMI**

23 AR_0037 97.1% 99.4% **SLVASFAVVGVLLFTYYVDFAAIFREHRDLKGMISPQNSISSLMSYYHKKAPKKNLPLVIYGQDAHQVQRVQKNLPKLMI**

24 AB-A 100.0% 99.5% **SLVASFAVVGVLLFTYYVDFAAIFREHRDLKGMISPQNSISSLMSYYHKKAPKKNLPLVIYGQDAHQVQRVQKNLPKLMI**

25 MBL_M9 47.0% 99.2% **--------------------------------------------------------------------------------**

26 R11 100.0% 99.5% **SLVASFAVVGVLLFTYYVDFAAIFREHRDLKGMISPQNSISSLMSYYHKKAPKKNLPLVIYGQDAHQVQRVQKNLPKLMI**

27 MIN-015 100.0% 99.5% **SLVASFAVVGVLLFTYYVDFAAIFREHRDLKGMISPQNSISSLMSYYHKKAPKKNLPLVIYGQDAHQVQRVQKNLPKLMI**

cov pid **241**  **: . . . . 3 . .** **320**

1 ATCC_19606 100.0% 100.0% **LVVGETARAESFSLNGYAKNTNPELSKQDIFNFSQVSSCGTATAVSVPCMFSGMPRVDYNEQLASHREGLLDIAKRAGYQ**

2 M02 100.0% 99.5% **LVVGETARAESFSLNGYAKNTNPELSKQDIFNFSQVSSCGTATAVSVPCMFSGMPRVDYDEQLASHREGLLDIAKRAGYQ**

3 M11 79.6% 98.4% **LVVGETARAESFSLNGYAKNTNPELSKQDIFNFSQVSSCGTATAVSVPCMFSGMPRVDYDEQLASHREGLLDIAKRAGYQ**

4 M18 100.0% 99.5% **LVVGETARAESFSLNGYAKNTNPELSKQDIFNFSQVSSCGTATAVSVPCMFSGMPRVDYDEQLASHREGLLDIAKRAGYQ**

5 MRSN15574 100.0% 99.5% **LVVGETARAESFSLNGYAKNTNPELSKQDIFNFSQVSSCGTATAVSVPCMFSGMPRVDYDEQLASHREGLLDIAKRAGYQ**

6 AB-C 100.0% 99.5% **LVVGETARAESFSLNGYAKNTNPELSKQDIFNFSQVSSCGTATAVSVPCMFSGMPRVDYDEQLASHREGLLDIAKRAGYQ**

7 ACMH-6201 97.1% 99.4% **LVVGETARAESFSLNGYAKNTNPELSKQDIFNFSQVSSCGTATAVSVPCMFSGMPRVDYDEQLASHREGLLDIAKRAGYQ**

8 Survcare112 97.1% 99.4% **LVVGETARAESFSLNGYAKNTNPELSKQDIFNFSQVSSCGTATAVSVPCMFSGMPRVDYDEQLASHREGLLDIAKRAGYQ**

9 15953 100.0% 99.5% **LVVGETARAESFSLNGYAKNTNPELSKQDIFNFSQVSSCGTATAVSVPCMFSGMPRVDYDEQLASHREGLLDIAKRAGYQ**

10 MBL_M10 100.0% 99.5% **LVVGETARAESFSLNGYAKNTNPELSKQDIFNFSQVSSCGTATAVSVPCMFSGMPRVDYDEQLASHREGLLDIAKRAGYQ**

11 AR_0033 97.1% 99.4% **LVVGETARAESFSLNGYAKNTNPELSKQDIFNFSQVSSCGTATAVSVPCMFSGMPRVDYDEQLASHREGLLDIAKRAGYQ**

12 17A1955 100.0% 99.5% **LVVGETARAESFSLNGYAKNTNPELSKQDIFNFSQVSSCGTATAVSVPCMFSGMPRVDYDEQLASHREGLLDIAKRAGYQ**

13 MBL_M1 100.0% 99.5% **LVVGETARAESFSLNGYAKNTNPELSKQDIFNFSQVSSCGTATAVSVPCMFSGMPRVDYDEQLASHREGLLDIAKRAGYQ**

14 15946 100.0% 99.5% **LVVGETARAESFSLNGYAKNTNPELSKQDIFNFSQVSSCGTATAVSVPCMFSGMPRVDYDEQLASHREGLLDIAKRAGYQ**

15 AB-B 100.0% 99.5% **LVVGETARAESFSLNGYAKNTNPELSKQDIFNFSQVSSCGTATAVSVPCMFSGMPRVDYDEQLASHREGLLDIAKRAGYQ**

16 26 100.0% 99.5% **LVVGETARAESFSLNGYAKNTNPELSKQDIFNFSQVSSCGTATAVSVPCMFSGMPRVDYDEQLASHREGLLDIAKRAGYQ**

17 Ab-NDM-1 97.1% 99.4% **LVVGETARAESFSLNGYAKNTNPELSKQDIFNFSQVSSCGTATAVSVPCMFSGMPRVDYDEQLASHREGLLDIAKRAGYQ**

18 P116A 100.0% 99.5% **LVVGETARAESFSLNGYAKNTNPELSKQDIFNFSQVSSCGTATAVSVPCMFSGMPRVDYDEQLASHREGLLDIAKRAGYQ**

19 MBL_M6 100.0% 99.5% **LVVGETARAESFSLNGYAKNTNPELSKQDIFNFSQVSSCGTATAVSVPCMFSGMPRVDYDEQLASHREGLLDIAKRAGYQ**

20 AE3M 97.1% 99.4% **LVVGETARAESFSLNGYAKNTNPELSKQDIFNFSQVSSCGTATAVSVPCMFSGMPRVDYDEQLASHREGLLDIAKRAGYQ**

21 AB177-VUB 100.0% 99.5% **LVVGETARAESFSLNGYAKNTNPELSKQDIFNFSQVSSCGTATAVSVPCMFSGMPRVDYDEQLASHREGLLDIAKRAGYQ**

22 Cl300 100.0% 99.5% **LVVGETARAESFSLNGYAKNTNPELSKQDIFNFSQVSSCGTATAVSVPCMFSGMPRVDYDEQLASHREGLLDIAKRAGYQ**

23 AR_0037 97.1% 99.4% **LVVGETARAESFSLNGYAKNTNPELSKQDIFNFSQVSSCGTATAVSVPCMFSGMPRVDYDEQLASHREGLLDIAKRAGYQ**

24 AB-A 100.0% 99.5% **LVVGETARAESFSLNGYAKNTNPELSKQDIFNFSQVSSCGTATAVSVPCMFSGMPRVDYDEQLASHREGLLDIAKRAGYQ**

25 MBL_M9 47.0% 99.2% **---------------------------------------------------SGMPRVDYDEQLASHREGLLDIAKRAGYQ**

26 R11 100.0% 99.5% **LVVGETARAESFSLNGYAKNTNPELSKQDIFNFSQVSSCGTATAVSVPCMFSGMPRVDYDEQLASHREGLLDIAKRAGYQ**

27 MIN-015 100.0% 99.5% **LVVGETARAESFSLNGYAKNTNPELSKQDIFNFSQVSSCGTATAVSVPCMFSGMPRVDYDEQLASHREGLLDIAKRAGYQ**

cov pid **321**  **. . : . . . . 4** **400**

1 ATCC_19606 100.0% 100.0% **VTWIDNNSGCKGACDRVEQYQIPENLKKKWCKDGECYDDILIDSLKQYLATIAKDDDRPRLIVLHQVGSHGPAYYKRAPE**

2 M02 100.0% 99.5% **VTWIDNNSGCKGACDRVEQYQTPENLKKKWCKDGECYDDILIDSLKQYLATIAKDDDRPRLIVLHQVGSHGPAYYKRAPE**

3 M11 79.6% 98.4% **VTWIDNNSGCKGACDRVEQYQTPENLKKKWCKDGECYDDILIDSLKQYLATIAKDDDRPRLIVLHQVGSHGPAYYKRAPE**

4 M18 100.0% 99.5% **VTWIDNNSGCKGACDRVEQYQTPENLKKKWCKDGECYDDILIDSLKQYLATIAKDDDRPRLIVLHQVGSHGPAYYKRAPE**

5 MRSN15574 100.0% 99.5% **VTWIDNNSGCKGACDRVEQYQTPENLKKKWCKDGECYDDILIDSLKQYLATIAKDDDRPRLIVLHQVGSHGPAYYKRAPE**

6 AB-C 100.0% 99.5% **VTWIDNNSGCKGACDRVEQYQTPENLKKKWCKDGECYDDILIDSLKQYLATIAKDDDRPRLIVLHQVGSHGPAYYKRAPE**

7 ACMH-6201 97.1% 99.4% **VTWIDNNSGCKGACDRVEQYQTPENLKKKWCKDGECYDDILIDSLKQYLATIAKDDDRPRLIVLHQVGSHGPAYYKRAPE**

8 Survcare112 97.1% 99.4% **VTWIDNNSGCKGACDRVEQYQTPENLKKKWCKDGECYDDILIDSLKQYLATIAKDDDRPRLIVLHQVGSHGPAYYKRAPE**

9 15953 100.0% 99.5% **VTWIDNNSGCKGACDRVEQYQTPENLKKKWCKDGECYDDILIDSLKQYLATIAKDDDRPRLIVLHQVGSHGPAYYKRAPE**

10 MBL_M10 100.0% 99.5% **VTWIDNNSGCKGACDRVEQYQTPENLKKKWCKDGECYDDILIDSLKQYLATIAKDDDRPRLIVLHQVGSHGPAYYKRAPE**

11 AR_0033 97.1% 99.4% **VTWIDNNSGCKGACDRVEQYQTPENLKKKWCKDGECYDDILIDSLKQYLATIAKDDDRPRLIVLHQVGSHGPAYYKRAPE**

12 17A1955 100.0% 99.5% **VTWIDNNSGCKGACDRVEQYQTPENLKKKWCKDGECYDDILIDSLKQYLATIAKDDDRPRLIVLHQVGSHGPAYYKRAPE**

13 MBL_M1 100.0% 99.5% **VTWIDNNSGCKGACDRVEQYQTPENLKKKWCKDGECYDDILIDSLKQYLATIAKDDDRPRLIVLHQVGSHGPAYYKRAPE**

14 15946 100.0% 99.5% **VTWIDNNSGCKGACDRVEQYQTPENLKKKWCKDGECYDDILIDSLKQYLATIAKDDDRPRLIVLHQVGSHGPAYYKRAPE**

15 AB-B 100.0% 99.5% **VTWIDNNSGCKGACDRVEQYQTPENLKKKWCKDGECYDDILIDSLKQYLATIAKDDDRPRLIVLHQVGSHGPAYYKRAPE**

16 26 100.0% 99.5% **VTWIDNNSGCKGACDRVEQYQTPENLKKKWCKDGECYDDILIDSLKQYLATIAKDDDRPRLIVLHQVGSHGPAYYKRAPE**

17 Ab-NDM-1 97.1% 99.4% **VTWIDNNSGCKGACDRVEQYQTPENLKKKWCKDGECYDDILIDSLKQYLATIAKDDDRPRLIVLHQVGSHGPAYYKRAPE**

18 P116A 100.0% 99.5% **VTWIDNNSGCKGACDRVEQYQTPENLKKKWCKDGECYDDILIDSLKQYLATIAKDDDRPRLIVLHQVGSHGPAYYKRAPE**

19 MBL_M6 100.0% 99.5% **VTWIDNNSGCKGACDRVEQYQTPENLKKKWCKDGECYDDILIDSLKQYLATIAKDDDRPRLIVLHQVGSHGPAYYKRAPE**

20 AE3M 97.1% 99.4% **VTWIDNNSGCKGACDRVEQYQTPENLKKKWCKDGECYDDILIDSLKQYLATIAKDDDRPRLIVLHQVGSHGPAYYKRAPE**

21 AB177-VUB 100.0% 99.5% **VTWIDNNSGCKGACDRVEQYQTPENLKKKWCKDGECYDDILIDSLKQYLATIAKDDDRPRLIVLHQVGSHGPAYYKRAPE**

22 Cl300 100.0% 99.5% **VTWIDNNSGCKGACDRVEQYQTPENLKKKWCKDGECYDDILIDSLKQYLATIAKDDDRPRLIVLHQVGSHGPAYYKRAPE**

23 AR_0037 97.1% 99.4% **VTWIDNNSGCKGACDRVEQYQTPENLKKKWCKDGECYDDILIDSLKQYLATIAKDDDRPRLIVLHQVGSHGPAYYKRAPE**

24 AB-A 100.0% 99.5% **VTWIDNNSGCKGACDRVEQYQTPENLKKKWCKDGECYDDILIDSLKQYLATIAKDDDRPRLIVLHQVGSHGPAYYKRAPE**

25 MBL_M9 47.0% 99.2% **VTWIDNNSGCKGACDRVEQYQTPENLKKKWCKDGECYDDILIDSLKQYLATIAKDDDRPRLIVLHQVGSHGPAYYKRAPE**

26 R11 100.0% 99.5% **VTWIDNNSGCKGACDRVEQYQTPENLKKKWCKDGECYDDILIDSLKQYLATIAKDDDRPRLIVLHQVGSHGPAYYKRAPE**

27 MIN-015 100.0% 99.5% **VTWIDNNSGCKGACDRVEQYQTPENLKKKWCKDGECYDDILIDSLKQYLATIAKDDDRPRLIVLHQVGSHGPAYYKRAPE**

cov pid **401**  **. . . . : . . .** **480**

1 ATCC_19606 100.0% 100.0% **AYQPFKPTCDTNAIQGCSQTELLNSYDNTIVYTDHVLSQMINTLKEISKYQTGLWYLSDHGESTGEHGLYLHGSPYAIAP**

2 M02 100.0% 99.5% **AYQPFKPTCDTNAIQGCSQTELLNSYDNTIVYTDHVLSQMINTLKEISKYQTGLWYLSDHGESTGEHGLYLHGSPYAIAP**

3 M11 79.6% 98.4% **AYQPFKPTCDTNAIQGCSQTELLNSYDNTIVYTSLKH-------------------------------------------**

4 M18 100.0% 99.5% **AYQPFKPTCDTNAIQGCSQTELLNSYDNTIVYTDHVLSQMINTLKEISKYQTGLWYLSDHGESTGEHGLYLHGSPYAIAP**

5 MRSN15574 100.0% 99.5% **AYQPFKPTCDTNAIQGCSQTELLNSYDNTIVYTDHVLSQMINTLKEISKYQTGLWYLSDHGESTGEHGLYLHGSPYAIAP**

6 AB-C 100.0% 99.5% **AYQPFKPTCDTNAIQGCSQTELLNSYDNTIVYTDHVLSQMINTLKEISKYQTGLWYLSDHGESTGEHGLYLHGSPYAIAP**

7 ACMH-6201 97.1% 99.4% **AYQPFKPTCDTNAIQGCSQTELLNSYDNTIVYTDHVLSQMINTLKEISKYQTGLWYLSDHGESTGEHGLYLHGSPYAIAP**

8 Survcare112 97.1% 99.4% **AYQPFKPTCDTNAIQGCSQTELLNSYDNTIVYTDHVLSQMINTLKEISKYQTGLWYLSDHGESTGEHGLYLHGSPYAIAP**

9 15953 100.0% 99.5% **AYQPFKPTCDTNAIQGCSQTELLNSYDNTIVYTDHVLSQMINTLKEISKYQTGLWYLSDHGESTGEHGLYLHGSPYAIAP**

10 MBL_M10 100.0% 99.5% **AYQPFKPTCDTNAIQGCSQTELLNSYDNTIVYTDHVLSQMINTLKEISKYQTGLWYLSDHGESTGEHGLYLHGSPYAIAP**

11 AR_0033 97.1% 99.4% **AYQPFKPTCDTNAIQGCSQTELLNSYDNTIVYTDHVLSQMINTLKEISKYQTGLWYLSDHGESTGEHGLYLHGSPYAIAP**

12 17A1955 100.0% 99.5% **AYQPFKPTCDTNAIQGCSQTELLNSYDNTIVYTDHVLSQMINTLKEISKYQTGLWYLSDHGESTGEHGLYLHGSPYAIAP**

13 MBL_M1 100.0% 99.5% **AYQPFKPTCDTNAIQGCSQTELLNSYDNTIVYTDHVLSQMINTLKEISKYQTGLWYLSDHGESTGEHGLYLHGSPYAIAP**

14 15946 100.0% 99.5% **AYQPFKPTCDTNAIQGCSQTELLNSYDNTIVYTDHVLSQMINTLKEISKYQTGLWYLSDHGESTGEHGLYLHGSPYAIAP**

15 AB-B 100.0% 99.5% **AYQPFKPTCDTNAIQGCSQTELLNSYDNTIVYTDHVLSQMINTLKEISKYQTGLWYLSDHGESTGEHGLYLHGSPYAIAP**

16 26 100.0% 99.5% **AYQPFKPTCDTNAIQGCSQTELLNSYDNTIVYTDHVLSQMINTLKEISKYQTGLWYLSDHGESTGEHGLYLHGSPYAIAP**

17 Ab-NDM-1 97.1% 99.4% **AYQPFKPTCDTNAIQGCSQTELLNSYDNTIVYTDHVLSQMINTLKEISKYQTGLWYLSDHGESTGEHGLYLHGSPYAIAP**

18 P116A 100.0% 99.5% **AYQPFKPTCDTNAIQGCSQTELLNSYDNTIVYTDHVLSQMINTLKEISKYQTGLWYLSDHGESTGEHGLYLHGSPYAIAP**

19 MBL_M6 100.0% 99.5% **AYQPFKPTCDTNAIQGCSQTELLNSYDNTIVYTDHVLSQMINTLKEISKYQTGLWYLSDHGESTGEHGLYLHGSPYAIAP**

20 AE3M 97.1% 99.4% **AYQPFKPTCDTNAIQGCSQTELLNSYDNTIVYTDHVLSQMINTLKEISKYQTGLWYLSDHGESTGEHGLYLHGSPYAIAP**

21 AB177-VUB 100.0% 99.5% **AYQPFKPTCDTNAIQGCSQTELLNSYDNTIVYTDHVLSQMINTLKEISKYQTGLWYLSDHGESTGEHGLYLHGSPYAIAP**

22 Cl300 100.0% 99.5% **AYQPFKPTCDTNAIQGCSQTELLNSYDNTIVYTDHVLSQMINTLKEISKYQTGLWYLSDHGESTGEHGLYLHGSPYAIAP**

23 AR_0037 97.1% 99.4% **AYQPFKPTCDTNAIQGCSQTELLNSYDNTIVYTDHVLSQMINTLKEISKYQTGLWYLSDHGESTGEHGLYLHGSPYAIAP**

24 AB-A 100.0% 99.5% **AYQPFKPTCDTNAIQGCSQTELLNSYDNTIVYTDHVLSQMINTLKEISKYQTGLWYLSDHGESTGEHGLYLHGSPYAIAP**

25 MBL_M9 47.0% 99.2% **AYQPFKPTCDTNAIQGCSQTELLNSYDNTIVYTDHVLSQMINTLKEISKYQTGLWYLSDHGESTGEHGLYLHGSPYAIAP**

26 R11 100.0% 99.5% **AYQPFKPTCDTNAIQGCSQTELLNSYDNTIVYTDHVLSQMINTLKEISKYQTGLWYLSDHGESTGEHGLYLHGSPYAIAP**

27 MIN-015 100.0% 99.5% **AYQPFKPTCDTNAIQGCSQTELLNSYDNTIVYTDHVLSQMINTLKEISKYQTGLWYLSDHGESTGEHGLYLHGSPYAIAP**

cov pid **481**  **. 5 . . . . ]** **549**

1 ATCC_19606 100.0% 100.0% **SQQTHVPMIMWFSESWKQHNLAQVNCLSQQTKQKLSQDNLFPSLLSLLDVKTQVINPQLDMLHSCAHVN**

2 M02 100.0% 99.5% **SQQTHVPMIMWFSESWKQHNLAQVNCLSQQTKQKLSQDNLFPSLLSLLDVKTQVINPQLDMLHSCAHVN**

3 M11 79.6% 98.4% **---------------------------------------------------------------------**

4 M18 100.0% 99.5% **SQQTHVPMIMWFSESWKQHNLAQVNCLSQQTKQKLSQDNLFPSLLSLLDVKTQVINPQLDMLHSCAHVN**

5 MRSN15574 100.0% 99.5% **SQQTHVPMIMWFSESWKQHNLAQVNCLSQQTKQKLSQDNLFPSLLSLLDVKTQVINPQLDMLHSCAHVN**

6 AB-C 100.0% 99.5% **SQQTHVPMIMWFSESWKQHNLAQVNCLSQQTKQKLSQDNLFPSLLSLLDVKTQVINPQLDMLHSCAHVN**

7 ACMH-6201 97.1% 99.4% **SQQTHVPMIMWFSESWKQHNLAQVNCLSQQTKQKLSQDNLFPSLLSLLDVKTQVINPQLDMLHSCAHVN**

8 Survcare112 97.1% 99.4% **SQQTHVPMIMWFSESWKQHNLAQVNCLSQQTKQKLSQDNLFPSLLSLLDVKTQVINPQLDMLHSCAHVN**

9 15953 100.0% 99.5% **SQQTHVPMIMWFSESWKQHNLAQVNCLSQQTKQKLSQDNLFPSLLSLLDVKTQVINPQLDMLHSCAHVN**

10 MBL_M10 100.0% 99.5% **SQQTHVPMIMWFSESWKQHNLAQVNCLSQQTKQKLSQDNLFPSLLSLLDVKTQVINPQLDMLHSCAHVN**

11 AR_0033 97.1% 99.4% **SQQTHVPMIMWFSESWKQHNLAQVNCLSQQTKQKLSQDNLFPSLLSLLDVKTQVINPQLDMLHSCAHVN**

12 17A1955 100.0% 99.5% **SQQTHVPMIMWFSESWKQHNLAQVNCLSQQTKQKLSQDNLFPSLLSLLDVKTQVINPQLDMLHSCAHVN**

13 MBL_M1 100.0% 99.5% **SQQTHVPMIMWFSESWKQHNLAQVNCLSQQTKQKLSQDNLFPSLLSLLDVKTQVINPQLDMLHSCAHVN**

14 15946 100.0% 99.5% **SQQTHVPMIMWFSESWKQHNLAQVNCLSQQTKQKLSQDNLFPSLLSLLDVKTQVINPQLDMLHSCAHVN**

15 AB-B 100.0% 99.5% **SQQTHVPMIMWFSESWKQHNLAQVNCLSQQTKQKLSQDNLFPSLLSLLDVKTQVINPQLDMLHSCAHVN**

16 26 100.0% 99.5% **SQQTHVPMIMWFSESWKQHNLAQVNCLSQQTKQKLSQDNLFPSLLSLLDVKTQVINPQLDMLHSCAHVN**

17 Ab-NDM-1 97.1% 99.4% **SQQTHVPMIMWFSESWKQHNLAQVNCLSQQTKQKLSQDNLFPSLLSLLDVKTQVINPQLDMLHSCAHVN**

18 P116A 100.0% 99.5% **SQQTHVPMIMWFSESWKQHNLAQVNCLSQQTKQKLSQDNLFPSLLSLLDVKTQVINPQLDMLHSCAHVN**

19 MBL_M6 100.0% 99.5% **SQQTHVPMIMWFSESWKQHNLAQVNCLSQQTKQKLSQDNLFPSLLSLLDVKTQVINPQLDMLHSCAHVN**

20 AE3M 97.1% 99.4% **SQQTHVPMIMWFSESWKQHNLAQVNCLSQQTKQKLSQDNLFPSLLSLLDVKTQVINPQLDMLHSCAHVN**

21 AB177-VUB 100.0% 99.5% **SQQTHVPMIMWFSESWKQHNLAQVNCLSQQTKQKLSQDNLFPSLLSLLDVKTQVINPQLDMLHSCAHVN**

22 Cl300 100.0% 99.5% **SQQTHVPMIMWFSESWKQHNLAQVNCLSQQTKQKLSQDNLFPSLLSLLDVKTQVINPQLDMLHSCAHVN**

23 AR_0037 97.1% 99.4% **SQQTHVPMIMWFSESWKQHNLAQVNCLSQQTKQKLSQDNLFPSLLSLLDVKTQVINPQLDMLHSCAHVN**

24 AB-A 100.0% 99.5% **SQQTHVPMIMWFSESWKQHNLAQVNCLSQQTKQKLSQDNLFPSLLSLLDVKTQVINPQLDMLHSCAHVN**

25 MBL_M9 47.0% 99.2% **SQQTHVPMIMWFSESWKQHNLAQVNCLSQQTKQKLSQDNLFPSLLSLLDVKTQVINPQLDMLHSCAHVN**

26 R11 100.0% 99.5% **SQQTHVPMIMWFSESWKQHNLAQVNCLSQQTKQKLSQDNLFPSLLSLLDVKTQVINPQLDMLHSCAHVN**

27 MIN-015 100.0% 99.5% **SQQTHVPMIMWFSESWKQHNLAQVNCLSQQTKQKLSQDNLFPSLLSLLDVKTQVINPQLDMLHSCAHVN**

**Supplementary Figure 4: Multiple sequence alignment (MSA) of the predicted amino acid sequence of PmrC carried by ST85^Pas^ (GC9) and close genomes retrieved from the BV-BRC database compared to the respective gene in *A. baumannii* ATCC 19606.** MSA was created by the A multiple alignment viewer MView hosted by the EMBL-EBI; cov, coverage; pid, percent identity.

cov pid  **1** **[ . . . . : . . .** **80**

1 ATCC19606 100.0% 100.0% **MLNFFSTLRNKQISLFMFNLIIAIWLGAILNIGFYHQVHTLTPYFGVKAILFLAATLVILVATYYAVLQILNWKWTAKIF**

2 M10 46.8% 99.2% **--------------------------------------------------------------------------------**

3 M19 97.3% 99.4% **---------------FMFNLIIAIWLGAILNIGFYHQVHTLTPYFGVKAILFLAATLVILVATYYAVLQILNWKWTAKIF**

4 A5 100.0% 99.5% **MLNFFSTLRNKQISLFMFNLIIAIWLGAILNIGFYHQVHTLTPYFGVKAILFLAATLVILVATYYAVLQILNWKWTAKIF**

5 Aci00709 100.0% 99.5% **MLNFFSTLRNKQISLFMFNLIIAIWLGAILNIGFYHQVHTLTPYFGVKAILFLAATLVILVATYYAVLQILNWKWTAKIF**

6 Aci00711 100.0% 99.5% **MLNFFSTLRNKQISLFMFNLIIAIWLGAILNIGFYHQVHTLTPYFGVKAILFLAATLVILVATYYAVLQILNWKWTAKIF**

7 KAB15 100.0% 99.5% **MLNFFSTLRNKQISLFMFNLIIAIWLGAILNIGFYHQVHTLTPYFGVKAILFLAATLVILVATYYAVLQILNWKWTAKIF**

8 PT022 100.0% 99.5% **MLNFFSTLRNKQISLFMFNLIIAIWLGAILNIGFYHQVHTLTPYFGVKAILFLAATLVILVATYYAVLQILNWKWTAKIF**

9 PSU091 100.0% 99.5% **MLNFFSTLRNKQISLFMFNLIIAIWLGAILNIGFYHQVHTLTPYFGVKAILFLAATLVILVATYYAVLQILNWKWTAKIF**

10 151 100.0% 99.5% **MLNFFSTLRNKQISLFMFNLIIAIWLGAILNIGFYHQVHTLTPYFGVKAILFLAATLVILVATYYAVLQILNWKWTAKIF**

11 GML-KP48-AB-TR 100.0% 99.5% **MLNFFSTLRNKQISLFMFNLIIAIWLGAILNIGFYHQVHTLTPYFGVKAILFLAATLVILVATYYAVLQILNWKWTAKIF**

12 198 100.0% 99.5% **MLNFFSTLRNKQISLFMFNLIIAIWLGAILNIGFYHQVHTLTPYFGVKAILFLAATLVILVATYYAVLQILNWKWTAKIF**

13 4300STDY7045706 97.1% 99.4% **----------------MFNLIIAIWLGAILNIGFYHQVHTLTPYFGVKAILFLAATLVILVATYYAVLQILNWKWTAKIF**

14 4300STDY7045763 100.0% 99.5% **MLNFFSTLRNKQISLFMFNLIIAIWLGAILNIGFYHQVHTLTPYFGVKAILFLAATLVILVATYYAVLQILNWKWTAKIF**

15 4300STDY7045798 100.0% 99.5% **MLNFFSTLRNKQISLFMFNLIIAIWLGAILNIGFYHQVHTLTPYFGVKAILFLAATLVILVATYYAVLQILNWKWTAKIF**

16 4300STDY7045799 97.1% 99.4% **----------------MFNLIIAIWLGAILNIGFYHQVHTLTPYFGVKAILFLAATLVILVATYYAVLQILNWKWTAKIF**

17 4300STDY7045808 100.0% 99.5% **MLNFFSTLRNKQISLFMFNLIIAIWLGAILNIGFYHQVHTLTPYFGVKAILFLAATLVILVATYYAVLQILNWKWTAKIF**

18 4300STDY7045806 97.1% 99.4% **----------------MFNLIIAIWLGAILNIGFYHQVHTLTPYFGVKAILFLAATLVILVATYYAVLQILNWKWTAKIF**

19 4300STDY7045811 100.0% 99.5% **MLNFFSTLRNKQISLFMFNLIIAIWLGAILNIGFYHQVHTLTPYFGVKAILFLAATLVILVATYYAVLQILNWKWTAKIF**

20 4300STDY7045829 100.0% 99.5% **MLNFFSTLRNKQISLFMFNLIIAIWLGAILNIGFYHQVHTLTPYFGVKAILFLAATLVILVATYYAVLQILNWKWTAKIF**

21 4300STDY7045870 100.0% 99.5% **MLNFFSTLRNKQISLFMFNLIIAIWLGAILNIGFYHQVHTLTPYFGVKAILFLAATLVILVATYYAVLQILNWKWTAKIF**

22 4300STDY7045866 97.1% 99.4% **----------------MFNLIIAIWLGAILNIGFYHQVHTLTPYFGVKAILFLAATLVILVATYYAVLQILNWKWTAKIF**

23 4300STDY6542380 97.1% 99.4% **----------------MFNLIIAIWLGAILNIGFYHQVHTLTPYFGVKAILFLAATLVILVATYYAVLQILNWKWTAKIF**

cov pid  **81**  **. 1 . . . . : .** **160**

1 ATCC19606 100.0% 100.0% **AILLIFIGGFSSYFVNTLGVIISPDQIQNMVQTDVSEVTDLISLRFVLWTIFFVILPIFLITQVKFKQEKVSRLLLKKVF**

2 M10 46.8% 99.2% **--------------------------------------------------------------------------------**

3 M19 97.3% 99.4% **AILLIFIGGFSSYFVNTLGVIISPDQIQNMVQTDVSEVTDLISLRFVLWTIFFVILPIFLITQVKFKQEKVSRLLLKKVF**

4 A5 100.0% 99.5% **AILLIFIGGFSSYFVNTLGVIISPDQIQNMVQTDVSEVTDLISLRFVLWTIFFVILPIFLITQVKFKQEKVSRLLLKKVF**

5 Aci00709 100.0% 99.5% **AILLIFIGGFSSYFVNTLGVIISPDQIQNMVQTDVSEVTDLISLRFVLWTIFFVILPIFLITQVKFKQEKVSRLLLKKVF**

6 Aci00711 100.0% 99.5% **AILLIFIGGFSSYFVNTLGVIISPDQIQNMVQTDVSEVTDLISLRFVLWTIFFVILPIFLITQVKFKQEKVSRLLLKKVF**

7 KAB15 100.0% 99.5% **AILLIFIGGFSSYFVNTLGVIISPDQIQNMVQTDVSEVTDLISLRFVLWTIFFVILPIFLITQVKFKQEKVSRLLLKKVF**

8 PT022 100.0% 99.5% **AILLIFIGGFSSYFVNTLGVIISPDQIQNMVQTDVSEVTDLISLRFVLWTIFFVILPIFLITQVKFKQEKVSRLLLKKVF**

9 PSU091 100.0% 99.5% **AILLIFIGGFSSYFVNTLGVIISPDQIQNMVQTDVSEVTDLISLRFVLWTIFFVILPIFLITQVKFKQEKVSRLLLKKVF**

10 151 100.0% 99.5% **AILLIFIGGFSSYFVNTLGVIISPDQIQNMVQTDVSEVTDLISLRFVLWTIFFVILPIFLITQVKFKQEKVSRLLLKKVF**

11 GML-KP48-AB-TR 100.0% 99.5% **AILLIFIGGFSSYFVNTLGVIISPDQIQNMVQTDVSEVTDLISLRFVLWTIFFVILPIFLITQVKFKQEKVSRLLLKKVF**

12 198 100.0% 99.5% **AILLIFIGGFSSYFVNTLGVIISPDQIQNMVQTDVSEVTDLISLRFVLWTIFFVILPIFLITQVKFKQEKVSRLLLKKVF**

13 4300STDY7045706 97.1% 99.4% **AILLIFIGGFSSYFVNTLGVIISPDQIQNMVQTDVSEVTDLISLRFVLWTIFFVILPIFLITQVKFKQEKVSRLLLKKVF**

14 4300STDY7045763 100.0% 99.5% **AILLIFIGGFSSYFVNTLGVIISPDQIQNMVQTDVSEVTDLISLRFVLWTIFFVILPIFLITQVKFKQEKVSRLLLKKVF**

15 4300STDY7045798 100.0% 99.5% **AILLIFIGGFSSYFVNTLGVIISPDQIQNMVQTDVSEVTDLISLRFVLWTIFFVILPIFLITQVKFKQEKVSRLLLKKVF**

16 4300STDY7045799 97.1% 99.4% **AILLIFIGGFSSYFVNTLGVIISPDQIQNMVQTDVSEVTDLISLRFVLWTIFFVILPIFLITQVKFKQEKVSRLLLKKVF**

17 4300STDY7045808 100.0% 99.5% **AILLIFIGGFSSYFVNTLGVIISPDQIQNMVQTDVSEVTDLISLRFVLWTIFFVILPIFLITQVKFKQEKVSRLLLKKVF**

18 4300STDY7045806 97.1% 99.4% **AILLIFIGGFSSYFVNTLGVIISPDQIQNMVQTDVSEVTDLISLRFVLWTIFFVILPIFLITQVKFKQEKVSRLLLKKVF**

19 4300STDY7045811 100.0% 99.5% **AILLIFIGGFSSYFVNTLGVIISPDQIQNMVQTDVSEVTDLISLRFVLWTIFFVILPIFLITQVKFKQEKVSRLLLKKVF**

20 4300STDY7045829 100.0% 99.5% **AILLIFIGGFSSYFVNTLGVIISPDQIQNMVQTDVSEVTDLISLRFVLWTIFFVILPIFLITQVKFKQEKVSRLLLKKVF**

21 4300STDY7045870 100.0% 99.5% **AILLIFIGGFSSYFVNTLGVIISPDQIQNMVQTDVSEVTDLISLRFVLWTIFFVILPIFLITQVKFKQEKVSRLLLKKVF**

22 4300STDY7045866 97.1% 99.4% **AILLIFIGGFSSYFVNTLGVIISPDQIQNMVQTDVSEVTDLISLRFVLWTIFFVILPIFLITQVKFKQEKVSRLLLKKVF**

23 4300STDY6542380 97.1% 99.4% **AILLIFIGGFSSYFVNTLGVIISPDQIQNMVQTDVSEVTDLISLRFVLWTIFFVILPIFLITQVKFKQEKVSRLLLKKVF**

cov pid **161**  **. . . 2 . . . .** **240**

1 ATCC19606 100.0% 100.0% **SLVASFAVVGVLLFTYYVDFAAIFREHRDLKGMISPQNSISSLMSYYHKKAPKKNLPLVIYGQDAHQVQRVQKNLPKLMI**

2 M10 46.8% 99.2% **---------------YYVDFAAIFREHRDLKGMISPQNSISSLMSYYHKKAPKKNLPLVIYGQDAHQVQRVQKNLPKLMI**

3 M19 97.3% 99.4% **SLVASFAVVGVLLFTYYVDFAAIFREHRDLKGMISPQNSISSLMSYYHKKAPKKNLPLVIYGQDAHQVQRVQKNLPKLMI**

4 A5 100.0% 99.5% **SLVASFAVVGVLLFTYYVDFAAIFREHRDLKGMISPQNSISSLMSYYHKKAPKKNLPLVIYGQDAHQVQRVQKNLPKLMI**

5 Aci00709 100.0% 99.5% **SLVASFAVVGVLLFTYYVDFAAIFREHRDLKGMISPQNSISSLMSYYHKKAPKKNLPLVIYGQDAHQVQRVQKNLPKLMI**

6 Aci00711 100.0% 99.5% **SLVASFAVVGVLLFTYYVDFAAIFREHRDLKGMISPQNSISSLMSYYHKKAPKKNLPLVIYGQDAHQVQRVQKNLPKLMI**

7 KAB15 100.0% 99.5% **SLVASFAVVGVLLFTYYVDFAAIFREHRDLKGMISPQNSISSLMSYYHKKAPKKNLPLVIYGQDAHQVQRVQKNLPKLMI**

8 PT022 100.0% 99.5% **SLVASFAVVGVLLFTYYVDFAAIFREHRDLKGMISPQNSISSLMSYYHKKAPKKNLPLVIYGQDAHQVQRVQKNLPKLMI**

9 PSU091 100.0% 99.5% **SLVASFAVVGVLLFTYYVDFAAIFREHRDLKGMISPQNSISSLMSYYHKKAPKKNLPLVIYGQDAHQVQRVQKNLPKLMI**

10 151 100.0% 99.5% **SLVASFAVVGVLLFTYYVDFAAIFREHRDLKGMISPQNSISSLMSYYHKKAPKKNLPLVIYGQDAHQVQRVQKNLPKLMI**

11 GML-KP48-AB-TR 100.0% 99.5% **SLVASFAVVGVLLFTYYVDFAAIFREHRDLKGMISPQNSISSLMSYYHKKAPKKNLPLVIYGQDAHQVQRVQKNLPKLMI**

12 198 100.0% 99.5% **SLVASFAVVGVLLFTYYVDFAAIFREHRDLKGMISPQNSISSLMSYYHKKAPKKNLPLVIYGQDAHQVQRVQKNLPKLMI**

13 4300STDY7045706 97.1% 99.4% **SLVASFAVVGVLLFTYYVDFAAIFREHRDLKGMISPQNSISSLMSYYHKKAPKKNLPLVIYGQDAHQVQRVQKNLPKLMI**

14 4300STDY7045763 100.0% 99.5% **SLVASFAVVGVLLFTYYVDFAAIFREHRDLKGMISPQNSISSLMSYYHKKAPKKNLPLVIYGQDAHQVQRVQKNLPKLMI**

15 4300STDY7045798 100.0% 99.5% **SLVASFAVVGVLLFTYYVDFAAIFREHRDLKGMISPQNSISSLMSYYHKKAPKKNLPLVIYGQDAHQVQRVQKNLPKLMI**

16 4300STDY7045799 97.1% 99.4% **SLVASFAVVGVLLFTYYVDFAAIFREHRDLKGMISPQNSISSLMSYYHKKAPKKNLPLVIYGQDAHQVQRVQKNLPKLMI**

17 4300STDY7045808 100.0% 99.5% **SLVASFAVVGVLLFTYYVDFAAIFREHRDLKGMISPQNSISSLMSYYHKKAPKKNLPLVIYGQDAHQVQRVQKNLPKLMI**

18 4300STDY7045806 97.1% 99.4% **SLVASFAVVGVLLFTYYVDFAAIFREHRDLKGMISPQNSISSLMSYYHKKAPKKNLPLVIYGQDAHQVQRVQKNLPKLMI**

19 4300STDY7045811 100.0% 99.5% **SLVASFAVVGVLLFTYYVDFAAIFREHRDLKGMISPQNSISSLMSYYHKKAPKKNLPLVIYGQDAHQVQRVQKNLPKLMI**

20 4300STDY7045829 100.0% 99.5% **SLVASFAVVGVLLFTYYVDFAAIFREHRDLKGMISPQNSISSLMSYYHKKAPKKNLPLVIYGQDAHQVQRVQKNLPKLMI**

21 4300STDY7045870 100.0% 99.5% **SLVASFAVVGVLLFTYYVDFAAIFREHRDLKGMISPQNSISSLMSYYHKKAPKKNLPLVIYGQDAHQVQRVQKNLPKLMI**

22 4300STDY7045866 97.1% 99.4% **SLVASFAVVGVLLFTYYVDFAAIFREHRDLKGMISPQNSISSLMSYYHKKAPKKNLPLVIYGQDAHQVQRVQKNLPKLMI**

23 4300STDY6542380 97.1% 99.4% **SLVASFAVVGVLLFTYYVDFAAIFREHRDLKGMISPQNSISSLMSYYHKKAPKKNLPLVIYGQDAHQVQRVQKNLPKLMI**

cov pid **241**  **: . . . . 3 . .** **320**

1 ATCC19606 100.0% 100.0% **LVVGETARAESFSLNGYAKNTNPELSKQDIFNFSQVSSCGTATAVSVPCMFSGMPRVDYNEQLASHREGLLDIAKRAGYQ**

2 M10 46.8% 99.2% **LVVGETARAESFSLNGYAKNTNPELSKQDIFNFSQVSSCGTATAVSVPCMFSGMPRVDYDEQLASHREGLLDIAKRAGYQ**

3 M19 97.3% 99.4% **LVVGETARAESFSLNGYAKNTNPELSKQDIFNFSQVSSCGTATAVSVPCMFSGMPRVDYDEQLASHREGLLDIAKRAGYQ**

4 A5 100.0% 99.5% **LVVGETARAESFSLNGYAKNTNPELSKQDIFNFSQVSSCGTATAVSVPCMFSGMPRVDYDEQLASHREGLLDIAKRAGYQ**

5 Aci00709 100.0% 99.5% **LVVGETARAESFSLNGYAKNTNPELSKQDIFNFSQVSSCGTATAVSVPCMFSGMPRVDYDEQLASHREGLLDIAKRAGYQ**

6 Aci00711 100.0% 99.5% **LVVGETARAESFSLNGYAKNTNPELSKQDIFNFSQVSSCGTATAVSVPCMFSGMPRVDYDEQLASHREGLLDIAKRAGYQ**

7 KAB15 100.0% 99.5% **LVVGETARAESFSLNGYAKNTNPELSKQDIFNFSQVSSCGTATAVSVPCMFSGMPRVDYDEQLASHREGLLDIAKRAGYQ**

8 PT022 100.0% 99.5% **LVVGETARAESFSLNGYAKNTNPELSKQDIFNFSQVSSCGTATAVSVPCMFSGMPRVDYDEQLASHREGLLDIAKRAGYQ**

9 PSU091 100.0% 99.5% **LVVGETARAESFSLNGYAKNTNPELSKQDIFNFSQVSSCGTATAVSVPCMFSGMPRVDYDEQLASHREGLLDIAKRAGYQ**

10 151 100.0% 99.5% **LVVGETARAESFSLNGYAKNTNPELSKQDIFNFSQVSSCGTATAVSVPCMFSGMPRVDYDEQLASHREGLLDIAKRAGYQ**

11 GML-KP48-AB-TR 100.0% 99.5% **LVVGETARAESFSLNGYAKNTNPELSKQDIFNFSQVSSCGTATAVSVPCMFSGMPRVDYDEQLASHREGLLDIAKRAGYQ**

12 198 100.0% 99.5% **LVVGETARAESFSLNGYAKNTNPELSKQDIFNFSQVSSCGTATAVSVPCMFSGMPRVDYDEQLASHREGLLDIAKRAGYQ**

13 4300STDY7045706 97.1% 99.4% **LVVGETARAESFSLNGYAKNTNPELSKQDIFNFSQVSSCGTATAVSVPCMFSGMPRVDYDEQLASHREGLLDIAKRAGYQ**

14 4300STDY7045763 100.0% 99.5% **LVVGETARAESFSLNGYAKNTNPELSKQDIFNFSQVSSCGTATAVSVPCMFSGMPRVDYDEQLASHREGLLDIAKRAGYQ**

15 4300STDY7045798 100.0% 99.5% **LVVGETARAESFSLNGYAKNTNPELSKQDIFNFSQVSSCGTATAVSVPCMFSGMPRVDYDEQLASHREGLLDIAKRAGYQ**

16 4300STDY7045799 97.1% 99.4% **LVVGETARAESFSLNGYAKNTNPELSKQDIFNFSQVSSCGTATAVSVPCMFSGMPRVDYDEQLASHREGLLDIAKRAGYQ**

17 4300STDY7045808 100.0% 99.5% **LVVGETARAESFSLNGYAKNTNPELSKQDIFNFSQVSSCGTATAVSVPCMFSGMPRVDYDEQLASHREGLLDIAKRAGYQ**

18 4300STDY7045806 97.1% 99.4% **LVVGETARAESFSLNGYAKNTNPELSKQDIFNFSQVSSCGTATAVSVPCMFSGMPRVDYDEQLASHREGLLDIAKRAGYQ**

19 4300STDY7045811 100.0% 99.5% **LVVGETARAESFSLNGYAKNTNPELSKQDIFNFSQVSSCGTATAVSVPCMFSGMPRVDYDEQLASHREGLLDIAKRAGYQ**

20 4300STDY7045829 100.0% 99.5% **LVVGETARAESFSLNGYAKNTNPELSKQDIFNFSQVSSCGTATAVSVPCMFSGMPRVDYDEQLASHREGLLDIAKRAGYQ**

21 4300STDY7045870 100.0% 99.5% **LVVGETARAESFSLNGYAKNTNPELSKQDIFNFSQVSSCGTATAVSVPCMFSGMPRVDYDEQLASHREGLLDIAKRAGYQ**

22 4300STDY7045866 97.1% 99.4% **LVVGETARAESFSLNGYAKNTNPELSKQDIFNFSQVSSCGTATAVSVPCMFSGMPRVDYDEQLASHREGLLDIAKRAGYQ**

23 4300STDY6542380 97.1% 99.4% **LVVGETARAESFSLNGYAKNTNPELSKQDIFNFSQVSSCGTATAVSVPCMFSGMPRVDYDEQLASHREGLLDIAKRAGYQ**

cov pid **321**  **. . : . . . . 4** **400**

1 ATCC19606 100.0% 100.0% **VTWIDNNSGCKGACDRVEQYQIPENLKKKWCKDGECYDDILIDSLKQYLATIAKDDDRPRLIVLHQVGSHGPAYYKRAPE**

2 M10 46.8% 99.2% **VTWIDNNSGCKGACDRVEQYQIPENLKKKWCKDGECYDDILIDSLKQYLSTIAKDDDRPRLIVLHQVGSHGPAYYKRAPE**

3 M19 97.3% 99.4% **VTWIDNNSGCKGACDRVEQYQIPENLKKKWCKDGECYDDILIDSLKQYLSTIAKDDDRPRLIVLHQVGSHGPAYYKRAPE**

4 A5 100.0% 99.5% **VTWIDNNSGCKGACDRVEQYQIPENLKKKWCKDGECYDDILIDSLKQYLSTIAKDDDRPRLIVLHQVGSHGPAYYKRAPE**

5 Aci00709 100.0% 99.5% **VTWIDNNSGCKGACDRVEQYQIPENLKKKWCKDGECYDDILIDSLKQYLSTIAKDDDRPRLIVLHQVGSHGPAYYKRAPE**

6 Aci00711 100.0% 99.5% **VTWIDNNSGCKGACDRVEQYQIPENLKKKWCKDGECYDDILIDSLKQYLSTIAKDDDRPRLIVLHQVGSHGPAYYKRAPE**

7 KAB15 100.0% 99.5% **VTWIDNNSGCKGACDRVEQYQIPENLKKKWCKDGECYDDILIDSLKQYLSTIAKDDDRPRLIVLHQVGSHGPAYYKRAPE**

8 PT022 100.0% 99.5% **VTWIDNNSGCKGACDRVEQYQIPENLKKKWCKDGECYDDILIDSLKQYLSTIAKDDDRPRLIVLHQVGSHGPAYYKRAPE**

9 PSU091 100.0% 99.5% **VTWIDNNSGCKGACDRVEQYQIPENLKKKWCKDGECYDDILIDSLKQYLSTIAKDDDRPRLIVLHQVGSHGPAYYKRAPE**

10 151 100.0% 99.5% **VTWIDNNSGCKGACDRVEQYQIPENLKKKWCKDGECYDDILIDSLKQYLSTIAKDDDRPRLIVLHQVGSHGPAYYKRAPE**

11 GML-KP48-AB-TR 100.0% 99.5% **VTWIDNNSGCKGACDRVEQYQIPENLKKKWCKDGECYDDILIDSLKQYLSTIAKDDDRPRLIVLHQVGSHGPAYYKRAPE**

12 198 100.0% 99.5% **VTWIDNNSGCKGACDRVEQYQIPENLKKKWCKDGECYDDILIDSLKQYLSTIAKDDDRPRLIVLHQVGSHGPAYYKRAPE**

13 4300STDY7045706 97.1% 99.4% **VTWIDNNSGCKGACDRVEQYQIPENLKKKWCKDGECYDDILIDSLKQYLSTIAKDDDRPRLIVLHQVGSHGPAYYKRAPE**

14 4300STDY7045763 100.0% 99.5% **VTWIDNNSGCKGACDRVEQYQIPENLKKKWCKDGECYDDILIDSLKQYLSTIAKDDDRPRLIVLHQVGSHGPAYYKRAPE**

15 4300STDY7045798 100.0% 99.5% **VTWIDNNSGCKGACDRVEQYQIPENLKKKWCKDGECYDDILIDSLKQYLSTIAKDDDRPRLIVLHQVGSHGPAYYKRAPE**

16 4300STDY7045799 97.1% 99.4% **VTWIDNNSGCKGACDRVEQYQIPENLKKKWCKDGECYDDILIDSLKQYLSTIAKDDDRPRLIVLHQVGSHGPAYYKRAPE**

17 4300STDY7045808 100.0% 99.5% **VTWIDNNSGCKGACDRVEQYQIPENLKKKWCKDGECYDDILIDSLKQYLSTIAKDDDRPRLIVLHQVGSHGPAYYKRAPE**

18 4300STDY7045806 97.1% 99.4% **VTWIDNNSGCKGACDRVEQYQIPENLKKKWCKDGECYDDILIDSLKQYLSTIAKDDDRPRLIVLHQVGSHGPAYYKRAPE**

19 4300STDY7045811 100.0% 99.5% **VTWIDNNSGCKGACDRVEQYQIPENLKKKWCKDGECYDDILIDSLKQYLSTIAKDDDRPRLIVLHQVGSHGPAYYKRAPE**

20 4300STDY7045829 100.0% 99.5% **VTWIDNNSGCKGACDRVEQYQIPENLKKKWCKDGECYDDILIDSLKQYLSTIAKDDDRPRLIVLHQVGSHGPAYYKRAPE**

21 4300STDY7045870 100.0% 99.5% **VTWIDNNSGCKGACDRVEQYQIPENLKKKWCKDGECYDDILIDSLKQYLSTIAKDDDRPRLIVLHQVGSHGPAYYKRAPE**

22 4300STDY7045866 97.1% 99.4% **VTWIDNNSGCKGACDRVEQYQIPENLKKKWCKDGECYDDILIDSLKQYLSTIAKDDDRPRLIVLHQVGSHGPAYYKRAPE**

23 4300STDY6542380 97.1% 99.4% **VTWIDNNSGCKGACDRVEQYQIPENLKKKWCKDGECYDDILIDSLKQYLSTIAKDDDRPRLIVLHQVGSHGPAYYKRAPE**

cov pid **401**  **. . . . : . . .** **480**

1 ATCC19606 100.0% 100.0% **AYQPFKPTCDTNAIQGCSQTELLNSYDNTIVYTDHVLSQMINTLKEISKYQTGLWYLSDHGESTGEHGLYLHGSPYAIAP**

2 M10 46.8% 99.2% **AYQPFKPTCDTNAIQGCSQTELLNSYDNTIVY------------------------------------------------**

3 M19 97.3% 99.4% **AYQPFKPTCDTNAIQGCSQTELLNSYDNTIVYTDHVLSQMINTLKEISKYQTGLWYLSDHGESTGEHGLYLHGSPYAIAP**

4 A5 100.0% 99.5% **AYQPFKPTCDTNAIQGCSQTELLNSYDNTIVYTDHVLSQMINTLKEISKYQTGLWYLSDHGESTGEHGLYLHGSPYAIAP**

5 Aci00709 100.0% 99.5% **AYQPFKPTCDTNAIQGCSQTELLNSYDNTIVYTDHVLSQMINTLKEISKYQTGLWYLSDHGESTGEHGLYLHGSPYAIAP**

6 Aci00711 100.0% 99.5% **AYQPFKPTCDTNAIQGCSQTELLNSYDNTIVYTDHVLSQMINTLKEISKYQTGLWYLSDHGESTGEHGLYLHGSPYAIAP**

7 KAB15 100.0% 99.5% **AYQPFKPTCDTNAIQGCSQTELLNSYDNTIVYTDHVLSQMINTLKEISKYQTGLWYLSDHGESTGEHGLYLHGSPYAIAP**

8 PT022 100.0% 99.5% **AYQPFKPTCDTNAIQGCSQTELLNSYDNTIVYTDHVLSQMINTLKEISKYQTGLWYLSDHGESTGEHGLYLHGSPYAIAP**

9 PSU091 100.0% 99.5% **AYQPFKPTCDTNAIQGCSQTELLNSYDNTIVYTDHVLSQMINTLKEISKYQTGLWYLSDHGESTGEHGLYLHGSPYAIAP**

10 151 100.0% 99.5% **AYQPFKPTCDTNAIQGCSQTELLNSYDNTIVYTDHVLSQMINTLKEISKYQTGLWYLSDHGESTGEHGLYLHGSPYAIAP**

11 GML-KP48-AB-TR 100.0% 99.5% **AYQPFKPTCDTNAIQGCSQTELLNSYDNTIVYTDHVLSQMINTLKEISKYQTGLWYLSDHGESTGEHGLYLHGSPYAIAP**

12 198 100.0% 99.5% **AYQPFKPTCDTNAIQGCSQTELLNSYDNTIVYTDHVLSQMINTLKEISKYQTGLWYLSDHGESTGEHGLYLHGSPYAIAP**

13 4300STDY7045706 97.1% 99.4% **AYQPFKPTCDTNAIQGCSQTELLNSYDNTIVYTDHVLSQMINTLKEISKYQTGLWYLSDHGESTGEHGLYLHGSPYAIAP**

14 4300STDY7045763 100.0% 99.5% **AYQPFKPTCDTNAIQGCSQTELLNSYDNTIVYTDHVLSQMINTLKEISKYQTGLWYLSDHGESTGEHGLYLHGSPYAIAP**

15 4300STDY7045798 100.0% 99.5% **AYQPFKPTCDTNAIQGCSQTELLNSYDNTIVYTDHVLSQMINTLKEISKYQTGLWYLSDHGESTGEHGLYLHGSPYAIAP**

16 4300STDY7045799 97.1% 99.4% **AYQPFKPTCDTNAIQGCSQTELLNSYDNTIVYTDHVLSQMINTLKEISKYQTGLWYLSDHGESTGEHGLYLHGSPYAIAP**

17 4300STDY7045808 100.0% 99.5% **AYQPFKPTCDTNAIQGCSQTELLNSYDNTIVYTDHVLSQMINTLKEISKYQTGLWYLSDHGESTGEHGLYLHGSPYAIAP**

18 4300STDY7045806 97.1% 99.4% **AYQPFKPTCDTNAIQGCSQTELLNSYDNTIVYTDHVLSQMINTLKEISKYQTGLWYLSDHGESTGEHGLYLHGSPYAIAP**

19 4300STDY7045811 100.0% 99.5% **AYQPFKPTCDTNAIQGCSQTELLNSYDNTIVYTDHVLSQMINTLKEISKYQTGLWYLSDHGESTGEHGLYLHGSPYAIAP**

20 4300STDY7045829 100.0% 99.5% **AYQPFKPTCDTNAIQGCSQTELLNSYDNTIVYTDHVLSQMINTLKEISKYQTGLWYLSDHGESTGEHGLYLHGSPYAIAP**

21 4300STDY7045870 100.0% 99.5% **AYQPFKPTCDTNAIQGCSQTELLNSYDNTIVYTDHVLSQMINTLKEISKYQTGLWYLSDHGESTGEHGLYLHGSPYAIAP**

22 4300STDY7045866 97.1% 99.4% **AYQPFKPTCDTNAIQGCSQTELLNSYDNTIVYTDHVLSQMINTLKEISKYQTGLWYLSDHGESTGEHGLYLHGSPYAIAP**

23 4300STDY6542380 97.1% 99.4% **AYQPFKPTCDTNAIQGCSQTELLNSYDNTIVYTDHVLSQMINTLKEISKYQTGLWYLSDHGESTGEHGLYLHGSPYAIAP**

cov pid **481**  **. 5 . . . . ]** **549**

1 ATCC19606 100.0% 100.0% **SQQTHVPMIMWFSESWKQHNLAQVNCLSQQTKQKLSQDNLFPSLLSLLDVKTQVINPQLDMLHSCAHVN**

2 M10 46.8% 99.2% **---------------------------------------------------------------------**

3 M19 97.3% 99.4% **SQQTHIPMIMWFSESWKQHNLAQVNCLSQQTKQKLSQDNLFPSLLSLLDVKTQVINPQLDMLHSCAHVN**

4 A5 100.0% 99.5% **SQQTHIPMIMWFSESWKQHNLAQVNCLSQQTKQKLSQDNLFPSLLSLLDVKTQVINPQLDMLHSCAHVN**

5 Aci00709 100.0% 99.5% **SQQTHIPMIMWFSESWKQHNLAQVNCLSQQTKQKLSQDNLFPSLLSLLDVKTQVINPQLDMLHSCAHVN**

6 Aci00711 100.0% 99.5% **SQQTHIPMIMWFSESWKQHNLAQVNCLSQQTKQKLSQDNLFPSLLSLLDVKTQVINPQLDMLHSCAHVN**

7 KAB15 100.0% 99.5% **SQQTHIPMIMWFSESWKQHNLAQVNCLSQQTKQKLSQDNLFPSLLSLLDVKTQVINPQLDMLHSCAHVN**

8 PT022 100.0% 99.5% **SQQTHIPMIMWFSESWKQHNLAQVNCLSQQTKQKLSQDNLFPSLLSLLDVKTQVINPQLDMLHSCAHVN**

9 PSU091 100.0% 99.5% **SQQTHIPMIMWFSESWKQHNLAQVNCLSQQTKQKLSQDNLFPSLLSLLDVKTQVINPQLDMLHSCAHVN**

10 151 100.0% 99.5% **SQQTHIPMIMWFSESWKQHNLAQVNCLSQQTKQKLSQDNLFPSLLSLLDVKTQVINPQLDMLHSCAHVN**

11 GML-KP48-AB-TR 100.0% 99.5% **SQQTHIPMIMWFSESWKQHNLAQVNCLSQQTKQKLSQDNLFPSLLSLLDVKTQVINPQLDMLHSCAHVN**

12 198 100.0% 99.5% **SQQTHIPMIMWFSESWKQHNLAQVNCLSQQTKQKLSQDNLFPSLLSLLDVKTQVINPQLDMLHSCAHVN**

13 4300STDY7045706 97.1% 99.4% **SQQTHIPMIMWFSESWKQHNLAQVNCLSQQTKQKLSQDNLFPSLLSLLDVKTQVINPQLDMLHSCAHVN**

14 4300STDY7045763 100.0% 99.5% **SQQTHIPMIMWFSESWKQHNLAQVNCLSQQTKQKLSQDNLFPSLLSLLDVKTQVINPQLDMLHSCAHVN**

15 4300STDY7045798 100.0% 99.5% **SQQTHIPMIMWFSESWKQHNLAQVNCLSQQTKQKLSQDNLFPSLLSLLDVKTQVINPQLDMLHSCAHVN**

16 4300STDY7045799 97.1% 99.4% **SQQTHIPMIMWFSESWKQHNLAQVNCLSQQTKQKLSQDNLFPSLLSLLDVKTQVINPQLDMLHSCAHVN**

17 4300STDY7045808 100.0% 99.5% **SQQTHIPMIMWFSESWKQHNLAQVNCLSQQTKQKLSQDNLFPSLLSLLDVKTQVINPQLDMLHSCAHVN**

18 4300STDY7045806 97.1% 99.4% **SQQTHIPMIMWFSESWKQHNLAQVNCLSQQTKQKLSQDNLFPSLLSLLDVKTQVINPQLDMLHSCAHVN**

19 4300STDY7045811 100.0% 99.5% **SQQTHIPMIMWFSESWKQHNLAQVNCLSQQTKQKLSQDNLFPSLLSLLDVKTQVINPQLDMLHSCAHVN**

20 4300STDY7045829 100.0% 99.5% **SQQTHIPMIMWFSESWKQHNLAQVNCLSQQTKQKLSQDNLFPSLLSLLDVKTQVINPQLDMLHSCAHVN**

21 4300STDY7045870 100.0% 99.5% **SQQTHIPMIMWFSESWKQHNLAQVNCLSQQTKQKLSQDNLFPSLLSLLDVKTQVINPQLDMLHSCAHVN**

22 4300STDY7045866 97.1% 99.4% **SQQTHIPMIMWFSESWKQHNLAQVNCLSQQTKQKLSQDNLFPSLLSLLDVKTQVINPQLDMLHSCAHVN**

23 4300STDY6542380 97.1% 99.4% **SQQTHIPMIMWFSESWKQHNLAQVNCLSQQTKQKLSQDNLFPSLLSLLDVKTQVINPQLDMLHSCAHVN**

**Supplementary Figure 5: Multiple sequence alignment (MSA) of the predicted amino acid sequence of PmrC carried by ST164^Pas^ and the phylogenetically related isolate M10 and close genomes retrieved from the BV-BRC database compared to the respective gene in *A. baumannii* ATCC 19606.** MSA was created by the A multiple alignment viewer MView hosted by the EMBL-EBI; cov, coverage; pid, percent identity.

cov pid  **1** **[ . . . . : . . .** **80**

1 ATCC19606 100.0% 100.0% **MHYSLKKRLIWGTSIFSVILGCILIFSAYKVALQEVDEILDTQMKYLAERTAEHPLKTVSSKFDFHKTYHEEDLFIDIWA**

2 M06 100.0% 98.9% **MHYSLKKRLIWGTSIFSVILGCILIFSAYKVALQEVDEILDTQMKYLAERTAEHPLKTVSSKFDFHKTYHEEDLFIDIWA**

3 M09 100.0% 99.5% **MHYSLKKRLIWGTSIFSVILGCILIFSAYKVALQEVDEILDTQMKYLAERTAEHPLKTVSSKFDFHKTYHEEDLFIDIWA**

4 M12 100.0% 99.5% **MHYSLKKRLIWGTSIFSVILGCILIFSAYKVALQEVDEILDTQMKYLAERTAEHPLKTVSSKFDFHKTYHEEDLFIDIWA**

5 M15 100.0% 99.5% **MHYSLKKRLIWGTSIFSVILGCILIFSAYKVALQEVDEILDTQMKYLAERTAEHPLKTVSSKFDFHKTYHEEDLFIDIWA**

6 SRR3222490 100.0% 99.5% **MHYSLKKRLIWGTSIFSVILGCILIFSAYKVALQEVDEILDTQMKYLAERTAEHPLKTVSSKFDFHKTYHEEDLFIDIWA**

7 SRR3227013 100.0% 99.5% **MHYSLKKRLIWGTSIFSVILGCILIFSAYKVALQEVDEILDTQMKYLAERTAEHPLKTVSSKFDFHKTYHEEDLFIDIWA**

8 SRR3228488 100.0% 99.5% **MHYSLKKRLIWGTSIFSVILGCILIFSAYKVALQEVDEILDTQMKYLAERTAEHPLKTVSSKFDFHKTYHEEDLFIDIWA**

9 SRR3228565 100.0% 99.5% **MHYSLKKRLIWGTSIFSVILGCILIFSAYKVALQEVDEILDTQMKYLAERTAEHPLKTVSSKFDFHKTYHEEDLFIDIWA**

10 A18 100.0% 99.5% **MHYSLKKRLIWGTSIFSVILGCILIFSAYKVALQEVDEILDTQMKYLAERTAEHPLKTVSSKFDFHKTYHEEDLFIDIWA**

11 15A1042 100.0% 99.5% **MHYSLKKRLIWGTSIFSVILGCILIFSAYKVALQEVDEILDTQMKYLAERTAEHPLKTVSSKFDFHKTYHEEDLFIDIWA**

12 MRSN7133 100.0% 99.5% **MHYSLKKRLIWGTSIFSVILGCILIFSAYKVALQEVDEILDTQMKYLAERTAEHPLKTVSSKFDFHKTYHEEDLFIDIWA**

13 MRSN7130 100.0% 99.5% **MHYSLKKRLIWGTSIFSVILGCILIFSAYKVALQEVDEILDTQMKYLAERTAEHPLKTVSSKFDFHKTYHEEDLFIDIWA**

14 MRSN7202 100.0% 99.5% **MHYSLKKRLIWGTSIFSVILGCILIFSAYKVALQEVDEILDTQMKYLAERTAEHPLKTVSSKFDFHKTYHEEDLFIDIWA**

15 MRSN7224 100.0% 99.5% **MHYSLKKRLIWGTSIFSVILGCILIFSAYKVALQEVDEILDTQMKYLAERTAEHPLKTVSSKFDFHKTYHEEDLFIDIWA**

16 OIFC074 100.0% 99.5% **MHYSLKKRLIWGTSIFSVILGCILIFSAYKVALQEVDEILDTQMKYLAERTAEHPLKTVSSKFDFHKTYHEEDLFIDIWA**

cov pid  **81**  **. 1 . . . . : .** **160**

1 ATCC19606 100.0% 100.0% **YKDQAHLSHHLHLLVPPVEQAGFYSHKTAQGIVRTYVLPLKDYQIQVSQQERVREAFAWELAGSMFIPYLIILPFAIFAL**

2 M06 100.0% 98.9% **YKDQAHLSHHLHLLVPPVEQAGFYSHKTAKGIVRTYVLPLKDYQIQVSQQERVREAFAWELACSMCIPYLIILPFAIFAL**

3 M09 100.0% 99.5% **YKDQAHLSHHLHLLVPPVEQAGFYSHKTAQGIVRTYVLPLKDYQIQVSQQERVREAFAWELAGSMFIPYLIILPFAIFAL**

4 M12 100.0% 99.5% **YKDQAHLSHHLHLLVPPVEQAGFYSHKTAQGIVRTYVLPLKDYQIQVSQQERVREAFAWELAGSMFIPYLIILPFAIFAL**

5 M15 100.0% 99.5% **YKDQAHLSHHLHLLVPPVEQAGFYSHKTAQGIVRTYVLPLKDYQIQVSQQERVREAFAWELAGSMFIPYLIILPFAIFAL**

6 SRR3222490 100.0% 99.5% **YKDQAHLSHHLHLLVPPVEQAGFYSHKTAQGIVRTYVLPLKDYQIQVSQQERVREAFAWELAGSMFIPYLIILPFAIFAL**

7 SRR3227013 100.0% 99.5% **YKDQAHLSHHLHLLVPPVEQAGFYSHKTAQGIVRTYVLPLKDYQIQVSQQERVREAFAWELAGSMFIPYLIILPFAIFAL**

8 SRR3228488 100.0% 99.5% **YKDQAHLSHHLHLLVPPVEQAGFYSHKTAQGIVRTYVLPLKDYQIQVSQQERVREAFAWELAGSMFIPYLIILPFAIFAL**

9 SRR3228565 100.0% 99.5% **YKDQAHLSHHLHLLVPPVEQAGFYSHKTAQGIVRTYVLPLKDYQIQVSQQERVREAFAWELAGSMFIPYLIILPFAIFAL**

10 A18 100.0% 99.5% **YKDQAHLSHHLHLLVPPVEQAGFYSHKTAQGIVRTYVLPLKDYQIQVSQQERVREAFAWELAGSMFIPYLIILPFAIFAL**

11 15A1042 100.0% 99.5% **YKDQAHLSHHLHLLVPPVEQAGFYSHKTAQGIVRTYVLPLKDYQIQVSQQERVREAFAWELAGSMFIPYLIILPFAIFAL**

12 MRSN7133 100.0% 99.5% **YKDQAHLSHHLHLLVPPVEQAGFYSHKTAQGIVRTYVLPLKDYQIQVSQQERVREAFAWELAGSMFIPYLIILPFAIFAL**

13 MRSN7130 100.0% 99.5% **YKDQAHLSHHLHLLVPPVEQAGFYSHKTAQGIVRTYVLPLKDYQIQVSQQERVREAFAWELAGSMFIPYLIILPFAIFAL**

14 MRSN7202 100.0% 99.5% **YKDQAHLSHHLHLLVPPVEQAGFYSHKTAQGIVRTYVLPLKDYQIQVSQQERVREAFAWELAGSMFIPYLIILPFAIFAL**

15 MRSN7224 100.0% 99.5% **YKDQAHLSHHLHLLVPPVEQAGFYSHKTAQGIVRTYVLPLKDYQIQVSQQERVREAFAWELAGSMFIPYLIILPFAIFAL**

16 OIFC074 100.0% 99.5% **YKDQAHLSHHLHLLVPPVEQAGFYSHKTAQGIVRTYVLPLKDYQIQVSQQERVREAFAWELAGSMFIPYLIILPFAIFAL**

cov pid **161**  **. . . 2 . . . .** **240**

1 ATCC19606 100.0% 100.0% **AAIIRRGLKPIDDFKNELKERDSEELTPIEVHDYPQELLPTIDEMNRLFERISKAQNEQKQFIADAAHELRTPVTALNLQ**

2 M06 100.0% 98.9% **AAIIRRGLKPIDDFKNELKERDSEELTPIEVHDYPQELLPTIDEMNRLFERISKAQNEQKQFIADAAHELRTPVTALNLQ**

3 M09 100.0% 99.5% **AAIIRRGLKPIDDFKNELKERDSEELTPIEVHDYPQELLPTIDEMNRLFERISKAQNEQKQFIADAAHELRTPVTALNLQ**

4 M12 100.0% 99.5% **AAIIRRGLKPIDDFKNELKERDSEELTPIEVHDYPQELLPTIDEMNRLFERISKAQNEQKQFIADAAHELRTPVTALNLQ**

5 M15 100.0% 99.5% **AAIIRRGLKPIDDFKNELKERDSEELTPIEVHDYPQELLPTIDEMNRLFERISKAQNEQKQFIADAAHELRTPVTALNLQ**

6 SRR3222490 100.0% 99.5% **AAIIRRGLKPIDDFKNELKERDSEELTPIEVHDYPQELLPTIDEMNRLFERISKAQNEQKQFIADAAHELRTPVTALNLQ**

7 SRR3227013 100.0% 99.5% **AAIIRRGLKPIDDFKNELKERDSEELTPIEVHDYPQELLPTIDEMNRLFERISKAQNEQKQFIADAAHELRTPVTALNLQ**

8 SRR3228488 100.0% 99.5% **AAIIRRGLKPIDDFKNELKERDSEELTPIEVHDYPQELLPTIDEMNRLFERISKAQNEQKQFIADAAHELRTPVTALNLQ**

9 SRR3228565 100.0% 99.5% **AAIIRRGLKPIDDFKNELKERDSEELTPIEVHDYPQELLPTIDEMNRLFERISKAQNEQKQFIADAAHELRTPVTALNLQ**

10 A18 100.0% 99.5% **AAIIRRGLKPIDDFKNELKERDSEELTPIEVHDYPQELLPTIDEMNRLFERISKAQNEQKQFIADAAHELRTPVTALNLQ**

11 15A1042 100.0% 99.5% **AAIIRRGLKPIDDFKNELKERDSEELTPIEVHDYPQELLPTIDEMNRLFERISKAQNEQKQFIADAAHELRTPVTALNLQ**

12 MRSN7133 100.0% 99.5% **AAIIRRGLKPIDDFKNELKERDSEELTPIEVHDYPQELLPTIDEMNRLFERISKAQNEQKQFIADAAHELRTPVTALNLQ**

13 MRSN7130 100.0% 99.5% **AAIIRRGLKPIDDFKNELKERDSEELTPIEVHDYPQELLPTIDEMNRLFERISKAQNEQKQFIADAAHELRTPVTALNLQ**

14 MRSN7202 100.0% 99.5% **AAIIRRGLKPIDDFKNELKERDSEELTPIEVHDYPQELLPTIDEMNRLFERISKAQNEQKQFIADAAHELRTPVTALNLQ**

15 MRSN7224 100.0% 99.5% **AAIIRRGLKPIDDFKNELKERDSEELTPIEVHDYPQELLPTIDEMNRLFERISKAQNEQKQFIADAAHELRTPVTALNLQ**

16 OIFC074 100.0% 99.5% **AAIIRRGLKPIDDFKNELKERDSEELTPIEVHDYPQELLPTIDEMNRLFERISKAQNEQKQFIADAAHELRTPVTALNLQ**

cov pid **241**  **: . . . . 3 . .** **320**

1 ATCC19606 100.0% 100.0% **TKILLSQFPEHESLQNLSKGLARIQHLVTQLLALAKQDVTLSMVEPTGYFQLNDVALNCVEQLVNLAMQKEIDLGFVRNE**

2 M06 100.0% 98.9% **TKILLSQFPEHESLQNLSKGLARIQHLVTQLLALAKQDVTLSMVEPTGYFQLNDVALNCVEQLVNLAMQKEIDLGFVRNE**

3 M09 100.0% 99.5% **TKILLSQFPEHESLQNLSKGLARIQHLVTQLLALAKQDVTLSMVEPTGYFQLNDVALNCVEQLVNLAMQKEIDLGFVRNE**

4 M12 100.0% 99.5% **TKILLSQFPEHESLQNLSKGLARIQHLVTQLLALAKQDVTLSMVEPTGYFQLNDVALNCVEQLVNLAMQKEIDLGFVRNE**

5 M15 100.0% 99.5% **TKILLSQFPEHESLQNLSKGLARIQHLVTQLLALAKQDVTLSMVEPTGYFQLNDVALNCVEQLVNLAMQKEIDLGFVRNE**

6 SRR3222490 100.0% 99.5% **TKILLSQFPEHESLQNLSKGLARIQHLVTQLLALAKQDVTLSMVEPTGYFQLNDVALNCVEQLVNLAMQKEIDLGFVRNE**

7 SRR3227013 100.0% 99.5% **TKILLSQFPEHESLQNLSKGLARIQHLVTQLLALAKQDVTLSMVEPTGYFQLNDVALNCVEQLVNLAMQKEIDLGFVRNE**

8 SRR3228488 100.0% 99.5% **TKILLSQFPEHESLQNLSKGLARIQHLVTQLLALAKQDVTLSMVEPTGYFQLNDVALNCVEQLVNLAMQKEIDLGFVRNE**

9 SRR3228565 100.0% 99.5% **TKILLSQFPEHESLQNLSKGLARIQHLVTQLLALAKQDVTLSMVEPTGYFQLNDVALNCVEQLVNLAMQKEIDLGFVRNE**

10 A18 100.0% 99.5% **TKILLSQFPEHESLQNLSKGLARIQHLVTQLLALAKQDVTLSMVEPTGYFQLNDVALNCVEQLVNLAMQKEIDLGFVRNE**

11 15A1042 100.0% 99.5% **TKILLSQFPEHESLQNLSKGLARIQHLVTQLLALAKQDVTLSMVEPTGYFQLNDVALNCVEQLVNLAMQKEIDLGFVRNE**

12 MRSN7133 100.0% 99.5% **TKILLSQFPEHESLQNLSKGLARIQHLVTQLLALAKQDVTLSMVEPTGYFQLNDVALNCVEQLVNLAMQKEIDLGFVRNE**

13 MRSN7130 100.0% 99.5% **TKILLSQFPEHESLQNLSKGLARIQHLVTQLLALAKQDVTLSMVEPTGYFQLNDVALNCVEQLVNLAMQKEIDLGFVRNE**

14 MRSN7202 100.0% 99.5% **TKILLSQFPEHESLQNLSKGLARIQHLVTQLLALAKQDVTLSMVEPTGYFQLNDVALNCVEQLVNLAMQKEIDLGFVRNE**

15 MRSN7224 100.0% 99.5% **TKILLSQFPEHESLQNLSKGLARIQHLVTQLLALAKQDVTLSMVEPTGYFQLNDVALNCVEQLVNLAMQKEIDLGFVRNE**

16 OIFC074 100.0% 99.5% **TKILLSQFPEHESLQNLSKGLARIQHLVTQLLALAKQDVTLSMVEPTGYFQLNDVALNCVEQLVNLAMQKEIDLGFVRNE**

cov pid **321**  **. . : . . . . 4** **400**

1 ATCC19606 100.0% 100.0% **PIEMHSIEPTVHSIIFNLIDNAIKYTPHQGVINISVYTDPDHYACIQIEDSGAGIDPENYDKVLKRFYRVHHHLEVGSGL**

2 M06 100.0% 98.9% **PIEMHSIEPTVHSIIFNLIDNAIKYTPHQGVINISVYTDQDHYACIQIEDSGAGIDPENYDKVLKRFYRVHHHLEVGSGL**

3 M09 100.0% 99.5% **PIEMHSIEPTVHSIIFNLIDNAIKYTPHQGVINISVYTDQDHYACIQIEDSGAGIDPENYDKVLKRFYRVHHHLEVGSGL**

4 M12 100.0% 99.5% **PIEMHSIEPTVHSIIFNLIDNAIKYTPHQGVINISVYTDQDHYACIQIEDSGAGIDPENYDKVLKRFYRVHHHLEVGSGL**

5 M15 100.0% 99.5% **PIEMHSIEPTVHSIIFNLIDNAIKYTPHQGVINISVYTDQDHYACIQIEDSGAGIDPENYDKVLKRFYRVHHHLEVGSGL**

6 SRR3222490 100.0% 99.5% **PIEMHSIEPTVHSIIFNLIDNAIKYTPHQGVINISVYTDQDHYACIQIEDSGAGIDPENYDKVLKRFYRVHHHLEVGSGL**

7 SRR3227013 100.0% 99.5% **PIEMHSIEPTVHSIIFNLIDNAIKYTPHQGVINISVYTDQDHYACIQIEDSGAGIDPENYDKVLKRFYRVHHHLEVGSGL**

8 SRR3228488 100.0% 99.5% **PIEMHSIEPTVHSIIFNLIDNAIKYTPHQGVINISVYTDQDHYACIQIEDSGAGIDPENYDKVLKRFYRVHHHLEVGSGL**

9 SRR3228565 100.0% 99.5% **PIEMHSIEPTVHSIIFNLIDNAIKYTPHQGVINISVYTDQDHYACIQIEDSGAGIDPENYDKVLKRFYRVHHHLEVGSGL**

10 A18 100.0% 99.5% **PIEMHSIEPTVHSIIFNLIDNAIKYTPHQGVINISVYTDQDHYACIQIEDSGAGIDPENYDKVLKRFYRVHHHLEVGSGL**

11 15A1042 100.0% 99.5% **PIEMHSIEPTVHSIIFNLIDNAIKYTPHQGVINISVYTDQDHYACIQIEDSGAGIDPENYDKVLKRFYRVHHHLEVGSGL**

12 MRSN7133 100.0% 99.5% **PIEMHSIEPTVHSIIFNLIDNAIKYTPHQGVINISVYTDQDHYACIQIEDSGAGIDPENYDKVLKRFYRVHHHLEVGSGL**

13 MRSN7130 100.0% 99.5% **PIEMHSIEPTVHSIIFNLIDNAIKYTPHQGVINISVYTDQDHYACIQIEDSGAGIDPENYDKVLKRFYRVHHHLEVGSGL**

14 MRSN7202 100.0% 99.5% **PIEMHSIEPTVHSIIFNLIDNAIKYTPHQGVINISVYTDQDHYACIQIEDSGAGIDPENYDKVLKRFYRVHHHLEVGSGL**

15 MRSN7224 100.0% 99.5% **PIEMHSIEPTVHSIIFNLIDNAIKYTPHQGVINISVYTDQDHYACIQIEDSGAGIDPENYDKVLKRFYRVHHHLEVGSGL**

16 OIFC074 100.0% 99.5% **PIEMHSIEPTVHSIIFNLIDNAIKYTPHQGVINISVYTDQDHYACIQIEDSGAGIDPENYDKVLKRFYRVHHHLEVGSGL**

cov pid **401**  **. . . . ]** **444**

1 ATCC19606 100.0% 100.0% **GLSIVDRATQRLGGTLTLDKSLELGGLSVLVKLPKVLHLNETRA**

2 M06 100.0% 98.9% **GLSIVDRATQRLGGTLTLDKSLELGGLSVLVKLPKVLHLHETRA**

3 M09 100.0% 99.5% **GLSIVDRATQRLGGTLTLDKSLELGGLSVLVKLPKVLHLHETRA**

4 M12 100.0% 99.5% **GLSIVDRATQRLGGTLTLDKSLELGGLSVLVKLPKVLHLHETRA**

5 M15 100.0% 99.5% **GLSIVDRATQRLGGTLTLDKSLELGGLSVLVKLPKVLHLHETRA**

6 SRR3222490 100.0% 99.5% **GLSIVDRATQRLGGTLTLDKSLELGGLSVLVKLPKVLHLHETRA**

7 SRR3227013 100.0% 99.5% **GLSIVDRATQRLGGTLTLDKSLELGGLSVLVKLPKVLHLHETRA**

8 SRR3228488 100.0% 99.5% **GLSIVDRATQRLGGTLTLDKSLELGGLSVLVKLPKVLHLHETRA**

9 SRR3228565 100.0% 99.5% **GLSIVDRATQRLGGTLTLDKSLELGGLSVLVKLPKVLHLHETRA**

10 A18 100.0% 99.5% **GLSIVDRATQRLGGTLTLDKSLELGGLSVLVKLPKVLHLHETRA**

11 15A1042 100.0% 99.5% **GLSIVDRATQRLGGTLTLDKSLELGGLSVLVKLPKVLHLHETRA**

12 MRSN7133 100.0% 99.5% **GLSIVDRATQRLGGTLTLDKSLELGGLSVLVKLPKVLHLHETRA**

13 MRSN7130 100.0% 99.5% **GLSIVDRATQRLGGTLTLDKSLELGGLSVLVKLPKVLHLHETRA**

14 MRSN7202 100.0% 99.5% **GLSIVDRATQRLGGTLTLDKSLELGGLSVLVKLPKVLHLHETRA**

15 MRSN7224 100.0% 99.5% **GLSIVDRATQRLGGTLTLDKSLELGGLSVLVKLPKVLHLHETRA**

16 OIFC074 100.0% 99.5% **GLSIVDRATQRLGGTLTLDKSLELGGLSVLVKLPKVLHLHETRA**

**Supplementary Figure 6: Multiple sequence alignment (MSA) of the predicted amino acid sequence of PmrB carried by ST19^Pas^ (GC1) isolates and close genomes retrieved from the BV-BRC database compared to the respective gene in *A. baumannii* ATCC19606.** MSA was created by the A multiple alignment viewer MView hosted by the EMBL-EBI; cov, coverage; pid, percent identity.

cov pid  **1** **[ . . . . : . . .** **80**

1 ATCC19606 100.0% 100.0% **MHYSLKKRLIWGTSIFSVILGCILIFSAYKVALQEVDEILDTQMKYLAERTAEHPLKTVSSKFDFHKTYHEEDLFIDIWA**

2 M01 100.0% 99.5% **MHYSLKKRLIWGTSIFSVILGCILIFSAYKVALQEVDEILDTQMKYLAERTAEHPLKTVSSKFDFHKTYHEEDLFIDIWA**

3 M04 100.0% 99.3% **MHYSLKKRLIWGTSIFSVILGCILIFSAYKVALQEVDEILDTQMKYLAERTAEHPLKTVSSKFDFHKTYHEEDLFIDIWA**

4 M05 77.9% 99.4% **MHYSLKKRLIWGTSIFSVILGCILIFSAYKVALQEVDEILDTQMKYLAERTAEHPLKTVSSKFDFHKTYHEEDLFIDIWA**

5 M13 100.0% 99.3% **MHYSLKKRLIWGTSIFSVILGCILIFSAYKVALQEVDEILDTQMKYLAERTAEHPLKTVSSKFDFHKTYHEEDLFIDIWA**

6 M16 100.0% 99.5% **MHYSLKKRLIWGTSIFSVILGCILIFSAYKVALQEVDEILDTQMKYLAERTAEHPLKTVSSKFDFHKTYHEEDLFIDIWA**

7 M17 100.0% 99.5% **MHYSLKKRLIWGTSIFSVILGCILIFSAYKVALQEVDEILDTQMKYLAERTAEHPLKTVSSKFDFHKTYHEEDLFIDIWA**

8 M20 100.0% 99.3% **MHYSLKKRLIWGTSIFSVILGCILIFSAYKVALQEVDEILDTQMKYLAERTAEHPLKTVSSKFDFHKTYHEEDLFIDIWA**

9 MS14413 100.0% 99.3% **MHYSLKKRLIWGTSIFSVILGCILIFSAYKVALQEVDEILDTQMKYLAERTAEHPLKTVSSKFDFHKTYHEEDLFIDIWA**

10 SQ093 100.0% 99.3% **MHYSLKKRLIWGTSIFSVILGCILIFSAYKVALQEVDEILDTQMKYLAERTAEHPLKTVSSKFDFHKTYHEEDLFIDIWA**

11 KAB3 100.0% 99.3% **MHYSLKKRLIWGTSIFSVILGCILIFSAYKVALQEVDEILDTQMKYLAERTAEHPLKTVSSKFDFHKTYHEEDLFIDIWA**

12 AC-40 75.7% 95.5% **MHYSLKKRLIWGTSIFSVILGCILIFSAYKVALQEVDEILDTQMKYLAERTAEHPLKTVSSKFDFHKTYHEEDLFIDIWA**

13 AC-14 89.9% 99.2% **MHYSLKKRLIWGTSIFSVILGCILIFSAYKVALQEVDEILDTQMKYLAERTAEHPLKTVSSKFDFHKTYHEEDLFIDIWA**

14 AC-45 89.9% 99.2% **MHYSLKKRLIWGTSIFSVILGCILIFSAYKVALQEVDEILDTQMKYLAERTAEHPLKTVSSKFDFHKTYHEEDLFIDIWA**

15 AC-23 89.9% 99.2% **MHYSLKKRLIWGTSIFSVILGCILIFSAYKVALQEVDEILDTQMKYLAERTAEHPLKTVSSKFDFHKTYHEEDLFIDIWA**

16 SUH-26-2 100.0% 99.5% **MHYSLKKRLIWGTSIFSVILGCILIFSAYKVALQEVDEILDTQMKYLAERTAEHPLKTVSSKFDFHKTYHEEDLFIDIWA**

17 SUH-11-2 100.0% 99.5% **MHYSLKKRLIWGTSIFSVILGCILIFSAYKVALQEVDEILDTQMKYLAERTAEHPLKTVSSKFDFHKTYHEEDLFIDIWA**

18 SUH-11-1 100.0% 99.5% **MHYSLKKRLIWGTSIFSVILGCILIFSAYKVALQEVDEILDTQMKYLAERTAEHPLKTVSSKFDFHKTYHEEDLFIDIWA**

19 A21 100.0% 99.3% **MHYSLKKRLIWGTSIFSVILGCILIFSAYKVALQEVDEILDTQMKYLAERTAEHPLKTVSSKFDFHKTYHEEDLFIDIWA**

20 SK044 100.0% 99.1% **MHYSLKKRLIWGTSIFSVILGCILIFSAYKVALQEVDEILDTQMKYLAERTAEHPLKTVSSKFDFHKTYHEEDLFIDIWA**

21 SK011 100.0% 99.1% **MHYSLKKRLIWGTSIFSVILGCILIFSAYKVALQEVDEILDTQMKYLAERTAEHPLKTVSSKFDFHKTYHEEDLFIDIWA**

22 SK002 100.0% 99.1% **MHYSLKKRLIWGTSIFSVILGCILIFSAYKVALQEVDEILDTQMKYLAERTAEHPLKTVSSKFDFHKTYHEEDLFIDIWA**

23 PSU120 100.0% 99.1% **MHYSLKKRLIWGTSIFSVILGCILIFSAYKVALQEVDEILDTQMKYLAERTAEHPLKTVSSKFDFHKTYHEEDLFIDIWA**

24 KUSSH35 100.0% 99.3% **MHYSLKKRLIWGTSIFSVILGCILIFSAYKVALQEVDEILDTQMKYLAERTAEHPLKTVSSKFDFHKTYHEEDLFIDIWA**

25 KUFAR56 100.0% 99.3% **MHYSLKKRLIWGTSIFSVILGCILIFSAYKVALQEVDEILDTQMKYLAERTAEHPLKTVSSKFDFHKTYHEEDLFIDIWA**

26 KUSSH15 100.0% 99.3% **MHYSLKKRLIWGTSIFSVILGCILIFSAYKVALQEVDEILDTQMKYLAERTAEHPLKTVSSKFDFHKTYHEEDLFIDIWA**

27 KUSSH37 100.0% 99.3% **MHYSLKKRLIWGTSIFSVILGCILIFSAYKVALQEVDEILDTQMKYLAERTAEHPLKTVSSKFDFHKTYHEEDLFIDIWA**

28 KUSSH36 100.0% 99.3% **MHYSLKKRLIWGTSIFSVILGCILIFSAYKVALQEVDEILDTQMKYLAERTAEHPLKTVSSKFDFHKTYHEEDLFIDIWA**

29 130 100.0% 99.3% **MHYSLKKRLIWGTSIFSVILGCILIFSAYKVALQEVDEILDTQMKYLAERTAEHPLKTVSSKFDFHKTYHEEDLFIDIWA**

30 Ab34 100.0% 99.3% **MHYSLKKRLIWGTSIFSVILGCILIFSAYKVALQEVDEILDTQMKYLAERTAEHPLKTVSSKFDFHKTYHEEDLFIDIWA**

31 Ab35 100.0% 99.3% **MHYSLKKRLIWGTSIFSVILGCILIFSAYKVALQEVDEILDTQMKYLAERTAEHPLKTVSSKFDFHKTYHEEDLFIDIWA**

32 Ab36 100.0% 99.3% **MHYSLKKRLIWGTSIFSVILGCILIFSAYKVALQEVDEILDTQMKYLAERTAEHPLKTVSSKFDFHKTYHEEDLFIDIWA**

33 Ab38 100.0% 99.3% **MHYSLKKRLIWGTSIFSVILGCILIFSAYKVALQEVDEILDTQMKYLAERTAEHPLKTVSSKFDFHKTYHEEDLFIDIWA**

34 Ab40 100.0% 99.3% **MHYSLKKRLIWGTSIFSVILGCILIFSAYKVALQEVDEILDTQMKYLAERTAEHPLKTVSSKFDFHKTYHEEDLFIDIWA**

35 Ab41 100.0% 99.3% **MHYSLKKRLIWGTSIFSVILGCILIFSAYKVALQEVDEILDTQMKYLAERTAEHPLKTVSSKFDFHKTYHEEDLFIDIWA**

36 Ab15 100.0% 99.3% **MHYSLKKRLIWGTSIFSVILGCILIFSAYKVALQEVDEILDTQMKYLAERTAEHPLKTVSSKFDFHKTYHEEDLFIDIWA**

37 Ab65 100.0% 99.3% **MHYSLKKRLIWGTSIFSVILGCILIFSAYKVALQEVDEILDTQMKYLAERTAEHPLKTVSSKFDFHKTYHEEDLFIDIWA**

38 Ab64 100.0% 99.3% **MHYSLKKRLIWGTSIFSVILGCILIFSAYKVALQEVDEILDTQMKYLAERTAEHPLKTVSSKFDFHKTYHEEDLFIDIWA**

39 AB22 100.0% 99.1% **MHYSLKKRLIWGTSIFSVILGCILIFSAYKVALQEVDEILDTQMKYLAERTAEHPLKTVSSKFDFHKTYHEEDLFIDIWA**

cov pid  **81**  **. 1 . . . . : .** **160**

1 ATCC19606 100.0% 100.0% **YKDQAHLSHHLHLLVPPVEQAGFYSHKTAQGIVRTYVLPLKDYQIQVSQQERVREAFAWELAGSMFIPYLIILPFAIFAL**

2 M01 100.0% 99.5% **YKDQAHLSHHLHLLVPPVEQAGFYSHKTAQGIVRTYVLPLKDYQIQVSQQERVREAFAWELAGSMFIPYLIILPFAIFAL**

3 M04 100.0% 99.3% **YKDQAHLSHHLHLLVPPVEQAGFYSHKTAQGIVRTYVLPLKDYQIQVSQQERVREAFTWELAGSMFIPYLIILPFAIFAL**

4 M05 77.9% 99.4% **YKDQAHLSHHLHLLVPPVEQAGFYSHKTAQGIVRTYVLPLKDYQIQVSQQERVREAFTWELAGSMFIPYLIILPFAIFAL**

5 M13 100.0% 99.3% **YKDQAHLSHHLHLLVPPVEQAGFYSHKTAQGIVRTYVLPLKDYQIQVSQQERVREAFTWELAGSMFIPYLIILPFAIFAL**

6 M16 100.0% 99.5% **YKDQAHLSHHLHLLVPPVEQAGFYSHKTAQGIVRTYVLPLKDYQIQVSQQERVREAFAWELAGSMFIPYLIILPFAIFAL**

7 M17 100.0% 99.5% **YKDQAHLSHHLHLLVPPVEQAGFYSHKTAQGIVRTYVLPLKDYQIQVSQQERVREAFAWELAGSMFIPYLIILPFAIFAL**

8 M20 100.0% 99.3% **YKDQAHLSHHLHLLVPPVEQAGFYSHKTAQGIVRTYVLPLKDYQIQVSQQERVREAFTWELAGSMFIPYLIILPFAIFAL**

9 MS14413 100.0% 99.3% **YKDQAHLSHHLHLLVPPVEQAGFYSHKTAQGIVRTYVLPLKDYQIQVSQQERVREAFAWELAGSMFIPYLIILPFAIFAL**

10 SQ093 100.0% 99.3% **YKDQAHLSHHLHLLVPPVEQAGFYSHKTAQGIVRTYVLPLKDYQIQVSQQERVREAFTWELAGSMFIPYLIILPFAIFAL**

11 KAB3 100.0% 99.3% **YKDQAHLSHHLHLLVPPVEQAGFYSHKTAQGIVRTYVLPLKDYQIQVSQQERVREAFTWELAGSMFIPYLIILPFAIFAL**

12 AC-40 75.7% 95.5% **YKDQAHLSHHLHLLVPPVEQAGFYSHKTAQGIVRTYVLPLKDYQIQVSQQERVREAFTWELAGSMFIPYLIILPFAIFAL**

13 AC-14 89.9% 99.2% **YKDQAHLSHHLHLLVPPVEQAGFYSHKTAQGIVRTYVLPLKDYQIQVSQQERVREAFTWELAGSMFIPYLIILPFAIFAL**

14 AC-45 89.9% 99.2% **YKDQAHLSHHLHLLVPPVEQAGFYSHKTAQGIVRTYVLPLKDYQIQVSQQERVREAFTWELAGSMFIPYLIILPFAIFAL**

15 AC-23 89.9% 99.2% **YKDQAHLSHHLHLLVPPVEQAGFYSHKTAQGIVRTYVLPLKDYQIQVSQQERVREAFTWELAGSMFIPYLIILPFAIFAL**

16 SUH-26-2 100.0% 99.5% **YKDQAHLSHHLHLLVPPVEQAGFYSHKTAQGIVRTYVLPLKDYQIQVSQQERVREAFAWELAGSMFIPYLIILPFAIFAL**

17 SUH-11-2 100.0% 99.5% **YKDQAHLSHHLHLLVPPVEQAGFYSHKTAQGIVRTYVLPLKDYQIQVSQQERVREAFAWELAGSMFIPYLIILPFAIFAL**

18 SUH-11-1 100.0% 99.5% **YKDQAHLSHHLHLLVPPVEQAGFYSHKTAQGIVRTYVLPLKDYQIQVSQQERVREAFAWELAGSMFIPYLIILPFAIFAL**

19 A21 100.0% 99.3% **YKDQAHLSHHLHLLVPPVEQAGFYSHKTAQGIVRTYVLPLKDYQIQVSQQERVREAFTWELAGSMFIPYLIILPFAIFAL**

20 SK044 100.0% 99.1% **YKDQAHLSHHLHLLVPPVEQAGFYSHKTAQGIVRTYVLPLKDYQIQVSQQERVREAFTWELAGSMFIPYLIILPFAIFAL**

21 SK011 100.0% 99.1% **YKDQAHLSHHLHLLVPPVEQAGFYSHKTAQGIVRTYVLPLKDYQIQVSQQERVREAFTWELAGSMFIPYLIILPFAIFAL**

22 SK002 100.0% 99.1% **YKDQAHLSHHLHLLVPPVEQAGFYSHKTAQGIVRTYVLPLKDYQIQVSQQERVREAFTWELAGSMFIPYLIILPFAIFAL**

23 PSU120 100.0% 99.1% **YKDQAHLSHHLHLLVPPVEQAGFYSHKTAQGIVRTYVLPLKDYQIQVSQQERVREAFTWELAGSMFIPYLIILPFAIFAL**

24 KUSSH35 100.0% 99.3% **YKDQAHLSHHLHLLVPPVEQAGFYSHKTAQGIVRTYVLPLKDYQIQVSQQERVREAFTWELAGSMFIPYLIILPFAIFAL**

25 KUFAR56 100.0% 99.3% **YKDQAHLSHHLHLLVPPVEQAGFYSHKTAQGIVRTYVLPLKDYQIQVSQQERVREAFTWELAGSMFIPYLIILPFAIFAL**

26 KUSSH15 100.0% 99.3% **YKDQAHLSHHLHLLVPPVEQAGFYSHKTAQGIVRTYVLPLKDYQIQVSQQERVREAFTWELAGSMFIPYLIILPFAIFAL**

27 KUSSH37 100.0% 99.3% **YKDQAHLSHHLHLLVPPVEQAGFYSHKTAQGIVRTYVLPLKDYQIQVSQQERVREAFTWELAGSMFIPYLIILPFAIFAL**

28 KUSSH36 100.0% 99.3% **YKDQAHLSHHLHLLVPPVEQAGFYSHKTAQGIVRTYVLPLKDYQIQVSQQERVREAFTWELAGSMFIPYLIILPFAIFAL**

29 130 100.0% 99.3% **YKDQAHLSHHLHLLVPPVEQAGFYSHKTAQGIVRTYVLPLKDYQIQVSQQERVREAFTWELAGSMFIPYLIILPFAIFAL**

30 Ab34 100.0% 99.3% **YKDQAHLSHHLHLLVPPVEQAGFYSHKTAQGIVRTYVLPLKDYQIQVSQQERVREAFTWELAGSMFIPYLIILPFAIFAL**

31 Ab35 100.0% 99.3% **YKDQAHLSHHLHLLVPPVEQAGFYSHKTAQGIVRTYVLPLKDYQIQVSQQERVREAFTWELAGSMFIPYLIILPFAIFAL**

32 Ab36 100.0% 99.3% **YKDQAHLSHHLHLLVPPVEQAGFYSHKTAQGIVRTYVLPLKDYQIQVSQQERVREAFTWELAGSMFIPYLIILPFAIFAL**

33 Ab38 100.0% 99.3% **YKDQAHLSHHLHLLVPPVEQAGFYSHKTAQGIVRTYVLPLKDYQIQVSQQERVREAFTWELAGSMFIPYLIILPFAIFAL**

34 Ab40 100.0% 99.3% **YKDQAHLSHHLHLLVPPVEQAGFYSHKTAQGIVRTYVLPLKDYQIQVSQQERVREAFTWELAGSMFIPYLIILPFAIFAL**

35 Ab41 100.0% 99.3% **YKDQAHLSHHLHLLVPPVEQAGFYSHKTAQGIVRTYVLPLKDYQIQVSQQERVREAFTWELAGSMFIPYLIILPFAIFAL**

36 Ab15 100.0% 99.3% **YKDQAHLSHHLHLLVPPVEQAGFYSHKTAQGIVRTYVLPLKDYQIQVSQQERVREAFTWELAGSMFIPYLIILPFAIFAL**

37 Ab65 100.0% 99.3% **YKDQAHLSHHLHLLVPPVEQAGFYSHKTAQGIVRTYVLPLKDYQIQVSQQERVREAFTWELAGSMFIPYLIILPFAIFAL**

38 Ab64 100.0% 99.3% **YKDQAHLSHHLHLLVPPVEQAGFYSHKTAQGIVRTYVLPLKDYQIQVSQQERVREAFTWELAGSMFIPYLIILPFAIFAL**

39 AB22 100.0% 99.1% **YKDQAHLSHHLHLLVPPVEQAGFYSHKTAQGIVRTYVLPLKDYQIQVSQQERVREAFTWELAGSMFIPYLIILPFAIFAL**

cov pid **161**  **. . . 2 . . . .** **240**

1 ATCC19606 100.0% 100.0% **AAIIRRGLKPIDDFKNELKERDSEELTPIEVHDYPQELLPTIDEMNRLFERISKAQNEQKQFIADAAHELRTPVTALNLQ**

2 M01 100.0% 99.5% **AAIIRRGLKPIDDFKNELKERDSEELTPIEVHDYPQELLPTIDEMNRLFERISKAQNEQKQFIADAAHELRTPVTALNLQ**

3 M04 100.0% 99.3% **AAIIRRGLKPIDDFKNELKERDSEELTPIEVHDYPQELLPTIDEMNRLFERISKAQNEQKQFIADAAHELRTPVTALNLQ**

4 M05 77.9% 99.4% **AAIIRRGLKPIDDFKNELKERDSEELTPIEVHDYPQELLPTIDEMNRLFERISKAQNEQKQFIADAAHELRTPVTALNLQ**

5 M13 100.0% 99.3% **AAIIRRGLKPIDDFKNELKERDSEELTPIEVHDYPQELLPTIDEMNRLFERISKAQNEQKQFIADAAHELRTPVTALNLQ**

6 M16 100.0% 99.5% **AAIIRRGLKPIDDFKNELKERDSEELTPIEVHDYPQELLPTIDEMNRLFERISKAQNEQKQFIADAAHELRTPVTALNLQ**

7 M17 100.0% 99.5% **AAIIRRGLKPIDDFKNELKERDSEELTPIEVHDYPQELLPTIDEMNRLFERISKAQNEQKQFIADAAHELRTPVTALNLQ**

8 M20 100.0% 99.3% **AAIIRRGLKPIDDFKNELKERDSEELTPIEVHDYPQELLPTIDEMNRLFERISKAQNEQKQFIADAAHELRTPVTALNLQ**

9 MS14413 100.0% 99.3% **AAIIRRGLKPIDDFKNELKERDSEELTPIEVHDYPQELLPTIDEMNRLFERISKAQNEQKQFIADAAHELRIPVTALNLQ**

10 SQ093 100.0% 99.3% **AAIIRRGLKPIDDFKNELKERDSEELTPIEVHDYPQELLPTIDEMNRLFERISKAQNEQKQFIADAAHELRTPVTALNLQ**

11 KAB3 100.0% 99.3% **AAIIRRGLKPIDDFKNELKERDSEELTPIEVHDYPQELLPTIDEMNRLFERISKAQNEQKQFIADAAHELRTPVTALNLQ**

12 AC-40 75.7% 95.5% **AAIIRRGLKPIDDFKNELKERDSEELTPIEVHDYPQELLPTIDEMNRLFERISKAQNEQKQFIADAAHELRTSVTALNLQ**

13 AC-14 89.9% 99.2% **AAIIRRGLKPIDDFKNELKERDSEELTPIEVHDYPQELLPTIDEMNRLFERISKAQNEQKQFIADAAHELRTSVTALNLQ**

14 AC-45 89.9% 99.2% **AAIIRRGLKPIDDFKNELKERDSEELTPIEVHDYPQELLPTIDEMNRLFERISKAQNEQKQFIADAAHELRTSVTALNLQ**

15 AC-23 89.9% 99.2% **AAIIRRGLKPIDDFKNELKERDSEELTPIEVHDYPQELLPTIDEMNRLFERISKAQNEQKQFIADAAHELRTSVTALNLQ**

16 SUH-26-2 100.0% 99.5% **AAIIRRGLKPIDDFKNELKERDSEELTPIEVHDYPQELLPTIDEMNRLFERISKAQNEQKQFIADAAHELRTPVTALNLQ**

17 SUH-11-2 100.0% 99.5% **AAIIRRGLKPIDDFKNELKERDSEELTPIEVHDYPQELLPTIDEMNRLFERISKAQNEQKQFIADAAHELRTPVTALNLQ**

18 SUH-11-1 100.0% 99.5% **AAIIRRGLKPIDDFKNELKERDSEELTPIEVHDYPQELLPTIDEMNRLFERISKAQNEQKQFIADAAHELRTPVTALNLQ**

19 A21 100.0% 99.3% **AAIIRRGLKPIDDFKNELKERDSEELTPIEVHDYPQELLPTIDEMNRLFERISKAQNEQKQFIADAAHELRTPVTALNLQ**

20 SK044 100.0% 99.1% **AAIIRRGLKPIDDFKNELKERDSEELTPIEVHDYPQELLPTIDEMNRLFERISKAQNEQKQFIADVAHELRTPVTALNLQ**

21 SK011 100.0% 99.1% **AAIIRRGLKPIDDFKNELKERDSEELTPIEVHDYPQELLPTIDEMNRLFERISKAQNEQKQFIADVAHELRTPVTALNLQ**

22 SK002 100.0% 99.1% **AAIIRRGLKPIDDFKNELKERDSEELTPIEVHDYPQELLPTIDEMNRLFERISKAQNEQKQFIADVAHELRTPVTALNLQ**

23 PSU120 100.0% 99.1% **AAIIRRGLKPIDDFKNELKERDSEELTPIEVHDYPQELLPTIDEMNRLFERISKAQNEQKQFIADVAHELRTPVTALNLQ**

24 KUSSH35 100.0% 99.3% **AAIIRRGLKPIDDFKNELKERDSEELTPIEVHDYPQELLPTIDEMNRLFERISKAQNEQKQFIADAAHELRTPVTALNLQ**

25 KUFAR56 100.0% 99.3% **AAIIRRGLKPIDDFKNELKERDSEELTPIEVHDYPQELLPTIDEMNRLFERISKAQNEQKQFIADAAHELRTPVTALNLQ**

26 KUSSH15 100.0% 99.3% **AAIIRRGLKPIDDFKNELKERDSEELTPIEVHDYPQELLPTIDEMNRLFERISKAQNEQKQFIADAAHELRTPVTALNLQ**

27 KUSSH37 100.0% 99.3% **AAIIRRGLKPIDDFKNELKERDSEELTPIEVHDYPQELLPTIDEMNRLFERISKAQNEQKQFIADAAHELRTPVTALNLQ**

28 KUSSH36 100.0% 99.3% **AAIIRRGLKPIDDFKNELKERDSEELTPIEVHDYPQELLPTIDEMNRLFERISKAQNEQKQFIADAAHELRTPVTALNLQ**

29 130 100.0% 99.3% **AAIIRRGLKPIDDFKNELKERDSEELTPIEVHDYPQELLPTIDEMNRLFERISKAQNEQKQFIADAAHELRTPVTALNLQ**

30 Ab34 100.0% 99.3% **AAIIRRGLKPIDDFKNELKERDSEELTPIEVHDYPQELLPTIDEMNRLFERISKAQNEQKQFIADAAHELRTPVTALNLQ**

31 Ab35 100.0% 99.3% **AAIIRRGLKPIDDFKNELKERDSEELTPIEVHDYPQELLPTIDEMNRLFERISKAQNEQKQFIADAAHELRTPVTALNLQ**

32 Ab36 100.0% 99.3% **AAIIRRGLKPIDDFKNELKERDSEELTPIEVHDYPQELLPTIDEMNRLFERISKAQNEQKQFIADAAHELRTPVTALNLQ**

33 Ab38 100.0% 99.3% **AAIIRRGLKPIDDFKNELKERDSEELTPIEVHDYPQELLPTIDEMNRLFERISKAQNEQKQFIADAAHELRTPVTALNLQ**

34 Ab40 100.0% 99.3% **AAIIRRGLKPIDDFKNELKERDSEELTPIEVHDYPQELLPTIDEMNRLFERISKAQNEQKQFIADAAHELRTPVTALNLQ**

35 Ab41 100.0% 99.3% **AAIIRRGLKPIDDFKNELKERDSEELTPIEVHDYPQELLPTIDEMNRLFERISKAQNEQKQFIADAAHELRTPVTALNLQ**

36 Ab15 100.0% 99.3% **AAIIRRGLKPIDDFKNELKERDSEELTPIEVHDYPQELLPTIDEMNRLFERISKAQNEQKQFIADAAHELRTPVTALNLQ**

37 Ab65 100.0% 99.3% **AAIIRRGLKPIDDFKNELKERDSEELTPIEVHDYPQELLPTIDEMNRLFERISKAQNEQKQFIADAAHELRTPVTALNLQ**

38 Ab64 100.0% 99.3% **AAIIRRGLKPIDDFKNELKERDSEELTPIEVHDYPQELLPTIDEMNRLFERISKAQNEQKQFIADAAHELRTPVTALNLQ**

39 AB22 100.0% 99.1% **AAIIRRGLKPIDDFKNELKERDSEELTPIEVHDYPQELLPTIDEMNRLFERISKAQNEQKQFIADVAHELRTPVTALNLQ**

cov pid **241**  **: . . . . 3 . .** **320**

1 ATCC19606 100.0% 100.0% **TKILLSQFPEHESLQNLSKGLARIQHLVTQLLALAKQDVTLSMVEPTGYFQLNDVALNCVEQLVNLAMQKEIDLGFVRNE**

2 M01 100.0% 99.5% **TKILLSQFPEHESLQNLSKGLARIQHLVTQLLALAKQDVTLSMVEPTGYFQLNDVALNCVEQLVNLAMQKEIDLGFVRNE**

3 M04 100.0% 99.3% **TKILLSQFPEHESLQNLSKGLARIQHLVTQLLALAKQDVTLSMVEPTGYFQLNDVALNCVEQLVNLAMQKEIDLGFVRNE**

4 M05 77.9% 99.4% **TKILLSQFPEHESLQNLSKGLARIQHLVTQLLALAKQDVTLSMVEPTGYFQLNDVALNCVEQLVNLAMQKEIDLGFVRNE**

5 M13 100.0% 99.3% **TKILLSQFPEHESLQNLSKGLARIQHLVTQLLALAKQDVTLSMVEPTGYFQLNDVALNCVEQLVNLAMQKEIDLGFVRNE**

6 M16 100.0% 99.5% **TKILLSQFPEHESLQNLSKGLARIQHLVTQLLALAKQDVTLSMVEPTGYFQLNDVALNCVEQLVNLAMQKEIDLGFVRNE**

7 M17 100.0% 99.5% **TKILLSQFPEHESLQNLSKGLARIQHLVTQLLALAKQDVTLSMVEPTGYFQLNDVALNCVEQLVNLAMQKEIDLGFVRNE**

8 M20 100.0% 99.3% **TKILLSQFPEHESLQNLSKGLARIQHLVTQLLALAKQDVTLSMVEPTGYFQLNDVALNCVEQLVNLAMQKEIDLGFVRNE**

9 MS14413 100.0% 99.3% **TKILLSQFPEHESLQNLSKGLARIQHLVTQLLALAKQDVTLSMVEPTGYFQLNDVALNCVEQLVNLAMQKEIDLGFVRNE**

10 SQ093 100.0% 99.3% **TKILLSQFPEHESLQNLSKGLARIQHLVTQLLALAKQDVTLSMVEPTGYFQLNDVALNCVEQLVNLAMQKEIDLGFVRNE**

11 KAB3 100.0% 99.3% **TKILLSQFPEHESLQNLSKGLARIQHLVTQLLALAKQDVTLSMVEPTGYFQLNDVALNCVEQLVNLAMQKEIDLGFVRNE**

12 AC-40 75.7% 95.5% **TKILLSQFPEHESLQNLSKGLARIQHLVTQLLALAKQDVTLSMVEPTGYFQLNDVALNCVEQLVNLAMQKEIDLGFVRNE**

13 AC-14 89.9% 99.2% **TKILLSQFPEHESLQNLSKGLARIQHLVTQLLALAKQDVTLSMVEPTGYFQLNDVALNCVEQLVNLAMQKEIDLGFVRNE**

14 AC-45 89.9% 99.2% **TKILLSQFPEHESLQNLSKGLARIQHLVTQLLALAKQDVTLSMVEPTGYFQLNDVALNCVEQLVNLAMQKEIDLGFVRNE**

15 AC-23 89.9% 99.2% **TKILLSQFPEHESLQNLSKGLARIQHLVTQLLALAKQDVTLSMVEPTGYFQLNDVALNCVEQLVNLAMQKEIDLGFVRNE**

16 SUH-26-2 100.0% 99.5% **TKILLSQFPEHESLQNLSKGLARIQHLVTQLLALAKQDVTLSMVEPTGYFQLNDVALNCVEQLVNLAMQKEIDLGFVRNE**

17 SUH-11-2 100.0% 99.5% **TKILLSQFPEHESLQNLSKGLARIQHLVTQLLALAKQDVTLSMVEPTGYFQLNDVALNCVEQLVNLAMQKEIDLGFVRNE**

18 SUH-11-1 100.0% 99.5% **TKILLSQFPEHESLQNLSKGLARIQHLVTQLLALAKQDVTLSMVEPTGYFQLNDVALNCVEQLVNLAMQKEIDLGFVRNE**

19 A21 100.0% 99.3% **TKILLSQFPEHESLQNLSKGLARIQHLVTQLLALAKQDVTLSMVEPTGYFQLNDVALNCVEQLVNLAMQKEIDLGFVRNE**

20 SK044 100.0% 99.1% **TKILLSQFPEHESLQNLSKGLARIQHLVTQLLALAKQDVTLSMVEPTGYFQLNDVALNCVEQLVNLAMQKEIDLGFVRNE**

21 SK011 100.0% 99.1% **TKILLSQFPEHESLQNLSKGLARIQHLVTQLLALAKQDVTLSMVEPTGYFQLNDVALNCVEQLVNLAMQKEIDLGFVRNE**

22 SK002 100.0% 99.1% **TKILLSQFPEHESLQNLSKGLARIQHLVTQLLALAKQDVTLSMVEPTGYFQLNDVALNCVEQLVNLAMQKEIDLGFVRNE**

23 PSU120 100.0% 99.1% **TKILLSQFPEHESLQNLSKGLARIQHLVTQLLALAKQDVTLSMVEPTGYFQLNDVALNCVEQLVNLAMQKEIDLGFVRNE**

24 KUSSH35 100.0% 99.3% **TKILLSQFPEHESLQNLSKGLARIQHLVTQLLALAKQDVTLSMVEPTGYFQLNDVALNCVEQLVNLAMQKEIDLGFVRNE**

25 KUFAR56 100.0% 99.3% **TKILLSQFPEHESLQNLSKGLARIQHLVTQLLALAKQDVTLSMVEPTGYFQLNDVALNCVEQLVNLAMQKEIDLGFVRNE**

26 KUSSH15 100.0% 99.3% **TKILLSQFPEHESLQNLSKGLARIQHLVTQLLALAKQDVTLSMVEPTGYFQLNDVALNCVEQLVNLAMQKEIDLGFVRNE**

27 KUSSH37 100.0% 99.3% **TKILLSQFPEHESLQNLSKGLARIQHLVTQLLALAKQDVTLSMVEPTGYFQLNDVALNCVEQLVNLAMQKEIDLGFVRNE**

28 KUSSH36 100.0% 99.3% **TKILLSQFPEHESLQNLSKGLARIQHLVTQLLALAKQDVTLSMVEPTGYFQLNDVALNCVEQLVNLAMQKEIDLGFVRNE**

29 130 100.0% 99.3% **TKILLSQFPEHESLQNLSKGLARIQHLVTQLLALAKQDVTLSMVEPTGYFQLNDVALNCVEQLVNLAMQKEIDLGFVRNE**

30 Ab34 100.0% 99.3% **TKILLSQFPEHESLQNLSKGLARIQHLVTQLLALAKQDVTLSMVEPTGYFQLNDVALNCVEQLVNLAMQKEIDLGFVRNE**

31 Ab35 100.0% 99.3% **TKILLSQFPEHESLQNLSKGLARIQHLVTQLLALAKQDVTLSMVEPTGYFQLNDVALNCVEQLVNLAMQKEIDLGFVRNE**

32 Ab36 100.0% 99.3% **TKILLSQFPEHESLQNLSKGLARIQHLVTQLLALAKQDVTLSMVEPTGYFQLNDVALNCVEQLVNLAMQKEIDLGFVRNE**

33 Ab38 100.0% 99.3% **TKILLSQFPEHESLQNLSKGLARIQHLVTQLLALAKQDVTLSMVEPTGYFQLNDVALNCVEQLVNLAMQKEIDLGFVRNE**

34 Ab40 100.0% 99.3% **TKILLSQFPEHESLQNLSKGLARIQHLVTQLLALAKQDVTLSMVEPTGYFQLNDVALNCVEQLVNLAMQKEIDLGFVRNE**

35 Ab41 100.0% 99.3% **TKILLSQFPEHESLQNLSKGLARIQHLVTQLLALAKQDVTLSMVEPTGYFQLNDVALNCVEQLVNLAMQKEIDLGFVRNE**

36 Ab15 100.0% 99.3% **TKILLSQFPEHESLQNLSKGLARIQHLVTQLLALAKQDVTLSMVEPTGYFQLNDVALNCVEQLVNLAMQKEIDLGFVRNE**

37 Ab65 100.0% 99.3% **TKILLSQFPEHESLQNLSKGLARIQHLVTQLLALAKQDVTLSMVEPTGYFQLNDVALNCVEQLVNLAMQKEIDLGFVRNE**

38 Ab64 100.0% 99.3% **TKILLSQFPEHESLQNLSKGLARIQHLVTQLLALAKQDVTLSMVEPTGYFQLNDVALNCVEQLVNLAMQKEIDLGFVRNE**

39 AB22 100.0% 99.1% **TKILLSQFPEHESLQNLSKGLARIQHLVTQLLALAKQDVTLSMVEPTGYFQLNDVALNCVEQLVNLAMQKEIDLGFVRNE**

cov pid **321**  **. . : . . . . 4** **400**

1 ATCC19606 100.0% 100.0% **PIEMHSIEPTVHSIIFNLIDNAIKYTPHQGVINISVYTDPDHYACIQIEDSGAGIDPENYDKVLKRFYRVHHHLEVGSGL**

2 M01 100.0% 99.5% **PIEMHSIEPTVHSIIFNLIDNAIKYTPHQGVINISVYTDPDHYACIQIEDSGAGIDPENYDKVLKRFYRVHHHLEVGSGL**

3 M04 100.0% 99.3% **PIEMHSIEPTVHSIIFNLIDNAIKYTPHQGVINISVYTDPDHYACIQIEDSGAGIDPENYDKVLKRFYRVHHHLEVGSGL**

4 M05 77.9% 99.4% **PIEMHSIEPTVHSIIFNLIDNAIKY-------------------------------------------------------**

5 M13 100.0% 99.3% **PIEMHSIEPTVHSIIFNLIDNAIKYTPHQGVINISVYTDPDHYACIQIEDSGAGIDPENYDKVLKRFYRVHHHLEVGSGL**

6 M16 100.0% 99.5% **PIEMHSIEPTVHSIIFNLIDNAIKYTPHQGVINISVYTDPDHYACIQIEDSGAGIDPENYDKVLKRFYRVHHHLEVGSGL**

7 M17 100.0% 99.5% **PIEMHSIEPTVHSIIFNLIDNAIKYTPHQGVINISVYTDPDHYACIQIEDSGAGIDPENYDKVLKRFYRVHHHLEVGSGL**

8 M20 100.0% 99.3% **PIEMHSIEPTVHSIIFNLIDNAIKYTPHQGVINISVYTDPDHYACIQIEDSGAGIDPENYDKVLKRFYRVHHHLEVGSGL**

9 MS14413 100.0% 99.3% **PIEMHSIEPTVHSIIFNLIDNAIKYTPHQGVINISVYTDPDHYACIQIEDSGAGIDPENYDKVLKRFYRVHHHLEVGSGL**

10 SQ093 100.0% 99.3% **PIEMHSIEPTVHSIIFNLIDNAIKYTPHQGVINISVYTDPDHYACIQIEDSGAGIDPENYDKVLKRFYRVHHHLEVGSGL**

11 KAB3 100.0% 99.3% **PIEMHSIEPTVHSIIFNLIDNAIKYTPHQGVINISVYTDPDHYACIQIEDSGAGIDPENYDKVLKRFYRVHHHLEVGSGL**

12 AC-40 75.7% 95.5% **PIEIVLNLLYIRLFLI----------------------------------------------------------------**

13 AC-14 89.9% 99.2% **PIEMHSIEPTVHSIIFNLIDNAIKYTPHQGVINISVYTDPDHYACIQIEDSGAGIDPENYDKVLKRFYRVHHHLEVGSV-**

14 AC-45 89.9% 99.2% **PIEMHSIEPTVHSIIFNLIDNAIKYTPHQGVINISVYTDPDHYACIQIEDSGAGIDPENYDKVLKRFYRVHHHLEVGSV-**

15 AC-23 89.9% 99.2% **PIEMHSIEPTVHSIIFNLIDNAIKYTPHQGVINISVYTDPDHYACIQIEDSGAGIDPENYDKVLKRFYRVHHHLEVGSV-**

16 SUH-26-2 100.0% 99.5% **PIEMHSIEPTVHSIIFNLIDNAIKYTPHQGVINISVYTDPDHYACIQIEDSGAGIDPENYDKVLKRFYRVHHHLEVGSGL**

17 SUH-11-2 100.0% 99.5% **PIEMHSIEPTVHSIIFNLIDNAIKYTPHQGVINISVYTDPDHYACIQIEDSGAGIDPENYDKVLKRFYRVHHHLEVGSGL**

18 SUH-11-1 100.0% 99.5% **PIEMHSIEPTVHSIIFNLIDNAIKYTPHQGVINISVYTDPDHYACIQIEDSGAGIDPENYDKVLKRFYRVHHHLEVGSGL**

19 A21 100.0% 99.3% **PIEMHSIEPTVHSIIFNLIDNAIKYTPHQGVINISVYTDPDHYACIQIEDSGAGIDPENYDKVLKRFYRVHHHLEVGSGL**

20 SK044 100.0% 99.1% **PIEMHSIEPTVHSIIFNLIDNAIKYTPHQGVINISVYTDPDHYACIQIEDSGAGIDPENYDKVLKRFYRVHHHLEVGSGL**

21 SK011 100.0% 99.1% **PIEMHSIEPTVHSIIFNLIDNAIKYTPHQGVINISVYTDPDHYACIQIEDSGAGIDPENYDKVLKRFYRVHHHLEVGSGL**

22 SK002 100.0% 99.1% **PIEMHSIEPTVHSIIFNLIDNAIKYTPHQGVINISVYTDPDHYACIQIEDSGAGIDPENYDKVLKRFYRVHHHLEVGSGL**

23 PSU120 100.0% 99.1% **PIEMHSIEPTVHSIIFNLIDNAIKYTPHQGVINISVYTDPDHYACIQIEDSGAGIDPENYDKVLKRFYRVHHHLEVGSGL**

24 KUSSH35 100.0% 99.3% **PIEMHSIEPTVHSIIFNLIDNAIKYTPHQGVINISVYTDPDHYACIQIEDSGAGIDPENYDKVLKRFYRVHHHLEVGSGL**

25 KUFAR56 100.0% 99.3% **PIEMHSIEPTVHSIIFNLIDNAIKYTPHQGVINISVYTDPDHYACIQIEDSGAGIDPENYDKVLKRFYRVHHHLEVGSGL**

26 KUSSH15 100.0% 99.3% **PIEMHSIEPTVHSIIFNLIDNAIKYTPHQGVINISVYTDPDHYACIQIEDSGAGIDPENYDKVLKRFYRVHHHLEVGSGL**

27 KUSSH37 100.0% 99.3% **PIEMHSIEPTVHSIIFNLIDNAIKYTPHQGVINISVYTDPDHYACIQIEDSGAGIDPENYDKVLKRFYRVHHHLEVGSGL**

28 KUSSH36 100.0% 99.3% **PIEMHSIEPTVHSIIFNLIDNAIKYTPHQGVINISVYTDPDHYACIQIEDSGAGIDPENYDKVLKRFYRVHHHLEVGSGL**

29 130 100.0% 99.3% **PIEMHSIEPTVHSIIFNLIDNAIKYTPHQGVINISVYTDPDHYACIQIEDSGAGIDPENYDKVLKRFYRVHHHLEVGSGL**

30 Ab34 100.0% 99.3% **PIEMHSIEPTVHSIIFNLIDNAIKYTPHQGVINISVYTDPDHYACIQIEDSGAGIDPENYDKVLKRFYRVHHHLEVGSGL**

31 Ab35 100.0% 99.3% **PIEMHSIEPTVHSIIFNLIDNAIKYTPHQGVINISVYTDPDHYACIQIEDSGAGIDPENYDKVLKRFYRVHHHLEVGSGL**

32 Ab36 100.0% 99.3% **PIEMHSIEPTVHSIIFNLIDNAIKYTPHQGVINISVYTDPDHYACIQIEDSGAGIDPENYDKVLKRFYRVHHHLEVGSGL**

33 Ab38 100.0% 99.3% **PIEMHSIEPTVHSIIFNLIDNAIKYTPHQGVINISVYTDPDHYACIQIEDSGAGIDPENYDKVLKRFYRVHHHLEVGSGL**

34 Ab40 100.0% 99.3% **PIEMHSIEPTVHSIIFNLIDNAIKYTPHQGVINISVYTDPDHYACIQIEDSGAGIDPENYDKVLKRFYRVHHHLEVGSGL**

35 Ab41 100.0% 99.3% **PIEMHSIEPTVHSIIFNLIDNAIKYTPHQGVINISVYTDPDHYACIQIEDSGAGIDPENYDKVLKRFYRVHHHLEVGSGL**

36 Ab15 100.0% 99.3% **PIEMHSIEPTVHSIIFNLIDNAIKYTPHQGVINISVYTDPDHYACIQIEDSGAGIDPENYDKVLKRFYRVHHHLEVGSGL**

37 Ab65 100.0% 99.3% **PIEMHSIEPTVHSIIFNLIDNAIKYTPHQGVINISVYTDPDHYACIQIEDSGAGIDPENYDKVLKRFYRVHHHLEVGSGL**

38 Ab64 100.0% 99.3% **PIEMHSIEPTVHSIIFNLIDNAIKYTPHQGVINISVYTDPDHYACIQIEDSGAGIDPENYDKVLKRFYRVHHHLEVGSGL**

39 AB22 100.0% 99.1% **PIEMHSIEPTVHSIIFNLIDNAIKYTPHQGVINISVYTDPDHYACIQIEDSGAGIDPENYDKVLKRFYRVHHHLEVGSGL**

cov pid **401**  **. . . . ]** **444**

1 ATCC19606 100.0% 100.0% **GLSIVDRATQRLGGTLTLDKSLELGGLSVLVKLPKVLHLNETRA**

2 M01 100.0% 99.5% **GLSIVDRATQRLGGTLTLDKSLELGGLSVLVKLPKVLHLHETRV**

3 M04 100.0% 99.3% **GLSIVDRATQRLGGTLTLDKSLELGGLSVLVKLPKVLHLHETRV**

4 M05 77.9% 99.4% **--------------------------------------------**

5 M13 100.0% 99.3% **GLSIVDRATQRLGGTLTLDKSLELGGLSVLVKLPKVLHLHETRV**

6 M16 100.0% 99.5% **GLSIVDRATQRLGGTLTLDKSLELGGLSVLVKLPKVLHLHETRV**

7 M17 100.0% 99.5% **GLSIVDRATQRLGGTLTLDKSLELGGLSVLVKLPKVLHLHETRV**

8 M20 100.0% 99.3% **GLSIVDRATQRLGGTLTLDKSLELGGLSVLVKLPKVLHLHETRV**

9 MS14413 100.0% 99.3% **GLSIVDRATQRLGGTLTLDKSLELGGLSVLVKLPKVLHLHETRV**

10 SQ093 100.0% 99.3% **GLSIVDRATQRLGGTLTLDKSLELGGLSVLVKLPKVLHLHETRV**

11 KAB3 100.0% 99.3% **GLSIVDRATQRLGGTLTLDKSLELGGLSVLVKLPKVLHLHETRV**

12 AC-40 75.7% 95.5% **--------------------------------------------**

13 AC-14 89.9% 99.2% **--------------------------------------------**

14 AC-45 89.9% 99.2% **--------------------------------------------**

15 AC-23 89.9% 99.2% **--------------------------------------------**

16 SUH-26-2 100.0% 99.5% **GLSIVDRATQRLGGTLTLDKSLELGGLSVLVKLPKVLHLHETRV**

17 SUH-11-2 100.0% 99.5% **GLSIVDRATQRLGGTLTLDKSLELGGLSVLVKLPKVLHLHETRV**

18 SUH-11-1 100.0% 99.5% **GLSIVDRATQRLGGTLTLDKSLELGGLSVLVKLPKVLHLHETRV**

19 A21 100.0% 99.3% **GLSIVDRATQRLGGTLTLDKSLELGGLSVLVKLPKVLHLHETRV**

20 SK044 100.0% 99.1% **GLSIVDRATQRLGGTLTLDKSLELGGLSVLVKLPKVLHLHETRV**

21 SK011 100.0% 99.1% **GLSIVDRATQRLGGTLTLDKSLELGGLSVLVKLPKVLHLHETRV**

22 SK002 100.0% 99.1% **GLSIVDRATQRLGGTLTLDKSLELGGLSVLVKLPKVLHLHETRV**

23 PSU120 100.0% 99.1% **GLSIVDRATQRLGGTLTLDKSLELGGLSVLVKLPKVLHLHETRV**

24 KUSSH35 100.0% 99.3% **GLSIVDRATQRLGGTLTLDKSLELGGLSVLVKLPKVLHLHETRV**

25 KUFAR56 100.0% 99.3% **GLSIVDRATQRLGGTLTLDKSLELGGLSVLVKLPKVLHLHETRV**

26 KUSSH15 100.0% 99.3% **GLSIVDRATQRLGGTLTLDKSLELGGLSVLVKLPKVLHLHETRV**

27 KUSSH37 100.0% 99.3% **GLSIVDRATQRLGGTLTLDKSLELGGLSVLVKLPKVLHLHETRV**

28 KUSSH36 100.0% 99.3% **GLSIVDRATQRLGGTLTLDKSLELGGLSVLVKLPKVLHLHETRV**

29 130 100.0% 99.3% **GLSIVDRATQRLGGTLTLDKSLELGGLSVLVKLPKVLHLHETRV**

30 Ab34 100.0% 99.3% **GLSIVDRATQRLGGTLTLDKSLELGGLSVLVKLPKVLHLHETRV**

31 Ab35 100.0% 99.3% **GLSIVDRATQRLGGTLTLDKSLELGGLSVLVKLPKVLHLHETRV**

32 Ab36 100.0% 99.3% **GLSIVDRATQRLGGTLTLDKSLELGGLSVLVKLPKVLHLHETRV**

33 Ab38 100.0% 99.3% **GLSIVDRATQRLGGTLTLDKSLELGGLSVLVKLPKVLHLHETRV**

34 Ab40 100.0% 99.3% **GLSIVDRATQRLGGTLTLDKSLELGGLSVLVKLPKVLHLHETRV**

35 Ab41 100.0% 99.3% **GLSIVDRATQRLGGTLTLDKSLELGGLSVLVKLPKVLHLHETRV**

36 Ab15 100.0% 99.3% **GLSIVDRATQRLGGTLTLDKSLELGGLSVLVKLPKVLHLHETRV**

37 Ab65 100.0% 99.3% **GLSIVDRATQRLGGTLTLDKSLELGGLSVLVKLPKVLHLHETRV**

38 Ab64 100.0% 99.3% **GLSIVDRATQRLGGTLTLDKSLELGGLSVLVKLPKVLHLHETRV**

39 AB22 100.0% 99.1% **GLSIVDRATQRLGGTLTLDKSLELGGLSVLVKLPKVLHLHETRV**

**Supplementary Figure 7: Multiple sequence alignment (MSA) of the predicted amino acid sequence of PmrB carried by ST2^Pas^ and ST570^Pas^ (GC2) isolates and close genomes retrieved from the BV-BRC database compared to the respective gene in *A. baumannii* ATCC19606.** MSA was created by the A multiple alignment viewer MView hosted by the EMBL-EBI; cov, coverage; pid, percent identity.

cov pid  **1** **[ . . . . : . . .** **80**

1 ATCC19606 100.0% 100.0% **MHYSLKKRLIWGTSIFSVILGCILIFSAYKVALQEVDEILDTQMKYLAERTAEHPLKTVSSKFDFHKTYHEEDLFIDIWA**

2 M03 100.0% 99.8% **MHYSLKKRLIWGTSIFSVILGCILIFSAYKVALQEVDEILDTQMKYLAERTAEHPLKTVSSKFDFHKTYHEEDLFIDIWA**

3 M14 100.0% 99.8% **MHYSLKKRLIWGTSIFSVILGCILIFSAYKVALQEVDEILDTQMKYLAERTAEHPLKTVSSKFDFHKTYHEEDLFIDIWA**

4 AB_1649-8 100.0% 99.8% **MHYSLKKRLIWGTSIFSVILGCILIFSAYKVALQEVDEILDTQMKYLAERTAEHPLKTVSSKFDFHKTYHEEDLFIDIWA**

5 AB_1650-8 100.0% 99.8% **MHYSLKKRLIWGTSIFSVILGCILIFSAYKVALQEVDEILDTQMKYLAERTAEHPLKTVSSKFDFHKTYHEEDLFIDIWA**

6 UV_1036 100.0% 99.8% **MHYSLKKRLIWGTSIFSVILGCILIFSAYKVALQEVDEILDTQMKYLAERTAEHPLKTVSSKFDFHKTYHEEDLFIDIWA**

7 259_an 100.0% 99.8% **MHYSLKKRLIWGTSIFSVILGCILIFSAYKVALQEVDEILDTQMKYLAERTAEHPLKTVSSKFDFHKTYHEEDLFIDIWA**

8 276_ax 100.0% 99.8% **MHYSLKKRLIWGTSIFSVILGCILIFSAYKVALQEVDEILDTQMKYLAERTAEHPLKTVSSKFDFHKTYHEEDLFIDIWA**

9 PT061 100.0% 99.5% **MHYSLKKRLIWGTSIFSVILGCILIFSAYKVALQEVDEILDTQMKYLAERTAEHPLKTVSSKFDFHKTYHEEDLFIDIWA**

10 PT003 100.0% 99.5% **MHYSLKKRLIWGTSIFSVILGCILIFSAYKVALQEVDEILDTQMKYLAERTAEHPLKTVSSKFDFHKTYHEEDLFIDIWA**

11 PSU068 100.0% 99.5% **MHYSLKKRLIWGTSIFSVILGCILIFSAYKVALQEVDEILDTQMKYLAERTAEHPLKTVSSKFDFHKTYHEEDLFIDIWA**

12 PSU073 100.0% 99.5% **MHYSLKKRLIWGTSIFSVILGCILIFSAYKVALQEVDEILDTQMKYLAERTAEHPLKTVSSKFDFHKTYHEEDLFIDIWA**

13 CCBH26501 100.0% 99.5% **MHYSLKKRLIWGTSIFSVILGCILIFSAYKVALQEVDEILDTQMKYLAERTAEHPLKTVSSKFDFHKTYHEEDLFIDIWA**

14 AB363 100.0% 99.8% **MHYSLKKRLIWGTSIFSVILGCILIFSAYKVALQEVDEILDTQMKYLAERTAEHPLKTVSSKFDFHKTYHEEDLFIDIWA**

15 KUSSH08 100.0% 99.8% **MHYSLKKRLIWGTSIFSVILGCILIFSAYKVALQEVDEILDTQMKYLAERTAEHPLKTVSSKFDFHKTYHEEDLFIDIWA**

16 KUSSH14 100.0% 99.8% **MHYSLKKRLIWGTSIFSVILGCILIFSAYKVALQEVDEILDTQMKYLAERTAEHPLKTVSSKFDFHKTYHEEDLFIDIWA**

17 TUMA 100.0% 99.8% **MHYSLKKRLIWGTSIFSVILGCILIFSAYKVALQEVDEILDTQMKYLAERTAEHPLKTVSSKFDFHKTYHEEDLFIDIWA**

18 4300STDY7045886 100.0% 99.8% **MHYSLKKRLIWGTSIFSVILGCILIFSAYKVALQEVDEILDTQMKYLAERTAEHPLKTVSSKFDFHKTYHEEDLFIDIWA**

19 Aci00866 100.0% 99.8% **MHYSLKKRLIWGTSIFSVILGCILIFSAYKVALQEVDEILDTQMKYLAERTAEHPLKTVSSKFDFHKTYHEEDLFIDIWA**

20 Aci00860 100.0% 99.8% **MHYSLKKRLIWGTSIFSVILGCILIFSAYKVALQEVDEILDTQMKYLAERTAEHPLKTVSSKFDFHKTYHEEDLFIDIWA**

21 Aci00848 100.0% 99.8% **MHYSLKKRLIWGTSIFSVILGCILIFSAYKVALQEVDEILDTQMKYLAERTAEHPLKTVSSKFDFHKTYHEEDLFIDIWA**

22 MRSN351524 100.0% 99.8% **MHYSLKKRLIWGTSIFSVILGCILIFSAYKVALQEVDEILDTQMKYLAERTAEHPLKTVSSKFDFHKTYHEEDLFIDIWA**

cov pid  **81**  **. 1 . . . . : .** **160**

1 ATCC19606 100.0% 100.0% **YKDQAHLSHHLHLLVPPVEQAGFYSHKTAQGIVRTYVLPLKDYQIQVSQQERVREAFAWELAGSMFIPYLIILPFAIFAL**

2 M03 100.0% 99.8% **YKDQAHLSHHLHLLVPPVEQAGFYSHKTAQGIVRTYVLPLKDYQIQVSQQERVREAFAWELAGSMFIPYLIILPFAIFAL**

3 M14 100.0% 99.8% **YKDQAHLSHHLHLLVPPVEQAGFYSHKTAQGIVRTYVLPLKDYQIQVSQQERVREAFAWELAGSMFIPYLIILPFAIFAL**

4 AB_1649-8 100.0% 99.8% **YKDQAHLSHHLHLLVPPVEQAGFYSHKTAQGIVRTYVLPLKDYQIQVSQQERVREAFAWELAGSMFIPYLIILPFAIFAL**

5 AB_1650-8 100.0% 99.8% **YKDQAHLSHHLHLLVPPVEQAGFYSHKTAQGIVRTYVLPLKDYQIQVSQQERVREAFAWELAGSMFIPYLIILPFAIFAL**

6 UV_1036 100.0% 99.8% **YKDQAHLSHHLHLLVPPVEQAGFYSHKTAQGIVRTYVLPLKDYQIQVSQQERVREAFAWELAGSMFIPYLIILPFAIFAL**

7 259_an 100.0% 99.8% **YKDQAHLSHHLHLLVPPVEQAGFYSHKTAQGIVRTYVLPLKDYQIQVSQQERVREAFAWELAGSMFIPYLIILPFAIFAL**

8 276_ax 100.0% 99.8% **YKDQAHLSHHLHLLVPPVEQAGFYSHKTAQGIVRTYVLPLKDYQIQVSQQERVREAFAWELAGSMFIPYLIILPFAIFAL**

9 PT061 100.0% 99.5% **YKDQAHLSHHLHLLVPPVEQAGFYSHKTAQGIVRTYVLPLKDYQIQVSQQERVREAFAWELAGSMFIPYLIILPFAIFAL**

10 PT003 100.0% 99.5% **YKDQAHLSHHLHLLVPPVEQAGFYSHKTAQGIVRTYVLPLKDYQIQVSQQERVREAFAWELAGSMFIPYLIILPFAIFAL**

11 PSU068 100.0% 99.5% **YKDQAHLSHHLHLLVPPVEQAGFYSHKTAQGIVRTYVLPLKDYQIQVSQQERVREAFAWELAGSMFIPYLIILPFAIFAL**

12 PSU073 100.0% 99.5% **YKDQAHLSHHLHLLVPPVEQAGFYSHKTAQGIVRTYVLPLKDYQIQVSQQERVREAFAWELAGSMFIPYLIILPFAIFAL**

13 CCBH26501 100.0% 99.5% **YKDQAHLSHHLHLLVPPVEQAGFYSHKTAQGIVRTYVLPLKDYQIQVSQQERVREAFAWELAGSMFIPYLIILPFAIFAL**

14 AB363 100.0% 99.8% **YKDQAHLSHHLHLLVPPVEQAGFYSHKTAQGIVRTYVLPLKDYQIQVSQQERVREAFAWELAGSMFIPYLIILPFAIFAL**

15 KUSSH08 100.0% 99.8% **YKDQAHLSHHLHLLVPPVEQAGFYSHKTAQGIVRTYVLPLKDYQIQVSQQERVREAFAWELAGSMFIPYLIILPFAIFAL**

16 KUSSH14 100.0% 99.8% **YKDQAHLSHHLHLLVPPVEQAGFYSHKTAQGIVRTYVLPLKDYQIQVSQQERVREAFAWELAGSMFIPYLIILPFAIFAL**

17 TUMA 100.0% 99.8% **YKDQAHLSHHLHLLVPPVEQAGFYSHKTAQGIVRTYVLPLKDYQIQVSQQERVREAFAWELAGSMFIPYLIILPFAIFAL**

18 4300STDY7045886 100.0% 99.8% **YKDQAHLSHHLHLLVPPVEQAGFYSHKTAQGIVRTYVLPLKDYQIQVSQQERVREAFAWELAGSMFIPYLIILPFAIFAL**

19 Aci00866 100.0% 99.8% **YKDQAHLSHHLHLLVPPVEQAGFYSHKTAQGIVRTYVLPLKDYQIQVSQQERVREAFAWELAGSMFIPYLIILPFAIFAL**

20 Aci00860 100.0% 99.8% **YKDQAHLSHHLHLLVPPVEQAGFYSHKTAQGIVRTYVLPLKDYQIQVSQQERVREAFAWELAGSMFIPYLIILPFAIFAL**

21 Aci00848 100.0% 99.8% **YKDQAHLSHHLHLLVPPVEQAGFYSHKTAQGIVRTYVLPLKDYQIQVSQQERVREAFAWELAGSMFIPYLIILPFAIFAL**

22 MRSN351524 100.0% 99.8% **YKDQAHLSHHLHLLVPPVEQAGFYSHKTAQGIVRTYVLPLKDYQIQVSQQERVREAFAWELAGSMFIPYLIILPFAIFAL**

cov pid **161**  **. . . 2 . . . .** **240**

1 ATCC19606 100.0% 100.0% **AAIIRRGLKPIDDFKNELKERDSEELTPIEVHDYPQELLPTIDEMNRLFERISKAQNEQKQFIADAAHELRTPVTALNLQ**

2 M03 100.0% 99.8% **AAIIRRGLKPIDDFKNELKERDSEELTPIEVHDYPQELLPTIDEMNRLFERISKAQNEQKQFIADAAHELRTPVTALNLQ**

3 M14 100.0% 99.8% **AAIIRRGLKPIDDFKNELKERDSEELTPIEVHDYPQELLPTIDEMNRLFERISKAQNEQKQFIADAAHELRTPVTALNLQ**

4 AB_1649-8 100.0% 99.8% **AAIIRRGLKPIDDFKNELKERDSEELTPIEVHDYPQELLPTIDEMNRLFERISKAQNEQKQFIADAAHELRTPVTALNLQ**

5 AB_1650-8 100.0% 99.8% **AAIIRRGLKPIDDFKNELKERDSEELTPIEVHDYPQELLPTIDEMNRLFERISKAQNEQKQFIADAAHELRTPVTALNLQ**

6 UV_1036 100.0% 99.8% **AAIIRRGLKPIDDFKNELKERDSEELTPIEVHDYPQELLPTIDEMNRLFERISKAQNEQKQFIADAAHELRTPVTALNLQ**

7 259_an 100.0% 99.8% **AAIIRRGLKPIDDFKNELKERDSEELTPIEVHDYPQELLPTIDEMNRLFERISKAQNEQKQFIADAAHELRTPVTALNLQ**

8 276_ax 100.0% 99.8% **AAIIRRGLKPIDDFKNELKERDSEELTPIEVHDYPQELLPTIDEMNRLFERISKAQNEQKQFIADAAHELRTPVTALNLQ**

9 PT061 100.0% 99.5% **AAIIRRGLKPIDDFKNELKERDSEELTPIEVHDYPQELLPTIDEMNRLFERISKAQNEQKQFIADAAHELRTPVTALNLQ**

10 PT003 100.0% 99.5% **AAIIRRGLKPIDDFKNELKERDSEELTPIEVHDYPQELLPTIDEMNRLFERISKAQNEQKQFIADAAHELRTPVTALNLQ**

11 PSU068 100.0% 99.5% **AAIIRRGLKPIDDFKNELKERDSEELTPIEVHDYPQELLPTIDEMNRLFERISKAQNEQKQFIADAAHELRTPVTALNLQ**

12 PSU073 100.0% 99.5% **AAIIRRGLKPIDDFKNELKERDSEELTPIEVHDYPQELLPTIDEMNRLFERISKAQNEQKQFIADAAHELRTPVTALNLQ**

13 CCBH26501 100.0% 99.5% **AAIIRRGLKPIDDFKNELKERDSEELTPIEVHDYPQELLPTIDEMNRLFERISKAQNEQKQFIADAAHELRTPVTALNLQ**

14 AB363 100.0% 99.8% **AAIIRRGLKPIDDFKNELKERDSEELTPIEVHDYPQELLPTIDEMNRLFERISKAQNEQKQFIADAAHELRTPVTALNLQ**

15 KUSSH08 100.0% 99.8% **AAIIRRGLKPIDDFKNELKERDSEELTPIEVHDYPQELLPTIDEMNRLFERISKAQNEQKQFIADAAHELRTPVTALNLQ**

16 KUSSH14 100.0% 99.8% **AAIIRRGLKPIDDFKNELKERDSEELTPIEVHDYPQELLPTIDEMNRLFERISKAQNEQKQFIADAAHELRTPVTALNLQ**

17 TUMA 100.0% 99.8% **AAIIRRGLKPIDDFKNELKERDSEELTPIEVHDYPQELLPTIDEMNRLFERISKAQNEQKQFIADAAHELRTPVTALNLQ**

18 4300STDY7045886 100.0% 99.8% **AAIIRRGLKPIDDFKNELKERDSEELTPIEVHDYPQELLPTIDEMNRLFERISKAQNEQKQFIADAAHELRTPVTALNLQ**

19 Aci00866 100.0% 99.8% **AAIIRRGLKPIDDFKNELKERDSEELTPIEVHDYPQELLPTIDEMNRLFERISKAQNEQKQFIADAAHELRTPVTALNLQ**

20 Aci00860 100.0% 99.8% **AAIIRRGLKPIDDFKNELKERDSEELTPIEVHDYPQELLPTIDEMNRLFERISKAQNEQKQFIADAAHELRTPVTALNLQ**

21 Aci00848 100.0% 99.8% **AAIIRRGLKPIDDFKNELKERDSEELTPIEVHDYPQELLPTIDEMNRLFERISKAQNEQKQFIADAAHELRTPVTALNLQ**

22 MRSN351524 100.0% 99.8% **AAIIRRGLKPIDDFKNELKERDSEELTPIEVHDYPQELLPTIDEMNRLFERISKAQNEQKQFIADAAHELRTPVTALNLQ**

cov pid **241**  **: . . . . 3 . .** **320**

1 ATCC19606 100.0% 100.0% **TKILLSQFPEHESLQNLSKGLARIQHLVTQLLALAKQDVTLSMVEPTGYFQLNDVALNCVEQLVNLAMQKEIDLGFVRNE**

2 M03 100.0% 99.8% **TKILLSQFPEHESLQNLSKGLARIQHLVTQLLALAKQDVTLSMVEPTGYFQLNDVALNCVEQLVNLAMQKEIDLGFVRNE**

3 M14 100.0% 99.8% **TKILLSQFPEHESLQNLSKGLARIQHLVTQLLALAKQDVTLSMVEPTGYFQLNDVALNCVEQLVNLAMQKEIDLGFVRNE**

4 AB_1649-8 100.0% 99.8% **TKILLSQFPEHESLQNLSKGLARIQHLVTQLLALAKQDVTLSMVEPTGYFQLNDVALNCVEQLVNLAMQKEIDLGFVRNE**

5 AB_1650-8 100.0% 99.8% **TKILLSQFPEHESLQNLSKGLARIQHLVTQLLALAKQDVTLSMVEPTGYFQLNDVALNCVEQLVNLAMQKEIDLGFVRNE**

6 UV_1036 100.0% 99.8% **TKILLSQFPEHESLQNLSKGLARIQHLVTQLLALAKQDVTLSMVEPTGYFQLNDVALNCVEQLVNLAMQKEIDLGFVRNE**

7 259_an 100.0% 99.8% **TKILLSQFPEHESLQNLSKGLARIQHLVTQLLALAKQDVTLSMVEPTGYFQLNDVALNCVEQLVNLAMQKEIDLGFVRNE**

8 276_ax 100.0% 99.8% **TKILLSQFPEHESLQNLSKGLARIQHLVTQLLALAKQDVTLSMVEPTGYFQLNDVALNCVEQLVNLAMQKEIDLGFVRNE**

9 PT061 100.0% 99.5% **TKIMLSQFPEHESLQNLSKGLARIQHLVTQLLALAKQDVTLSMVEPTGYFQLNDVALNCVEQLVNLAMQKEIDLGFVRNE**

10 PT003 100.0% 99.5% **TKIMLSQFPEHESLQNLSKGLARIQHLVTQLLALAKQDVTLSMVEPTGYFQLNDVALNCVEQLVNLAMQKEIDLGFVRNE**

11 PSU068 100.0% 99.5% **TKIMLSQFPEHESLQNLSKGLARIQHLVTQLLALAKQDVTLSMVEPTGYFQLNDVALNCVEQLVNLAMQKEIDLGFVRNE**

12 PSU073 100.0% 99.5% **TKIMLSQFPEHESLQNLSKGLARIQHLVTQLLALAKQDVTLSMVEPTGYFQLNDVALNCVEQLVNLAMQKEIDLGFVRNE**

13 CCBH26501 100.0% 99.5% **TKILLSQFPEHESLQNLSKGLARIQHLVTQFLALAKQDVTLSMVEPTGYFQLNDVALNCVEQLVNLAMQKEIDLGFVRNE**

14 AB363 100.0% 99.8% **TKILLSQFPEHESLQNLSKGLARIQHLVTQLLALAKQDVTLSMVEPTGYFQLNDVALNCVEQLVNLAMQKEIDLGFVRNE**

15 KUSSH08 100.0% 99.8% **TKILLSQFPEHESLQNLSKGLARIQHLVTQLLALAKQDVTLSMVEPTGYFQLNDVALNCVEQLVNLAMQKEIDLGFVRNE**

16 KUSSH14 100.0% 99.8% **TKILLSQFPEHESLQNLSKGLARIQHLVTQLLALAKQDVTLSMVEPTGYFQLNDVALNCVEQLVNLAMQKEIDLGFVRNE**

17 TUMA 100.0% 99.8% **TKILLSQFPEHESLQNLSKGLARIQHLVTQLLALAKQDVTLSMVEPTGYFQLNDVALNCVEQLVNLAMQKEIDLGFVRNE**

18 4300STDY7045886 100.0% 99.8% **TKILLSQFPEHESLQNLSKGLARIQHLVTQLLALAKQDVTLSMVEPTGYFQLNDVALNCVEQLVNLAMQKEIDLGFVRNE**

19 Aci00866 100.0% 99.8% **TKILLSQFPEHESLQNLSKGLARIQHLVTQLLALAKQDVTLSMVEPTGYFQLNDVALNCVEQLVNLAMQKEIDLGFVRNE**

20 Aci00860 100.0% 99.8% **TKILLSQFPEHESLQNLSKGLARIQHLVTQLLALAKQDVTLSMVEPTGYFQLNDVALNCVEQLVNLAMQKEIDLGFVRNE**

21 Aci00848 100.0% 99.8% **TKILLSQFPEHESLQNLSKGLARIQHLVTQLLALAKQDVTLSMVEPTGYFQLNDVALNCVEQLVNLAMQKEIDLGFVRNE**

22 MRSN351524 100.0% 99.8% **TKILLSQFPEHESLQNLSKGLARIQHLVTQLLALAKQDVTLSMVEPTGYFQLNDVALNCVEQLVNLAMQKEIDLGFVRNE**

cov pid **321**  **. . : . . . . 4** **400**

1 ATCC19606 100.0% 100.0% **PIEMHSIEPTVHSIIFNLIDNAIKYTPHQGVINISVYTDPDHYACIQIEDSGAGIDPENYDKVLKRFYRVHHHLEVGSGL**

2 M03 100.0% 99.8% **PIEMHSIEPTVHSIIFNLIDNAIKYTPHQGVINISVYTDPDHYACIQIEDSGAGIDPENYDKVLKRFYRVHHHLEVGSGL**

3 M14 100.0% 99.8% **PIEMHSIEPTVHSIIFNLIDNAIKYTPHQGVINISVYTDPDHYACIQIEDSGAGIDPENYDKVLKRFYRVHHHLEVGSGL**

4 AB_1649-8 100.0% 99.8% **PIEMHSIEPTVHSIIFNLIDNAIKYTPHQGVINISVYTDPDHYACIQIEDSGAGIDPENYDKVLKRFYRVHHHLEVGSGL**

5 AB_1650-8 100.0% 99.8% **PIEMHSIEPTVHSIIFNLIDNAIKYTPHQGVINISVYTDPDHYACIQIEDSGAGIDPENYDKVLKRFYRVHHHLEVGSGL**

6 UV_1036 100.0% 99.8% **PIEMHSIEPTVHSIIFNLIDNAIKYTPHQGVINISVYTDPDHYACIQIEDSGAGIDPENYDKVLKRFYRVHHHLEVGSGL**

7 259_an 100.0% 99.8% **PIEMHSIEPTVHSIIFNLIDNAIKYTPHQGVINISVYTDPDHYACIQIEDSGAGIDPENYDKVLKRFYRVHHHLEVGSGL**

8 276_ax 100.0% 99.8% **PIEMHSIEPTVHSIIFNLIDNAIKYTPHQGVINISVYTDPDHYACIQIEDSGAGIDPENYDKVLKRFYRVHHHLEVGSGL**

9 PT061 100.0% 99.5% **PIEMHSIEPTVHSIIFNLIDNAIKYTPHQGVINISVYTDPDHYACIQIEDSGAGIDPENYDKVLKRFYRVHHHLEVGSGL**

10 PT003 100.0% 99.5% **PIEMHSIEPTVHSIIFNLIDNAIKYTPHQGVINISVYTDPDHYACIQIEDSGAGIDPENYDKVLKRFYRVHHHLEVGSGL**

11 PSU068 100.0% 99.5% **PIEMHSIEPTVHSIIFNLIDNAIKYTPHQGVINISVYTDPDHYACIQIEDSGAGIDPENYDKVLKRFYRVHHHLEVGSGL**

12 PSU073 100.0% 99.5% **PIEMHSIEPTVHSIIFNLIDNAIKYTPHQGVINISVYTDPDHYACIQIEDSGAGIDPENYDKVLKRFYRVHHHLEVGSGL**

13 CCBH26501 100.0% 99.5% **PIEMHSIEPTVHSIIFNLIDNAIKYTPHQGVINISVYTDPDHYACIQIEDSGAGIDPENYDKVLKRFYRVHHHLEVGSGL**

14 AB363 100.0% 99.8% **PIEMHSIEPTVHSIIFNLIDNAIKYTPHQGVINISVYTDPDHYACIQIEDSGAGIDPENYDKVLKRFYRVHHHLEVGSGL**

15 KUSSH08 100.0% 99.8% **PIEMHSIEPTVHSIIFNLIDNAIKYTPHQGVINISVYTDPDHYACIQIEDSGAGIDPENYDKVLKRFYRVHHHLEVGSGL**

16 KUSSH14 100.0% 99.8% **PIEMHSIEPTVHSIIFNLIDNAIKYTPHQGVINISVYTDPDHYACIQIEDSGAGIDPENYDKVLKRFYRVHHHLEVGSGL**

17 TUMA 100.0% 99.8% **PIEMHSIEPTVHSIIFNLIDNAIKYTPHQGVINISVYTDPDHYACIQIEDSGAGIDPENYDKVLKRFYRVHHHLEVGSGL**

18 4300STDY7045886 100.0% 99.8% **PIEMHSIEPTVHSIIFNLIDNAIKYTPHQGVINISVYTDPDHYACIQIEDSGAGIDPENYDKVLKRFYRVHHHLEVGSGL**

19 Aci00866 100.0% 99.8% **PIEMHSIEPTVHSIIFNLIDNAIKYTPHQGVINISVYTDPDHYACIQIEDSGAGIDPENYDKVLKRFYRVHHHLEVGSGL**

20 Aci00860 100.0% 99.8% **PIEMHSIEPTVHSIIFNLIDNAIKYTPHQGVINISVYTDPDHYACIQIEDSGAGIDPENYDKVLKRFYRVHHHLEVGSGL**

21 Aci00848 100.0% 99.8% **PIEMHSIEPTVHSIIFNLIDNAIKYTPHQGVINISVYTDPDHYACIQIEDSGAGIDPENYDKVLKRFYRVHHHLEVGSGL**

22 MRSN351524 100.0% 99.8% **PIEMHSIEPTVHSIIFNLIDNAIKYTPHQGVINISVYTDPDHYACIQIEDSGAGIDPENYDKVLKRFYRVHHHLEVGSGL**

cov pid **401**  **. . . . ]** **444**

1 ATCC19606 100.0% 100.0% **GLSIVDRATQRLGGTLTLDKSLELGGLSVLVKLPKVLHLNETRA**

2 M03 100.0% 99.8% **GLSIVDRATQRLGGTLTLDKSLELGGLSVLVKLPKVLHLHETRA**

3 M14 100.0% 99.8% **GLSIVDRATQRLGGTLTLDKSLELGGLSVLVKLPKVLHLHETRA**

4 AB_1649-8 100.0% 99.8% **GLSIVDRATQRLGGTLTLDKSLELGGLSVLVKLPKVLHLHETRA**

5 AB_1650-8 100.0% 99.8% **GLSIVDRATQRLGGTLTLDKSLELGGLSVLVKLPKVLHLHETRA**

6 UV_1036 100.0% 99.8% **GLSIVDRATQRLGGTLTLDKSLELGGLSVLVKLPKVLHLHETRA**

7 259_an 100.0% 99.8% **GLSIVDRATQRLGGTLTLDKSLELGGLSVLVKLPKVLHLHETRA**

8 276_ax 100.0% 99.8% **GLSIVDRATQRLGGTLTLDKSLELGGLSVLVKLPKVLHLHETRA**

9 PT061 100.0% 99.5% **GLSIVDRATQRLGGTLTLDKSLELGGLSVLVKLPKVLHLHETRA**

10 PT003 100.0% 99.5% **GLSIVDRATQRLGGTLTLDKSLELGGLSVLVKLPKVLHLHETRA**

11 PSU068 100.0% 99.5% **GLSIVDRATQRLGGTLTLDKSLELGGLSVLVKLPKVLHLHETRA**

12 PSU073 100.0% 99.5% **GLSIVDRATQRLGGTLTLDKSLELGGLSVLVKLPKVLHLHETRA**

13 CCBH26501 100.0% 99.5% **GLSIVDRATQRLGGTLTLDKSLELGGLSVLVKLPKVLHLHETRA**

14 AB363 100.0% 99.8% **GLSIVDRATQRLGGTLTLDKSLELGGLSVLVKLPKVLHLHETRA**

15 KUSSH08 100.0% 99.8% **GLSIVDRATQRLGGTLTLDKSLELGGLSVLVKLPKVLHLHETRA**

16 KUSSH14 100.0% 99.8% **GLSIVDRATQRLGGTLTLDKSLELGGLSVLVKLPKVLHLHETRA**

17 TUMA 100.0% 99.8% **GLSIVDRATQRLGGTLTLDKSLELGGLSVLVKLPKVLHLHETRA**

18 4300STDY7045886 100.0% 99.8% **GLSIVDRATQRLGGTLTLDKSLELGGLSVLVKLPKVLHLHETRA**

19 Aci00866 100.0% 99.8% **GLSIVDRATQRLGGTLTLDKSLELGGLSVLVKLPKVLHLHETRA**

20 Aci00860 100.0% 99.8% **GLSIVDRATQRLGGTLTLDKSLELGGLSVLVKLPKVLHLHETRA**

21 Aci00848 100.0% 99.8% **GLSIVDRATQRLGGTLTLDKSLELGGLSVLVKLPKVLHLHETRA**

22 MRSN351524 100.0% 99.8% **GLSIVDRATQRLGGTLTLDKSLELGGLSVLVKLPKVLHLHETRA**

**Supplementary Figure 8: Multiple sequence alignment (MSA) of the predicted amino acid sequence of PmrB carried by ST113^Pas^ (GC7) isolates and close genomes retrieved from the BV-BRC database compared to the respective gene in *A. baumannii* ATCC19606.** MSA was created by the A multiple alignment viewer MView hosted by the EMBL-EBI; cov, coverage; pid, percent identity.

cov pid  **1** **[ . . . . : . . .** **80**

1 ATCC19606 100.0% 100.0% **MHYSLKKRLIWGTSIFSVILGCILIFSAYKVALQEVDEILDTQMKYLAERTAEHPLKTVSSKFDFHKTYHEEDLFIDIWA**

2 M02 100.0% 99.8% **MHYSLKKRLIWGTSIFSVILGCILIFSAYKVALQEVDEILDTQMKYLAERTAEHPLKTVSSKFDFHKTYHEEDLFIDIWA**

3 M11 100.0% 99.8% **MHYSLKKRLIWGTSIFSVILGCILIFSAYKVALQEVDEILDTQMKYLAERTAEHPLKTVSSKFDFHKTYHEEDLFIDIWA**

4 M18 100.0% 99.8% **MHYSLKKRLIWGTSIFSVILGCILIFSAYKVALQEVDEILDTQMKYLAERTAEHPLKTVSSKFDFHKTYHEEDLFIDIWA**

5 15953 100.0% 99.8% **MHYSLKKRLIWGTSIFSVILGCILIFSAYKVALQEVDEILDTQMKYLAERTAEHPLKTVSSKFDFHKTYHEEDLFIDIWA**

6 15946 100.0% 99.8% **MHYSLKKRLIWGTSIFSVILGCILIFSAYKVALQEVDEILDTQMKYLAERTAEHPLKTVSSKFDFHKTYHEEDLFIDIWA**

7 Survcare112 100.0% 99.8% **MHYSLKKRLIWGTSIFSVILGCILIFSAYKVALQEVDEILDTQMKYLAERTAEHPLKTVSSKFDFHKTYHEEDLFIDIWA**

8 Cl300 100.0% 99.8% **MHYSLKKRLIWGTSIFSVILGCILIFSAYKVALQEVDEILDTQMKYLAERTAEHPLKTVSSKFDFHKTYHEEDLFIDIWA**

9 17A1955 100.0% 99.8% **MHYSLKKRLIWGTSIFSVILGCILIFSAYKVALQEVDEILDTQMKYLAERTAEHPLKTVSSKFDFHKTYHEEDLFIDIWA**

10 ACMH-6200 100.0% 99.8% **MHYSLKKRLIWGTSIFSVILGCILIFSAYKVALQEVDEILDTQMKYLAERTAEHPLKTVSSKFDFHKTYHEEDLFIDIWA**

11 ACMH-6201 100.0% 99.8% **MHYSLKKRLIWGTSIFSVILGCILIFSAYKVALQEVDEILDTQMKYLAERTAEHPLKTVSSKFDFHKTYHEEDLFIDIWA**

12 MIN-015 100.0% 99.8% **MHYSLKKRLIWGTSIFSVILGCILIFSAYKVALQEVDEILDTQMKYLAERTAEHPLKTVSSKFDFHKTYHEEDLFIDIWA**

13 AB177-VUB 100.0% 99.8% **MHYSLKKRLIWGTSIFSVILGCILIFSAYKVALQEVDEILDTQMKYLAERTAEHPLKTVSSKFDFHKTYHEEDLFIDIWA**

14 P116A 100.0% 99.8% **MHYSLKKRLIWGTSIFSVILGCILIFSAYKVALQEVDEILDTQMKYLAERTAEHPLKTVSSKFDFHKTYHEEDLFIDIWA**

15 26 100.0% 99.8% **MHYSLKKRLIWGTSIFSVILGCILIFSAYKVALQEVDEILDTQMKYLAERTAEHPLKTVSSKFDFHKTYHEEDLFIDIWA**

16 AR_0037 100.0% 99.8% **MHYSLKKRLIWGTSIFSVILGCILIFSAYKVALQEVDEILDTQMKYLAERTAEHPLKTVSSKFDFHKTYHEEDLFIDIWA**

17 AR_0033 100.0% 99.8% **MHYSLKKRLIWGTSIFSVILGCILIFSAYKVALQEVDEILDTQMKYLAERTAEHPLKTVSSKFDFHKTYHEEDLFIDIWA**

18 AB-A 100.0% 99.8% **MHYSLKKRLIWGTSIFSVILGCILIFSAYKVALQEVDEILDTQMKYLAERTAEHPLKTVSSKFDFHKTYHEEDLFIDIWA**

19 AB-C 100.0% 99.8% **MHYSLKKRLIWGTSIFSVILGCILIFSAYKVALQEVDEILDTQMKYLAERTAEHPLKTVSSKFDFHKTYHEEDLFIDIWA**

20 AB-B 100.0% 99.8% **MHYSLKKRLIWGTSIFSVILGCILIFSAYKVALQEVDEILDTQMKYLAERTAEHPLKTVSSKFDFHKTYHEEDLFIDIWA**

21 AE27M 100.0% 99.8% **MHYSLKKRLIWGTSIFSVILGCILIFSAYKVALQEVDEILDTQMKYLAERTAEHPLKTVSSKFDFHKTYHEEDLFIDIWA**

22 AE3M 100.0% 99.8% **MHYSLKKRLIWGTSIFSVILGCILIFSAYKVALQEVDEILDTQMKYLAERTAEHPLKTVSSKFDFHKTYHEEDLFIDIWA**

23 MBL_M1 100.0% 99.8% **MHYSLKKRLIWGTSIFSVILGCILIFSAYKVALQEVDEILDTQMKYLAERTAEHPLKTVSSKFDFHKTYHEEDLFIDIWA**

24 MBL_M6 100.0% 99.8% **MHYSLKKRLIWGTSIFSVILGCILIFSAYKVALQEVDEILDTQMKYLAERTAEHPLKTVSSKFDFHKTYHEEDLFIDIWA**

25 MBL_M10 100.0% 99.8% **MHYSLKKRLIWGTSIFSVILGCILIFSAYKVALQEVDEILDTQMKYLAERTAEHPLKTVSSKFDFHKTYHEEDLFIDIWA**

26 MBL_M9 100.0% 99.8% **MHYSLKKRLIWGTSIFSVILGCILIFSAYKVALQEVDEILDTQMKYLAERTAEHPLKTVSSKFDFHKTYHEEDLFIDIWA**

27 R11 100.0% 99.8% **MHYSLKKRLIWGTSIFSVILGCILIFSAYKVALQEVDEILDTQMKYLAERTAEHPLKTVSSKFDFHKTYHEEDLFIDIWA**

28 Ab-NDM-1 100.0% 99.5% **MHYSLKKRLIWGTSIFSVILGCILIFSAYKVALQEVDEILDTQMKYLAERTAEHPLKTVSSKFDFHKTYHEEDLFIDIWA**

29 MRSN15574 100.0% 99.8% **MHYSLKKRLIWGTSIFSVILGCILIFSAYKVALQEVDEILDTQMKYLAERTAEHPLKTVSSKFDFHKTYHEEDLFIDIWA**

cov pid  **81**  **. 1 . . . . : .** **160**

1 ATCC19606 100.0% 100.0% **YKDQAHLSHHLHLLVPPVEQAGFYSHKTAQGIVRTYVLPLKDYQIQVSQQERVREAFAWELAGSMFIPYLIILPFAIFAL**

2 M02 100.0% 99.8% **YKDQAHLSHHLHLLVPPVEQAGFYSHKTAQGIVRTYVLPLKDYQIQVSQQERVREAFAWELAGSMFIPYLIILPFAIFAL**

3 M11 100.0% 99.8% **YKDQAHLSHHLHLLVPPVEQAGFYSHKTAQGIVRTYVLPLKDYQIQVSQQERVREAFAWELAGSMFIPYLIILPFAIFAL**

4 M18 100.0% 99.8% **YKDQAHLSHHLHLLVPPVEQAGFYSHKTAQGIVRTYVLPLKDYQIQVSQQERVREAFAWELAGSMFIPYLIILPFAIFAL**

5 15953 100.0% 99.8% **YKDQAHLSHHLHLLVPPVEQAGFYSHKTAQGIVRTYVLPLKDYQIQVSQQERVREAFAWELAGSMFIPYLIILPFAIFAL**

6 15946 100.0% 99.8% **YKDQAHLSHHLHLLVPPVEQAGFYSHKTAQGIVRTYVLPLKDYQIQVSQQERVREAFAWELAGSMFIPYLIILPFAIFAL**

7 Survcare112 100.0% 99.8% **YKDQAHLSHHLHLLVPPVEQAGFYSHKTAQGIVRTYVLPLKDYQIQVSQQERVREAFAWELAGSMFIPYLIILPFAIFAL**

8 Cl300 100.0% 99.8% **YKDQAHLSHHLHLLVPPVEQAGFYSHKTAQGIVRTYVLPLKDYQIQVSQQERVREAFAWELAGSMFIPYLIILPFAIFAL**

9 17A1955 100.0% 99.8% **YKDQAHLSHHLHLLVPPVEQAGFYSHKTAQGIVRTYVLPLKDYQIQVSQQERVREAFAWELAGSMFIPYLIILPFAIFAL**

10 ACMH-6200 100.0% 99.8% **YKDQAHLSHHLHLLVPPVEQAGFYSHKTAQGIVRTYVLPLKDYQIQVSQQERVREAFAWELAGSMFIPYLIILPFAIFAL**

11 ACMH-6201 100.0% 99.8% **YKDQAHLSHHLHLLVPPVEQAGFYSHKTAQGIVRTYVLPLKDYQIQVSQQERVREAFAWELAGSMFIPYLIILPFAIFAL**

12 MIN-015 100.0% 99.8% **YKDQAHLSHHLHLLVPPVEQAGFYSHKTAQGIVRTYVLPLKDYQIQVSQQERVREAFAWELAGSMFIPYLIILPFAIFAL**

13 AB177-VUB 100.0% 99.8% **YKDQAHLSHHLHLLVPPVEQAGFYSHKTAQGIVRTYVLPLKDYQIQVSQQERVREAFAWELAGSMFIPYLIILPFAIFAL**

14 P116A 100.0% 99.8% **YKDQAHLSHHLHLLVPPVEQAGFYSHKTAQGIVRTYVLPLKDYQIQVSQQERVREAFAWELAGSMFIPYLIILPFAIFAL**

15 26 100.0% 99.8% **YKDQAHLSHHLHLLVPPVEQAGFYSHKTAQGIVRTYVLPLKDYQIQVSQQERVREAFAWELAGSMFIPYLIILPFAIFAL**

16 AR_0037 100.0% 99.8% **YKDQAHLSHHLHLLVPPVEQAGFYSHKTAQGIVRTYVLPLKDYQIQVSQQERVREAFAWELAGSMFIPYLIILPFAIFAL**

17 AR_0033 100.0% 99.8% **YKDQAHLSHHLHLLVPPVEQAGFYSHKTAQGIVRTYVLPLKDYQIQVSQQERVREAFAWELAGSMFIPYLIILPFAIFAL**

18 AB-A 100.0% 99.8% **YKDQAHLSHHLHLLVPPVEQAGFYSHKTAQGIVRTYVLPLKDYQIQVSQQERVREAFAWELAGSMFIPYLIILPFAIFAL**

19 AB-C 100.0% 99.8% **YKDQAHLSHHLHLLVPPVEQAGFYSHKTAQGIVRTYVLPLKDYQIQVSQQERVREAFAWELAGSMFIPYLIILPFAIFAL**

20 AB-B 100.0% 99.8% **YKDQAHLSHHLHLLVPPVEQAGFYSHKTAQGIVRTYVLPLKDYQIQVSQQERVREAFAWELAGSMFIPYLIILPFAIFAL**

21 AE27M 100.0% 99.8% **YKDQAHLSHHLHLLVPPVEQAGFYSHKTAQGIVRTYVLPLKDYQIQVSQQERVREAFAWELAGSMFIPYLIILPFAIFAL**

22 AE3M 100.0% 99.8% **YKDQAHLSHHLHLLVPPVEQAGFYSHKTAQGIVRTYVLPLKDYQIQVSQQERVREAFAWELAGSMFIPYLIILPFAIFAL**

23 MBL_M1 100.0% 99.8% **YKDQAHLSHHLHLLVPPVEQAGFYSHKTAQGIVRTYVLPLKDYQIQVSQQERVREAFAWELAGSMFIPYLIILPFAIFAL**

24 MBL_M6 100.0% 99.8% **YKDQAHLSHHLHLLVPPVEQAGFYSHKTAQGIVRTYVLPLKDYQIQVSQQERVREAFAWELAGSMFIPYLIILPFAIFAL**

25 MBL_M10 100.0% 99.8% **YKDQAHLSHHLHLLVPPVEQAGFYSHKTAQGIVRTYVLPLKDYQIQVSQQERVREAFAWELAGSMFIPYLIILPFAIFAL**

26 MBL_M9 100.0% 99.8% **YKDQAHLSHHLHLLVPPVEQAGFYSHKTAQGIVRTYVLPLKDYQIQVSQQERVREAFAWELAGSMFIPYLIILPFAIFAL**

27 R11 100.0% 99.8% **YKDQAHLSHHLHLLVPPVEQAGFYSHKTAQGIVRTYVLPLKDYQIQVSQQERVREAFAWELAGSMFIPYLIILPFAIFAL**

28 Ab-NDM-1 100.0% 99.5% **YKDQAHLSHHLHLLVPPVEQAGFYSHKTAQGIVRTYVLPLKDYQIQVSQQERVREAFAWELAGSMFIPYLIILPFAIFAL**

29 MRSN15574 100.0% 99.8% **YKDQAHLSHHLHLLVPPVEQAGFYSHKTAQGIVRTYVLPLKDYQIQVSQQERVREAFAWELAGSMFIPYLIILPFAIFAL**

cov pid **161**  **. . . 2 . . . .** **240**

1 ATCC19606 100.0% 100.0% **AAIIRRGLKPIDDFKNELKERDSEELTPIEVHDYPQELLPTIDEMNRLFERISKAQNEQKQFIADAAHELRTPVTALNLQ**

2 M02 100.0% 99.8% **AAIIRRGLKPIDDFKNELKERDSEELTPIEVHDYPQELLPTIDEMNRLFERISKAQNEQKQFIADAAHELRTPVTALNLQ**

3 M11 100.0% 99.8% **AAIIRRGLKPIDDFKNELKERDSEELTPIEVHDYPQELLPTIDEMNRLFERISKAQNEQKQFIADAAHELRTPVTALNLQ**

4 M18 100.0% 99.8% **AAIIRRGLKPIDDFKNELKERDSEELTPIEVHDYPQELLPTIDEMNRLFERISKAQNEQKQFIADAAHELRTPVTALNLQ**

5 15953 100.0% 99.8% **AAIIRRGLKPIDDFKNELKERDSEELTPIEVHDYPQELLPTIDEMNRLFERISKAQNEQKQFIADAAHELRTPVTALNLQ**

6 15946 100.0% 99.8% **AAIIRRGLKPIDDFKNELKERDSEELTPIEVHDYPQELLPTIDEMNRLFERISKAQNEQKQFIADAAHELRTPVTALNLQ**

7 Survcare112 100.0% 99.8% **AAIIRRGLKPIDDFKNELKERDSEELTPIEVHDYPQELLPTIDEMNRLFERISKAQNEQKQFIADAAHELRTPVTALNLQ**

8 Cl300 100.0% 99.8% **AAIIRRGLKPIDDFKNELKERDSEELTPIEVHDYPQELLPTIDEMNRLFERISKAQNEQKQFIADAAHELRTPVTALNLQ**

9 17A1955 100.0% 99.8% **AAIIRRGLKPIDDFKNELKERDSEELTPIEVHDYPQELLPTIDEMNRLFERISKAQNEQKQFIADAAHELRTPVTALNLQ**

10 ACMH-6200 100.0% 99.8% **AAIIRRGLKPIDDFKNELKERDSEELTPIEVHDYPQELLPTIDEMNRLFERISKAQNEQKQFIADAAHELRTPVTALNLQ**

11 ACMH-6201 100.0% 99.8% **AAIIRRGLKPIDDFKNELKERDSEELTPIEVHDYPQELLPTIDEMNRLFERISKAQNEQKQFIADAAHELRTPVTALNLQ**

12 MIN-015 100.0% 99.8% **AAIIRRGLKPIDDFKNELKERDSEELTPIEVHDYPQELLPTIDEMNRLFERISKAQNEQKQFIADAAHELRTPVTALNLQ**

13 AB177-VUB 100.0% 99.8% **AAIIRRGLKPIDDFKNELKERDSEELTPIEVHDYPQELLPTIDEMNRLFERISKAQNEQKQFIADAAHELRTPVTALNLQ**

14 P116A 100.0% 99.8% **AAIIRRGLKPIDDFKNELKERDSEELTPIEVHDYPQELLPTIDEMNRLFERISKAQNEQKQFIADAAHELRTPVTALNLQ**

15 26 100.0% 99.8% **AAIIRRGLKPIDDFKNELKERDSEELTPIEVHDYPQELLPTIDEMNRLFERISKAQNEQKQFIADAAHELRTPVTALNLQ**

16 AR_0037 100.0% 99.8% **AAIIRRGLKPIDDFKNELKERDSEELTPIEVHDYPQELLPTIDEMNRLFERISKAQNEQKQFIADAAHELRTPVTALNLQ**

17 AR_0033 100.0% 99.8% **AAIIRRGLKPIDDFKNELKERDSEELTPIEVHDYPQELLPTIDEMNRLFERISKAQNEQKQFIADAAHELRTPVTALNLQ**

18 AB-A 100.0% 99.8% **AAIIRRGLKPIDDFKNELKERDSEELTPIEVHDYPQELLPTIDEMNRLFERISKAQNEQKQFIADAAHELRTPVTALNLQ**

19 AB-C 100.0% 99.8% **AAIIRRGLKPIDDFKNELKERDSEELTPIEVHDYPQELLPTIDEMNRLFERISKAQNEQKQFIADAAHELRTPVTALNLQ**

20 AB-B 100.0% 99.8% **AAIIRRGLKPIDDFKNELKERDSEELTPIEVHDYPQELLPTIDEMNRLFERISKAQNEQKQFIADAAHELRTPVTALNLQ**

21 AE27M 100.0% 99.8% **AAIIRRGLKPIDDFKNELKERDSEELTPIEVHDYPQELLPTIDEMNRLFERISKAQNEQKQFIADAAHELRTPVTALNLQ**

22 AE3M 100.0% 99.8% **AAIIRRGLKPIDDFKNELKERDSEELTPIEVHDYPQELLPTIDEMNRLFERISKAQNEQKQFIADAAHELRTPVTALNLQ**

23 MBL_M1 100.0% 99.8% **AAIIRRGLKPIDDFKNELKERDSEELTPIEVHDYPQELLPTIDEMNRLFERISKAQNEQKQFIADAAHELRTPVTALNLQ**

24 MBL_M6 100.0% 99.8% **AAIIRRGLKPIDDFKNELKERDSEELTPIEVHDYPQELLPTIDEMNRLFERISKAQNEQKQFIADAAHELRTPVTALNLQ**

25 MBL_M10 100.0% 99.8% **AAIIRRGLKPIDDFKNELKERDSEELTPIEVHDYPQELLPTIDEMNRLFERISKAQNEQKQFIADAAHELRTPVTALNLQ**

26 MBL_M9 100.0% 99.8% **AAIIRRGLKPIDDFKNELKERDSEELTPIEVHDYPQELLPTIDEMNRLFERISKAQNEQKQFIADAAHELRTPVTALNLQ**

27 R11 100.0% 99.8% **AAIIRRGLKPIDDFKNELKERDSEELTPIEVHDYPQELLPTIDEMNRLFERISKAQNEQKQFIADAAHELRTPVTALNLQ**

28 Ab-NDM-1 100.0% 99.5% **AAIIRRGLKPIDDFKNELKERDSEELPPIEVHDYPQELLPTIDEMNRLFERISKAQNEQKQFIADAAHELRTPVTALNLQ**

29 MRSN15574 100.0% 99.8% **AAIIRRGLKPIDDFKNELKERDSEELTPIEVHDYPQELLPTIDEMNRLFERISKAQNEQKQFIADAAHELRTPVTALNLQ**

cov pid **241**  **: . . . . 3 . .** **320**

1 ATCC19606 100.0% 100.0% **TKILLSQFPEHESLQNLSKGLARIQHLVTQLLALAKQDVTLSMVEPTGYFQLNDVALNCVEQLVNLAMQKEIDLGFVRNE**

2 M02 100.0% 99.8% **TKILLSQFPEHESLQNLSKGLARIQHLVTQLLALAKQDVTLSMVEPTGYFQLNDVALNCVEQLVNLAMQKEIDLGFVRNE**

3 M11 100.0% 99.8% **TKILLSQFPEHESLQNLSKGLARIQHLVTQLLALAKQDVTLSMVEPTGYFQLNDVALNCVEQLVNLAMQKEIDLGFVRNE**

4 M18 100.0% 99.8% **TKILLSQFPEHESLQNLSKGLARIQHLVTQLLALAKQDVTLSMVEPTGYFQLNDVALNCVEQLVNLAMQKEIDLGFVRNE**

5 15953 100.0% 99.8% **TKILLSQFPEHESLQNLSKGLARIQHLVTQLLALAKQDVTLSMVEPTGYFQLNDVALNCVEQLVNLAMQKEIDLGFVRNE**

6 15946 100.0% 99.8% **TKILLSQFPEHESLQNLSKGLARIQHLVTQLLALAKQDVTLSMVEPTGYFQLNDVALNCVEQLVNLAMQKEIDLGFVRNE**

7 Survcare112 100.0% 99.8% **TKILLSQFPEHESLQNLSKGLARIQHLVTQLLALAKQDVTLSMVEPTGYFQLNDVALNCVEQLVNLAMQKEIDLGFVRNE**

8 Cl300 100.0% 99.8% **TKILLSQFPEHESLQNLSKGLARIQHLVTQLLALAKQDVTLSMVEPTGYFQLNDVALNCVEQLVNLAMQKEIDLGFVRNE**

9 17A1955 100.0% 99.8% **TKILLSQFPEHESLQNLSKGLARIQHLVTQLLALAKQDVTLSMVEPTGYFQLNDVALNCVEQLVNLAMQKEIDLGFVRNE**

10 ACMH-6200 100.0% 99.8% **TKILLSQFPEHESLQNLSKGLARIQHLVTQLLALAKQDVTLSMVEPTGYFQLNDVALNCVEQLVNLAMQKEIDLGFVRNE**

11 ACMH-6201 100.0% 99.8% **TKILLSQFPEHESLQNLSKGLARIQHLVTQLLALAKQDVTLSMVEPTGYFQLNDVALNCVEQLVNLAMQKEIDLGFVRNE**

12 MIN-015 100.0% 99.8% **TKILLSQFPEHESLQNLSKGLARIQHLVTQLLALAKQDVTLSMVEPTGYFQLNDVALNCVEQLVNLAMQKEIDLGFVRNE**

13 AB177-VUB 100.0% 99.8% **TKILLSQFPEHESLQNLSKGLARIQHLVTQLLALAKQDVTLSMVEPTGYFQLNDVALNCVEQLVNLAMQKEIDLGFVRNE**

14 P116A 100.0% 99.8% **TKILLSQFPEHESLQNLSKGLARIQHLVTQLLALAKQDVTLSMVEPTGYFQLNDVALNCVEQLVNLAMQKEIDLGFVRNE**

15 26 100.0% 99.8% **TKILLSQFPEHESLQNLSKGLARIQHLVTQLLALAKQDVTLSMVEPTGYFQLNDVALNCVEQLVNLAMQKEIDLGFVRNE**

16 AR_0037 100.0% 99.8% **TKILLSQFPEHESLQNLSKGLARIQHLVTQLLALAKQDVTLSMVEPTGYFQLNDVALNCVEQLVNLAMQKEIDLGFVRNE**

17 AR_0033 100.0% 99.8% **TKILLSQFPEHESLQNLSKGLARIQHLVTQLLALAKQDVTLSMVEPTGYFQLNDVALNCVEQLVNLAMQKEIDLGFVRNE**

18 AB-A 100.0% 99.8% **TKILLSQFPEHESLQNLSKGLARIQHLVTQLLALAKQDVTLSMVEPTGYFQLNDVALNCVEQLVNLAMQKEIDLGFVRNE**

19 AB-C 100.0% 99.8% **TKILLSQFPEHESLQNLSKGLARIQHLVTQLLALAKQDVTLSMVEPTGYFQLNDVALNCVEQLVNLAMQKEIDLGFVRNE**

20 AB-B 100.0% 99.8% **TKILLSQFPEHESLQNLSKGLARIQHLVTQLLALAKQDVTLSMVEPTGYFQLNDVALNCVEQLVNLAMQKEIDLGFVRNE**

21 AE27M 100.0% 99.8% **TKILLSQFPEHESLQNLSKGLARIQHLVTQLLALAKQDVTLSMVEPTGYFQLNDVALNCVEQLVNLAMQKEIDLGFVRNE**

22 AE3M 100.0% 99.8% **TKILLSQFPEHESLQNLSKGLARIQHLVTQLLALAKQDVTLSMVEPTGYFQLNDVALNCVEQLVNLAMQKEIDLGFVRNE**

23 MBL_M1 100.0% 99.8% **TKILLSQFPEHESLQNLSKGLARIQHLVTQLLALAKQDVTLSMVEPTGYFQLNDVALNCVEQLVNLAMQKEIDLGFVRNE**

24 MBL_M6 100.0% 99.8% **TKILLSQFPEHESLQNLSKGLARIQHLVTQLLALAKQDVTLSMVEPTGYFQLNDVALNCVEQLVNLAMQKEIDLGFVRNE**

25 MBL_M10 100.0% 99.8% **TKILLSQFPEHESLQNLSKGLARIQHLVTQLLALAKQDVTLSMVEPTGYFQLNDVALNCVEQLVNLAMQKEIDLGFVRNE**

26 MBL_M9 100.0% 99.8% **TKILLSQFPEHESLQNLSKGLARIQHLVTQLLALAKQDVTLSMVEPTGYFQLNDVALNCVEQLVNLAMQKEIDLGFVRNE**

27 R11 100.0% 99.8% **TKILLSQFPEHESLQNLSKGLARIQHLVTQLLALAKQDVTLSMVEPTGYFQLNDVALNCVEQLVNLAMQKEIDLGFVRNE**

28 Ab-NDM-1 100.0% 99.5% **TKILLSQFPEHESLQNLSKGLARIQHLVTQLLALAKQDVTLSMVEPTGYFQLNDVALNCVEQLVNLAMQKEIDLGFVRNE**

29 MRSN15574 100.0% 99.8% **TKILLSQFPEHESLQNLSKGLARIQHLVTQLLALAKQDVTLSMVEPTGYFQLNDVALNCVEQLVNLAMQKEIDLGFVRNE**

cov pid **321**  **. . : . . . . 4** **400**

1 ATCC19606 100.0% 100.0% **PIEMHSIEPTVHSIIFNLIDNAIKYTPHQGVINISVYTDPDHYACIQIEDSGAGIDPENYDKVLKRFYRVHHHLEVGSGL**

2 M02 100.0% 99.8% **PIEMHSIEPTVHSIIFNLIDNAIKYTPHQGVINISVYTDPDHYACIQIEDSGAGIDPENYDKVLKRFYRVHHHLEVGSGL**

3 M11 100.0% 99.8% **PIEMHSIEPTVHSIIFNLIDNAIKYTPHQGVINISVYTDPDHYACIQIEDSGAGIDPENYDKVLKRFYRVHHHLEVGSGL**

4 M18 100.0% 99.8% **PIEMHSIEPTVHSIIFNLIDNAIKYTPHQGVINISVYTDPDHYACIQIEDSGAGIDPENYDKVLKRFYRVHHHLEVGSGL**

5 15953 100.0% 99.8% **PIEMHSIEPTVHSIIFNLIDNAIKYTPHQGVINISVYTDPDHYACIQIEDSGAGIDPENYDKVLKRFYRVHHHLEVGSGL**

6 15946 100.0% 99.8% **PIEMHSIEPTVHSIIFNLIDNAIKYTPHQGVINISVYTDPDHYACIQIEDSGAGIDPENYDKVLKRFYRVHHHLEVGSGL**

7 Survcare112 100.0% 99.8% **PIEMHSIEPTVHSIIFNLIDNAIKYTPHQGVINISVYTDPDHYACIQIEDSGAGIDPENYDKVLKRFYRVHHHLEVGSGL**

8 Cl300 100.0% 99.8% **PIEMHSIEPTVHSIIFNLIDNAIKYTPHQGVINISVYTDPDHYACIQIEDSGAGIDPENYDKVLKRFYRVHHHLEVGSGL**

9 17A1955 100.0% 99.8% **PIEMHSIEPTVHSIIFNLIDNAIKYTPHQGVINISVYTDPDHYACIQIEDSGAGIDPENYDKVLKRFYRVHHHLEVGSGL**

10 ACMH-6200 100.0% 99.8% **PIEMHSIEPTVHSIIFNLIDNAIKYTPHQGVINISVYTDPDHYACIQIEDSGAGIDPENYDKVLKRFYRVHHHLEVGSGL**

11 ACMH-6201 100.0% 99.8% **PIEMHSIEPTVHSIIFNLIDNAIKYTPHQGVINISVYTDPDHYACIQIEDSGAGIDPENYDKVLKRFYRVHHHLEVGSGL**

12 MIN-015 100.0% 99.8% **PIEMHSIEPTVHSIIFNLIDNAIKYTPHQGVINISVYTDPDHYACIQIEDSGAGIDPENYDKVLKRFYRVHHHLEVGSGL**

13 AB177-VUB 100.0% 99.8% **PIEMHSIEPTVHSIIFNLIDNAIKYTPHQGVINISVYTDPDHYACIQIEDSGAGIDPENYDKVLKRFYRVHHHLEVGSGL**

14 P116A 100.0% 99.8% **PIEMHSIEPTVHSIIFNLIDNAIKYTPHQGVINISVYTDPDHYACIQIEDSGAGIDPENYDKVLKRFYRVHHHLEVGSGL**

15 26 100.0% 99.8% **PIEMHSIEPTVHSIIFNLIDNAIKYTPHQGVINISVYTDPDHYACIQIEDSGAGIDPENYDKVLKRFYRVHHHLEVGSGL**

16 AR_0037 100.0% 99.8% **PIEMHSIEPTVHSIIFNLIDNAIKYTPHQGVINISVYTDPDHYACIQIEDSGAGIDPENYDKVLKRFYRVHHHLEVGSGL**

17 AR_0033 100.0% 99.8% **PIEMHSIEPTVHSIIFNLIDNAIKYTPHQGVINISVYTDPDHYACIQIEDSGAGIDPENYDKVLKRFYRVHHHLEVGSGL**

18 AB-A 100.0% 99.8% **PIEMHSIEPTVHSIIFNLIDNAIKYTPHQGVINISVYTDPDHYACIQIEDSGAGIDPENYDKVLKRFYRVHHHLEVGSGL**

19 AB-C 100.0% 99.8% **PIEMHSIEPTVHSIIFNLIDNAIKYTPHQGVINISVYTDPDHYACIQIEDSGAGIDPENYDKVLKRFYRVHHHLEVGSGL**

20 AB-B 100.0% 99.8% **PIEMHSIEPTVHSIIFNLIDNAIKYTPHQGVINISVYTDPDHYACIQIEDSGAGIDPENYDKVLKRFYRVHHHLEVGSGL**

21 AE27M 100.0% 99.8% **PIEMHSIEPTVHSIIFNLIDNAIKYTPHQGVINISVYTDPDHYACIQIEDSGAGIDPENYDKVLKRFYRVHHHLEVGSGL**

22 AE3M 100.0% 99.8% **PIEMHSIEPTVHSIIFNLIDNAIKYTPHQGVINISVYTDPDHYACIQIEDSGAGIDPENYDKVLKRFYRVHHHLEVGSGL**

23 MBL_M1 100.0% 99.8% **PIEMHSIEPTVHSIIFNLIDNAIKYTPHQGVINISVYTDPDHYACIQIEDSGAGIDPENYDKVLKRFYRVHHHLEVGSGL**

24 MBL_M6 100.0% 99.8% **PIEMHSIEPTVHSIIFNLIDNAIKYTPHQGVINISVYTDPDHYACIQIEDSGAGIDPENYDKVLKRFYRVHHHLEVGSGL**

25 MBL_M10 100.0% 99.8% **PIEMHSIEPTVHSIIFNLIDNAIKYTPHQGVINISVYTDPDHYACIQIEDSGAGIDPENYDKVLKRFYRVHHHLEVGSGL**

26 MBL_M9 100.0% 99.8% **PIEMHSIEPTVHSIIFNLIDNAIKYTPHQGVINISVYTDPDHYACIQIEDSGAGIDPENYDKVLKRFYRVHHHLEVGSGL**

27 R11 100.0% 99.8% **PIEMHSIEPTVHSIIFNLIDNAIKYTPHQGVINISVYTDPDHYACIQIEDSGAGIDPENYDKVLKRFYRVHHHLEVGSGL**

28 Ab-NDM-1 100.0% 99.5% **PIEMHSIEPTVHSIIFNLIDNAIKYTPHQGVINISVYTDPDHYACIQIEDSGAGIDPENYDKVLKRFYRVHHHLEVGSGL**

29 MRSN15574 100.0% 99.8% **PIEMHSIEPTVHSIIFNLIDNAIKYTPHQGVINISVYTDPDHYACIQIEDSGAGIDPENYDKVLKRFYRVHHHLEVGSGL**

cov pid **401**  **. . . . ]** **444**

1 ATCC19606 100.0% 100.0% **GLSIVDRATQRLGGTLTLDKSLELGGLSVLVKLPKVLHLNETRA**

2 M02 100.0% 99.8% **GLSIVDRATQRLGGTLTLDKSLELGGLSVLVKLPKVLHLHETRA**

3 M11 100.0% 99.8% **GLSIVDRATQRLGGTLTLDKSLELGGLSVLVKLPKVLHLHETRA**

4 M18 100.0% 99.8% **GLSIVDRATQRLGGTLTLDKSLELGGLSVLVKLPKVLHLHETRA**

5 15953 100.0% 99.8% **GLSIVDRATQRLGGTLTLDKSLELGGLSVLVKLPKVLHLHETRA**

6 15946 100.0% 99.8% **GLSIVDRATQRLGGTLTLDKSLELGGLSVLVKLPKVLHLHETRA**

7 Survcare112 100.0% 99.8% **GLSIVDRATQRLGGTLTLDKSLELGGLSVLVKLPKVLHLHETRA**

8 Cl300 100.0% 99.8% **GLSIVDRATQRLGGTLTLDKSLELGGLSVLVKLPKVLHLHETRA**

9 17A1955 100.0% 99.8% **GLSIVDRATQRLGGTLTLDKSLELGGLSVLVKLPKVLHLHETRA**

10 ACMH-6200 100.0% 99.8% **GLSIVDRATQRLGGTLTLDKSLELGGLSVLVKLPKVLHLHETRA**

11 ACMH-6201 100.0% 99.8% **GLSIVDRATQRLGGTLTLDKSLELGGLSVLVKLPKVLHLHETRA**

12 MIN-015 100.0% 99.8% **GLSIVDRATQRLGGTLTLDKSLELGGLSVLVKLPKVLHLHETRA**

13 AB177-VUB 100.0% 99.8% **GLSIVDRATQRLGGTLTLDKSLELGGLSVLVKLPKVLHLHETRA**

14 P116A 100.0% 99.8% **GLSIVDRATQRLGGTLTLDKSLELGGLSVLVKLPKVLHLHETRA**

15 26 100.0% 99.8% **GLSIVDRATQRLGGTLTLDKSLELGGLSVLVKLPKVLHLHETRA**

16 AR_0037 100.0% 99.8% **GLSIVDRATQRLGGTLTLDKSLELGGLSVLVKLPKVLHLHETRA**

17 AR_0033 100.0% 99.8% **GLSIVDRATQRLGGTLTLDKSLELGGLSVLVKLPKVLHLHETRA**

18 AB-A 100.0% 99.8% **GLSIVDRATQRLGGTLTLDKSLELGGLSVLVKLPKVLHLHETRA**

19 AB-C 100.0% 99.8% **GLSIVDRATQRLGGTLTLDKSLELGGLSVLVKLPKVLHLHETRA**

20 AB-B 100.0% 99.8% **GLSIVDRATQRLGGTLTLDKSLELGGLSVLVKLPKVLHLHETRA**

21 AE27M 100.0% 99.8% **GLSIVDRATQRLGGTLTLDKSLELGGLSVLVKLPKVLHLHETRA**

22 AE3M 100.0% 99.8% **GLSIVDRATQRLGGTLTLDKSLELGGLSVLVKLPKVLHLHETRA**

23 MBL_M1 100.0% 99.8% **GLSIVDRATQRLGGTLTLDKSLELGGLSVLVKLPKVLHLHETRA**

24 MBL_M6 100.0% 99.8% **GLSIVDRATQRLGGTLTLDKSLELGGLSVLVKLPKVLHLHETRA**

25 MBL_M10 100.0% 99.8% **GLSIVDRATQRLGGTLTLDKSLELGGLSVLVKLPKVLHLHETRA**

26 MBL_M9 100.0% 99.8% **GLSIVDRATQRLGGTLTLDKSLELGGLSVLVKLPKVLHLHETRA**

27 R11 100.0% 99.8% **GLSIVDRATQRLGGTLTLDKSLELGGLSVLVKLPKVLHLHETRA**

28 Ab-NDM-1 100.0% 99.5% **GLSIVDRATQRLGGTLTLDKSLELGGLSVLVKLPKVLHLHETRA**

29 MRSN15574 100.0% 99.8% **GLSIVDRATQRLGGTLTLDKSLELGGLSVLVKLPKVLHLHETRA**

**Supplementary Figure 9: Multiple sequence alignment (MSA) of the predicted amino acid sequence of PmrB carried by ST85^Pas^ (GC9) isolates and close genomes retrieved from the BV-BRC database compared to the respective gene in *A. baumannii* ATCC19606.** MSA was created by the A multiple alignment viewer MView hosted by the EMBL-EBI; cov, coverage; pid, percent identity.

cov pid  **1** **[ . . . . : . . .** **80**

1 ATCC19606 100.0% 100.0% **MHYSLKKRLIWGTSIFSVILGCILIFSAYKVALQEVDEILDTQMKYLAERTAEHPLKTVSSKFDFHKTYHEEDLFIDIWA**

2 M19 74.5% 99.7% **MHYSLKKRLIWGTSIFSVILGCILIFSAYKVALQEVDEILDTQMKYLAERTAEHPLKTVSSKFDFHKTYHEEDLFIDIWA**

3 M19b 21.2% 93.6% **--------------------------------------------------------------------------------**

4 M10 66.4% 99.7% **--------------------------------------------------------------------------------**

5 A5 100.0% 99.8% **MHYSLKKRLIWGTSIFSVILGCILIFSAYKVALQEVDEILDTQMKYLAERTAEHPLKTVSSKFDFHKTYHEEDLFIDIWA**

6 Aci00709 100.0% 99.8% **MHYSLKKRLIWGTSIFSVILGCILIFSAYKVALQEVDEILDTQMKYLAERTAEHPLKTVSSKFDFHKTYHEEDLFIDIWA**

7 Aci00711 100.0% 99.8% **MHYSLKKRLIWGTSIFSVILGCILIFSAYKVALQEVDEILDTQMKYLAERTAEHPLKTVSSKFDFHKTYHEEDLFIDIWA**

8 KAB15 100.0% 99.8% **MHYSLKKRLIWGTSIFSVILGCILIFSAYKVALQEVDEILDTQMKYLAERTAEHPLKTVSSKFDFHKTYHEEDLFIDIWA**

9 PT022 100.0% 99.8% **MHYSLKKRLIWGTSIFSVILGCILIFSAYKVALQEVDEILDTQMKYLAERTAEHPLKTVSSKFDFHKTYHEEDLFIDIWA**

10 PSU091 100.0% 99.8% **MHYSLKKRLIWGTSIFSVILGCILIFSAYKVALQEVDEILDTQMKYLAERTAEHPLKTVSSKFDFHKTYHEEDLFIDIWA**

11 151 100.0% 99.5% **MHYSLKKRLIWGTSIFSVILGCILIFSAYKVALQEVDEILDTQMKYLAERTAEHPLKTVSSKFDFHKTYHEEDLFIDIWA**

12 GML-KP48-AB-TR 100.0% 99.3% **MHYSLKKRLIWGTSIFSVILGCILIFSAYKVALQEVDEILDTQMKYLAERTAEHPLKTVSSKFDFHKTYHEEDLFIDIWA**

13 198 100.0% 99.5% **MHYSLKKRLIWGTSIFSVILGCILIFSAYKVALQEVDEILDTQMKYLAERTAEHPLKTVSSKFDFHKTYHEEDLFIDIWA**

14 4300STDY7045706 100.0% 99.8% **MHYSLKKRLIWGTSIFSVILGCILIFSAYKVALQEVDEILDTQMKYLAERTAEHPLKTVSSKFDFHKTYHEEDLFIDIWA**

15 4300STDY7045763 100.0% 99.8% **MHYSLKKRLIWGTSIFSVILGCILIFSAYKVALQEVDEILDTQMKYLAERTAEHPLKTVSSKFDFHKTYHEEDLFIDIWA**

16 4300STDY7045798 100.0% 99.8% **MHYSLKKRLIWGTSIFSVILGCILIFSAYKVALQEVDEILDTQMKYLAERTAEHPLKTVSSKFDFHKTYHEEDLFIDIWA**

17 4300STDY7045799 100.0% 99.8% **MHYSLKKRLIWGTSIFSVILGCILIFSAYKVALQEVDEILDTQMKYLAERTAEHPLKTVSSKFDFHKTYHEEDLFIDIWA**

18 4300STDY7045808 100.0% 99.8% **MHYSLKKRLIWGTSIFSVILGCILIFSAYKVALQEVDEILDTQMKYLAERTAEHPLKTVSSKFDFHKTYHEEDLFIDIWA**

19 4300STDY7045806 100.0% 99.8% **MHYSLKKRLIWGTSIFSVILGCILIFSAYKVALQEVDEILDTQMKYLAERTAEHPLKTVSSKFDFHKTYHEEDLFIDIWA**

20 4300STDY7045811 100.0% 99.8% **MHYSLKKRLIWGTSIFSVILGCILIFSAYKVALQEVDEILDTQMKYLAERTAEHPLKTVSSKFDFHKTYHEEDLFIDIWA**

21 4300STDY7045829 100.0% 99.8% **MHYSLKKRLIWGTSIFSVILGCILIFSAYKVALQEVDEILDTQMKYLAERTAEHPLKTVSSKFDFHKTYHEEDLFIDIWA**

22 4300STDY7045870 100.0% 99.8% **MHYSLKKRLIWGTSIFSVILGCILIFSAYKVALQEVDEILDTQMKYLAERTAEHPLKTVSSKFDFHKTYHEEDLFIDIWA**

23 4300STDY7045866 100.0% 99.8% **MHYSLKKRLIWGTSIFSVILGCILIFSAYKVALQEVDEILDTQMKYLAERTAEHPLKTVSSKFDFHKTYHEEDLFIDIWA**

cov pid  **81**  **. 1 . . . . : .** **160**

1 ATCC19606 100.0% 100.0% **YKDQAHLSHHLHLLVPPVEQAGFYSHKTAQGIVRTYVLPLKDYQIQVSQQERVREAFAWELAGSMFIPYLIILPFAIFAL**

2 M19 74.5% 99.7% **YKDQAHLSLHLHLLVPPVEQAGFYSHKTAQGIVRTYVLPLKDYQIQVSQQERVREAFAWELAGSMFIPYLIILPFAIFAL**

3 M19b 21.2% 93.6% **--------------------------------------------------------------------------------**

4 M10 66.4% 99.7% **---------------------------------------------------------------------LIILPFAIFAL**

5 A5 100.0% 99.8% **YKDQAHLSHHLHLLVPPVEQAGFYSHKTAQGIVRTYVLPLKDYQIQVSQQERVREAFAWELAGSMFIPYLIILPFAIFAL**

6 Aci00709 100.0% 99.8% **YKDQAHLSHHLHLLVPPVEQAGFYSHKTAQGIVRTYVLPLKDYQIQVSQQERVREAFAWELAGSMFIPYLIILPFAIFAL**

7 Aci00711 100.0% 99.8% **YKDQAHLSHHLHLLVPPVEQAGFYSHKTAQGIVRTYVLPLKDYQIQVSQQERVREAFAWELAGSMFIPYLIILPFAIFAL**

8 KAB15 100.0% 99.8% **YKDQAHLSHHLHLLVPPVEQAGFYSHKTAQGIVRTYVLPLKDYQIQVSQQERVREAFAWELAGSMFIPYLIILPFAIFAL**

9 PT022 100.0% 99.8% **YKDQAHLSHHLHLLVPPVEQAGFYSHKTAQGIVRTYVLPLKDYQIQVSQQERVREAFAWELAGSMFIPYLIILPFAIFAL**

10 PSU091 100.0% 99.8% **YKDQAHLSHHLHLLVPPVEQAGFYSHKTAQGIVRTYVLPLKDYQIQVSQQERVREAFAWELAGSMFIPYLIILPFAIFAL**

11 151 100.0% 99.5% **YKDQAHLSLHLHLLVPPVEQAGFYSHKTAQGIVRTYVLPLKDYQIQVSQQERVREAFAWELAGSMFIPYLIILPFAIFAL**

12 GML-KP48-AB-TR 100.0% 99.3% **YKDQAHLSLHLHLLVPPVEQAGFYSHKTAQGIVRTYVLPLKDYQIQVSQQERVREAFAWELAGSMFIPYLIILPFAIFAL**

13 198 100.0% 99.5% **YKDQAHLSLHLHLLVPPVEQAGFYSHKTAQGIVRTYVLPLKDYQIQVSQQERVREAFAWELAGSMFIPYLIILPFAIFAL**

14 4300STDY7045706 100.0% 99.8% **YKDQAHLSHHLHLLVPPVEQAGFYSHKTAQGIVRTYVLPLKDYQIQVSQQERVREAFAWELAGSMFIPYLIILPFAIFAL**

15 4300STDY7045763 100.0% 99.8% **YKDQAHLSHHLHLLVPPVEQAGFYSHKTAQGIVRTYVLPLKDYQIQVSQQERVREAFAWELAGSMFIPYLIILPFAIFAL**

16 4300STDY7045798 100.0% 99.8% **YKDQAHLSHHLHLLVPPVEQAGFYSHKTAQGIVRTYVLPLKDYQIQVSQQERVREAFAWELAGSMFIPYLIILPFAIFAL**

17 4300STDY7045799 100.0% 99.8% **YKDQAHLSHHLHLLVPPVEQAGFYSHKTAQGIVRTYVLPLKDYQIQVSQQERVREAFAWELAGSMFIPYLIILPFAIFAL**

18 4300STDY7045808 100.0% 99.8% **YKDQAHLSHHLHLLVPPVEQAGFYSHKTAQGIVRTYVLPLKDYQIQVSQQERVREAFAWELAGSMFIPYLIILPFAIFAL**

19 4300STDY7045806 100.0% 99.8% **YKDQAHLSHHLHLLVPPVEQAGFYSHKTAQGIVRTYVLPLKDYQIQVSQQERVREAFAWELAGSMFIPYLIILPFAIFAL**

20 4300STDY7045811 100.0% 99.8% **YKDQAHLSHHLHLLVPPVEQAGFYSHKTAQGIVRTYVLPLKDYQIQVSQQERVREAFAWELAGSMFIPYLIILPFAIFAL**

21 4300STDY7045829 100.0% 99.8% **YKDQAHLSHHLHLLVPPVEQAGFYSHKTAQGIVRTYVLPLKDYQIQVSQQERVREAFAWELAGSMFIPYLIILPFAIFAL**

22 4300STDY7045870 100.0% 99.8% **YKDQAHLSHHLHLLVPPVEQAGFYSHKTAQGIVRTYVLPLKDYQIQVSQQERVREAFAWELAGSMFIPYLIILPFAIFAL**

23 4300STDY7045866 100.0% 99.8% **YKDQAHLSHHLHLLVPPVEQAGFYSHKTAQGIVRTYVLPLKDYQIQVSQQERVREAFAWELAGSMFIPYLIILPFAIFAL**

cov pid **161**  **. . . 2 . . . .** **240**

1 ATCC19606 100.0% 100.0% **AAIIRRGLKPIDDFKNELKERDSEELTPIEVHDYPQELLPTIDEMNRLFERISKAQNEQKQFIADAAHELRTPVTALNLQ**

2 M19 74.5% 99.7% **AAIIRRGLKPIDDFKNELKERDSEELTPIEVHDYPQELLPTIDEMNRLFERISKAQNEQKQFIADAAHELRTPVTALNLQ**

3 M19b 21.2% 93.6% **--------------------------------------------------------------------------------**

4 M10 66.4% 99.7% **AAIIRRGLKPIDDFKNELKERDSEELTPIEVHDYPQELLPTIDEMNRLFERISKAQNEQKQFIADAAHELRTPVTALNLQ**

5 A5 100.0% 99.8% **AAIIRRGLKPIDDFKNELKERDSEELTPIEVHDYPQELLPTIDEMNRLFERISKAQNEQKQFIADAAHELRTPVTALNLQ**

6 Aci00709 100.0% 99.8% **AAIIRRGLKPIDDFKNELKERDSEELTPIEVHDYPQELLPTIDEMNRLFERISKAQNEQKQFIADAAHELRTPVTALNLQ**

7 Aci00711 100.0% 99.8% **AAIIRRGLKPIDDFKNELKERDSEELTPIEVHDYPQELLPTIDEMNRLFERISKAQNEQKQFIADAAHELRTPVTALNLQ**

8 KAB15 100.0% 99.8% **AAIIRRGLKPIDDFKNELKERDSEELTPIEVHDYPQELLPTIDEMNRLFERISKAQNEQKQFIADAAHELRTPVTALNLQ**

9 PT022 100.0% 99.8% **AAIIRRGLKPIDDFKNELKERDSEELTPIEVHDYPQELLPTIDEMNRLFERISKAQNEQKQFIADAAHELRTPVTALNLQ**

10 PSU091 100.0% 99.8% **AAIIRRGLKPIDDFKNELKERDSEELTPIEVHDYPQELLPTIDEMNRLFERISKAQNEQKQFIADAAHELRTPVTALNLQ**

11 151 100.0% 99.5% **AAIIRRGLKPIDDFKNELKERDSEELTPIEVHDYPQELLPTIDEMNRLFERISKAQNEQKQFIADAAHELRTPVTALNLQ**

12 GML-KP48-AB-TR 100.0% 99.3% **AAIIRRGLKPIDDFKNELKERDSEELTPIEVHDYPQELLPTIDEMNRLFERISKAQNEQKQFIADAAHELRTPVTALNLQ**

13 198 100.0% 99.5% **AAIIRRGLKPIDDFKNELKERDSEELTPIEVHDYPQELLPTIDEMNRLFERISKAQNEQKQFIADAAHELRTPVTALNLQ**

14 4300STDY7045706 100.0% 99.8% **AAIIRRGLKPIDDFKNELKERDSEELTPIEVHDYPQELLPTIDEMNRLFERISKAQNEQKQFIADAAHELRTPVTALNLQ**

15 4300STDY7045763 100.0% 99.8% **AAIIRRGLKPIDDFKNELKERDSEELTPIEVHDYPQELLPTIDEMNRLFERISKAQNEQKQFIADAAHELRTPVTALNLQ**

16 4300STDY7045798 100.0% 99.8% **AAIIRRGLKPIDDFKNELKERDSEELTPIEVHDYPQELLPTIDEMNRLFERISKAQNEQKQFIADAAHELRTPVTALNLQ**

17 4300STDY7045799 100.0% 99.8% **AAIIRRGLKPIDDFKNELKERDSEELTPIEVHDYPQELLPTIDEMNRLFERISKAQNEQKQFIADAAHELRTPVTALNLQ**

18 4300STDY7045808 100.0% 99.8% **AAIIRRGLKPIDDFKNELKERDSEELTPIEVHDYPQELLPTIDEMNRLFERISKAQNEQKQFIADAAHELRTPVTALNLQ**

19 4300STDY7045806 100.0% 99.8% **AAIIRRGLKPIDDFKNELKERDSEELTPIEVHDYPQELLPTIDEMNRLFERISKAQNEQKQFIADAAHELRTPVTALNLQ**

20 4300STDY7045811 100.0% 99.8% **AAIIRRGLKPIDDFKNELKERDSEELTPIEVHDYPQELLPTIDEMNRLFERISKAQNEQKQFIADAAHELRTPVTALNLQ**

21 4300STDY7045829 100.0% 99.8% **AAIIRRGLKPIDDFKNELKERDSEELTPIEVHDYPQELLPTIDEMNRLFERISKAQNEQKQFIADAAHELRTPVTALNLQ**

22 4300STDY7045870 100.0% 99.8% **AAIIRRGLKPIDDFKNELKERDSEELTPIEVHDYPQELLPTIDEMNRLFERISKAQNEQKQFIADAAHELRTPVTALNLQ**

23 4300STDY7045866 100.0% 99.8% **AAIIRRGLKPIDDFKNELKERDSEELTPIEVHDYPQELLPTIDEMNRLFERISKAQNEQKQFIADAAHELRTPVTALNLQ**

cov pid **241**  **: . . . . 3 . .** **320**

1 ATCC19606 100.0% 100.0% **TKILLSQFPEHESLQNLSKGLARIQHLVTQLLALAKQDVTLSMVEPTGYFQLNDVALNCVEQLVNLAMQKEIDLGFVRNE**

2 M19 74.5% 99.7% **TKILLSQFPEHESLQNLSKGLARIQHLVTQLLALAKQDVTLSMVEPTGYFQLNDVALNCVEQLVNLAMQKEIDLGFVRNE**

3 M19b 21.2% 93.6% **--------------------------------------------------------------------------------**

4 M10 66.4% 99.7% **TKILLSQFPEHESLQNLSKGLARIQHLVTQLLALAKQDVTLSMVEPTGYFQLNDVALNCVEQLVNLAMQKEIDLGFVRNE**

5 A5 100.0% 99.8% **TKILLSQFPEHESLQNLSKGLARIQHLVTQLLALAKQDVTLSMVEPTGYFQLNDVALNCVEQLVNLAMQKEIDLGFVRNE**

6 Aci00709 100.0% 99.8% **TKILLSQFPEHESLQNLSKGLARIQHLVTQLLALAKQDVTLSMVEPTGYFQLNDVALNCVEQLVNLAMQKEIDLGFVRNE**

7 Aci00711 100.0% 99.8% **TKILLSQFPEHESLQNLSKGLARIQHLVTQLLALAKQDVTLSMVEPTGYFQLNDVALNCVEQLVNLAMQKEIDLGFVRNE**

8 KAB15 100.0% 99.8% **TKILLSQFPEHESLQNLSKGLARIQHLVTQLLALAKQDVTLSMVEPTGYFQLNDVALNCVEQLVNLAMQKEIDLGFVRNE**

9 PT022 100.0% 99.8% **TKILLSQFPEHESLQNLSKGLARIQHLVTQLLALAKQDVTLSMVEPTGYFQLNDVALNCVEQLVNLAMQKEIDLGFVRNE**

10 PSU091 100.0% 99.8% **TKILLSQFPEHESLQNLSKGLARIQHLVTQLLALAKQDVTLSMVEPTGYFQLNDVALNCVEQLVNLAMQKEIDLGFVRNE**

11 151 100.0% 99.5% **TKILLSQFPEHESLQNLSKGLARIQHLVTQLLALAKQDVTLSMVEPTGYFQLNDVALNCVEQLVNLAMQKEIDLGFVRNE**

12 GML-KP48-AB-TR 100.0% 99.3% **TKILLSQFPEHESLQNLSKGLARIQHLVTQLLALAKQDVTLSMVEPTGYFQLNDVALNCVEQLVNLAMQKEIDLGFVRNE**

13 198 100.0% 99.5% **TKILLSQFPEHESLQNLSKGLARIQHLVTQLLALAKQDVTLSMVEPTGYFQLNDVALNCVEQLVNLAMQKEIDLGFVRNE**

14 4300STDY7045706 100.0% 99.8% **TKILLSQFPEHESLQNLSKGLARIQHLVTQLLALAKQDVTLSMVEPTGYFQLNDVALNCVEQLVNLAMQKEIDLGFVRNE**

15 4300STDY7045763 100.0% 99.8% **TKILLSQFPEHESLQNLSKGLARIQHLVTQLLALAKQDVTLSMVEPTGYFQLNDVALNCVEQLVNLAMQKEIDLGFVRNE**

16 4300STDY7045798 100.0% 99.8% **TKILLSQFPEHESLQNLSKGLARIQHLVTQLLALAKQDVTLSMVEPTGYFQLNDVALNCVEQLVNLAMQKEIDLGFVRNE**

17 4300STDY7045799 100.0% 99.8% **TKILLSQFPEHESLQNLSKGLARIQHLVTQLLALAKQDVTLSMVEPTGYFQLNDVALNCVEQLVNLAMQKEIDLGFVRNE**

18 4300STDY7045808 100.0% 99.8% **TKILLSQFPEHESLQNLSKGLARIQHLVTQLLALAKQDVTLSMVEPTGYFQLNDVALNCVEQLVNLAMQKEIDLGFVRNE**

19 4300STDY7045806 100.0% 99.8% **TKILLSQFPEHESLQNLSKGLARIQHLVTQLLALAKQDVTLSMVEPTGYFQLNDVALNCVEQLVNLAMQKEIDLGFVRNE**

20 4300STDY7045811 100.0% 99.8% **TKILLSQFPEHESLQNLSKGLARIQHLVTQLLALAKQDVTLSMVEPTGYFQLNDVALNCVEQLVNLAMQKEIDLGFVRNE**

21 4300STDY7045829 100.0% 99.8% **TKILLSQFPEHESLQNLSKGLARIQHLVTQLLALAKQDVTLSMVEPTGYFQLNDVALNCVEQLVNLAMQKEIDLGFVRNE**

22 4300STDY7045870 100.0% 99.8% **TKILLSQFPEHESLQNLSKGLARIQHLVTQLLALAKQDVTLSMVEPTGYFQLNDVALNCVEQLVNLAMQKEIDLGFVRNE**

23 4300STDY7045866 100.0% 99.8% **TKILLSQFPEHESLQNLSKGLARIQHLVTQLLALAKQDVTLSMVEPTGYFQLNDVALNCVEQLVNLAMQKEIDLGFVRNE**

cov pid **321**  **. . : . . . . 4** **400**

1 ATCC19606 100.0% 100.0% **PIEMHSIEPTVHSIIFNLIDNAIKYTPHQGVINISVYTDPDHYACIQIEDSGAGIDPENYDKVLKRFYRVHHHLEVGSGL**

2 M19 74.5% 99.7% **PIEMHSIEPTV---------------------------------------------------------------------**

3 M19b 21.2% 93.6% **------------------------------MYKRQVYTDPDHYACIQIEDSGAGIDPENYDKVLKRFYRVHHHLEVGSGL**

4 M10 66.4% 99.7% **PIEMHSIEPTVHSIIFNLIDNAIKYTPHQGVINISVYTDPDHYACIQIEDSGAGIDPENYDKVLKRFYRVHHHLEVGSGL**

5 A5 100.0% 99.8% **PIEMHSIEPTVHSIIFNLIDNAIKYTPHQGVINISVYTDPDHYACIQIEDSGAGIDPENYDKVLKRFYRVHHHLEVGSGL**

6 Aci00709 100.0% 99.8% **PIEMHSIEPTVHSIIFNLIDNAIKYTPHQGVINISVYTDPDHYACIQIEDSGAGIDPENYDKVLKRFYRVHHHLEVGSGL**

7 Aci00711 100.0% 99.8% **PIEMHSIEPTVHSIIFNLIDNAIKYTPHQGVINISVYTDPDHYACIQIEDSGAGIDPENYDKVLKRFYRVHHHLEVGSGL**

8 KAB15 100.0% 99.8% **PIEMHSIEPTVHSIIFNLIDNAIKYTPHQGVINISVYTDPDHYACIQIEDSGAGIDPENYDKVLKRFYRVHHHLEVGSGL**

9 PT022 100.0% 99.8% **PIEMHSIEPTVHSIIFNLIDNAIKYTPHQGVINISVYTDPDHYACIQIEDSGAGIDPENYDKVLKRFYRVHHHLEVGSGL**

10 PSU091 100.0% 99.8% **PIEMHSIEPTVHSIIFNLIDNAIKYTPHQGVINISVYTDPDHYACIQIEDSGAGIDPENYDKVLKRFYRVHHHLEVGSGL**

11 151 100.0% 99.5% **PIEMHSIEPTVHSIIFNLIDNAIKYTPHQGVINISVYTDPDHYACIQIEDSGAGIDPENYDKVLKRFYRVHHHLEVGSGL**

12 GML-KP48-AB-TR 100.0% 99.3% **PIEMHSIEPTVHSIIFNLIDNAIKYTPHQGVINISVYTDPDHYACIQIEDSGAGIDPENYDKVLKRFYRVHHHLEVGSGL**

13 198 100.0% 99.5% **PIEMHSIEPTVHSIIFNLIDNAIKYTPHQGVINISVYTDPDHYACIQIEDSGAGIDPENYDKVLKRFYRVHHHLEVGSGL**

14 4300STDY7045706 100.0% 99.8% **PIEMHSIEPTVHSIIFNLIDNAIKYTPHQGVINISVYTDPDHYACIQIEDSGAGIDPENYDKVLKRFYRVHHHLEVGSGL**

15 4300STDY7045763 100.0% 99.8% **PIEMHSIEPTVHSIIFNLIDNAIKYTPHQGVINISVYTDPDHYACIQIEDSGAGIDPENYDKVLKRFYRVHHHLEVGSGL**

16 4300STDY7045798 100.0% 99.8% **PIEMHSIEPTVHSIIFNLIDNAIKYTPHQGVINISVYTDPDHYACIQIEDSGAGIDPENYDKVLKRFYRVHHHLEVGSGL**

17 4300STDY7045799 100.0% 99.8% **PIEMHSIEPTVHSIIFNLIDNAIKYTPHQGVINISVYTDPDHYACIQIEDSGAGIDPENYDKVLKRFYRVHHHLEVGSGL**

18 4300STDY7045808 100.0% 99.8% **PIEMHSIEPTVHSIIFNLIDNAIKYTPHQGVINISVYTDPDHYACIQIEDSGAGIDPENYDKVLKRFYRVHHHLEVGSGL**

19 4300STDY7045806 100.0% 99.8% **PIEMHSIEPTVHSIIFNLIDNAIKYTPHQGVINISVYTDPDHYACIQIEDSGAGIDPENYDKVLKRFYRVHHHLEVGSGL**

20 4300STDY7045811 100.0% 99.8% **PIEMHSIEPTVHSIIFNLIDNAIKYTPHQGVINISVYTDPDHYACIQIEDSGAGIDPENYDKVLKRFYRVHHHLEVGSGL**

21 4300STDY7045829 100.0% 99.8% **PIEMHSIEPTVHSIIFNLIDNAIKYTPHQGVINISVYTDPDHYACIQIEDSGAGIDPENYDKVLKRFYRVHHHLEVGSGL**

22 4300STDY7045870 100.0% 99.8% **PIEMHSIEPTVHSIIFNLIDNAIKYTPHQGVINISVYTDPDHYACIQIEDSGAGIDPENYDKVLKRFYRVHHHLEVGSGL**

23 4300STDY7045866 100.0% 99.8% **PIEMHSIEPTVHSIIFNLIDNAIKYTPHQGVINISVYTDPDHYACIQIEDSGAGIDPENYDKVLKRFYRVHHHLEVGSGL**

cov pid **401**  **. . . . ]** **444**

1 ATCC19606 100.0% 100.0% **GLSIVDRATQRLGGTLTLDKSLELGGLSVLVKLPKVLHLNETRA**

2 M19 74.5% 99.7% **--------------------------------------------**

3 M19b 21.2% 93.6% **GLSIVDRATQRLGGTLTLDKSLELGGLSVLVKLPKVLHLHETRA**

4 M10 66.4% 99.7% **GLSIVDRATQRLGGTLTLDKSLELGGLSVLVKLPKVLHLHETRA**

5 A5 100.0% 99.8% **GLSIVDRATQRLGGTLTLDKSLELGGLSVLVKLPKVLHLHETRA**

6 Aci00709 100.0% 99.8% **GLSIVDRATQRLGGTLTLDKSLELGGLSVLVKLPKVLHLHETRA**

7 Aci00711 100.0% 99.8% **GLSIVDRATQRLGGTLTLDKSLELGGLSVLVKLPKVLHLHETRA**

8 KAB15 100.0% 99.8% **GLSIVDRATQRLGGTLTLDKSLELGGLSVLVKLPKVLHLHETRA**

9 PT022 100.0% 99.8% **GLSIVDRATQRLGGTLTLDKSLELGGLSVLVKLPKVLHLHETRA**

10 PSU091 100.0% 99.8% **GLSIVDRATQRLGGTLTLDKSLELGGLSVLVKLPKVLHLHETRA**

11 151 100.0% 99.5% **GLSIVDRATQRLGGTLTLDKSLELGGLSVLVKLPKVLHLHETRA**

12 GML-KP48-AB-TR 100.0% 99.3% **GLSIVDRETQRLGGTLTLDKSLELGGLSVLVKLPKVLHLHETRA**

13 198 100.0% 99.5% **GLSIVDRATQRLGGTLTLDKSLELGGLSVLVKLPKVLHLHETRA**

14 4300STDY7045706 100.0% 99.8% **GLSIVDRATQRLGGTLTLDKSLELGGLSVLVKLPKVLHLHETRA**

15 4300STDY7045763 100.0% 99.8% **GLSIVDRATQRLGGTLTLDKSLELGGLSVLVKLPKVLHLHETRA**

16 4300STDY7045798 100.0% 99.8% **GLSIVDRATQRLGGTLTLDKSLELGGLSVLVKLPKVLHLHETRA**

17 4300STDY7045799 100.0% 99.8% **GLSIVDRATQRLGGTLTLDKSLELGGLSVLVKLPKVLHLHETRA**

18 4300STDY7045808 100.0% 99.8% **GLSIVDRATQRLGGTLTLDKSLELGGLSVLVKLPKVLHLHETRA**

19 4300STDY7045806 100.0% 99.8% **GLSIVDRATQRLGGTLTLDKSLELGGLSVLVKLPKVLHLHETRA**

20 4300STDY7045811 100.0% 99.8% **GLSIVDRATQRLGGTLTLDKSLELGGLSVLVKLPKVLHLHETRA**

21 4300STDY7045829 100.0% 99.8% **GLSIVDRATQRLGGTLTLDKSLELGGLSVLVKLPKVLHLHETRA**

22 4300STDY7045870 100.0% 99.8% **GLSIVDRATQRLGGTLTLDKSLELGGLSVLVKLPKVLHLHETRA**

23 4300STDY7045866 100.0% 99.8% **GLSIVDRATQRLGGTLTLDKSLELGGLSVLVKLPKVLHLHETRA**

**Supplementary Figure 10: Multiple sequence alignment (MSA) of the predicted amino acid sequence of PmrB carried by ST164^Pas^ isolates and the phylogenetically related isolate M10 and close genomes retrieved from the BV-BRC database compared to the respective gene in *A. baumannii* ATCC19606.** MSA was created by the A multiple alignment viewer MView hosted by the EMBL-EBI; cov, coverage; pid, percent identity.

cov pid  **1** **[ . . . . : . . .** **80**

1 ATCC19606 100.0% 100.0% **MVKQRTLNRVVKASGIGLHSGQKVMINFIPHTVDGGIVFRRIDLDPPVDIPANALLIQEAFMCSNLVTGDIKVGTIEHVM**

2 M06 100.0% 99.7% **MVKQRTLNRVVKASGIGLHSGQKVMINFIPHTVDGGIVFRRIDLDPPVDIPANALLIQEAFMCSNLVTGDIKVGTIEHVM**

3 M09 40.0% 100.0% **MVKQRTLNRVVKASGIGLHSGQKVMINFIPHTVDGGIVFRRIDLDPPVDIPANALLIQEAFMCSNLVTGDIKVGTIEHVM**

4 M09b 59.7% 92.7% **--------------------------------------------------------------------------------**

5 M12 100.0% 99.7% **MVKQRTLNRVVKASGIGLHSGQKVMINFIPHTVDGGIVFRRIDLDPPVDIPANALLIQEAFMCSNLVTGDIKVGTIEHVM**

6 M15 100.0% 99.7% **MVKQRTLNRVVKASGIGLHSGQKVMINFIPHTVDGGIVFRRIDLDPPVDIPANALLIQEAFMCSNLVTGDIKVGTIEHVM**

7 MRSN7133 100.0% 99.7% **MVKQRTLNRVVKASGIGLHSGQKVMINFIPHTVDGGIVFRRIDLDPPVDIPANALLIQEAFMCSNLVTGDIKVGTIEHVM**

8 SRR3222490 100.0% 99.7% **MVKQRTLNRVVKASGIGLHSGQKVMINFIPHTVDGGIVFRRIDLDPPVDIPANALLIQEAFMCSNLVTGDIKVGTIEHVM**

9 MRSN7224 100.0% 99.7% **MVKQRTLNRVVKASGIGLHSGQKVMINFIPHTVDGGIVFRRIDLDPPVDIPANALLIQEAFMCSNLVTGDIKVGTIEHVM**

10 SRR3227013 100.0% 99.7% **MVKQRTLNRVVKASGIGLHSGQKVMINFIPHTVDGGIVFRRIDLDPPVDIPANALLIQEAFMCSNLVTGDIKVGTIEHVM**

11 SRR3228488 100.0% 99.7% **MVKQRTLNRVVKASGIGLHSGQKVMINFIPHTVDGGIVFRRIDLDPPVDIPANALLIQEAFMCSNLVTGDIKVGTIEHVM**

12 OIFC074 100.0% 99.7% **MVKQRTLNRVVKASGIGLHSGQKVMINFIPHTVDGGIVFRRIDLDPPVDIPANALLIQEAFMCSNLVTGDIKVGTIEHVM**

13 15A1042 100.0% 99.7% **MVKQRTLNRVVKASGIGLHSGQKVMINFIPHTVDGGIVFRRIDLDPPVDIPANALLIQEAFMCSNLVTGDIKVGTIEHVM**

14 SRR3228565 100.0% 99.7% **MVKQRTLNRVVKASGIGLHSGQKVMINFIPHTVDGGIVFRRIDLDPPVDIPANALLIQEAFMCSNLVTGDIKVGTIEHVM**

15 MRSN7202 100.0% 99.7% **MVKQRTLNRVVKASGIGLHSGQKVMINFIPHTVDGGIVFRRIDLDPPVDIPANALLIQEAFMCSNLVTGDIKVGTIEHVM**

16 A18 100.0% 99.7% **MVKQRTLNRVVKASGIGLHSGQKVMINFIPHTVDGGIVFRRIDLDPPVDIPANALLIQEAFMCSNLVTGDIKVGTIEHVM**

17 MRSN7130 100.0% 99.7% **MVKQRTLNRVVKASGIGLHSGQKVMINFIPHTVDGGIVFRRIDLDPPVDIPANALLIQEAFMCSNLVTGDIKVGTIEHVM**

cov pid  **81**  **. 1 . . . . : .** **160**

1 ATCC19606 100.0% 100.0% **SAIAGLGIDNLIVEVSASEVPIMDGSAGPFIYLLMQGGLREQDAPKKFIKILKPVEALIDDKKAIFSPHNGFQLNFTIDF**

2 M06 100.0% 99.7% **SAIAGLGIDNLIVEVSASEVPIMDGSAGPFIYLLMQGGLREQDAPKKFIKILKPVEALIDDKKAIFSPHNGFQLNFTIDF**

3 M09 40.0% 100.0% **SAIAGLGIDNLIVEVSASEVPIMDGSAGPFIYLLMQGGLR----------------------------------------**

4 M09b 59.7% 99.7% **---------------------------------------------------------------------------FTIDF**

5 M12 100.0% 99.7% **SAIAGLGIDNLIVEVSASEVPIMDGSAGPFIYLLMQGGLREQDAPKKFIKILKPVEALIDDKKAIFSPHNGFQLNFTIDF**

6 M15 100.0% 99.7% **SAIAGLGIDNLIVEVSASEVPIMDGSAGPFIYLLMQGGLREQDAPKKFIKILKPVEALIDDKKAIFSPHNGFQLNFTIDF**

7 MRSN7133 100.0% 99.7% **SAIAGLGIDNLIVEVSASEVPIMDGSAGPFIYLLMQGGLREQDAPKKFIKILKPVEALIDDKKAIFSPHNGFQLNFTIDF**

8 SRR3222490 100.0% 99.7% **SAIAGLGIDNLIVEVSASEVPIMDGSAGPFIYLLMQGGLREQDAPKKFIKILKPVEALIDDKKAIFSPHNGFQLNFTIDF**

9 MRSN7224 100.0% 99.7% **SAIAGLGIDNLIVEVSASEVPIMDGSAGPFIYLLMQGGLREQDAPKKFIKILKPVEALIDDKKAIFSPHNGFQLNFTIDF**

10 SRR3227013 100.0% 99.7% **SAIAGLGIDNLIVEVSASEVPIMDGSAGPFIYLLMQGGLREQDAPKKFIKILKPVEALIDDKKAIFSPHNGFQLNFTIDF**

11 SRR3228488 100.0% 99.7% **SAIAGLGIDNLIVEVSASEVPIMDGSAGPFIYLLMQGGLREQDAPKKFIKILKPVEALIDDKKAIFSPHNGFQLNFTIDF**

12 OIFC074 100.0% 99.7% **SAIAGLGIDNLIVEVSASEVPIMDGSAGPFIYLLMQGGLREQDAPKKFIKILKPVEALIDDKKAIFSPHNGFQLNFTIDF**

13 15A1042 100.0% 99.7% **SAIAGLGIDNLIVEVSASEVPIMDGSAGPFIYLLMQGGLREQDAPKKFIKILKPVEALIDDKKAIFSPHNGFQLNFTIDF**

14 SRR3228565 100.0% 99.7% **SAIAGLGIDNLIVEVSASEVPIMDGSAGPFIYLLMQGGLREQDAPKKFIKILKPVEALIDDKKAIFSPHNGFQLNFTIDF**

15 MRSN7202 100.0% 99.7% **SAIAGLGIDNLIVEVSASEVPIMDGSAGPFIYLLMQGGLREQDAPKKFIKILKPVEALIDDKKAIFSPHNGFQLNFTIDF**

16 A18 100.0% 99.7% **SAIAGLGIDNLIVEVSASEVPIMDGSAGPFIYLLMQGGLREQDAPKKFIKILKPVEALIDDKKAIFSPHNGFQLNFTIDF**

17 MRSN7130 100.0% 99.7% **SAIAGLGIDNLIVEVSASEVPIMDGSAGPFIYLLMQGGLREQDAPKKFIKILKPVEALIDDKKAIFSPHNGFQLNFTIDF**

cov pid **161**  **. . . 2 . . . .** **240**

1 ATCC19606 100.0% 100.0% **DHPAFAKEYQSATIDFSTETFVYEVSEARTFGFMKDLDYLKANNLALGASLDNAIGVDDTGVVNEEGLRFADEFVRHKIL**

2 M06 100.0% 99.7% **DHPAFAKEYQSATIDFSTETFVYEVSEARTFGFMKDLDYLKANNLALGASLDNAIGVDDTGVVNEEGLRFADEFVRHKIL**

3 M09 40.0% 100.0% **--------------------------------------------------------------------------------**

4 M09b 59.7% 92.7% **DHPAFAKEYQSATIDFSTETFVYEVSEARTFGFMKDLDYLKANNLALGASLDNAIGVDDTGVVNEEGLRFADEFVRHKIL**

5 M12 100.0% 99.7% **DHPAFAKEYQSATIDFSTETFVYEVSEARTFGFMKDLDYLKANNLALGASLDNAIGVDDTGVVNEEGLRFADEFVRHKIL**

6 M15 100.0% 99.7% **DHPAFAKEYQSATIDFSTETFVYEVSEARTFGFMKDLDYLKANNLALGASLDNAIGVDDTGVVNEEGLRFADEFVRHKIL**

7 MRSN7133 100.0% 99.7% **DHPAFAKEYQSATIDFSTETFVYEVSEARTFGFMKDLDYLKANNLALGASLDNAIGVDDTGVVNEEGLRFADEFVRHKIL**

8 SRR3222490 100.0% 99.7% **DHPAFAKEYQSATIDFSTETFVYEVSEARTFGFMKDLDYLKANNLALGASLDNAIGVDDTGVVNEEGLRFADEFVRHKIL**

9 MRSN7224 100.0% 99.7% **DHPAFAKEYQSATIDFSTETFVYEVSEARTFGFMKDLDYLKANNLALGASLDNAIGVDDTGVVNEEGLRFADEFVRHKIL**

10 SRR3227013 100.0% 99.7% **DHPAFAKEYQSATIDFSTETFVYEVSEARTFGFMKDLDYLKANNLALGASLDNAIGVDDTGVVNEEGLRFADEFVRHKIL**

11 SRR3228488 100.0% 99.7% **DHPAFAKEYQSATIDFSTETFVYEVSEARTFGFMKDLDYLKANNLALGASLDNAIGVDDTGVVNEEGLRFADEFVRHKIL**

12 OIFC074 100.0% 99.7% **DHPAFAKEYQSATIDFSTETFVYEVSEARTFGFMKDLDYLKANNLALGASLDNAIGVDDTGVVNEEGLRFADEFVRHKIL**

13 15A1042 100.0% 99.7% **DHPAFAKEYQSATIDFSTETFVYEVSEARTFGFMKDLDYLKANNLALGASLDNAIGVDDTGVVNEEGLRFADEFVRHKIL**

14 SRR3228565 100.0% 99.7% **DHPAFAKEYQSATIDFSTETFVYEVSEARTFGFMKDLDYLKANNLALGASLDNAIGVDDTGVVNEEGLRFADEFVRHKIL**

15 MRSN7202 100.0% 99.7% **DHPAFAKEYQSATIDFSTETFVYEVSEARTFGFMKDLDYLKANNLALGASLDNAIGVDDTGVVNEEGLRFADEFVRHKIL**

16 A18 100.0% 99.7% **DHPAFAKEYQSATIDFSTETFVYEVSEARTFGFMKDLDYLKANNLALGASLDNAIGVDDTGVVNEEGLRFADEFVRHKIL**

17 MRSN7130 100.0% 99.7% **DHPAFAKEYQSATIDFSTETFVYEVSEARTFGFMKDLDYLKANNLALGASLDNAIGVDDTGVVNEEGLRFADEFVRHKIL**

cov pid **241**  **: . . . . ]** **300**

1 ATCC19606 100.0% 100.0% **DAVGDLYLLGHQIIAKFDGYKSGHALNNQLLRNVQSDPSNYEIVTFNDEKDCPIPYVSVT**

2 M06 100.0% 99.7% **DAVGDLYLLGHQIIAKFDGYKSGHALNNQLLRNVQSDPSNYEIVTFDDEKDCPIPYVSVT**

3 M09 40.0% 100.0% **------------------------------------------------------------**

4 M09b 59.7% 92.7% **DAVGDLYLLGHQIIAKFDGYKSGHALNNQLLRNVQSDPSNYEIVTFDDEKDCPIPYVSVT**

5 M12 100.0% 99.7% **DAVGDLYLLGHQIIAKFDGYKSGHALNNQLLRNVQSDPSNYEIVTFDDEKDCPIPYVSVT**

6 M15 100.0% 99.7% **DAVGDLYLLGHQIIAKFDGYKSGHALNNQLLRNVQSDPSNYEIVTFDDEKDCPIPYVSVT**

7 MRSN7133 100.0% 99.7% **DAVGDLYLLGHQIIAKFDGYKSGHALNNQLLRNVQSDPSNYEIVTFDDEKDCPIPYVSVT**

8 SRR3222490 100.0% 99.7% **DAVGDLYLLGHQIIAKFDGYKSGHALNNQLLRNVQSDPSNYEIVTFDDEKDCPIPYVSVT**

9 MRSN7224 100.0% 99.7% **DAVGDLYLLGHQIIAKFDGYKSGHALNNQLLRNVQSDPSNYEIVTFDDEKDCPIPYVSVT**

10 SRR3227013 100.0% 99.7% **DAVGDLYLLGHQIIAKFDGYKSGHALNNQLLRNVQSDPSNYEIVTFDDEKDCPIPYVSVT**

11 SRR3228488 100.0% 99.7% **DAVGDLYLLGHQIIAKFDGYKSGHALNNQLLRNVQSDPSNYEIVTFDDEKDCPIPYVSVT**

12 OIFC074 100.0% 99.7% **DAVGDLYLLGHQIIAKFDGYKSGHALNNQLLRNVQSDPSNYEIVTFDDEKDCPIPYVSVT**

13 15A1042 100.0% 99.7% **DAVGDLYLLGHQIIAKFDGYKSGHALNNQLLRNVQSDPSNYEIVTFDDEKDCPIPYVSVT**

14 SRR3228565 100.0% 99.7% **DAVGDLYLLGHQIIAKFDGYKSGHALNNQLLRNVQSDPSNYEIVTFDDEKDCPIPYVSVT**

15 MRSN7202 100.0% 99.7% **DAVGDLYLLGHQIIAKFDGYKSGHALNNQLLRNVQSDPSNYEIVTFDDEKDCPIPYVSVT**

16 A18 100.0% 99.7% **DAVGDLYLLGHQIIAKFDGYKSGHALNNQLLRNVQSDPSNYEIVTFDDEKDCPIPYVSVT**

17 MRSN7130 100.0% 99.7% **DAVGDLYLLGHQIIAKFDGYKSGHALNNQLLRNVQSDPSNYEIVTFDDEKDCPIPYVSVT**

**Supplementary Figure 11: Multiple sequence alignment (MSA) of the predicted amino acid sequence of LpxC carried by ST19^Pas^ (GC1) and the phylogenetically related isolates M06 and M09 and close genomes retrieved from the BV-BRC database compared to the respective gene in *A. baumannii* ATCC 19606.** MSA was created by the A multiple alignment viewer MView hosted by the EMBL-EBI; cov, coverage; pid, percent identity.

cov pid  **1** **[ . . . . : . . .** **80**

1 ATCC19606 100.0% 100.0% **MVKQRTLNRVVKASGIGLHSGQKVMINFIPHTVDGGIVFRRIDLDPPVDIPANALLIQEAFMCSNLVTGDIKVGTIEHVM**

2 M01 100.0% 99.7% **MVKQRTLNRVVKASGIGLHSGQKVMINFIPHTVDGGIVFRRIDLDPPVDIPANALLIQEAFMCSNLVTGDIKVGTIEHVM**

3 M04 100.0% 99.7% **MVKQRTLNRVVKASGIGLHSGQKVMINFIPHTVDGGIVFRRIDLDPPVDIPANALLIQEAFMCSNLVTGDIKVGTIEHVM**

4 M05 100.0% 99.7% **MVKQRTLNRVVKASGIGLHSGQKVMINFIPHTVDGGIVFRRIDLDPPVDIPANALLIQEAFMCSNLVTGDIKVGTIEHVM**

5 M13 100.0% 99.7% **MVKQRTLNRVVKASGIGLHSGQKVMINFIPHTVDGGIVFRRIDLDPPVDIPANALLIQEAFMCSNLVTGDIKVGTIEHVM**

6 M16 100.0% 99.7% **MVKQRTLNRVVKASGIGLHSGQKVMINFIPHTVDGGIVFRRIDLDPPVDIPANALLIQEAFMCSNLVTGDIKVGTIEHVM**

7 M17 100.0% 99.7% **MVKQRTLNRVVKASGIGLHSGQKVMINFIPHTVDGGIVFRRIDLDPPVDIPANALLIQEAFMCSNLVTGDIKVGTIEHVM**

8 M20 100.0% 99.7% **MVKQRTLNRVVKASGIGLHSGQKVMINFIPHTVDGGIVFRRIDLDPPVDIPANALLIQEAFMCSNLVTGDIKVGTIEHVM**

9 MS14413 100.0% 99.7% **MVKQRTLNRVVKASGIGLHSGQKVMINFIPHTVDGGIVFRRIDLDPPVDIPANALLIQEAFMCSNLVTGDIKVGTIEHVM**

10 SQ093 100.0% 99.7% **MVKQRTLNRVVKASGIGLHSGQKVMINFIPHTVDGGIVFRRIDLDPPVDIPANALLIQEAFMCSNLVTGDIKVGTIEHVM**

11 KAB3 100.0% 99.7% **MVKQRTLNRVVKASGIGLHSGQKVMINFIPHTVDGGIVFRRIDLDPPVDIPANALLIQEAFMCSNLVTGDIKVGTIEHVM**

12 AC-40 100.0% 99.7% **MVKQRTLNRVVKASGIGLHSGQKVMINFIPHTVDGGIVFRRIDLDPPVDIPANALLIQEAFMCSNLVTGDIKVGTIEHVM**

13 AC-14 100.0% 99.7% **MVKQRTLNRVVKASGIGLHSGQKVMINFIPHTVDGGIVFRRIDLDPPVDIPANALLIQEAFMCSNLVTGDIKVGTIEHVM**

14 AC-45 100.0% 99.7% **MVKQRTLNRVVKASGIGLHSGQKVMINFIPHTVDGGIVFRRIDLDPPVDIPANALLIQEAFMCSNLVTGDIKVGTIEHVM**

15 AC-23 100.0% 99.7% **MVKQRTLNRVVKASGIGLHSGQKVMINFIPHTVDGGIVFRRIDLDPPVDIPANALLIQEAFMCSNLVTGDIKVGTIEHVM**

16 SUH-26-2 100.0% 99.7% **MVKQRTLNRVVKASGIGLHSGQKVMINFIPHTVDGGIVFRRIDLDPPVDIPANALLIQEAFMCSNLVTGDIKVGTIEHVM**

17 SUH-11-2 100.0% 99.7% **MVKQRTLNRVVKASGIGLHSGQKVMINFIPHTVDGGIVFRRIDLDPPVDIPANALLIQEAFMCSNLVTGDIKVGTIEHVM**

18 SUH-11-1 100.0% 99.7% **MVKQRTLNRVVKASGIGLHSGQKVMINFIPHTVDGGIVFRRIDLDPPVDIPANALLIQEAFMCSNLVTGDIKVGTIEHVM**

19 A21 100.0% 99.7% **MVKQRTLNRVVKASGIGLHSGQKVMINFIPHTVDGGIVFRRIDLDPPVDIPANALLIQEAFMCSNLVTGDIKVGTIEHVM**

20 SK044 100.0% 99.7% **MVKQRTLNRVVKASGIGLHSGQKVMINFIPHTVDGGIVFRRIDLDPPVDIPANALLIQEAFMCSNLVTGDIKVGTIEHVM**

21 SK011 100.0% 99.7% **MVKQRTLNRVVKASGIGLHSGQKVMINFIPHTVDGGIVFRRIDLDPPVDIPANALLIQEAFMCSNLVTGDIKVGTIEHVM**

22 SK002 100.0% 99.7% **MVKQRTLNRVVKASGIGLHSGQKVMINFIPHTVDGGIVFRRIDLDPPVDIPANALLIQEAFMCSNLVTGDIKVGTIEHVM**

23 PSU120 100.0% 99.7% **MVKQRTLNRVVKASGIGLHSGQKVMINFIPHTVDGGIVFRRIDLDPPVDIPANALLIQEAFMCSNLVTGDIKVGTIEHVM**

24 KUSSH35 100.0% 99.7% **MVKQRTLNRVVKASGIGLHSGQKVMINFIPHTVDGGIVFRRIDLDPPVDIPANALLIQEAFMCSNLVTGDIKVGTIEHVM**

25 KUFAR56 100.0% 99.7% **MVKQRTLNRVVKASGIGLHSGQKVMINFIPHTVDGGIVFRRIDLDPPVDIPANALLIQEAFMCSNLVTGDIKVGTIEHVM**

26 KUSSH15 100.0% 99.7% **MVKQRTLNRVVKASGIGLHSGQKVMINFIPHTVDGGIVFRRIDLDPPVDIPANALLIQEAFMCSNLVTGDIKVGTIEHVM**

27 KUSSH37 100.0% 99.7% **MVKQRTLNRVVKASGIGLHSGQKVMINFIPHTVDGGIVFRRIDLDPPVDIPANALLIQEAFMCSNLVTGDIKVGTIEHVM**

28 KUSSH36 100.0% 99.7% **MVKQRTLNRVVKASGIGLHSGQKVMINFIPHTVDGGIVFRRIDLDPPVDIPANALLIQEAFMCSNLVTGDIKVGTIEHVM**

29 130 100.0% 99.7% **MVKQRTLNRVVKASGIGLHSGQKVMINFIPHTVDGGIVFRRIDLDPPVDIPANALLIQEAFMCSNLVTGDIKVGTIEHVM**

30 Ab34 100.0% 99.7% **MVKQRTLNRVVKASGIGLHSGQKVMINFIPHTVDGGIVFRRIDLDPPVDIPANALLIQEAFMCSNLVTGDIKVGTIEHVM**

31 Ab35 100.0% 99.7% **MVKQRTLNRVVKASGIGLHSGQKVMINFIPHTVDGGIVFRRIDLDPPVDIPANALLIQEAFMCSNLVTGDIKVGTIEHVM**

32 Ab36 100.0% 99.7% **MVKQRTLNRVVKASGIGLHSGQKVMINFIPHTVDGGIVFRRIDLDPPVDIPANALLIQEAFMCSNLVTGDIKVGTIEHVM**

33 Ab38 100.0% 99.7% **MVKQRTLNRVVKASGIGLHSGQKVMINFIPHTVDGGIVFRRIDLDPPVDIPANALLIQEAFMCSNLVTGDIKVGTIEHVM**

34 Ab40 100.0% 99.7% **MVKQRTLNRVVKASGIGLHSGQKVMINFIPHTVDGGIVFRRIDLDPPVDIPANALLIQEAFMCSNLVTGDIKVGTIEHVM**

35 Ab41 100.0% 99.7% **MVKQRTLNRVVKASGIGLHSGQKVMINFIPHTVDGGIVFRRIDLDPPVDIPANALLIQEAFMCSNLVTGDIKVGTIEHVM**

36 Ab15 100.0% 99.7% **MVKQRTLNRVVKASGIGLHSGQKVMINFIPHTVDGGIVFRRIDLDPPVDIPANALLIQEAFMCSNLVTGDIKVGTIEHVM**

37 Ab65 100.0% 99.7% **MVKQRTLNRVVKASGIGLHSGQKVMINFIPHTVDGGIVFRRIDLDPPVDIPANALLIQEAFMCSNLVTGDIKVGTIEHVM**

38 Ab64 100.0% 99.7% **MVKQRTLNRVVKASGIGLHSGQKVMINFIPHTVDGGIVFRRIDLDPPVDIPANALLIQEAFMCSNLVTGDIKVGTIEHVM**

39 AB22 100.0% 99.7% **MVKQRTLNRVVKASGIGLHSGQKVMINFIPHTVDGGIVFRRIDLDPPVDIPANALLIQEAFMCSNLVTGDIKVGTIEHVM**

cov pid  **81**  **. 1 . . . . : .** **160**

1 ATCC19606 100.0% 100.0% **SAIAGLGIDNLIVEVSASEVPIMDGSAGPFIYLLMQGGLREQDAPKKFIKILKPVEALIDDKKAIFSPHNGFQLNFTIDF**

2 M01 100.0% 99.7% **SAIAGLGIDNLIVEVSASEVPIMDGSAGPFIYLLMQGGLREQDAPKKFIKILKPVEALIDDKKAIFSPHNGFQLNFTIDF**

3 M04 100.0% 99.7% **SAIAGLGIDNLIVEVSASEVPIMDGSAGPFIYLLMQGGLREQDAPKKFIKILKPVEALIDDKKAIFSPHNGFQLNFTIDF**

4 M05 100.0% 99.7% **SAIAGLGIDNLIVEVSASEVPIMDGSAGPFIYLLMQGGLREQDAPKKFIKILKPVEALIDDKKAIFSPHNGFQLNFTIDF**

5 M13 100.0% 99.7% **SAIAGLGIDNLIVEVSASEVPIMDGSAGPFIYLLMQGGLREQDAPKKFIKILKPVEALIDDKKAIFSPHNGFQLNFTIDF**

6 M16 100.0% 99.7% **SAIAGLGIDNLIVEVSASEVPIMDGSAGPFIYLLMQGGLREQDAPKKFIKILKPVEALIDDKKAIFSPHNGFQLNFTIDF**

7 M17 100.0% 99.7% **SAIAGLGIDNLIVEVSASEVPIMDGSAGPFIYLLMQGGLREQDAPKKFIKILKPVEALIDDKKAIFSPHNGFQLNFTIDF**

8 M20 100.0% 99.7% **SAIAGLGIDNLIVEVSASEVPIMDGSAGPFIYLLMQGGLREQDAPKKFIKILKPVEALIDDKKAIFSPHNGFQLNFTIDF**

9 MS14413 100.0% 99.7% **SAIAGLGIDNLIVEVSASEVPIMDGSAGPFIYLLMQGGLREQDAPKKFIKILKPVEALIDDKKAIFSPHNGFQLNFTIDF**

10 SQ093 100.0% 99.7% **SAIAGLGIDNLIVEVSASEVPIMDGSAGPFIYLLMQGGLREQDAPKKFIKILKPVEALIDDKKAIFSPHNGFQLNFTIDF**

11 KAB3 100.0% 99.7% **SAIAGLGIDNLIVEVSASEVPIMDGSAGPFIYLLMQGGLREQDAPKKFIKILKPVEALIDDKKAIFSPHNGFQLNFTIDF**

12 AC-40 100.0% 99.7% **SAIAGLGIDNLIVEVSASEVPIMDGSAGPFIYLLMQGGLREQDAPKKFIKILKPVEALIDDKKAIFSPHNGFQLNFTIDF**

13 AC-14 100.0% 99.7% **SAIAGLGIDNLIVEVSASEVPIMDGSAGPFIYLLMQGGLREQDAPKKFIKILKPVEALIDDKKAIFSPHNGFQLNFTIDF**

14 AC-45 100.0% 99.7% **SAIAGLGIDNLIVEVSASEVPIMDGSAGPFIYLLMQGGLREQDAPKKFIKILKPVEALIDDKKAIFSPHNGFQLNFTIDF**

15 AC-23 100.0% 99.7% **SAIAGLGIDNLIVEVSASEVPIMDGSAGPFIYLLMQGGLREQDAPKKFIKILKPVEALIDDKKAIFSPHNGFQLNFTIDF**

16 SUH-26-2 100.0% 99.7% **SAIAGLGIDNLIVEVSASEVPIMDGSAGPFIYLLMQGGLREQDAPKKFIKILKPVEALIDDKKAIFSPHNGFQLNFTIDF**

17 SUH-11-2 100.0% 99.7% **SAIAGLGIDNLIVEVSASEVPIMDGSAGPFIYLLMQGGLREQDAPKKFIKILKPVEALIDDKKAIFSPHNGFQLNFTIDF**

18 SUH-11-1 100.0% 99.7% **SAIAGLGIDNLIVEVSASEVPIMDGSAGPFIYLLMQGGLREQDAPKKFIKILKPVEALIDDKKAIFSPHNGFQLNFTIDF**

19 A21 100.0% 99.7% **SAIAGLGIDNLIVEVSASEVPIMDGSAGPFIYLLMQGGLREQDAPKKFIKILKPVEALIDDKKAIFSPHNGFQLNFTIDF**

20 SK044 100.0% 99.7% **SAIAGLGIDNLIVEVSASEVPIMDGSAGPFIYLLMQGGLREQDAPKKFIKILKPVEALIDDKKAIFSPHNGFQLNFTIDF**

21 SK011 100.0% 99.7% **SAIAGLGIDNLIVEVSASEVPIMDGSAGPFIYLLMQGGLREQDAPKKFIKILKPVEALIDDKKAIFSPHNGFQLNFTIDF**

22 SK002 100.0% 99.7% **SAIAGLGIDNLIVEVSASEVPIMDGSAGPFIYLLMQGGLREQDAPKKFIKILKPVEALIDDKKAIFSPHNGFQLNFTIDF**

23 PSU120 100.0% 99.7% **SAIAGLGIDNLIVEVSASEVPIMDGSAGPFIYLLMQGGLREQDAPKKFIKILKPVEALIDDKKAIFSPHNGFQLNFTIDF**

24 KUSSH35 100.0% 99.7% **SAIAGLGIDNLIVEVSASEVPIMDGSAGPFIYLLMQGGLREQDAPKKFIKILKPVEALIDDKKAIFSPHNGFQLNFTIDF**

25 KUFAR56 100.0% 99.7% **SAIAGLGIDNLIVEVSASEVPIMDGSAGPFIYLLMQGGLREQDAPKKFIKILKPVEALIDDKKAIFSPHNGFQLNFTIDF**

26 KUSSH15 100.0% 99.7% **SAIAGLGIDNLIVEVSASEVPIMDGSAGPFIYLLMQGGLREQDAPKKFIKILKPVEALIDDKKAIFSPHNGFQLNFTIDF**

27 KUSSH37 100.0% 99.7% **SAIAGLGIDNLIVEVSASEVPIMDGSAGPFIYLLMQGGLREQDAPKKFIKILKPVEALIDDKKAIFSPHNGFQLNFTIDF**

28 KUSSH36 100.0% 99.7% **SAIAGLGIDNLIVEVSASEVPIMDGSAGPFIYLLMQGGLREQDAPKKFIKILKPVEALIDDKKAIFSPHNGFQLNFTIDF**

29 130 100.0% 99.7% **SAIAGLGIDNLIVEVSASEVPIMDGSAGPFIYLLMQGGLREQDAPKKFIKILKPVEALIDDKKAIFSPHNGFQLNFTIDF**

30 Ab34 100.0% 99.7% **SAIAGLGIDNLIVEVSASEVPIMDGSAGPFIYLLMQGGLREQDAPKKFIKILKPVEALIDDKKAIFSPHNGFQLNFTIDF**

31 Ab35 100.0% 99.7% **SAIAGLGIDNLIVEVSASEVPIMDGSAGPFIYLLMQGGLREQDAPKKFIKILKPVEALIDDKKAIFSPHNGFQLNFTIDF**

32 Ab36 100.0% 99.7% **SAIAGLGIDNLIVEVSASEVPIMDGSAGPFIYLLMQGGLREQDAPKKFIKILKPVEALIDDKKAIFSPHNGFQLNFTIDF**

33 Ab38 100.0% 99.7% **SAIAGLGIDNLIVEVSASEVPIMDGSAGPFIYLLMQGGLREQDAPKKFIKILKPVEALIDDKKAIFSPHNGFQLNFTIDF**

34 Ab40 100.0% 99.7% **SAIAGLGIDNLIVEVSASEVPIMDGSAGPFIYLLMQGGLREQDAPKKFIKILKPVEALIDDKKAIFSPHNGFQLNFTIDF**

35 Ab41 100.0% 99.7% **SAIAGLGIDNLIVEVSASEVPIMDGSAGPFIYLLMQGGLREQDAPKKFIKILKPVEALIDDKKAIFSPHNGFQLNFTIDF**

36 Ab15 100.0% 99.7% **SAIAGLGIDNLIVEVSASEVPIMDGSAGPFIYLLMQGGLREQDAPKKFIKILKPVEALIDDKKAIFSPHNGFQLNFTIDF**

37 Ab65 100.0% 99.7% **SAIAGLGIDNLIVEVSASEVPIMDGSAGPFIYLLMQGGLREQDAPKKFIKILKPVEALIDDKKAIFSPHNGFQLNFTIDF**

38 Ab64 100.0% 99.7% **SAIAGLGIDNLIVEVSASEVPIMDGSAGPFIYLLMQGGLREQDAPKKFIKILKPVEALIDDKKAIFSPHNGFQLNFTIDF**

39 AB22 100.0% 99.7% **SAIAGLGIDNLIVEVSASEVPIMDGSAGPFIYLLMQGGLREQDAPKKFIKILKPVEALIDDKKAIFSPHNGFQLNFTIDF**

cov pid **161**  **. . . 2 . . . .** **240**

1 ATCC19606 100.0% 100.0% **DHPAFAKEYQSATIDFSTETFVYEVSEARTFGFMKDLDYLKANNLALGASLDNAIGVDDTGVVNEEGLRFADEFVRHKIL**

2 M01 100.0% 99.7% **DHPAFAKEYQSATIDFSTETFVYEVSEARTFGFMKDLDYLKANNLALGASLDNAIGVDDTGVVNEEGLRFADEFVRHKIL**

3 M04 100.0% 99.7% **DHPAFAKEYQSATIDFSTETFVYEVSEARTFGFMKDLDYLKANNLALGASLDNAIGVDDTGVVNEEGLRFADEFVRHKIL**

4 M05 100.0% 99.7% **DHPAFAKEYQSATIDFSTETFVYEVSEARTFGFMKDLDYLKANNLALGASLDNAIGVDDTGVVNEEGLRFADEFVRHKIL**

5 M13 100.0% 99.7% **DHPAFAKEYQSATIDFSTETFVYEVSEARTFGFMKDLDYLKANNLALGASLDNAIGVDDTGVVNEEGLRFADEFVRHKIL**

6 M16 100.0% 99.7% **DHPAFAKEYQSATIDFSTETFVYEVSEARTFGFMKDLDYLKANNLALGASLDNAIGVDDTGVVNEEGLRFADEFVRHKIL**

7 M17 100.0% 99.7% **DHPAFAKEYQSATIDFSTETFVYEVSEARTFGFMKDLDYLKANNLALGASLDNAIGVDDTGVVNEEGLRFADEFVRHKIL**

8 M20 100.0% 99.7% **DHPAFAKEYQSATIDFSTETFVYEVSEARTFGFMKDLDYLKANNLALGASLDNAIGVDDTGVVNEEGLRFADEFVRHKIL**

9 MS14413 100.0% 99.7% **DHPAFAKEYQSATIDFSTETFVYEVSEARTFGFMKDLDYLKANNLALGASLDNAIGVDDTGVVNEEGLRFADEFVRHKIL**

10 SQ093 100.0% 99.7% **DHPAFAKEYQSATIDFSTETFVYEVSEARTFGFMKDLDYLKANNLALGASLDNAIGVDDTGVVNEEGLRFADEFVRHKIL**

11 KAB3 100.0% 99.7% **DHPAFAKEYQSATIDFSTETFVYEVSEARTFGFMKDLDYLKANNLALGASLDNAIGVDDTGVVNEEGLRFADEFVRHKIL**

12 AC-40 100.0% 99.7% **DHPAFAKEYQSATIDFSTETFVYEVSEARTFGFMKDLDYLKANNLALGASLDNAIGVDDTGVVNEEGLRFADEFVRHKIL**

13 AC-14 100.0% 99.7% **DHPAFAKEYQSATIDFSTETFVYEVSEARTFGFMKDLDYLKANNLALGASLDNAIGVDDTGVVNEEGLRFADEFVRHKIL**

14 AC-45 100.0% 99.7% **DHPAFAKEYQSATIDFSTETFVYEVSEARTFGFMKDLDYLKANNLALGASLDNAIGVDDTGVVNEEGLRFADEFVRHKIL**

15 AC-23 100.0% 99.7% **DHPAFAKEYQSATIDFSTETFVYEVSEARTFGFMKDLDYLKANNLALGASLDNAIGVDDTGVVNEEGLRFADEFVRHKIL**

16 SUH-26-2 100.0% 99.7% **DHPAFAKEYQSATIDFSTETFVYEVSEARTFGFMKDLDYLKANNLALGASLDNAIGVDDTGVVNEEGLRFADEFVRHKIL**

17 SUH-11-2 100.0% 99.7% **DHPAFAKEYQSATIDFSTETFVYEVSEARTFGFMKDLDYLKANNLALGASLDNAIGVDDTGVVNEEGLRFADEFVRHKIL**

18 SUH-11-1 100.0% 99.7% **DHPAFAKEYQSATIDFSTETFVYEVSEARTFGFMKDLDYLKANNLALGASLDNAIGVDDTGVVNEEGLRFADEFVRHKIL**

19 A21 100.0% 99.7% **DHPAFAKEYQSATIDFSTETFVYEVSEARTFGFMKDLDYLKANNLALGASLDNAIGVDDTGVVNEEGLRFADEFVRHKIL**

20 SK044 100.0% 99.7% **DHPAFAKEYQSATIDFSTETFVYEVSEARTFGFMKDLDYLKANNLALGASLDNAIGVDDTGVVNEEGLRFADEFVRHKIL**

21 SK011 100.0% 99.7% **DHPAFAKEYQSATIDFSTETFVYEVSEARTFGFMKDLDYLKANNLALGASLDNAIGVDDTGVVNEEGLRFADEFVRHKIL**

22 SK002 100.0% 99.7% **DHPAFAKEYQSATIDFSTETFVYEVSEARTFGFMKDLDYLKANNLALGASLDNAIGVDDTGVVNEEGLRFADEFVRHKIL**

23 PSU120 100.0% 99.7% **DHPAFAKEYQSATIDFSTETFVYEVSEARTFGFMKDLDYLKANNLALGASLDNAIGVDDTGVVNEEGLRFADEFVRHKIL**

24 KUSSH35 100.0% 99.7% **DHPAFAKEYQSATIDFSTETFVYEVSEARTFGFMKDLDYLKANNLALGASLDNAIGVDDTGVVNEEGLRFADEFVRHKIL**

25 KUFAR56 100.0% 99.7% **DHPAFAKEYQSATIDFSTETFVYEVSEARTFGFMKDLDYLKANNLALGASLDNAIGVDDTGVVNEEGLRFADEFVRHKIL**

26 KUSSH15 100.0% 99.7% **DHPAFAKEYQSATIDFSTETFVYEVSEARTFGFMKDLDYLKANNLALGASLDNAIGVDDTGVVNEEGLRFADEFVRHKIL**

27 KUSSH37 100.0% 99.7% **DHPAFAKEYQSATIDFSTETFVYEVSEARTFGFMKDLDYLKANNLALGASLDNAIGVDDTGVVNEEGLRFADEFVRHKIL**

28 KUSSH36 100.0% 99.7% **DHPAFAKEYQSATIDFSTETFVYEVSEARTFGFMKDLDYLKANNLALGASLDNAIGVDDTGVVNEEGLRFADEFVRHKIL**

29 130 100.0% 99.7% **DHPAFAKEYQSATIDFSTETFVYEVSEARTFGFMKDLDYLKANNLALGASLDNAIGVDDTGVVNEEGLRFADEFVRHKIL**

30 Ab34 100.0% 99.7% **DHPAFAKEYQSATIDFSTETFVYEVSEARTFGFMKDLDYLKANNLALGASLDNAIGVDDTGVVNEEGLRFADEFVRHKIL**

31 Ab35 100.0% 99.7% **DHPAFAKEYQSATIDFSTETFVYEVSEARTFGFMKDLDYLKANNLALGASLDNAIGVDDTGVVNEEGLRFADEFVRHKIL**

32 Ab36 100.0% 99.7% **DHPAFAKEYQSATIDFSTETFVYEVSEARTFGFMKDLDYLKANNLALGASLDNAIGVDDTGVVNEEGLRFADEFVRHKIL**

33 Ab38 100.0% 99.7% **DHPAFAKEYQSATIDFSTETFVYEVSEARTFGFMKDLDYLKANNLALGASLDNAIGVDDTGVVNEEGLRFADEFVRHKIL**

34 Ab40 100.0% 99.7% **DHPAFAKEYQSATIDFSTETFVYEVSEARTFGFMKDLDYLKANNLALGASLDNAIGVDDTGVVNEEGLRFADEFVRHKIL**

35 Ab41 100.0% 99.7% **DHPAFAKEYQSATIDFSTETFVYEVSEARTFGFMKDLDYLKANNLALGASLDNAIGVDDTGVVNEEGLRFADEFVRHKIL**

36 Ab15 100.0% 99.7% **DHPAFAKEYQSATIDFSTETFVYEVSEARTFGFMKDLDYLKANNLALGASLDNAIGVDDTGVVNEEGLRFADEFVRHKIL**

37 Ab65 100.0% 99.7% **DHPAFAKEYQSATIDFSTETFVYEVSEARTFGFMKDLDYLKANNLALGASLDNAIGVDDTGVVNEEGLRFADEFVRHKIL**

38 Ab64 100.0% 99.7% **DHPAFAKEYQSATIDFSTETFVYEVSEARTFGFMKDLDYLKANNLALGASLDNAIGVDDTGVVNEEGLRFADEFVRHKIL**

39 AB22 100.0% 99.7% **DHPAFAKEYQSATIDFSTETFVYEVSEARTFGFMKDLDYLKANNLALGASLDNAIGVDDTGVVNEEGLRFADEFVRHKIL**

cov pid **241**  **: . . . . ]** **300**

1 ATCC19606 100.0% 100.0% **DAVGDLYLLGHQIIAKFDGYKSGHALNNQLLRNVQSDPSNYEIVTFNDEKDCPIPYVSVT**

2 M01 100.0% 99.7% **DAVGDLYLLGHQIIAKFDGYKSGHALNNQLLRNVQSDPSNYEIVTFDDEKDCPIPYVSVT**

3 M04 100.0% 99.7% **DAVGDLYLLGHQIIAKFDGYKSGHALNNQLLRNVQSDPSNYEIVTFDDEKDCPIPYVSVT**

4 M05 100.0% 99.7% **DAVGDLYLLGHQIIAKFDGYKSGHALNNQLLRNVQSDPSNYEIVTFDDEKDCPIPYVSVT**

5 M13 100.0% 99.7% **DAVGDLYLLGHQIIAKFDGYKSGHALNNQLLRNVQSDPSNYEIVTFDDEKDCPIPYVSVT**

6 M16 100.0% 99.7% **DAVGDLYLLGHQIIAKFDGYKSGHALNNQLLRNVQSDPSNYEIVTFDDEKDCPIPYVSVT**

7 M17 100.0% 99.7% **DAVGDLYLLGHQIIAKFDGYKSGHALNNQLLRNVQSDPSNYEIVTFDDEKDCPIPYVSVT**

8 M20 100.0% 99.7% **DAVGDLYLLGHQIIAKFDGYKSGHALNNQLLRNVQSDPSNYEIVTFDDEKDCPIPYVSVT**

9 MS14413 100.0% 99.7% **DAVGDLYLLGHQIIAKFDGYKSGHALNNQLLRNVQSDPSNYEIVTFDDEKDCPIPYVSVT**

10 SQ093 100.0% 99.7% **DAVGDLYLLGHQIIAKFDGYKSGHALNNQLLRNVQSDPSNYEIVTFDDEKDCPIPYVSVT**

11 KAB3 100.0% 99.7% **DAVGDLYLLGHQIIAKFDGYKSGHALNNQLLRNVQSDPSNYEIVTFDDEKDCPIPYVSVT**

12 AC-40 100.0% 99.7% **DAVGDLYLLGHQIIAKFDGYKSGHALNNQLLRNVQSDPSNYEIVTFDDEKDCPIPYVSVT**

13 AC-14 100.0% 99.7% **DAVGDLYLLGHQIIAKFDGYKSGHALNNQLLRNVQSDPSNYEIVTFDDEKDCPIPYVSVT**

14 AC-45 100.0% 99.7% **DAVGDLYLLGHQIIAKFDGYKSGHALNNQLLRNVQSDPSNYEIVTFDDEKDCPIPYVSVT**

15 AC-23 100.0% 99.7% **DAVGDLYLLGHQIIAKFDGYKSGHALNNQLLRNVQSDPSNYEIVTFDDEKDCPIPYVSVT**

16 SUH-26-2 100.0% 99.7% **DAVGDLYLLGHQIIAKFDGYKSGHALNNQLLRNVQSDPSNYEIVTFDDEKDCPIPYVSVT**

17 SUH-11-2 100.0% 99.7% **DAVGDLYLLGHQIIAKFDGYKSGHALNNQLLRNVQSDPSNYEIVTFDDEKDCPIPYVSVT**

18 SUH-11-1 100.0% 99.7% **DAVGDLYLLGHQIIAKFDGYKSGHALNNQLLRNVQSDPSNYEIVTFDDEKDCPIPYVSVT**

19 A21 100.0% 99.7% **DAVGDLYLLGHQIIAKFDGYKSGHALNNQLLRNVQSDPSNYEIVTFDDEKDCPIPYVSVT**

20 SK044 100.0% 99.7% **DAVGDLYLLGHQIIAKFDGYKSGHALNNQLLRNVQSDPSNYEIVTFDDEKDCPIPYVSVT**

21 SK011 100.0% 99.7% **DAVGDLYLLGHQIIAKFDGYKSGHALNNQLLRNVQSDPSNYEIVTFDDEKDCPIPYVSVT**

22 SK002 100.0% 99.7% **DAVGDLYLLGHQIIAKFDGYKSGHALNNQLLRNVQSDPSNYEIVTFDDEKDCPIPYVSVT**

23 PSU120 100.0% 99.7% **DAVGDLYLLGHQIIAKFDGYKSGHALNNQLLRNVQSDPSNYEIVTFDDEKDCPIPYVSVT**

24 KUSSH35 100.0% 99.7% **DAVGDLYLLGHQIIAKFDGYKSGHALNNQLLRNVQSDPSNYEIVTFDDEKDCPIPYVSVT**

25 KUFAR56 100.0% 99.7% **DAVGDLYLLGHQIIAKFDGYKSGHALNNQLLRNVQSDPSNYEIVTFDDEKDCPIPYVSVT**

26 KUSSH15 100.0% 99.7% **DAVGDLYLLGHQIIAKFDGYKSGHALNNQLLRNVQSDPSNYEIVTFDDEKDCPIPYVSVT**

27 KUSSH37 100.0% 99.7% **DAVGDLYLLGHQIIAKFDGYKSGHALNNQLLRNVQSDPSNYEIVTFDDEKDCPIPYVSVT**

28 KUSSH36 100.0% 99.7% **DAVGDLYLLGHQIIAKFDGYKSGHALNNQLLRNVQSDPSNYEIVTFDDEKDCPIPYVSVT**

29 130 100.0% 99.7% **DAVGDLYLLGHQIIAKFDGYKSGHALNNQLLRNVQSDPSNYEIVTFDDEKDCPIPYVSVT**

30 Ab34 100.0% 99.7% **DAVGDLYLLGHQIIAKFDGYKSGHALNNQLLRNVQSDPSNYEIVTFDDEKDCPIPYVSVT**

31 Ab35 100.0% 99.7% **DAVGDLYLLGHQIIAKFDGYKSGHALNNQLLRNVQSDPSNYEIVTFDDEKDCPIPYVSVT**

32 Ab36 100.0% 99.7% **DAVGDLYLLGHQIIAKFDGYKSGHALNNQLLRNVQSDPSNYEIVTFDDEKDCPIPYVSVT**

33 Ab38 100.0% 99.7% **DAVGDLYLLGHQIIAKFDGYKSGHALNNQLLRNVQSDPSNYEIVTFDDEKDCPIPYVSVT**

34 Ab40 100.0% 99.7% **DAVGDLYLLGHQIIAKFDGYKSGHALNNQLLRNVQSDPSNYEIVTFDDEKDCPIPYVSVT**

35 Ab41 100.0% 99.7% **DAVGDLYLLGHQIIAKFDGYKSGHALNNQLLRNVQSDPSNYEIVTFDDEKDCPIPYVSVT**

36 Ab15 100.0% 99.7% **DAVGDLYLLGHQIIAKFDGYKSGHALNNQLLRNVQSDPSNYEIVTFDDEKDCPIPYVSVT**

37 Ab65 100.0% 99.7% **DAVGDLYLLGHQIIAKFDGYKSGHALNNQLLRNVQSDPSNYEIVTFDDEKDCPIPYVSVT**

38 Ab64 100.0% 99.7% **DAVGDLYLLGHQIIAKFDGYKSGHALNNQLLRNVQSDPSNYEIVTFDDEKDCPIPYVSVT**

39 AB22 100.0% 99.7% **DAVGDLYLLGHQIIAKFDGYKSGHALNNQLLRNVQSDPSNYEIVTFDDEKDCPIPYVSVT**

**Supplementary Figure 12: Multiple sequence alignment (MSA) of the predicted amino acid sequence of LpxC carried by ST2^Pas^ and ST570^Pas^ (GC2) and close genomes retrieved from the BV-BRC database compared to the respective gene in *A. baumannii* ATCC 19606.** MSA was created by the A multiple alignment viewer MView hosted by the EMBL-EBI; cov, coverage; pid, percent identity.

cov pid  **1** **[ . . . . : . . .** **80**

1 ATCC19606 100.0% 100.0% **MVKQRTLNRVVKASGIGLHSGQKVMINFIPHTVDGGIVFRRIDLDPPVDIPANALLIQEAFMCSNLVTGDIKVGTIEHVM**

2 AB_1649-8 100.0% 99.7% **MVKQRTLNRVVKASGIGLHSGQKVMINFIPHTVDGGIVFRRIDLDPPVDIPANALLIQEAFMCSNLVTGDIKVGTIEHVM**

3 AB_1650-8 100.0% 99.7% **MVKQRTLNRVVKASGIGLHSGQKVMINFIPHTVDGGIVFRRIDLDPPVDIPANALLIQEAFMCSNLVTGDIKVGTIEHVM**

4 M14 100.0% 99.7% **MVKQRTLNRVVKASGIGLHSGQKVMINFIPHTVDGGIVFRRIDLDPPVDIPANALLIQEAFMCSNLVTGDIKVGTIEHVM**

5 M03 100.0% 99.7% **MVKQRTLNRVVKASGIGLHSGQKVMINFIPHTVDGGIVFRRIDLDPPVDIPANALLIQEAFMCSNLVTGDIKVGTIEHVM**

6 UV_1036 100.0% 99.7% **MVKQRTLNRVVKASGIGLHSGQKVMINFIPHTVDGGIVFRRIDLDPPVDIPANALLIQEAFMCSNLVTGDIKVGTIEHVM**

7 259_an 100.0% 99.7% **MVKQRTLNRVVKASGIGLHSGQKVMINFIPHTVDGGIVFRRIDLDPPVDIPANALLIQEAFMCSNLVTGDIKVGTIEHVM**

8 276_ax 100.0% 99.7% **MVKQRTLNRVVKASGIGLHSGQKVMINFIPHTVDGGIVFRRIDLDPPVDIPANALLIQEAFMCSNLVTGDIKVGTIEHVM**

9 PT061 100.0% 99.7% **MVKQRTLNRVVKASGIGLHSGQKVMINFIPHTVDGGIVFRRIDLDPPVDIPANALLIQEAFMCSNLVTGDIKVGTIEHVM**

10 PT003 100.0% 99.7% **MVKQRTLNRVVKASGIGLHSGQKVMINFIPHTVDGGIVFRRIDLDPPVDIPANALLIQEAFMCSNLVTGDIKVGTIEHVM**

11 PSU068 100.0% 99.7% **MVKQRTLNRVVKASGIGLHSGQKVMINFIPHTVDGGIVFRRIDLDPPVDIPANALLIQEAFMCSNLVTGDIKVGTIEHVM**

12 PSU073 100.0% 99.7% **MVKQRTLNRVVKASGIGLHSGQKVMINFIPHTVDGGIVFRRIDLDPPVDIPANALLIQEAFMCSNLVTGDIKVGTIEHVM**

13 CCBH26501 100.0% 99.7% **MVKQRTLNRVVKASGIGLHSGQKVMINFIPHTVDGGIVFRRIDLDPPVDIPANALLIQEAFMCSNLVTGDIKVGTIEHVM**

14 AB363 100.0% 99.7% **MVKQRTLNRVVKASGIGLHSGQKVMINFIPHTVDGGIVFRRIDLDPPVDIPANALLIQEAFMCSNLVTGDIKVGTIEHVM**

15 KUSSH08 100.0% 99.7% **MVKQRTLNRVVKASGIGLHSGQKVMINFIPHTVDGGIVFRRIDLDPPVDIPANALLIQEAFMCSNLVTGDIKVGTIEHVM**

16 KUSSH14 100.0% 99.7% **MVKQRTLNRVVKASGIGLHSGQKVMINFIPHTVDGGIVFRRIDLDPPVDIPANALLIQEAFMCSNLVTGDIKVGTIEHVM**

17 TUMA 100.0% 99.7% **MVKQRTLNRVVKASGIGLHSGQKVMINFIPHTVDGGIVFRRIDLDPPVDIPANALLIQEAFMCSNLVTGDIKVGTIEHVM**

18 4300STDY7045886 100.0% 99.7% **MVKQRTLNRVVKASGIGLHSGQKVMINFIPHTVDGGIVFRRIDLDPPVDIPANALLIQEAFMCSNLVTGDIKVGTIEHVM**

19 Aci00866 100.0% 99.7% **MVKQRTLNRVVKASGIGLHSGQKVMINFIPHTVDGGIVFRRIDLDPPVDIPANALLIQEAFMCSNLVTGDIKVGTIEHVM**

20 Aci00860 100.0% 99.7% **MVKQRTLNRVVKASGIGLHSGQKVMINFIPHTVDGGIVFRRIDLDPPVDIPANALLIQEAFMCSNLVTGDIKVGTIEHVM**

21 Aci00848 100.0% 99.7% **MVKQRTLNRVVKASGIGLHSGQKVMINFIPHTVDGGIVFRRIDLDPPVDIPANALLIQEAFMCSNLVTGDIKVGTIEHVM**

22 MRSN351524 100.0% 99.7% **MVKQRTLNRVVKASGIGLHSGQKVMINFIPHTVDGGIVFRRIDLDPPVDIPANALLIQEAFMCSNLVTGDIKVGTIEHVM**

cov pid  **81**  **. 1 . . . . : .** **160**

1 ATCC19606 100.0% 100.0% **SAIAGLGIDNLIVEVSASEVPIMDGSAGPFIYLLMQGGLREQDAPKKFIKILKPVEALIDDKKAIFSPHNGFQLNFTIDF**

2 AB_1649-8 100.0% 99.7% **SAIAGLGIDNLIVEVSASEVPIMDGSAGPFIYLLMQGGLREQDAPKKFIKILKPVEALIDDKKAIFSPHNGFQLNFTIDF**

3 AB_1650-8 100.0% 99.7% **SAIAGLGIDNLIVEVSASEVPIMDGSAGPFIYLLMQGGLREQDAPKKFIKILKPVEALIDDKKAIFSPHNGFQLNFTIDF**

4 M14 100.0% 99.7% **SAIAGLGIDNLIVEVSASEVPIMDGSAGPFIYLLMQGGLREQDAPKKFIKILKPVEALIDDKKAIFSPHNGFQLNFTIDF**

5 M03 100.0% 99.7% **SAIAGLGIDNLIVEVSASEVPIMDGSAGPFIYLLMQGGLREQDAPKKFIKILKPVEALIDDKKAIFSPHNGFQLNFTIDF**

6 UV_1036 100.0% 99.7% **SAIAGLGIDNLIVEVSASEVPIMDGSAGPFIYLLMQGGLREQDAPKKFIKILKPVEALIDDKKAIFSPHNGFQLNFTIDF**

7 259_an 100.0% 99.7% **SAIAGLGIDNLIVEVSASEVPIMDGSAGPFIYLLMQGGLREQDAPKKFIKILKPVEALIDDKKAIFSPHNGFQLNFTIDF**

8 276_ax 100.0% 99.7% **SAIAGLGIDNLIVEVSASEVPIMDGSAGPFIYLLMQGGLREQDAPKKFIKILKPVEALIDDKKAIFSPHNGFQLNFTIDF**

9 PT061 100.0% 99.7% **SAIAGLGIDNLIVEVSASEVPIMDGSAGPFIYLLMQGGLREQDAPKKFIKILKPVEALIDDKKAIFSPHNGFQLNFTIDF**

10 PT003 100.0% 99.7% **SAIAGLGIDNLIVEVSASEVPIMDGSAGPFIYLLMQGGLREQDAPKKFIKILKPVEALIDDKKAIFSPHNGFQLNFTIDF**

11 PSU068 100.0% 99.7% **SAIAGLGIDNLIVEVSASEVPIMDGSAGPFIYLLMQGGLREQDAPKKFIKILKPVEALIDDKKAIFSPHNGFQLNFTIDF**

12 PSU073 100.0% 99.7% **SAIAGLGIDNLIVEVSASEVPIMDGSAGPFIYLLMQGGLREQDAPKKFIKILKPVEALIDDKKAIFSPHNGFQLNFTIDF**

13 CCBH26501 100.0% 99.7% **SAIAGLGIDNLIVEVSASEVPIMDGSAGPFIYLLMQGGLREQDAPKKFIKILKPVEALIDDKKAIFSPHNGFQLNFTIDF**

14 AB363 100.0% 99.7% **SAIAGLGIDNLIVEVSASEVPIMDGSAGPFIYLLMQGGLREQDAPKKFIKILKPVEALIDDKKAIFSPHNGFQLNFTIDF**

15 KUSSH08 100.0% 99.7% **SAIAGLGIDNLIVEVSASEVPIMDGSAGPFIYLLMQGGLREQDAPKKFIKILKPVEALIDDKKAIFSPHNGFQLNFTIDF**

16 KUSSH14 100.0% 99.7% **SAIAGLGIDNLIVEVSASEVPIMDGSAGPFIYLLMQGGLREQDAPKKFIKILKPVEALIDDKKAIFSPHNGFQLNFTIDF**

17 TUMA 100.0% 99.7% **SAIAGLGIDNLIVEVSASEVPIMDGSAGPFIYLLMQGGLREQDAPKKFIKILKPVEALIDDKKAIFSPHNGFQLNFTIDF**

18 4300STDY7045886 100.0% 99.7% **SAIAGLGIDNLIVEVSASEVPIMDGSAGPFIYLLMQGGLREQDAPKKFIKILKPVEALIDDKKAIFSPHNGFQLNFTIDF**

19 Aci00866 100.0% 99.7% **SAIAGLGIDNLIVEVSASEVPIMDGSAGPFIYLLMQGGLREQDAPKKFIKILKPVEALIDDKKAIFSPHNGFQLNFTIDF**

20 Aci00860 100.0% 99.7% **SAIAGLGIDNLIVEVSASEVPIMDGSAGPFIYLLMQGGLREQDAPKKFIKILKPVEALIDDKKAIFSPHNGFQLNFTIDF**

21 Aci00848 100.0% 99.7% **SAIAGLGIDNLIVEVSASEVPIMDGSAGPFIYLLMQGGLREQDAPKKFIKILKPVEALIDDKKAIFSPHNGFQLNFTIDF**

22 MRSN351524 100.0% 99.7% **SAIAGLGIDNLIVEVSASEVPIMDGSAGPFIYLLMQGGLREQDAPKKFIKILKPVEALIDDKKAIFSPHNGFQLNFTIDF**

cov pid **161**  **. . . 2 . . . .** **240**

1 ATCC19606 100.0% 100.0% **DHPAFAKEYQSATIDFSTETFVYEVSEARTFGFMKDLDYLKANNLALGASLDNAIGVDDTGVVNEEGLRFADEFVRHKIL**

2 AB_1649-8 100.0% 99.7% **DHPAFAKEYQSATIDFSTETFVYEVSEARTFGFMKDLDYLKANNLALGASLDNAIGVDDTGVVNEEGLRFADEFVRHKIL**

3 AB_1650-8 100.0% 99.7% **DHPAFAKEYQSATIDFSTETFVYEVSEARTFGFMKDLDYLKANNLALGASLDNAIGVDDTGVVNEEGLRFADEFVRHKIL**

4 M14 100.0% 99.7% **DHPAFAKEYQSATIDFSTETFVYEVSEARTFGFMKDLDYLKANNLALGASLDNAIGVDDTGVVNEEGLRFADEFVRHKIL**

5 M03 100.0% 99.7% **DHPAFAKEYQSATIDFSTETFVYEVSEARTFGFMKDLDYLKANNLALGASLDNAIGVDDTGVVNEEGLRFADEFVRHKIL**

6 UV_1036 100.0% 99.7% **DHPAFAKEYQSATIDFSTETFVYEVSEARTFGFMKDLDYLKANNLALGASLDNAIGVDDTGVVNEEGLRFADEFVRHKIL**

7 259_an 100.0% 99.7% **DHPAFAKEYQSATIDFSTETFVYEVSEARTFGFMKDLDYLKANNLALGASLDNAIGVDDTGVVNEEGLRFADEFVRHKIL**

8 276_ax 100.0% 99.7% **DHPAFAKEYQSATIDFSTETFVYEVSEARTFGFMKDLDYLKANNLALGASLDNAIGVDDTGVVNEEGLRFADEFVRHKIL**

9 PT061 100.0% 99.7% **DHPAFAKEYQSATIDFSTETFVYEVSEARTFGFMKDLDYLKANNLALGASLDNAIGVDDTGVVNEEGLRFADEFVRHKIL**

10 PT003 100.0% 99.7% **DHPAFAKEYQSATIDFSTETFVYEVSEARTFGFMKDLDYLKANNLALGASLDNAIGVDDTGVVNEEGLRFADEFVRHKIL**

11 PSU068 100.0% 99.7% **DHPAFAKEYQSATIDFSTETFVYEVSEARTFGFMKDLDYLKANNLALGASLDNAIGVDDTGVVNEEGLRFADEFVRHKIL**

12 PSU073 100.0% 99.7% **DHPAFAKEYQSATIDFSTETFVYEVSEARTFGFMKDLDYLKANNLALGASLDNAIGVDDTGVVNEEGLRFADEFVRHKIL**

13 CCBH26501 100.0% 99.7% **DHPAFAKEYQSATIDFSTETFVYEVSEARTFGFMKDLDYLKANNLALGASLDNAIGVDDTGVVNEEGLRFADEFVRHKIL**

14 AB363 100.0% 99.7% **DHPAFAKEYQSATIDFSTETFVYEVSEARTFGFMKDLDYLKANNLALGASLDNAIGVDDTGVVNEEGLRFADEFVRHKIL**

15 KUSSH08 100.0% 99.7% **DHPAFAKEYQSATIDFSTETFVYEVSEARTFGFMKDLDYLKANNLALGASLDNAIGVDDTGVVNEEGLRFADEFVRHKIL**

16 KUSSH14 100.0% 99.7% **DHPAFAKEYQSATIDFSTETFVYEVSEARTFGFMKDLDYLKANNLALGASLDNAIGVDDTGVVNEEGLRFADEFVRHKIL**

17 TUMA 100.0% 99.7% **DHPAFAKEYQSATIDFSTETFVYEVSEARTFGFMKDLDYLKANNLALGASLDNAIGVDDTGVVNEEGLRFADEFVRHKIL**

18 4300STDY7045886 100.0% 99.7% **DHPAFAKEYQSATIDFSTETFVYEVSEARTFGFMKDLDYLKANNLALGASLDNAIGVDDTGVVNEEGLRFADEFVRHKIL**

19 Aci00866 100.0% 99.7% **DHPAFAKEYQSATIDFSTETFVYEVSEARTFGFMKDLDYLKANNLALGASLDNAIGVDDTGVVNEEGLRFADEFVRHKIL**

20 Aci00860 100.0% 99.7% **DHPAFAKEYQSATIDFSTETFVYEVSEARTFGFMKDLDYLKANNLALGASLDNAIGVDDTGVVNEEGLRFADEFVRHKIL**

21 Aci00848 100.0% 99.7% **DHPAFAKEYQSATIDFSTETFVYEVSEARTFGFMKDLDYLKANNLALGASLDNAIGVDDTGVVNEEGLRFADEFVRHKIL**

22 MRSN351524 100.0% 99.7% **DHPAFAKEYQSATIDFSTETFVYEVSEARTFGFMKDLDYLKANNLALGASLDNAIGVDDTGVVNEEGLRFADEFVRHKIL**

cov pid **241**  **: . . . . ]** **300**

1 ATCC19606 100.0% 100.0% **DAVGDLYLLGHQIIAKFDGYKSGHALNNQLLRNVQSDPSNYEIVTFNDEKDCPIPYVSVT**

2 AB_1649-8 100.0% 99.7% **DAVGDLYLLGHQIIAKFDGYKSGHALNNQLLRNVQSDPSNYEIVTFDDEKDCPIPYVSVT**

3 AB_1650-8 100.0% 99.7% **DAVGDLYLLGHQIIAKFDGYKSGHALNNQLLRNVQSDPSNYEIVTFDDEKDCPIPYVSVT**

4 M14 100.0% 99.7% **DAVGDLYLLGHQIIAKFDGYKSGHALNNQLLRNVQSDPSNYEIVTFDDEKDCPIPYVSVT**

5 M03 100.0% 99.7% **DAVGDLYLLGHQIIAKFDGYKSGHALNNQLLRNVQSDPSNYEIVTFDDEKDCPIPYVSVT**

6 UV_1036 100.0% 99.7% **DAVGDLYLLGHQIIAKFDGYKSGHALNNQLLRNVQSDPSNYEIVTFDDEKDCPIPYVSVT**

7 259_an 100.0% 99.7% **DAVGDLYLLGHQIIAKFDGYKSGHALNNQLLRNVQSDPSNYEIVTFDDEKDCPIPYVSVT**

8 276_ax 100.0% 99.7% **DAVGDLYLLGHQIIAKFDGYKSGHALNNQLLRNVQSDPSNYEIVTFDDEKDCPIPYVSVT**

9 PT061 100.0% 99.7% **DAVGDLYLLGHQIIAKFDGYKSGHALNNQLLRNVQSDPSNYEIVTFDDEKDCPIPYVSVT**

10 PT003 100.0% 99.7% **DAVGDLYLLGHQIIAKFDGYKSGHALNNQLLRNVQSDPSNYEIVTFDDEKDCPIPYVSVT**

11 PSU068 100.0% 99.7% **DAVGDLYLLGHQIIAKFDGYKSGHALNNQLLRNVQSDPSNYEIVTFDDEKDCPIPYVSVT**

12 PSU073 100.0% 99.7% **DAVGDLYLLGHQIIAKFDGYKSGHALNNQLLRNVQSDPSNYEIVTFDDEKDCPIPYVSVT**

13 CCBH26501 100.0% 99.7% **DAVGDLYLLGHQIIAKFDGYKSGHALNNQLLRNVQSDPSNYEIVTFDDEKDCPIPYVSVT**

14 AB363 100.0% 99.7% **DAVGDLYLLGHQIIAKFDGYKSGHALNNQLLRNVQSDPSNYEIVTFDDEKDCPIPYVSVT**

15 KUSSH08 100.0% 99.7% **DAVGDLYLLGHQIIAKFDGYKSGHALNNQLLRNVQSDPSNYEIVTFDDEKDCPIPYVSVT**

16 KUSSH14 100.0% 99.7% **DAVGDLYLLGHQIIAKFDGYKSGHALNNQLLRNVQSDPSNYEIVTFDDEKDCPIPYVSVT**

17 TUMA 100.0% 99.7% **DAVGDLYLLGHQIIAKFDGYKSGHALNNQLLRNVQSDPSNYEIVTFDDEKDCPIPYVSVT**

18 4300STDY7045886 100.0% 99.7% **DAVGDLYLLGHQIIAKFDGYKSGHALNNQLLRNVQSDPSNYEIVTFDDEKDCPIPYVSVT**

19 Aci00866 100.0% 99.7% **DAVGDLYLLGHQIIAKFDGYKSGHALNNQLLRNVQSDPSNYEIVTFDDEKDCPIPYVSVT**

20 Aci00860 100.0% 99.7% **DAVGDLYLLGHQIIAKFDGYKSGHALNNQLLRNVQSDPSNYEIVTFDDEKDCPIPYVSVT**

21 Aci00848 100.0% 99.7% **DAVGDLYLLGHQIIAKFDGYKSGHALNNQLLRNVQSDPSNYEIVTFDDEKDCPIPYVSVT**

22 MRSN351524 100.0% 99.7% **DAVGDLYLLGHQIIAKFDGYKSGHALNNQLLRNVQSDPSNYEIVTFDDEKDCPIPYVSVT**

**Supplementary Figure 13: Multiple sequence alignment (MSA) of the predicted amino acid sequence of LpxC carried by ST113^Pas^ (GC7) and close genomes retrieved from the BV-BRC database compared to the respective gene in *A. baumannii* ATCC 19606.** MSA was created by the A multiple alignment viewer MView hosted by the EMBL-EBI; cov, coverage; pid, percent identity.

cov pid  **1** **[ . . . . : . . .** **80**

1 ATCC19606 100.0% 100.0% **MVKQRTLNRVVKASGIGLHSGQKVMINFIPHTVDGGIVFRRIDLDPPVDIPANALLIQEAFMCSNLVTGDIKVGTIEHVM**

2 M02 100.0% 99.7% **MVKQRTLNRVVKASGIGLHSGQKVMINFIPHTVDGGIVFRRIDLDPPVDIPANALLIQEAFMCSNLVTGDIKVGTIEHVM**

3 M11 100.0% 99.7% **MVKQRTLNRVVKASGIGLHSGQKVMINFIPHTVDGGIVFRRIDLDPPVDIPANALLIQEAFMCSNLVTGDIKVGTIEHVM**

4 M18 100.0% 99.7% **MVKQRTLNRVVKASGIGLHSGQKVMINFIPHTVDGGIVFRRIDLDPPVDIPANALLIQEAFMCSNLVTGDIKVGTIEHVM**

5 15953 100.0% 99.7% **MVKQRTLNRVVKASGIGLHSGQKVMINFIPHTVDGGIVFRRIDLDPPVDIPANALLIQEAFMCSNLVTGDIKVGTIEHVM**

6 15946 100.0% 99.7% **MVKQRTLNRVVKASGIGLHSGQKVMINFIPHTVDGGIVFRRIDLDPPVDIPANALLIQEAFMCSNLVTGDIKVGTIEHVM**

7 Survcare112 100.0% 99.7% **MVKQRTLNRVVKASGIGLHSGQKVMINFIPHTVDGGIVFRRIDLDPPVDIPANALLIQEAFMCSNLVTGDIKVGTIEHVM**

8 Cl300 100.0% 99.7% **MVKQRTLNRVVKASGIGLHSGQKVMINFIPHTVDGGIVFRRIDLDPPVDIPANALLIQEAFMCSNLVTGDIKVGTIEHVM**

9 17A1955 100.0% 99.7% **MVKQRTLNRVVKASGIGLHSGQKVMINFIPHTVDGGIVFRRIDLDPPVDIPANALLIQEAFMCSNLVTGDIKVGTIEHVM**

10 ACMH-6200 100.0% 99.7% **MVKQRTLNRVVKASGIGLHSGQKVMINFIPHTVDGGIVFRRIDLDPPVDIPANALLIQEAFMCSNLVTGDIKVGTIEHVM**

11 ACMH-6201 100.0% 99.7% **MVKQRTLNRVVKASGIGLHSGQKVMINFIPHTVDGGIVFRRIDLDPPVDIPANALLIQEAFMCSNLVTGDIKVGTIEHVM**

12 MIN-015 100.0% 99.7% **MVKQRTLNRVVKASGIGLHSGQKVMINFIPHTVDGGIVFRRIDLDPPVDIPANALLIQEAFMCSNLVTGDIKVGTIEHVM**

13 17A1955 100.0% 99.7% **MVKQRTLNRVVKASGIGLHSGQKVMINFIPHTVDGGIVFRRIDLDPPVDIPANALLIQEAFMCSNLVTGDIKVGTIEHVM**

14 AB177-VUB 100.0% 99.7% **MVKQRTLNRVVKASGIGLHSGQKVMINFIPHTVDGGIVFRRIDLDPPVDIPANALLIQEAFMCSNLVTGDIKVGTIEHVM**

15 P116A 100.0% 99.7% **MVKQRTLNRVVKASGIGLHSGQKVMINFIPHTVDGGIVFRRIDLDPPVDIPANALLIQEAFMCSNLVTGDIKVGTIEHVM**

16 26 100.0% 99.7% **MVKQRTLNRVVKASGIGLHSGQKVMINFIPHTVDGGIVFRRIDLDPPVDIPANALLIQEAFMCSNLVTGDIKVGTIEHVM**

17 AR_0037 100.0% 99.7% **MVKQRTLNRVVKASGIGLHSGQKVMINFIPHTVDGGIVFRRIDLDPPVDIPANALLIQEAFMCSNLVTGDIKVGTIEHVM**

18 AR_0033 100.0% 99.7% **MVKQRTLNRVVKASGIGLHSGQKVMINFIPHTVDGGIVFRRIDLDPPVDIPANALLIQEAFMCSNLVTGDIKVGTIEHVM**

19 AB-A 100.0% 99.7% **MVKQRTLNRVVKASGIGLHSGQKVMINFIPHTVDGGIVFRRIDLDPPVDIPANALLIQEAFMCSNLVTGDIKVGTIEHVM**

20 AB-C 100.0% 99.7% **MVKQRTLNRVVKASGIGLHSGQKVMINFIPHTVDGGIVFRRIDLDPPVDIPANALLIQEAFMCSNLVTGDIKVGTIEHVM**

21 AB-B 100.0% 99.7% **MVKQRTLNRVVKASGIGLHSGQKVMINFIPHTVDGGIVFRRIDLDPPVDIPANALLIQEAFMCSNLVTGDIKVGTIEHVM**

22 AE27M 100.0% 99.7% **MVKQRTLNRVVKASGIGLHSGQKVMINFIPHTVDGGIVFRRIDLDPPVDIPANALLIQEAFMCSNLVTGDIKVGTIEHVM**

23 AE3M 100.0% 99.7% **MVKQRTLNRVVKASGIGLHSGQKVMINFIPHTVDGGIVFRRIDLDPPVDIPANALLIQEAFMCSNLVTGDIKVGTIEHVM**

24 MBL_M1 100.0% 99.7% **MVKQRTLNRVVKASGIGLHSGQKVMINFIPHTVDGGIVFRRIDLDPPVDIPANALLIQEAFMCSNLVTGDIKVGTIEHVM**

25 MBL_M6 100.0% 99.7% **MVKQRTLNRVVKASGIGLHSGQKVMINFIPHTVDGGIVFRRIDLDPPVDIPANALLIQEAFMCSNLVTGDIKVGTIEHVM**

26 MBL_M10 100.0% 99.7% **MVKQRTLNRVVKASGIGLHSGQKVMINFIPHTVDGGIVFRRIDLDPPVDIPANALLIQEAFMCSNLVTGDIKVGTIEHVM**

27 MBL_M9 100.0% 99.7% **MVKQRTLNRVVKASGIGLHSGQKVMINFIPHTVDGGIVFRRIDLDPPVDIPANALLIQEAFMCSNLVTGDIKVGTIEHVM**

28 R11 100.0% 99.7% **MVKQRTLNRVVKASGIGLHSGQKVMINFIPHTVDGGIVFRRIDLDPPVDIPANALLIQEAFMCSNLVTGDIKVGTIEHVM**

29 Ab-NDM-1 100.0% 99.7% **MVKQRTLNRVVKASGIGLHSGQKVMINFIPHTVDGGIVFRRIDLDPPVDIPANALLIQEAFMCSNLVTGDIKVGTIEHVM**

30 MRSN15574 100.0% 99.7% **MVKQRTLNRVVKASGIGLHSGQKVMINFIPHTVDGGIVFRRIDLDPPVDIPANALLIQEAFMCSNLVTGDIKVGTIEHVM**

cov pid  **81**  **. 1 . . . . : .** **160**

1 ATCC19606 100.0% 100.0% **SAIAGLGIDNLIVEVSASEVPIMDGSAGPFIYLLMQGGLREQDAPKKFIKILKPVEALIDDKKAIFSPHNGFQLNFTIDF**

2 M02 100.0% 99.7% **SAIAGLGIDNLIVEVSASEVPIMDGSAGPFIYLLMQGGLREQDAPKKFIKILKPVEALIDDKKAIFSPHNGFQLNFTIDF**

3 M11 100.0% 99.7% **SAIAGLGIDNLIVEVSASEVPIMDGSAGPFIYLLMQGGLREQDAPKKFIKILKPVEALIDDKKAIFSPHNGFQLNFTIDF**

4 M18 100.0% 99.7% **SAIAGLGIDNLIVEVSASEVPIMDGSAGPFIYLLMQGGLREQDAPKKFIKILKPVEALIDDKKAIFSPHNGFQLNFTIDF**

5 15953 100.0% 99.7% **SAIAGLGIDNLIVEVSASEVPIMDGSAGPFIYLLMQGGLREQDAPKKFIKILKPVEALIDDKKAIFSPHNGFQLNFTIDF**

6 15946 100.0% 99.7% **SAIAGLGIDNLIVEVSASEVPIMDGSAGPFIYLLMQGGLREQDAPKKFIKILKPVEALIDDKKAIFSPHNGFQLNFTIDF**

7 Survcare112 100.0% 99.7% **SAIAGLGIDNLIVEVSASEVPIMDGSAGPFIYLLMQGGLREQDAPKKFIKILKPVEALIDDKKAIFSPHNGFQLNFTIDF**

8 Cl300 100.0% 99.7% **SAIAGLGIDNLIVEVSASEVPIMDGSAGPFIYLLMQGGLREQDAPKKFIKILKPVEALIDDKKAIFSPHNGFQLNFTIDF**

9 17A1955 100.0% 99.7% **SAIAGLGIDNLIVEVSASEVPIMDGSAGPFIYLLMQGGLREQDAPKKFIKILKPVEALIDDKKAIFSPHNGFQLNFTIDF**

10 ACMH-6200 100.0% 99.7% **SAIAGLGIDNLIVEVSASEVPIMDGSAGPFIYLLMQGGLREQDAPKKFIKILKPVEALIDDKKAIFSPHNGFQLNFTIDF**

11 ACMH-6201 100.0% 99.7% **SAIAGLGIDNLIVEVSASEVPIMDGSAGPFIYLLMQGGLREQDAPKKFIKILKPVEALIDDKKAIFSPHNGFQLNFTIDF**

12 MIN-015 100.0% 99.7% **SAIAGLGIDNLIVEVSASEVPIMDGSAGPFIYLLMQGGLREQDAPKKFIKILKPVEALIDDKKAIFSPHNGFQLNFTIDF**

13 17A1955 100.0% 99.7% **SAIAGLGIDNLIVEVSASEVPIMDGSAGPFIYLLMQGGLREQDAPKKFIKILKPVEALIDDKKAIFSPHNGFQLNFTIDF**

14 AB177-VUB 100.0% 99.7% **SAIAGLGIDNLIVEVSASEVPIMDGSAGPFIYLLMQGGLREQDAPKKFIKILKPVEALIDDKKAIFSPHNGFQLNFTIDF**

15 P116A 100.0% 99.7% **SAIAGLGIDNLIVEVSASEVPIMDGSAGPFIYLLMQGGLREQDAPKKFIKILKPVEALIDDKKAIFSPHNGFQLNFTIDF**

16 26 100.0% 99.7% **SAIAGLGIDNLIVEVSASEVPIMDGSAGPFIYLLMQGGLREQDAPKKFIKILKPVEALIDDKKAIFSPHNGFQLNFTIDF**

17 AR_0037 100.0% 99.7% **SAIAGLGIDNLIVEVSASEVPIMDGSAGPFIYLLMQGGLREQDAPKKFIKILKPVEALIDDKKAIFSPHNGFQLNFTIDF**

18 AR_0033 100.0% 99.7% **SAIAGLGIDNLIVEVSASEVPIMDGSAGPFIYLLMQGGLREQDAPKKFIKILKPVEALIDDKKAIFSPHNGFQLNFTIDF**

19 AB-A 100.0% 99.7% **SAIAGLGIDNLIVEVSASEVPIMDGSAGPFIYLLMQGGLREQDAPKKFIKILKPVEALIDDKKAIFSPHNGFQLNFTIDF**

20 AB-C 100.0% 99.7% **SAIAGLGIDNLIVEVSASEVPIMDGSAGPFIYLLMQGGLREQDAPKKFIKILKPVEALIDDKKAIFSPHNGFQLNFTIDF**

21 AB-B 100.0% 99.7% **SAIAGLGIDNLIVEVSASEVPIMDGSAGPFIYLLMQGGLREQDAPKKFIKILKPVEALIDDKKAIFSPHNGFQLNFTIDF**

22 AE27M 100.0% 99.7% **SAIAGLGIDNLIVEVSASEVPIMDGSAGPFIYLLMQGGLREQDAPKKFIKILKPVEALIDDKKAIFSPHNGFQLNFTIDF**

23 AE3M 100.0% 99.7% **SAIAGLGIDNLIVEVSASEVPIMDGSAGPFIYLLMQGGLREQDAPKKFIKILKPVEALIDDKKAIFSPHNGFQLNFTIDF**

24 MBL_M1 100.0% 99.7% **SAIAGLGIDNLIVEVSASEVPIMDGSAGPFIYLLMQGGLREQDAPKKFIKILKPVEALIDDKKAIFSPHNGFQLNFTIDF**

25 MBL_M6 100.0% 99.7% **SAIAGLGIDNLIVEVSASEVPIMDGSAGPFIYLLMQGGLREQDAPKKFIKILKPVEALIDDKKAIFSPHNGFQLNFTIDF**

26 MBL_M10 100.0% 99.7% **SAIAGLGIDNLIVEVSASEVPIMDGSAGPFIYLLMQGGLREQDAPKKFIKILKPVEALIDDKKAIFSPHNGFQLNFTIDF**

27 MBL_M9 100.0% 99.7% **SAIAGLGIDNLIVEVSASEVPIMDGSAGPFIYLLMQGGLREQDAPKKFIKILKPVEALIDDKKAIFSPHNGFQLNFTIDF**

28 R11 100.0% 99.7% **SAIAGLGIDNLIVEVSASEVPIMDGSAGPFIYLLMQGGLREQDAPKKFIKILKPVEALIDDKKAIFSPHNGFQLNFTIDF**

29 Ab-NDM-1 100.0% 99.7% **SAIAGLGIDNLIVEVSASEVPIMDGSAGPFIYLLMQGGLREQDAPKKFIKILKPVEALIDDKKAIFSPHNGFQLNFTIDF**

30 MRSN15574 100.0% 99.7% **SAIAGLGIDNLIVEVSASEVPIMDGSAGPFIYLLMQGGLREQDAPKKFIKILKPVEALIDDKKAIFSPHNGFQLNFTIDF**

cov pid **161**  **. . . 2 . . . .** **240**

1 ATCC19606 100.0% 100.0% **DHPAFAKEYQSATIDFSTETFVYEVSEARTFGFMKDLDYLKANNLALGASLDNAIGVDDTGVVNEEGLRFADEFVRHKIL**

2 M02 100.0% 99.7% **DHPAFAKEYQSATIDFSTETFVYEVSEARTFGFMKDLDYLKANNLALGASLDNAIGVDDTGVVNEEGLRFADEFVRHKIL**

3 M11 100.0% 99.7% **DHPAFAKEYQSATIDFSTETFVYEVSEARTFGFMKDLDYLKANNLALGASLDNAIGVDDTGVVNEEGLRFADEFVRHKIL**

4 M18 100.0% 99.7% **DHPAFAKEYQSATIDFSTETFVYEVSEARTFGFMKDLDYLKANNLALGASLDNAIGVDDTGVVNEEGLRFADEFVRHKIL**

5 15953 100.0% 99.7% **DHPAFAKEYQSATIDFSTETFVYEVSEARTFGFMKDLDYLKANNLALGASLDNAIGVDDTGVVNEEGLRFADEFVRHKIL**

6 15946 100.0% 99.7% **DHPAFAKEYQSATIDFSTETFVYEVSEARTFGFMKDLDYLKANNLALGASLDNAIGVDDTGVVNEEGLRFADEFVRHKIL**

7 Survcare112 100.0% 99.7% **DHPAFAKEYQSATIDFSTETFVYEVSEARTFGFMKDLDYLKANNLALGASLDNAIGVDDTGVVNEEGLRFADEFVRHKIL**

8 Cl300 100.0% 99.7% **DHPAFAKEYQSATIDFSTETFVYEVSEARTFGFMKDLDYLKANNLALGASLDNAIGVDDTGVVNEEGLRFADEFVRHKIL**

9 17A1955 100.0% 99.7% **DHPAFAKEYQSATIDFSTETFVYEVSEARTFGFMKDLDYLKANNLALGASLDNAIGVDDTGVVNEEGLRFADEFVRHKIL**

10 ACMH-6200 100.0% 99.7% **DHPAFAKEYQSATIDFSTETFVYEVSEARTFGFMKDLDYLKANNLALGASLDNAIGVDDTGVVNEEGLRFADEFVRHKIL**

11 ACMH-6201 100.0% 99.7% **DHPAFAKEYQSATIDFSTETFVYEVSEARTFGFMKDLDYLKANNLALGASLDNAIGVDDTGVVNEEGLRFADEFVRHKIL**

12 MIN-015 100.0% 99.7% **DHPAFAKEYQSATIDFSTETFVYEVSEARTFGFMKDLDYLKANNLALGASLDNAIGVDDTGVVNEEGLRFADEFVRHKIL**

13 17A1955 100.0% 99.7% **DHPAFAKEYQSATIDFSTETFVYEVSEARTFGFMKDLDYLKANNLALGASLDNAIGVDDTGVVNEEGLRFADEFVRHKIL**

14 AB177-VUB 100.0% 99.7% **DHPAFAKEYQSATIDFSTETFVYEVSEARTFGFMKDLDYLKANNLALGASLDNAIGVDDTGVVNEEGLRFADEFVRHKIL**

15 P116A 100.0% 99.7% **DHPAFAKEYQSATIDFSTETFVYEVSEARTFGFMKDLDYLKANNLALGASLDNAIGVDDTGVVNEEGLRFADEFVRHKIL**

16 26 100.0% 99.7% **DHPAFAKEYQSATIDFSTETFVYEVSEARTFGFMKDLDYLKANNLALGASLDNAIGVDDTGVVNEEGLRFADEFVRHKIL**

17 AR_0037 100.0% 99.7% **DHPAFAKEYQSATIDFSTETFVYEVSEARTFGFMKDLDYLKANNLALGASLDNAIGVDDTGVVNEEGLRFADEFVRHKIL**

18 AR_0033 100.0% 99.7% **DHPAFAKEYQSATIDFSTETFVYEVSEARTFGFMKDLDYLKANNLALGASLDNAIGVDDTGVVNEEGLRFADEFVRHKIL**

19 AB-A 100.0% 99.7% **DHPAFAKEYQSATIDFSTETFVYEVSEARTFGFMKDLDYLKANNLALGASLDNAIGVDDTGVVNEEGLRFADEFVRHKIL**

20 AB-C 100.0% 99.7% **DHPAFAKEYQSATIDFSTETFVYEVSEARTFGFMKDLDYLKANNLALGASLDNAIGVDDTGVVNEEGLRFADEFVRHKIL**

21 AB-B 100.0% 99.7% **DHPAFAKEYQSATIDFSTETFVYEVSEARTFGFMKDLDYLKANNLALGASLDNAIGVDDTGVVNEEGLRFADEFVRHKIL**

22 AE27M 100.0% 99.7% **DHPAFAKEYQSATIDFSTETFVYEVSEARTFGFMKDLDYLKANNLALGASLDNAIGVDDTGVVNEEGLRFADEFVRHKIL**

23 AE3M 100.0% 99.7% **DHPAFAKEYQSATIDFSTETFVYEVSEARTFGFMKDLDYLKANNLALGASLDNAIGVDDTGVVNEEGLRFADEFVRHKIL**

24 MBL_M1 100.0% 99.7% **DHPAFAKEYQSATIDFSTETFVYEVSEARTFGFMKDLDYLKANNLALGASLDNAIGVDDTGVVNEEGLRFADEFVRHKIL**

25 MBL_M6 100.0% 99.7% **DHPAFAKEYQSATIDFSTETFVYEVSEARTFGFMKDLDYLKANNLALGASLDNAIGVDDTGVVNEEGLRFADEFVRHKIL**

26 MBL_M10 100.0% 99.7% **DHPAFAKEYQSATIDFSTETFVYEVSEARTFGFMKDLDYLKANNLALGASLDNAIGVDDTGVVNEEGLRFADEFVRHKIL**

27 MBL_M9 100.0% 99.7% **DHPAFAKEYQSATIDFSTETFVYEVSEARTFGFMKDLDYLKANNLALGASLDNAIGVDDTGVVNEEGLRFADEFVRHKIL**

28 R11 100.0% 99.7% **DHPAFAKEYQSATIDFSTETFVYEVSEARTFGFMKDLDYLKANNLALGASLDNAIGVDDTGVVNEEGLRFADEFVRHKIL**

29 Ab-NDM-1 100.0% 99.7% **DHPAFAKEYQSATIDFSTETFVYEVSEARTFGFMKDLDYLKANNLALGASLDNAIGVDDTGVVNEEGLRFADEFVRHKIL**

30 MRSN15574 100.0% 99.7% **DHPAFAKEYQSATIDFSTETFVYEVSEARTFGFMKDLDYLKANNLALGASLDNAIGVDDTGVVNEEGLRFADEFVRHKIL**

cov pid **241**  **: . . . . ]** **300**

1 ATCC19606 100.0% 100.0% **DAVGDLYLLGHQIIAKFDGYKSGHALNNQLLRNVQSDPSNYEIVTFNDEKDCPIPYVSVT**

2 M02 100.0% 99.7% **DAVGDLYLLGHQIIAKFDGYKSGHALNNQLLRNVQSDPSNYEIVTFDDEKDCPIPYVSVT**

3 M11 100.0% 99.7% **DAVGDLYLLGHQIIAKFDGYKSGHALNNQLLRNVQSDPSNYEIVTFDDEKDCPIPYVSVT**

4 M18 100.0% 99.7% **DAVGDLYLLGHQIIAKFDGYKSGHALNNQLLRNVQSDPSNYEIVTFDDEKDCPIPYVSVT**

5 15953 100.0% 99.7% **DAVGDLYLLGHQIIAKFDGYKSGHALNNQLLRNVQSDPSNYEIVTFDDEKDCPIPYVSVT**

6 15946 100.0% 99.7% **DAVGDLYLLGHQIIAKFDGYKSGHALNNQLLRNVQSDPSNYEIVTFDDEKDCPIPYVSVT**

7 Survcare112 100.0% 99.7% **DAVGDLYLLGHQIIAKFDGYKSGHALNNQLLRNVQSDPSNYEIVTFDDEKDCPIPYVSVT**

8 Cl300 100.0% 99.7% **DAVGDLYLLGHQIIAKFDGYKSGHALNNQLLRNVQSDPSNYEIVTFDDEKDCPIPYVSVT**

9 17A1955 100.0% 99.7% **DAVGDLYLLGHQIIAKFDGYKSGHALNNQLLRNVQSDPSNYEIVTFDDEKDCPIPYVSVT**

10 ACMH-6200 100.0% 99.7% **DAVGDLYLLGHQIIAKFDGYKSGHALNNQLLRNVQSDPSNYEIVTFDDEKDCPIPYVSVT**

11 ACMH-6201 100.0% 99.7% **DAVGDLYLLGHQIIAKFDGYKSGHALNNQLLRNVQSDPSNYEIVTFDDEKDCPIPYVSVT**

12 MIN-015 100.0% 99.7% **DAVGDLYLLGHQIIAKFDGYKSGHALNNQLLRNVQSDPSNYEIVTFDDEKDCPIPYVSVT**

13 17A1955 100.0% 99.7% **DAVGDLYLLGHQIIAKFDGYKSGHALNNQLLRNVQSDPSNYEIVTFDDEKDCPIPYVSVT**

14 AB177-VUB 100.0% 99.7% **DAVGDLYLLGHQIIAKFDGYKSGHALNNQLLRNVQSDPSNYEIVTFDDEKDCPIPYVSVT**

15 P116A 100.0% 99.7% **DAVGDLYLLGHQIIAKFDGYKSGHALNNQLLRNVQSDPSNYEIVTFDDEKDCPIPYVSVT**

16 26 100.0% 99.7% **DAVGDLYLLGHQIIAKFDGYKSGHALNNQLLRNVQSDPSNYEIVTFDDEKDCPIPYVSVT**

17 AR_0037 100.0% 99.7% **DAVGDLYLLGHQIIAKFDGYKSGHALNNQLLRNVQSDPSNYEIVTFDDEKDCPIPYVSVT**

18 AR_0033 100.0% 99.7% **DAVGDLYLLGHQIIAKFDGYKSGHALNNQLLRNVQSDPSNYEIVTFDDEKDCPIPYVSVT**

19 AB-A 100.0% 99.7% **DAVGDLYLLGHQIIAKFDGYKSGHALNNQLLRNVQSDPSNYEIVTFDDEKDCPIPYVSVT**

20 AB-C 100.0% 99.7% **DAVGDLYLLGHQIIAKFDGYKSGHALNNQLLRNVQSDPSNYEIVTFDDEKDCPIPYVSVT**

21 AB-B 100.0% 99.7% **DAVGDLYLLGHQIIAKFDGYKSGHALNNQLLRNVQSDPSNYEIVTFDDEKDCPIPYVSVT**

22 AE27M 100.0% 99.7% **DAVGDLYLLGHQIIAKFDGYKSGHALNNQLLRNVQSDPSNYEIVTFDDEKDCPIPYVSVT**

23 AE3M 100.0% 99.7% **DAVGDLYLLGHQIIAKFDGYKSGHALNNQLLRNVQSDPSNYEIVTFDDEKDCPIPYVSVT**

24 MBL_M1 100.0% 99.7% **DAVGDLYLLGHQIIAKFDGYKSGHALNNQLLRNVQSDPSNYEIVTFDDEKDCPIPYVSVT**

25 MBL_M6 100.0% 99.7% **DAVGDLYLLGHQIIAKFDGYKSGHALNNQLLRNVQSDPSNYEIVTFDDEKDCPIPYVSVT**

26 MBL_M10 100.0% 99.7% **DAVGDLYLLGHQIIAKFDGYKSGHALNNQLLRNVQSDPSNYEIVTFDDEKDCPIPYVSVT**

27 MBL_M9 100.0% 99.7% **DAVGDLYLLGHQIIAKFDGYKSGHALNNQLLRNVQSDPSNYEIVTFDDEKDCPIPYVSVT**

28 R11 100.0% 99.7% **DAVGDLYLLGHQIIAKFDGYKSGHALNNQLLRNVQSDPSNYEIVTFDDEKDCPIPYVSVT**

29 Ab-NDM-1 100.0% 99.7% **DAVGDLYLLGHQIIAKFDGYKSGHALNNQLLRNVQSDPSNYEIVTFDDEKDCPIPYVSVT**

30 MRSN15574 100.0% 99.7% **DAVGDLYLLGHQIIAKFDGYKSGHALNNQLLRNVQSDPSNYEIVTFDDEKDCPIPYVSVT**

**Supplementary Figure 14: Multiple sequence alignment (MSA) of the predicted amino acid sequence of LpxC carried by ST85^Pas^ (GC9) and close genomes retrieved from the BV-BRC database compared to the respective gene in *A. baumannii* ATCC 19606.** MSA was created by the A multiple alignment viewer MView hosted by the EMBL-EBI; cov, coverage; pid, percent identity.

cov pid  **1** **[ . . . . : . . .** **80**

1 ATCC19606 100.0% 100.0% **MVKQRTLNRVVKASGIGLHSGQKVMINFIPHTVDGGIVFRRIDLDPPVDIPANALLIQEAFMCSNLVTGDIKVGTIEHVM**

2 M19 100.0% 99.7% **MVKQRTLNRVVKASGIGLHSGQKVMINFIPHTVDGGIVFRRIDLDPPVDIPANALLIQEAFMCSNLVTGDIKVGTIEHVM**

3 A5 100.0% 99.7% **MVKQRTLNRVVKASGIGLHSGQKVMINFIPHTVDGGIVFRRIDLDPPVDIPANALLIQEAFMCSNLVTGDIKVGTIEHVM**

4 Aci00709 100.0% 99.7% **MVKQRTLNRVVKASGIGLHSGQKVMINFIPHTVDGGIVFRRIDLDPPVDIPANALLIQEAFMCSNLVTGDIKVGTIEHVM**

5 Aci00711 100.0% 99.7% **MVKQRTLNRVVKASGIGLHSGQKVMINFIPHTVDGGIVFRRIDLDPPVDIPANALLIQEAFMCSNLVTGDIKVGTIEHVM**

6 KAB15 100.0% 99.7% **MVKQRTLNRVVKASGIGLHSGQKVMINFIPHTVDGGIVFRRIDLDPPVDIPANALLIQEAFMCSNLVTGDIKVGTIEHVM**

7 PT022 100.0% 99.7% **MVKQRTLNRVVKASGIGLHSGQKVMINFIPHTVDGGIVFRRIDLDPPVDIPANALLIQEAFMCSNLVTGDIKVGTIEHVM**

8 PSU091 100.0% 99.7% **MVKQRTLNRVVKASGIGLHSGQKVMINFIPHTVDGGIVFRRIDLDPPVDIPANALLIQEAFMCSNLVTGDIKVGTIEHVM**

9 151 100.0% 99.7% **MVKQRTLNRVVKASGIGLHSGQKVMINFIPHTVDGGIVFRRIDLDPPVDIPANALLIQEAFMCSNLVTGDIKVGTIEHVM**

10 GML-KP48-AB-TR 100.0% 99.7% **MVKQRTLNRVVKASGIGLHSGQKVMINFIPHTVDGGIVFRRIDLDPPVDIPANALLIQEAFMCSNLVTGDIKVGTIEHVM**

11 198 100.0% 99.7% **MVKQRTLNRVVKASGIGLHSGQKVMINFIPHTVDGGIVFRRIDLDPPVDIPANALLIQEAFMCSNLVTGDIKVGTIEHVM**

12 4300STDY7045706 100.0% 99.7% **MVKQRTLNRVVKASGIGLHSGQKVMINFIPHTVDGGIVFRRIDLDPPVDIPANALLIQEAFMCSNLVTGDIKVGTIEHVM**

13 4300STDY7045763 100.0% 99.7% **MVKQRTLNRVVKASGIGLHSGQKVMINFIPHTVDGGIVFRRIDLDPPVDIPANALLIQEAFMCSNLVTGDIKVGTIEHVM**

14 4300STDY7045798 100.0% 99.7% **MVKQRTLNRVVKASGIGLHSGQKVMINFIPHTVDGGIVFRRIDLDPPVDIPANALLIQEAFMCSNLVTGDIKVGTIEHVM**

15 4300STDY7045799 100.0% 99.7% **MVKQRTLNRVVKASGIGLHSGQKVMINFIPHTVDGGIVFRRIDLDPPVDIPANALLIQEAFMCSNLVTGDIKVGTIEHVM**

16 4300STDY7045808 100.0% 99.7% **MVKQRTLNRVVKASGIGLHSGQKVMINFIPHTVDGGIVFRRIDLDPPVDIPANALLIQEAFMCSNLVTGDIKVGTIEHVM**

17 4300STDY7045806 100.0% 99.7% **MVKQRTLNRVVKASGIGLHSGQKVMINFIPHTVDGGIVFRRIDLDPPVDIPANALLIQEAFMCSNLVTGDIKVGTIEHVM**

18 4300STDY7045811 100.0% 99.7% **MVKQRTLNRVVKASGIGLHSGQKVMINFIPHTVDGGIVFRRIDLDPPVDIPANALLIQEAFMCSNLVTGDIKVGTIEHVM**

19 4300STDY7045829 100.0% 99.7% **MVKQRTLNRVVKASGIGLHSGQKVMINFIPHTVDGGIVFRRIDLDPPVDIPANALLIQEAFMCSNLVTGDIKVGTIEHVM**

20 4300STDY7045870 100.0% 99.7% **MVKQRTLNRVVKASGIGLHSGQKVMINFIPHTVDGGIVFRRIDLDPPVDIPANALLIQEAFMCSNLVTGDIKVGTIEHVM**

21 4300STDY7045866 100.0% 99.7% **MVKQRTLNRVVKASGIGLHSGQKVMINFIPHTVDGGIVFRRIDLDPPVDIPANALLIQEAFMCSNLVTGDIKVGTIEHVM**

22 4300STDY6542380 100.0% 99.7% **MVKQRTLNRVVKASGIGLHSGQKVMINFIPHTVDGGIVFRRIDLDPPVDIPANALLIQEAFMCSNLVTGDIKVGTIEHVM**

23 M10 62.0% 99.5% **MVKQRTLNRVVKASGIGLHSGQKVMINFIPHTVDGGIVFRRIDLDPPVDIPANALLIQEAFMCSNLVTGDIKVGTIEHVM**

cov pid  **81**  **. 1 . . . . : .** **160**

1 ATCC19606 100.0% 100.0% **SAIAGLGIDNLIVEVSASEVPIMDGSAGPFIYLLMQGGLREQDAPKKFIKILKPVEALIDDKKAIFSPHNGFQLNFTIDF**

2 M19 100.0% 99.7% **SAIAGLGIDNLIVEVSASEVPIMDGSAGPFIYLLMQGGLREQDAPKKFIKILKPVEALIDDKKAIFSPHNGFQLNFTIDF**

3 A5 100.0% 99.7% **SAIAGLGIDNLIVEVSASEVPIMDGSAGPFIYLLMQGGLREQDAPKKFIKILKPVEALIDDKKAIFSPHNGFQLNFTIDF**

4 Aci00709 100.0% 99.7% **SAIAGLGIDNLIVEVSASEVPIMDGSAGPFIYLLMQGGLREQDAPKKFIKILKPVEALIDDKKAIFSPHNGFQLNFTIDF**

5 Aci00711 100.0% 99.7% **SAIAGLGIDNLIVEVSASEVPIMDGSAGPFIYLLMQGGLREQDAPKKFIKILKPVEALIDDKKAIFSPHNGFQLNFTIDF**

6 KAB15 100.0% 99.7% **SAIAGLGIDNLIVEVSASEVPIMDGSAGPFIYLLMQGGLREQDAPKKFIKILKPVEALIDDKKAIFSPHNGFQLNFTIDF**

7 PT022 100.0% 99.7% **SAIAGLGIDNLIVEVSASEVPIMDGSAGPFIYLLMQGGLREQDAPKKFIKILKPVEALIDDKKAIFSPHNGFQLNFTIDF**

8 PSU091 100.0% 99.7% **SAIAGLGIDNLIVEVSASEVPIMDGSAGPFIYLLMQGGLREQDAPKKFIKILKPVEALIDDKKAIFSPHNGFQLNFTIDF**

9 151 100.0% 99.7% **SAIAGLGIDNLIVEVSASEVPIMDGSAGPFIYLLMQGGLREQDAPKKFIKILKPVEALIDDKKAIFSPHNGFQLNFTIDF**

10 GML-KP48-AB-TR 100.0% 99.7% **SAIAGLGIDNLIVEVSASEVPIMDGSAGPFIYLLMQGGLREQDAPKKFIKILKPVEALIDDKKAIFSPHNGFQLNFTIDF**

11 198 100.0% 99.7% **SAIAGLGIDNLIVEVSASEVPIMDGSAGPFIYLLMQGGLREQDAPKKFIKILKPVEALIDDKKAIFSPHNGFQLNFTIDF**

12 4300STDY7045706 100.0% 99.7% **SAIAGLGIDNLIVEVSASEVPIMDGSAGPFIYLLMQGGLREQDAPKKFIKILKPVEALIDDKKAIFSPHNGFQLNFTIDF**

13 4300STDY7045763 100.0% 99.7% **SAIAGLGIDNLIVEVSASEVPIMDGSAGPFIYLLMQGGLREQDAPKKFIKILKPVEALIDDKKAIFSPHNGFQLNFTIDF**

14 4300STDY7045798 100.0% 99.7% **SAIAGLGIDNLIVEVSASEVPIMDGSAGPFIYLLMQGGLREQDAPKKFIKILKPVEALIDDKKAIFSPHNGFQLNFTIDF**

15 4300STDY7045799 100.0% 99.7% **SAIAGLGIDNLIVEVSASEVPIMDGSAGPFIYLLMQGGLREQDAPKKFIKILKPVEALIDDKKAIFSPHNGFQLNFTIDF**

16 4300STDY7045808 100.0% 99.7% **SAIAGLGIDNLIVEVSASEVPIMDGSAGPFIYLLMQGGLREQDAPKKFIKILKPVEALIDDKKAIFSPHNGFQLNFTIDF**

17 4300STDY7045806 100.0% 99.7% **SAIAGLGIDNLIVEVSASEVPIMDGSAGPFIYLLMQGGLREQDAPKKFIKILKPVEALIDDKKAIFSPHNGFQLNFTIDF**

18 4300STDY7045811 100.0% 99.7% **SAIAGLGIDNLIVEVSASEVPIMDGSAGPFIYLLMQGGLREQDAPKKFIKILKPVEALIDDKKAIFSPHNGFQLNFTIDF**

19 4300STDY7045829 100.0% 99.7% **SAIAGLGIDNLIVEVSASEVPIMDGSAGPFIYLLMQGGLREQDAPKKFIKILKPVEALIDDKKAIFSPHNGFQLNFTIDF**

20 4300STDY7045870 100.0% 99.7% **SAIAGLGIDNLIVEVSASEVPIMDGSAGPFIYLLMQGGLREQDAPKKFIKILKPVEALIDDKKAIFSPHNGFQLNFTIDF**

21 4300STDY7045866 100.0% 99.7% **SAIAGLGIDNLIVEVSASEVPIMDGSAGPFIYLLMQGGLREQDAPKKFIKILKPVEALIDDKKAIFSPHNGFQLNFTIDF**

22 4300STDY6542380 100.0% 99.7% **SAIAGLGIDNLIVEVSASEVPIMDGSAGPFIYLLMQGGLREQDAPKKFIKILKPVEALIDDKKAIFSPHNGFQLNFTIDF**

23 M10 62.0% 99.5% **SAIAGLGIDNLIVEVSASEVPIMDGSAGPFIYLLMQGGLREQDAPKKFITILKPVEALIDDKKAIFSPHNGFQLNFTIDF**

cov pid **161**  **. . . 2 . . . .** **240**

1 ATCC19606 100.0% 100.0% **DHPAFAKEYQSATIDFSTETFVYEVSEARTFGFMKDLDYLKANNLALGASLDNAIGVDDTGVVNEEGLRFADEFVRHKIL**

2 M19 100.0% 99.7% **DHPAFAKEYQSATIDFSTETFVYEVSEARTFGFMKDLDYLKANNLALGASLDNAIGVDDTGVVNEEGLRFADEFVRHKIL**

3 A5 100.0% 99.7% **DHPAFAKEYQSATIDFSTETFVYEVSEARTFGFMKDLDYLKANNLALGASLDNAIGVDDTGVVNEEGLRFADEFVRHKIL**

4 Aci00709 100.0% 99.7% **DHPAFAKEYQSATIDFSTETFVYEVSEARTFGFMKDLDYLKANNLALGASLDNAIGVDDTGVVNEEGLRFADEFVRHKIL**

5 Aci00711 100.0% 99.7% **DHPAFAKEYQSATIDFSTETFVYEVSEARTFGFMKDLDYLKANNLALGASLDNAIGVDDTGVVNEEGLRFADEFVRHKIL**

6 KAB15 100.0% 99.7% **DHPAFAKEYQSATIDFSTETFVYEVSEARTFGFMKDLDYLKANNLALGASLDNAIGVDDTGVVNEEGLRFADEFVRHKIL**

7 PT022 100.0% 99.7% **DHPAFAKEYQSATIDFSTETFVYEVSEARTFGFMKDLDYLKANNLALGASLDNAIGVDDTGVVNEEGLRFADEFVRHKIL**

8 PSU091 100.0% 99.7% **DHPAFAKEYQSATIDFSTETFVYEVSEARTFGFMKDLDYLKANNLALGASLDNAIGVDDTGVVNEEGLRFADEFVRHKIL**

9 151 100.0% 99.7% **DHPAFAKEYQSATIDFSTETFVYEVSEARTFGFMKDLDYLKANNLALGASLDNAIGVDDTGVVNEEGLRFADEFVRHKIL**

10 GML-KP48-AB-TR 100.0% 99.7% **DHPAFAKEYQSATIDFSTETFVYEVSEARTFGFMKDLDYLKANNLALGASLDNAIGVDDTGVVNEEGLRFADEFVRHKIL**

11 198 100.0% 99.7% **DHPAFAKEYQSATIDFSTETFVYEVSEARTFGFMKDLDYLKANNLALGASLDNAIGVDDTGVVNEEGLRFADEFVRHKIL**

12 4300STDY7045706 100.0% 99.7% **DHPAFAKEYQSATIDFSTETFVYEVSEARTFGFMKDLDYLKANNLALGASLDNAIGVDDTGVVNEEGLRFADEFVRHKIL**

13 4300STDY7045763 100.0% 99.7% **DHPAFAKEYQSATIDFSTETFVYEVSEARTFGFMKDLDYLKANNLALGASLDNAIGVDDTGVVNEEGLRFADEFVRHKIL**

14 4300STDY7045798 100.0% 99.7% **DHPAFAKEYQSATIDFSTETFVYEVSEARTFGFMKDLDYLKANNLALGASLDNAIGVDDTGVVNEEGLRFADEFVRHKIL**

15 4300STDY7045799 100.0% 99.7% **DHPAFAKEYQSATIDFSTETFVYEVSEARTFGFMKDLDYLKANNLALGASLDNAIGVDDTGVVNEEGLRFADEFVRHKIL**

16 4300STDY7045808 100.0% 99.7% **DHPAFAKEYQSATIDFSTETFVYEVSEARTFGFMKDLDYLKANNLALGASLDNAIGVDDTGVVNEEGLRFADEFVRHKIL**

17 4300STDY7045806 100.0% 99.7% **DHPAFAKEYQSATIDFSTETFVYEVSEARTFGFMKDLDYLKANNLALGASLDNAIGVDDTGVVNEEGLRFADEFVRHKIL**

18 4300STDY7045811 100.0% 99.7% **DHPAFAKEYQSATIDFSTETFVYEVSEARTFGFMKDLDYLKANNLALGASLDNAIGVDDTGVVNEEGLRFADEFVRHKIL**

19 4300STDY7045829 100.0% 99.7% **DHPAFAKEYQSATIDFSTETFVYEVSEARTFGFMKDLDYLKANNLALGASLDNAIGVDDTGVVNEEGLRFADEFVRHKIL**

20 4300STDY7045870 100.0% 99.7% **DHPAFAKEYQSATIDFSTETFVYEVSEARTFGFMKDLDYLKANNLALGASLDNAIGVDDTGVVNEEGLRFADEFVRHKIL**

21 4300STDY7045866 100.0% 99.7% **DHPAFAKEYQSATIDFSTETFVYEVSEARTFGFMKDLDYLKANNLALGASLDNAIGVDDTGVVNEEGLRFADEFVRHKIL**

22 4300STDY6542380 100.0% 99.7% **DHPAFAKEYQSATIDFSTETFVYEVSEARTFGFMKDLDYLKANNLALGASLDNAIGVDDTGVVNEEGLRFADEFVRHKIL**

23 M10 62.0% 99.5% **DHPAFAKEYQSATIDFSTETFVYEVS------------------------------------------------------**

cov pid **241**  **: . . . . ]** **300**

1 ATCC19606 100.0% 100.0% **DAVGDLYLLGHQIIAKFDGYKSGHALNNQLLRNVQSDPSNYEIVTFNDEKDCPIPYVSVT**

2 M19 100.0% 99.7% **DAVGDLYLLGHQIIAKFDGYKSGHALNNQLLRNVQSDPSNYEIVTFDDEKDCPIPYVSVT**

3 A5 100.0% 99.7% **DAVGDLYLLGHQIIAKFDGYKSGHALNNQLLRNVQSDPSNYEIVTFDDEKDCPIPYVSVT**

4 Aci00709 100.0% 99.7% **DAVGDLYLLGHQIIAKFDGYKSGHALNNQLLRNVQSDPSNYEIVTFDDEKDCPIPYVSVT**

5 Aci00711 100.0% 99.7% **DAVGDLYLLGHQIIAKFDGYKSGHALNNQLLRNVQSDPSNYEIVTFDDEKDCPIPYVSVT**

6 KAB15 100.0% 99.7% **DAVGDLYLLGHQIIAKFDGYKSGHALNNQLLRNVQSDPSNYEIVTFDDEKDCPIPYVSVT**

7 PT022 100.0% 99.7% **DAVGDLYLLGHQIIAKFDGYKSGHALNNQLLRNVQSDPSNYEIVTFDDEKDCPIPYVSVT**

8 PSU091 100.0% 99.7% **DAVGDLYLLGHQIIAKFDGYKSGHALNNQLLRNVQSDPSNYEIVTFDDEKDCPIPYVSVT**

9 151 100.0% 99.7% **DAVGDLYLLGHQIIAKFDGYKSGHALNNQLLRNVQSDPSNYEIVTFDDEKDCPIPYVSVT**

10 GML-KP48-AB-TR 100.0% 99.7% **DAVGDLYLLGHQIIAKFDGYKSGHALNNQLLRNVQSDPSNYEIVTFDDEKDCPIPYVSVT**

11 198 100.0% 99.7% **DAVGDLYLLGHQIIAKFDGYKSGHALNNQLLRNVQSDPSNYEIVTFDDEKDCPIPYVSVT**

12 4300STDY7045706 100.0% 99.7% **DAVGDLYLLGHQIIAKFDGYKSGHALNNQLLRNVQSDPSNYEIVTFDDEKDCPIPYVSVT**

13 4300STDY7045763 100.0% 99.7% **DAVGDLYLLGHQIIAKFDGYKSGHALNNQLLRNVQSDPSNYEIVTFDDEKDCPIPYVSVT**

14 4300STDY7045798 100.0% 99.7% **DAVGDLYLLGHQIIAKFDGYKSGHALNNQLLRNVQSDPSNYEIVTFDDEKDCPIPYVSVT**

15 4300STDY7045799 100.0% 99.7% **DAVGDLYLLGHQIIAKFDGYKSGHALNNQLLRNVQSDPSNYEIVTFDDEKDCPIPYVSVT**

16 4300STDY7045808 100.0% 99.7% **DAVGDLYLLGHQIIAKFDGYKSGHALNNQLLRNVQSDPSNYEIVTFDDEKDCPIPYVSVT**

17 4300STDY7045806 100.0% 99.7% **DAVGDLYLLGHQIIAKFDGYKSGHALNNQLLRNVQSDPSNYEIVTFDDEKDCPIPYVSVT**

18 4300STDY7045811 100.0% 99.7% **DAVGDLYLLGHQIIAKFDGYKSGHALNNQLLRNVQSDPSNYEIVTFDDEKDCPIPYVSVT**

19 4300STDY7045829 100.0% 99.7% **DAVGDLYLLGHQIIAKFDGYKSGHALNNQLLRNVQSDPSNYEIVTFDDEKDCPIPYVSVT**

20 4300STDY7045870 100.0% 99.7% **DAVGDLYLLGHQIIAKFDGYKSGHALNNQLLRNVQSDPSNYEIVTFDDEKDCPIPYVSVT**

21 4300STDY7045866 100.0% 99.7% **DAVGDLYLLGHQIIAKFDGYKSGHALNNQLLRNVQSDPSNYEIVTFDDEKDCPIPYVSVT**

22 4300STDY6542380 100.0% 99.7% **DAVGDLYLLGHQIIAKFDGYKSGHALNNQLLRNVQSDPSNYEIVTFDDEKDCPIPYVSVT**

23 M10 62.0% 99.5% **------------------------------------------------------------**

**Supplementary Figure 15: Multiple sequence alignment (MSA) of the predicted amino acid sequence of LpxC carried by ST164^Pas^ and the phylogenetically related isolate M10 and close genomes retrieved from the BV-BRC database compared to the respective gene in *A. baumannii* ATCC 19606.** MSA was created by the A multiple alignment viewer MView hosted by the EMBL-EBI; cov, coverage; pid, percent identity.

cov pid  **1** **[ . . . . : . . .** **80**

1 ATCC19606 100.0% 100.0% **MKVQQYRLDELAHLVKGELIGEGSLQFSNLASLENAEVNHLTFVNGEKHLDQAKVSRAGAYIVTAALKEHLPEKDNFIIV**

2 M15 100.0% 100.0% **MKVQQYRLDELAHLVKGELIGEGSLQFSNLASLENAEVNHLTFVNGEKHLDQAKVSRAGAYIVTAALKEHLPEKDNFIIV**

3 M12 91.0% 99.7% **---------------------------------ENAEVNHLTFVNGEKHLDQAKVSRAGAYIVTAALKEHLPEKDNFIIV**

4 M06 25.6% 100.0% **MKVQQYRLDELAHLVKGELIGEGSLQFSNLASLENAEVNHLTFVNGEKHLDQAKVSRAGAYIVTAALKEHLPEKDNFIIV**

5 M06b 46.9% 100.0% **--------------------------------------------------------------------------------**

6 M09 98.9% 99.0% **---------ELVHLVKGGLIGEGSLQFSNLASLENAEVNHLTFVNGEKHLDQAKVSRAGAYIVTAALKEHLPEKDNFIIV**

7 MRSN7224 100.0% 100.0% **MKVQQYRLDELAHLVKGELIGEGSLQFSNLASLENAEVNHLTFVNGEKHLDQAKVSRAGAYIVTAALKEHLPEKDNFIIV**

8 15A1042 100.0% 100.0% **MKVQQYRLDELAHLVKGELIGEGSLQFSNLASLENAEVNHLTFVNGEKHLDQAKVSRAGAYIVTAALKEHLPEKDNFIIV**

9 SRR3222490 100.0% 100.0% **MKVQQYRLDELAHLVKGELIGEGSLQFSNLASLENAEVNHLTFVNGEKHLDQAKVSRAGAYIVTAALKEHLPEKDNFIIV**

10 SRR3228565 100.0% 100.0% **MKVQQYRLDELAHLVKGELIGEGSLQFSNLASLENAEVNHLTFVNGEKHLDQAKVSRAGAYIVTAALKEHLPEKDNFIIV**

11 SRR3228488 100.0% 100.0% **MKVQQYRLDELAHLVKGELIGEGSLQFSNLASLENAEVNHLTFVNGEKHLDQAKVSRAGAYIVTAALKEHLPEKDNFIIV**

12 A18 100.0% 100.0% **MKVQQYRLDELAHLVKGELIGEGSLQFSNLASLENAEVNHLTFVNGEKHLDQAKVSRAGAYIVTAALKEHLPEKDNFIIV**

13 MRSN7133 100.0% 100.0% **MKVQQYRLDELAHLVKGELIGEGSLQFSNLASLENAEVNHLTFVNGEKHLDQAKVSRAGAYIVTAALKEHLPEKDNFIIV**

14 OIFC074 100.0% 100.0% **MKVQQYRLDELAHLVKGELIGEGSLQFSNLASLENAEVNHLTFVNGEKHLDQAKVSRAGAYIVTAALKEHLPEKDNFIIV**

15 SRR3227013 100.0% 100.0% **MKVQQYRLDELAHLVKGELIGEGSLQFSNLASLENAEVNHLTFVNGEKHLDQAKVSRAGAYIVTAALKEHLPEKDNFIIV**

16 MRSN7130 100.0% 100.0% **MKVQQYRLDELAHLVKGELIGEGSLQFSNLASLENAEVNHLTFVNGEKHLDQAKVSRAGAYIVTAALKEHLPEKDNFIIV**

17 MRSN7202 100.0% 100.0% **MKVQQYRLDELAHLVKGELIGEGSLQFSNLASLENAEVNHLTFVNGEKHLDQAKVSRAGAYIVTAALKEHLPEKDNFIIV**

cov pid  **81**  **. 1 . . . . : .** **160**

1 ATCC19606 100.0% 100.0% **DNPYLAFAILTHVFDKKISSTGIESTAQIHPSAVISETAYIGHYVVIGENCVVGDNTVIQSHTKLDDNVEVGKDCFIDSH**

2 M15 100.0% 100.0% **DNPYLAFAILTHVFDKKISSTGIESTAQIHPSAVISETAYIGHYVVIGENCVVGDNTVIQSHTKLDDNVEVGKDCFIDSH**

3 M12 91.0% 99.7% **DNPYLAFAILTHVFDKKISSTGIESTAQIHPSAVISETAYIGHYVVIGENCVVGDNTVIQSHTKLDDNVEVGKDCFIDSH**

4 M06 25.6% 100.0% **DNPYLAFAILT---------------------------------------------------------------------**

5 M06b 46.9% 100.0% **--------------------------------------------------------------------------------**

6 M09 98.9% 99.0% **DNPYLAFAILTHVFDKKISSTGIESTAQIHPSAVISETAYIGHYVVIGENCVVGDNTVIQSHTKLDDNVEVGKDCFIDSH**

7 MRSN7224 100.0% 100.0% **DNPYLAFAILTHVFDKKISSTGIESTAQIHPSAVISETAYIGHYVVIGENCVVGDNTVIQSHTKLDDNVEVGKDCFIDSH**

8 15A1042 100.0% 100.0% **DNPYLAFAILTHVFDKKISSTGIESTAQIHPSAVISETAYIGHYVVIGENCVVGDNTVIQSHTKLDDNVEVGKDCFIDSH**

9 SRR3222490 100.0% 100.0% **DNPYLAFAILTHVFDKKISSTGIESTAQIHPSAVISETAYIGHYVVIGENCVVGDNTVIQSHTKLDDNVEVGKDCFIDSH**

10 SRR3228565 100.0% 100.0% **DNPYLAFAILTHVFDKKISSTGIESTAQIHPSAVISETAYIGHYVVIGENCVVGDNTVIQSHTKLDDNVEVGKDCFIDSH**

11 SRR3228488 100.0% 100.0% **DNPYLAFAILTHVFDKKISSTGIESTAQIHPSAVISETAYIGHYVVIGENCVVGDNTVIQSHTKLDDNVEVGKDCFIDSH**

12 A18 100.0% 100.0% **DNPYLAFAILTHVFDKKISSTGIESTAQIHPSAVISETAYIGHYVVIGENCVVGDNTVIQSHTKLDDNVEVGKDCFIDSH**

13 MRSN7133 100.0% 100.0% **DNPYLAFAILTHVFDKKISSTGIESTAQIHPSAVISETAYIGHYVVIGENCVVGDNTVIQSHTKLDDNVEVGKDCFIDSH**

14 OIFC074 100.0% 100.0% **DNPYLAFAILTHVFDKKISSTGIESTAQIHPSAVISETAYIGHYVVIGENCVVGDNTVIQSHTKLDDNVEVGKDCFIDSH**

15 SRR3227013 100.0% 100.0% **DNPYLAFAILTHVFDKKISSTGIESTAQIHPSAVISETAYIGHYVVIGENCVVGDNTVIQSHTKLDDNVEVGKDCFIDSH**

16 MRSN7130 100.0% 100.0% **DNPYLAFAILTHVFDKKISSTGIESTAQIHPSAVISETAYIGHYVVIGENCVVGDNTVIQSHTKLDDNVEVGKDCFIDSH**

17 MRSN7202 100.0% 100.0% **DNPYLAFAILTHVFDKKISSTGIESTAQIHPSAVISETAYIGHYVVIGENCVVGDNTVIQSHTKLDDNVEVGKDCFIDSH**

cov pid **161**  **. . . 2 . . . .** **240**

1 ATCC19606 100.0% 100.0% **VTITGGSKLRDRVRIHSSTVIGGEGFGFAPYQGKWHRIAQLGSVLIGNDVRIGSNCSIDRGALDNTILEDGVIIDNLVQI**

2 M15 100.0% 100.0% **VTITGGSKLRDRVRIHSSTVIGGEGFGFAPYQGKWHRIAQLGSVLIGNDVRIGSNCSIDRGALDNTILEDGVIIDNLVQI**

3 M12 91.0% 99.7% **VTITGGSKLRDRVRIHSSTVIGGEGFGFAPYQGKWHRIAQLGSVLIGNDVRIGSNCSIDRGALDNTILEDGVIIDNLVQI**

4 M06 25.6% 100.0% **--------------------------------------------------------------------------------**

5 M06b 46.9% 100.0% **-----------------------------PYQGKWHRIAQLGSVLIGNDVRIGSNCSIDRGALDNTILEDGVIIDNLVQI**

6 M09 98.9% 99.0% **VTITGGSKLRDRVRIHSSTVIGGEGFGFAPYQGKWHRIAQLGSVLIGNDVRIGSNCSIDRGALDNTILEDGVIIDNLVQI**

7 MRSN7224 100.0% 100.0% **VTITGGSKLRDRVRIHSSTVIGGEGFGFAPYQGKWHRIAQLGSVLIGNDVRIGSNCSIDRGALDNTILEDGVIIDNLVQI**

8 15A1042 100.0% 100.0% **VTITGGSKLRDRVRIHSSTVIGGEGFGFAPYQGKWHRIAQLGSVLIGNDVRIGSNCSIDRGALDNTILEDGVIIDNLVQI**

9 SRR3222490 100.0% 100.0% **VTITGGSKLRDRVRIHSSTVIGGEGFGFAPYQGKWHRIAQLGSVLIGNDVRIGSNCSIDRGALDNTILEDGVIIDNLVQI**

10 SRR3228565 100.0% 100.0% **VTITGGSKLRDRVRIHSSTVIGGEGFGFAPYQGKWHRIAQLGSVLIGNDVRIGSNCSIDRGALDNTILEDGVIIDNLVQI**

11 SRR3228488 100.0% 100.0% **VTITGGSKLRDRVRIHSSTVIGGEGFGFAPYQGKWHRIAQLGSVLIGNDVRIGSNCSIDRGALDNTILEDGVIIDNLVQI**

12 A18 100.0% 100.0% **VTITGGSKLRDRVRIHSSTVIGGEGFGFAPYQGKWHRIAQLGSVLIGNDVRIGSNCSIDRGALDNTILEDGVIIDNLVQI**

13 MRSN7133 100.0% 100.0% **VTITGGSKLRDRVRIHSSTVIGGEGFGFAPYQGKWHRIAQLGSVLIGNDVRIGSNCSIDRGALDNTILEDGVIIDNLVQI**

14 OIFC074 100.0% 100.0% **VTITGGSKLRDRVRIHSSTVIGGEGFGFAPYQGKWHRIAQLGSVLIGNDVRIGSNCSIDRGALDNTILEDGVIIDNLVQI**

15 SRR3227013 100.0% 100.0% **VTITGGSKLRDRVRIHSSTVIGGEGFGFAPYQGKWHRIAQLGSVLIGNDVRIGSNCSIDRGALDNTILEDGVIIDNLVQI**

16 MRSN7130 100.0% 100.0% **VTITGGSKLRDRVRIHSSTVIGGEGFGFAPYQGKWHRIAQLGSVLIGNDVRIGSNCSIDRGALDNTILEDGVIIDNLVQI**

17 MRSN7202 100.0% 100.0% **VTITGGSKLRDRVRIHSSTVIGGEGFGFAPYQGKWHRIAQLGSVLIGNDVRIGSNCSIDRGALDNTILEDGVIIDNLVQI**

cov pid **241**  **: . . . . 3 . .** **320**

1 ATCC19606 100.0% 100.0% **AHNVHIGSNTAIAAKCGIAGSTKIGKNCILAGACGVAGHLSIADNVTLTGMSMVTKNISEAGTYSSGTGLFENNHWKKTI**

2 M15 100.0% 100.0% **AHNVHIGSNTAIAAKCGIAGSTKIGKNCILAGACGVAGHLSIADNVTLTGMSMVTKNISEAGTYSSGTGLFENNHWKKTI**

3 M12 91.0% 99.7% **AHNVHIGSNTAIAAKCGIAGSTKIGKNCILAGACGVAGHLSIADNVTLTGMSMVTKNISEAGTYSSGTGLFENNHWKKTI**

4 M06 25.6% 100.0% **--------------------------------------------------------------------------------**

5 M06b 46.9% 100.0% **AHNVHIGSNTAIAAKCGIAGSTKIGKNCILAGACGVAGHLSIADNVTLTGMSMVTKNISEAGTYSSGTGLFENNHWKKTI**

6 M09 98.9% 98.0% **AHNVHIGSNTAIAAKCGIAGSTKIGKNCILAGACGVAGHLSIADNVTLTGMSMVTKNISEAGTYSSGTGLFENNHWKKTI**

7 MRSN7224 100.0% 100.0% **AHNVHIGSNTAIAAKCGIAGSTKIGKNCILAGACGVAGHLSIADNVTLTGMSMVTKNISEAGTYSSGTGLFENNHWKKTI**

8 15A1042 100.0% 100.0% **AHNVHIGSNTAIAAKCGIAGSTKIGKNCILAGACGVAGHLSIADNVTLTGMSMVTKNISEAGTYSSGTGLFENNHWKKTI**

9 SRR3222490 100.0% 100.0% **AHNVHIGSNTAIAAKCGIAGSTKIGKNCILAGACGVAGHLSIADNVTLTGMSMVTKNISEAGTYSSGTGLFENNHWKKTI**

10 SRR3228565 100.0% 100.0% **AHNVHIGSNTAIAAKCGIAGSTKIGKNCILAGACGVAGHLSIADNVTLTGMSMVTKNISEAGTYSSGTGLFENNHWKKTI**

11 SRR3228488 100.0% 100.0% **AHNVHIGSNTAIAAKCGIAGSTKIGKNCILAGACGVAGHLSIADNVTLTGMSMVTKNISEAGTYSSGTGLFENNHWKKTI**

12 A18 100.0% 100.0% **AHNVHIGSNTAIAAKCGIAGSTKIGKNCILAGACGVAGHLSIADNVTLTGMSMVTKNISEAGTYSSGTGLFENNHWKKTI**

13 MRSN7133 100.0% 100.0% **AHNVHIGSNTAIAAKCGIAGSTKIGKNCILAGACGVAGHLSIADNVTLTGMSMVTKNISEAGTYSSGTGLFENNHWKKTI**

14 OIFC074 100.0% 100.0% **AHNVHIGSNTAIAAKCGIAGSTKIGKNCILAGACGVAGHLSIADNVTLTGMSMVTKNISEAGTYSSGTGLFENNHWKKTI**

15 SRR3227013 100.0% 100.0% **AHNVHIGSNTAIAAKCGIAGSTKIGKNCILAGACGVAGHLSIADNVTLTGMSMVTKNISEAGTYSSGTGLFENNHWKKTI**

16 MRSN7130 100.0% 100.0% **AHNVHIGSNTAIAAKCGIAGSTKIGKNCILAGACGVAGHLSIADNVTLTGMSMVTKNISEAGTYSSGTGLFENNHWKKTI**

17 MRSN7202 100.0% 100.0% **AHNVHIGSNTAIAAKCGIAGSTKIGKNCILAGACGVAGHLSIADNVTLTGMSMVTKNISEAGTYSSGTGLFENNHWKKTI**

cov pid **321**  **. . : ]** **356**

1 ATCC19606 100.0% 100.0% **VRLRQLADVPLTQITKRLDHIQAQIESLESTFNLRK**

2 M15 100.0% 100.0% **VRLRQLADVPLTQITKRLDHIQAQIESLESTFNLRK**

3 M12 91.0% 99.7% **VRLRQLADVPLTQITKRLDHIQAQIESLESTFNLRK**

4 M06 25.6% 100.0% **------------------------------------**

5 M06b 46.9% 100.0% **VRLRQLADVPLTQITKRLDHIQAQIESLESTFNLRK**

6 M09 98.9% 99.0% **VRLRQLADVPLTQITKRLDHIQAQIESLESTFNLRK**

7 MRSN7224 100.0% 100.0% **VRLRQLADVPLTQITKRLDHIQAQIESLESTFNLRK**

8 15A1042 100.0% 100.0% **VRLRQLADVPLTQITKRLDHIQAQIESLESTFNLRK**

9 SRR3222490 100.0% 100.0% **VRLRQLADVPLTQITKRLDHIQAQIESLESTFNLRK**

10 SRR3228565 100.0% 100.0% **VRLRQLADVPLTQITKRLDHIQAQIESLESTFNLRK**

11 SRR3228488 100.0% 100.0% **VRLRQLADVPLTQITKRLDHIQAQIESLESTFNLRK**

12 A18 100.0% 100.0% **VRLRQLADVPLTQITKRLDHIQAQIESLESTFNLRK**

13 MRSN7133 100.0% 100.0% **VRLRQLADVPLTQITKRLDHIQAQIESLESTFNLRK**

14 OIFC074 100.0% 100.0% **VRLRQLADVPLTQITKRLDHIQAQIESLESTFNLRK**

15 SRR3227013 100.0% 100.0% **VRLRQLADVPLTQITKRLDHIQAQIESLESTFNLRK**

16 MRSN7130 100.0% 100.0% **VRLRQLADVPLTQITKRLDHIQAQIESLESTFNLRK**

17 MRSN7202 100.0% 100.0% **VRLRQLADVPLTQITKRLDHIQAQIESLESTFNLRK**

**Supplementary Figure 16: Multiple sequence alignment (MSA) of the predicted amino acid sequence of LpxD carried by ST19^Pas^ (GC1) and the phylogenetically related isolates M06 and M09 isolates and close genomes retrieved from the BV-BRC database compared to the respective gene in *A. baumannii* ATCC 19606.** MSA was created by the A multiple alignment viewer MView hosted by the EMBL-EBI; cov, coverage; pid, percent identity.

cov pid  **1** **[ . . . . : . . .** **80**

1 ATCC19606 100.0% 100.0% **MKVQQYRLDELAHLVKGELIGEGSLQFSNLASLENAEVNHLTFVNGEKHLDQAKVSRAGAYIVTAALKEHLPEKDNFIIV**

2 M01 100.0% 99.7% **MKVQQYRLDELAHLVKGELIGEGSLQFSNLASLENAEVNHLTFVNGEKHLDQAKVSRAGAYIVTAALKEHLPEKDNFIIV**

3 M04 100.0% 99.7% **MKVQQYRLDELAHLVKGELIGEGSLQFSNLASLENAEVNHLTFVNGEKHLDQAKVSRAGAYIVTAALKEHLPEKDNFIIV**

4 M05 100.0% 99.7% **MKVQQYRLDELAHLVKGELIGEGSLQFSNLASLENAEVNHLTFVNGEKHLDQAKVSRAGAYIVTAALKEHLPEKDNFIIV**

5 M13 72.8% 99.6% **--------------------------------------------------------------------------------**

6 M16 100.0% 99.7% **MKVQQYRLDELAHLVKGELIGEGSLQFSNLASLENAEVNHLTFVNGEKHLDQAKVSRAGAYIVTAALKEHLPEKDNFIIV**

7 M17 100.0% 99.7% **MKVQQYRLDELAHLVKGELIGEGSLQFSNLASLENAEVNHLTFVNGEKHLDQAKVSRAGAYIVTAALKEHLPEKDNFIIV**

8 M20 100.0% 99.7% **MKVQQYRLDELAHLVKGELIGEGSLQFSNLASLENAEVNHLTFVNGEKHLDQAKVSRAGAYIVTAALKEHLPEKDNFIIV**

9 MS14413 100.0% 99.7% **MKVQQYRLDELAHLVKGELIGEGSLQFSNLASLENAEVNHLTFVNGEKHLDQAKVSRAGAYIVTAALKEHLPEKDNFIIV**

10 SQ093 100.0% 99.7% **MKVQQYRLDELAHLVKGELIGEGSLQFSNLASLENAEVNHLTFVNGEKHLDQAKVSRAGAYIVTAALKEHLPEKDNFIIV**

11 KAB3 100.0% 99.7% **MKVQQYRLDELAHLVKGELIGEGSLQFSNLASLENAEVNHLTFVNGEKHLDQAKVSRAGAYIVTAALKEHLPEKDNFIIV**

12 AC-40 100.0% 99.7% **MKVQQYRLDELAHLVKGELIGEGSLQFSNLASLENAEVNHLTFVNGEKHLDQAKVSRAGAYIVTAALKEHLPEKDNFIIV**

13 AC-45 100.0% 99.7% **MKVQQYRLDELAHLVKGELIGEGSLQFSNLASLENAEVNHLTFVNGEKHLDQAKVSRAGAYIVTAALKEHLPEKDNFIIV**

14 AC-23 100.0% 99.7% **MKVQQYRLDELAHLVKGELIGEGSLQFSNLASLENAEVNHLTFVNGEKHLDQAKVSRAGAYIVTAALKEHLPEKDNFIIV**

15 A21 100.0% 99.7% **MKVQQYRLDELAHLVKGELIGEGSLQFSNLASLENAEVNHLTFVNGEKHLDQAKVSRAGAYIVTAALKEHLPEKDNFIIV**

16 SK044 100.0% 99.7% **MKVQQYRLDELAHLVKGELIGEGSLQFSNLASLENAEVNHLTFVNGEKHLDQAKVSRAGAYIVTAALKEHLPEKDNFIIV**

17 SK011 100.0% 99.7% **MKVQQYRLDELAHLVKGELIGEGSLQFSNLASLENAEVNHLTFVNGEKHLDQAKVSRAGAYIVTAALKEHLPEKDNFIIV**

18 SK002 100.0% 99.7% **MKVQQYRLDELAHLVKGELIGEGSLQFSNLASLENAEVNHLTFVNGEKHLDQAKVSRAGAYIVTAALKEHLPEKDNFIIV**

19 PSU120 100.0% 99.7% **MKVQQYRLDELAHLVKGELIGEGSLQFSNLASLENAEVNHLTFVNGEKHLDQAKVSRAGAYIVTAALKEHLPEKDNFIIV**

20 KUSSH35 100.0% 99.7% **MKVQQYRLDELAHLVKGELIGEGSLQFSNLASLENAEVNHLTFVNGEKHLDQAKVSRAGAYIVTAALKEHLPEKDNFIIV**

21 KUFAR56 100.0% 99.4% **MKVQQYRLDELAHLVKGELIGEGSLQFSNLASLENAEVNHLTFVNGEKHLDQAKVSRAGAYIVTAALKEHLPEKDNFIIV**

22 KUSSH36 100.0% 99.7% **MKVQQYRLDELAHLVKGELIGEGSLQFSNLASLENAEVNHLTFVNGEKHLDQAKVSRAGAYIVTAALKEHLPEKDNFIIV**

23 130 100.0% 99.7% **MKVQQYRLDELAHLVKGELIGEGSLQFSNLASLENAEVNHLTFVNGEKHLDQAKVSRAGAYIVTAALKEHLPEKDNFIIV**

24 Ab34 100.0% 99.7% **MKVQQYRLDELAHLVKGELIGEGSLQFSNLASLENAEVNHLTFVNGEKHLDQAKVSRAGAYIVTAALKEHLPEKDNFIIV**

25 Ab36 100.0% 99.7% **MKVQQYRLDELAHLVKGELIGEGSLQFSNLASLENAEVNHLTFVNGEKHLDQAKVSRAGAYIVTAALKEHLPEKDNFIIV**

26 Ab40 100.0% 99.7% **MKVQQYRLDELAHLVKGELIGEGSLQFSNLASLENAEVNHLTFVNGEKHLDQAKVSRAGAYIVTAALKEHLPEKDNFIIV**

27 Ab15 100.0% 99.7% **MKVQQYRLDELAHLVKGELIGEGSLQFSNLASLENAEVNHLTFVNGEKHLDQAKVSRAGAYIVTAALKEHLPEKDNFIIV**

28 Ab65 100.0% 99.7% **MKVQQYRLDELAHLVKGELIGEGSLQFSNLASLENAEVNHLTFVNGEKHLDQAKVSRAGAYIVTAALKEHLPEKDNFIIV**

29 AC-14 100.0% 99.7% **MKVQQYRLDELAHLVKGELIGEGSLQFSNLASLENAEVNHLTFVNGEKHLDQAKVSRAGAYIVTAALKEHLPEKDNFIIV**

30 SUH-26-2 100.0% 99.7% **MKVQQYRLDELAHLVKGELIGEGSLQFSNLASLENAEVNHLTFVNGEKHLDQAKVSRAGAYIVTAALKEHLPEKDNFIIV**

31 SUH-11-2 100.0% 99.7% **MKVQQYRLDELAHLVKGELIGEGSLQFSNLASLENAEVNHLTFVNGEKHLDQAKVSRAGAYIVTAALKEHLPEKDNFIIV**

32 SUH-11-1 100.0% 99.7% **MKVQQYRLDELAHLVKGELIGEGSLQFSNLASLENAEVNHLTFVNGEKHLDQAKVSRAGAYIVTAALKEHLPEKDNFIIV**

33 KUSSH15 100.0% 99.7% **MKVQQYRLDELAHLVKGELIGEGSLQFSNLASLENAEVNHLTFVNGEKHLDQAKVSRAGAYIVTAALKEHLPEKDNFIIV**

34 KUSSH37 100.0% 99.7% **MKVQQYRLDELAHLVKGELIGEGSLQFSNLASLENAEVNHLTFVNGEKHLDQAKVSRAGAYIVTAALKEHLPEKDNFIIV**

35 Ab35 100.0% 99.7% **MKVQQYRLDELAHLVKGELIGEGSLQFSNLASLENAEVNHLTFVNGEKHLDQAKVSRAGAYIVTAALKEHLPEKDNFIIV**

36 Ab38 100.0% 99.7% **MKVQQYRLDELAHLVKGELIGEGSLQFSNLASLENAEVNHLTFVNGEKHLDQAKVSRAGAYIVTAALKEHLPEKDNFIIV**

37 Ab41 100.0% 99.7% **MKVQQYRLDELAHLVKGELIGEGSLQFSNLASLENAEVNHLTFVNGEKHLDQAKVSRAGAYIVTAALKEHLPEKDNFIIV**

38 Ab64 100.0% 99.7% **MKVQQYRLDELAHLVKGELIGEGSLQFSNLASLENAEVNHLTFVNGEKHLDQAKVSRAGAYIVTAALKEHLPEKDNFIIV**

39 AB22 100.0% 99.7% **MKVQQYRLDELAHLVKGELIGEGSLQFSNLASLENAEVNHLTFVNGEKHLDQAKVSRAGAYIVTAALKEHLPEKDNFIIV**

cov pid  **81**  **. 1 . . . . : .** **160**

1 ATCC19606 100.0% 100.0% **DNPYLAFAILTHVFDKKISSTGIESTAQIHPSAVISETAYIGHYVVIGENCVVGDNTVIQSHTKLDDNVEVGKDCFIDSH**

2 M01 100.0% 99.7% **DNPYLAFAILTHVFDKKISSTGIESTAQIHPSAVISKTAYIGHYVVIGENCVVGDNTVIQSHTKLDDNVEVGKDCFIDSH**

3 M04 100.0% 99.7% **DNPYLAFAILTHVFDKKISSTGIESTAQIHPSAVISKTAYIGHYVVIGENCVVGDNTVIQSHTKLDDNVEVGKDCFIDSH**

4 M05 100.0% 99.7% **DNPYLAFAILTHVFDKKISSTGIESTAQIHPSAVISKTAYIGHYVVIGENCVVGDNTVIQSHTKLDDNVEVGKDCFIDSH**

5 M13 72.8% 99.6% **-----------------ISSTGIESTAQIHPSAVISKTAYIGHYVVIGENCVVGDNTVIQSHTKLDDNVEVGKDCFIDSH**

6 M16 100.0% 99.7% **DNPYLAFAILTHVFDKKISSTGIESTAQIHPSAVISKTAYIGHYVVIGENCVVGDNTVIQSHTKLDDNVEVGKDCFIDSH**

7 M17 100.0% 99.7% **DNPYLAFAILTHVFDKKISSTGIESTAQIHPSAVISKTAYIGHYVVIGENCVVGDNTVIQSHTKLDDNVEVGKDCFIDSH**

8 M20 100.0% 99.7% **DNPYLAFAILTHVFDKKISSTGIESTAQIHPSAVISKTAYIGHYVVIGENCVVGDNTVIQSHTKLDDNVEVGKDCFIDSH**

9 MS14413 100.0% 99.7% **DNPYLAFAILTHVFDKKISSTGIESTAQIHPSAVISKTAYIGHYVVIGENCVVGDNTVIQSHTKLDDNVEVGKDCFIDSH**

10 SQ093 100.0% 99.7% **DNPYLAFAILTHVFDKKISSTGIESTAQIHPSAVISKTAYIGHYVVIGENCVVGDNTVIQSHTKLDDNVEVGKDCFIDSH**

11 KAB3 100.0% 99.7% **DNPYLAFAILTHVFDKKISSTGIESTAQIHPSAVISKTAYIGHYVVIGENCVVGDNTVIQSHTKLDDNVEVGKDCFIDSH**

12 AC-40 100.0% 99.7% **DNPYLAFAILTHVFDKKISSTGIESTAQIHPSAVISKTAYIGHYVVIGENCVVGDNTVIQSHTKLDDNVEVGKDCFIDSH**

13 AC-45 100.0% 99.7% **DNPYLAFAILTHVFDKKISSTGIESTAQIHPSAVISKTAYIGHYVVIGENCVVGDNTVIQSHTKLDDNVEVGKDCFIDSH**

14 AC-23 100.0% 99.7% **DNPYLAFAILTHVFDKKISSTGIESTAQIHPSAVISKTAYIGHYVVIGENCVVGDNTVIQSHTKLDDNVEVGKDCFIDSH**

15 A21 100.0% 99.7% **DNPYLAFAILTHVFDKKISSTGIESTAQIHPSAVISKTAYIGHYVVIGENCVVGDNTVIQSHTKLDDNVEVGKDCFIDSH**

16 SK044 100.0% 99.7% **DNPYLAFAILTHVFDKKISSTGIESTAQIHPSAVISKTAYIGHYVVIGENCVVGDNTVIQSHTKLDDNVEVGKDCFIDSH**

17 SK011 100.0% 99.7% **DNPYLAFAILTHVFDKKISSTGIESTAQIHPSAVISKTAYIGHYVVIGENCVVGDNTVIQSHTKLDDNVEVGKDCFIDSH**

18 SK002 100.0% 99.7% **DNPYLAFAILTHVFDKKISSTGIESTAQIHPSAVISKTAYIGHYVVIGENCVVGDNTVIQSHTKLDDNVEVGKDCFIDSH**

19 PSU120 100.0% 99.7% **DNPYLAFAILTHVFDKKISSTGIESTAQIHPSAVISKTAYIGHYVVIGENCVVGDNTVIQSHTKLDDNVEVGKDCFIDSH**

20 KUSSH35 100.0% 99.7% **DNPYLAFAILTHVFDKKISSTGIESTAQIHPSAVISKTAYIGHYVVIGENCVVGDNTVIQSHTKLDDNVEVGKDCFIDSH**

21 KUFAR56 100.0% 99.4% **DNPYLAFAILTHVFDKKISSTGIESTAQIHPSAVISKTAYIGHYVVIGENCVVGDNTVIQSHTKLDDNVEVGKDCFIDSH**

22 KUSSH36 100.0% 99.7% **DNPYLAFAILTHVFDKKISSTGIESTAQIHPSAVISKTAYIGHYVVIGENCVVGDNTVIQSHTKLDDNVEVGKDCFIDSH**

23 130 100.0% 99.7% **DNPYLAFAILTHVFDKKISSTGIESTAQIHPSAVISKTAYIGHYVVIGENCVVGDNTVIQSHTKLDDNVEVGKDCFIDSH**

24 Ab34 100.0% 99.7% **DNPYLAFAILTHVFDKKISSTGIESTAQIHPSAVISKTAYIGHYVVIGENCVVGDNTVIQSHTKLDDNVEVGKDCFIDSH**

25 Ab36 100.0% 99.7% **DNPYLAFAILTHVFDKKISSTGIESTAQIHPSAVISKTAYIGHYVVIGENCVVGDNTVIQSHTKLDDNVEVGKDCFIDSH**

26 Ab40 100.0% 99.7% **DNPYLAFAILTHVFDKKISSTGIESTAQIHPSAVISKTAYIGHYVVIGENCVVGDNTVIQSHTKLDDNVEVGKDCFIDSH**

27 Ab15 100.0% 99.7% **DNPYLAFAILTHVFDKKISSTGIESTAQIHPSAVISKTAYIGHYVVIGENCVVGDNTVIQSHTKLDDNVEVGKDCFIDSH**

28 Ab65 100.0% 99.7% **DNPYLAFAILTHVFDKKISSTGIESTAQIHPSAVISKTAYIGHYVVIGENCVVGDNTVIQSHTKLDDNVEVGKDCFIDSH**

29 AC-14 100.0% 99.7% **DNPYLAFAILTHVFDKKISSTGIESTAQIHPSAVISKTAYIGHYVVIGENCVVGDNTVIQSHTKLDDNVEVGKDCFIDSH**

30 SUH-26-2 100.0% 99.7% **DNPYLAFAILTHVFDKKISSTGIESTAQIHPSAVISKTAYIGHYVVIGENCVVGDNTVIQSHTKLDDNVEVGKDCFIDSH**

31 SUH-11-2 100.0% 99.7% **DNPYLAFAILTHVFDKKISSTGIESTAQIHPSAVISKTAYIGHYVVIGENCVVGDNTVIQSHTKLDDNVEVGKDCFIDSH**

32 SUH-11-1 100.0% 99.7% **DNPYLAFAILTHVFDKKISSTGIESTAQIHPSAVISKTAYIGHYVVIGENCVVGDNTVIQSHTKLDDNVEVGKDCFIDSH**

33 KUSSH15 100.0% 99.7% **DNPYLAFAILTHVFDKKISSTGIESTAQIHPSAVISKTAYIGHYVVIGENCVVGDNTVIQSHTKLDDNVEVGKDCFIDSH**

34 KUSSH37 100.0% 99.7% **DNPYLAFAILTHVFDKKISSTGIESTAQIHPSAVISKTAYIGHYVVIGENCVVGDNTVIQSHTKLDDNVEVGKDCFIDSH**

35 Ab35 100.0% 99.7% **DNPYLAFAILTHVFDKKISSTGIESTAQIHPSAVISKTAYIGHYVVIGENCVVGDNTVIQSHTKLDDNVEVGKDCFIDSH**

36 Ab38 100.0% 99.7% **DNPYLAFAILTHVFDKKISSTGIESTAQIHPSAVISKTAYIGHYVVIGENCVVGDNTVIQSHTKLDDNVEVGKDCFIDSH**

37 Ab41 100.0% 99.7% **DNPYLAFAILTHVFDKKISSTGIESTAQIHPSAVISKTAYIGHYVVIGENCVVGDNTVIQSHTKLDDNVEVGKDCFIDSH**

38 Ab64 100.0% 99.7% **DNPYLAFAILTHVFDKKISSTGIESTAQIHPSAVISKTAYIGHYVVIGENCVVGDNTVIQSHTKLDDNVEVGKDCFIDSH**

39 AB22 100.0% 99.7% **DNPYLAFAILTHVFDKKISSTGIESTAQIHPSAVISKTAYIGHYVVIGENCVVGDNTVIQSHTKLDDNVEVGKDCFIDSH**

cov pid **161**  **. . . 2 . . . .** **240**

1 ATCC19606 100.0% 100.0% **VTITGGSKLRDRVRIHSSTVIGGEGFGFAPYQGKWHRIAQLGSVLIGNDVRIGSNCSIDRGALDNTILEDGVIIDNLVQI**

2 M01 100.0% 99.7% **VTITGGSKLRDRVRIHSSTVIGGEGFGFAPYQGKWHRIAQLGSVLIGNDVRIGSNCSIDRGALDNTILEDGVIIDNLVQI**

3 M04 100.0% 99.7% **VTITGGSKLRDRVRIHSSTVIGGEGFGFAPYQGKWHRIAQLGSVLIGNDVRIGSNCSIDRGALDNTILEDGVIIDNLVQI**

4 M05 100.0% 99.7% **VTITGGSKLRDRVRIHSSTVIGGEGFGFAPYQGKWHRIAQLGSVLIGNDVRIGSNCSIDRGALDNTILEDGVIIDNLVQI**

5 M13 72.8% 99.6% **VTITGGSKLRDRVRIHSSTVIGGEGFGFAPYQGKWHRIAQLGSVLIGNDVRIGSNCSIDRGALDNTILEDGVIIDNLVQI**

6 M16 100.0% 99.7% **VTITGGSKLRDRVRIHSSTVIGGEGFGFAPYQGKWHRIAQLGSVLIGNDVRIGSNCSIDRGALDNTILEDGVIIDNLVQI**

7 M17 100.0% 99.7% **VTITGGSKLRDRVRIHSSTVIGGEGFGFAPYQGKWHRIAQLGSVLIGNDVRIGSNCSIDRGALDNTILEDGVIIDNLVQI**

8 M20 100.0% 99.7% **VTITGGSKLRDRVRIHSSTVIGGEGFGFAPYQGKWHRIAQLGSVLIGNDVRIGSNCSIDRGALDNTILEDGVIIDNLVQI**

9 MS14413 100.0% 99.7% **VTITGGSKLRDRVRIHSSTVIGGEGFGFAPYQGKWHRIAQLGSVLIGNDVRIGSNCSIDRGALDNTILEDGVIIDNLVQI**

10 SQ093 100.0% 99.7% **VTITGGSKLRDRVRIHSSTVIGGEGFGFAPYQGKWHRIAQLGSVLIGNDVRIGSNCSIDRGALDNTILEDGVIIDNLVQI**

11 KAB3 100.0% 99.7% **VTITGGSKLRDRVRIHSSTVIGGEGFGFAPYQGKWHRIAQLGSVLIGNDVRIGSNCSIDRGALDNTILEDGVIIDNLVQI**

12 AC-40 100.0% 99.7% **VTITGGSKLRDRVRIHSSTVIGGEGFGFAPYQGKWHRIAQLGSVLIGNDVRIGSNCSIDRGALDNTILEDGVIIDNLVQI**

13 AC-45 100.0% 99.7% **VTITGGSKLRDRVRIHSSTVIGGEGFGFAPYQGKWHRIAQLGSVLIGNDVRIGSNCSIDRGALDNTILEDGVIIDNLVQI**

14 AC-23 100.0% 99.7% **VTITGGSKLRDRVRIHSSTVIGGEGFGFAPYQGKWHRIAQLGSVLIGNDVRIGSNCSIDRGALDNTILEDGVIIDNLVQI**

15 A21 100.0% 99.7% **VTITGGSKLRDRVRIHSSTVIGGEGFGFAPYQGKWHRIAQLGSVLIGNDVRIGSNCSIDRGALDNTILEDGVIIDNLVQI**

16 SK044 100.0% 99.7% **VTITGGSKLRDRVRIHSSTVIGGEGFGFAPYQGKWHRIAQLGSVLIGNDVRIGSNCSIDRGALDNTILEDGVIIDNLVQI**

17 SK011 100.0% 99.7% **VTITGGSKLRDRVRIHSSTVIGGEGFGFAPYQGKWHRIAQLGSVLIGNDVRIGSNCSIDRGALDNTILEDGVIIDNLVQI**

18 SK002 100.0% 99.7% **VTITGGSKLRDRVRIHSSTVIGGEGFGFAPYQGKWHRIAQLGSVLIGNDVRIGSNCSIDRGALDNTILEDGVIIDNLVQI**

19 PSU120 100.0% 99.7% **VTITGGSKLRDRVRIHSSTVIGGEGFGFAPYQGKWHRIAQLGSVLIGNDVRIGSNCSIDRGALDNTILEDGVIIDNLVQI**

20 KUSSH35 100.0% 99.7% **VTITGGSKLRDRVRIHSSTVIGGEGFGFAPYQGKWHRIAQLGSVLIGNDVRIGSNCSIDRGALDNTILEDGVIIDNLVQI**

21 KUFAR56 100.0% 99.4% **VTITGGSKLRDRVRIHSSTVIGGEGFGFAPYQGKWHRIAQLGSVLIGNDVRIGSNCSIDRGALDNTILEDGVIIDNLVQI**

22 KUSSH36 100.0% 99.7% **VTITGGSKLRDRVRIHSSTVIGGEGFGFAPYQGKWHRIAQLGSVLIGNDVRIGSNCSIDRGALDNTILEDGVIIDNLVQI**

23 130 100.0% 99.7% **VTITGGSKLRDRVRIHSSTVIGGEGFGFAPYQGKWHRIAQLGSVLIGNDVRIGSNCSIDRGALDNTILEDGVIIDNLVQI**

24 Ab34 100.0% 99.7% **VTITGGSKLRDRVRIHSSTVIGGEGFGFAPYQGKWHRIAQLGSVLIGNDVRIGSNCSIDRGALDNTILEDGVIIDNLVQI**

25 Ab36 100.0% 99.7% **VTITGGSKLRDRVRIHSSTVIGGEGFGFAPYQGKWHRIAQLGSVLIGNDVRIGSNCSIDRGALDNTILEDGVIIDNLVQI**

26 Ab40 100.0% 99.7% **VTITGGSKLRDRVRIHSSTVIGGEGFGFAPYQGKWHRIAQLGSVLIGNDVRIGSNCSIDRGALDNTILEDGVIIDNLVQI**

27 Ab15 100.0% 99.7% **VTITGGSKLRDRVRIHSSTVIGGEGFGFAPYQGKWHRIAQLGSVLIGNDVRIGSNCSIDRGALDNTILEDGVIIDNLVQI**

28 Ab65 100.0% 99.7% **VTITGGSKLRDRVRIHSSTVIGGEGFGFAPYQGKWHRIAQLGSVLIGNDVRIGSNCSIDRGALDNTILEDGVIIDNLVQI**

29 AC-14 100.0% 99.7% **VTITGGSKLRDRVRIHSSTVIGGEGFGFAPYQGKWHRIAQLGSVLIGNDVRIGSNCSIDRGALDNTILEDGVIIDNLVQI**

30 SUH-26-2 100.0% 99.7% **VTITGGSKLRDRVRIHSSTVIGGEGFGFAPYQGKWHRIAQLGSVLIGNDVRIGSNCSIDRGALDNTILEDGVIIDNLVQI**

31 SUH-11-2 100.0% 99.7% **VTITGGSKLRDRVRIHSSTVIGGEGFGFAPYQGKWHRIAQLGSVLIGNDVRIGSNCSIDRGALDNTILEDGVIIDNLVQI**

32 SUH-11-1 100.0% 99.7% **VTITGGSKLRDRVRIHSSTVIGGEGFGFAPYQGKWHRIAQLGSVLIGNDVRIGSNCSIDRGALDNTILEDGVIIDNLVQI**

33 KUSSH15 100.0% 99.7% **VTITGGSKLRDRVRIHSSTVIGGEGFGFAPYQGKWHRIAQLGSVLIGNDVRIGSNCSIDRGALDNTILEDGVIIDNLVQI**

34 KUSSH37 100.0% 99.7% **VTITGGSKLRDRVRIHSSTVIGGEGFGFAPYQGKWHRIAQLGSVLIGNDVRIGSNCSIDRGALDNTILEDGVIIDNLVQI**

35 Ab35 100.0% 99.7% **VTITGGSKLRDRVRIHSSTVIGGEGFGFAPYQGKWHRIAQLGSVLIGNDVRIGSNCSIDRGALDNTILEDGVIIDNLVQI**

36 Ab38 100.0% 99.7% **VTITGGSKLRDRVRIHSSTVIGGEGFGFAPYQGKWHRIAQLGSVLIGNDVRIGSNCSIDRGALDNTILEDGVIIDNLVQI**

37 Ab41 100.0% 99.7% **VTITGGSKLRDRVRIHSSTVIGGEGFGFAPYQGKWHRIAQLGSVLIGNDVRIGSNCSIDRGALDNTILEDGVIIDNLVQI**

38 Ab64 100.0% 99.7% **VTITGGSKLRDRVRIHSSTVIGGEGFGFAPYQGKWHRIAQLGSVLIGNDVRIGSNCSIDRGALDNTILEDGVIIDNLVQI**

39 AB22 100.0% 99.7% **VTITGGSKLRDRVRIHSSTVIGGEGFGFAPYQGKWHRIAQLGSVLIGNDVRIGSNCSIDRGALDNTILEDGVIIDNLVQI**

cov pid **241**  **: . . . . 3 . .** **320**

1 ATCC19606 100.0% 100.0% **AHNVHIGSNTAIAAKCGIAGSTKIGKNCILAGACGVAGHLSIADNVTLTGMSMVTKNISEAGTYSSGTGLFENNHWKKTI**

2 M01 100.0% 99.7% **AHNVHIGSNTAIAAKCGIAGSTKIGKNCILAGACGVAGHLSIADNVTLTGMSMVTKNISEAGTYSSGTGLFENNHWKKTI**

3 M04 100.0% 99.7% **AHNVHIGSNTAIAAKCGIAGSTKIGKNCILAGACGVAGHLSIADNVTLTGMSMVTKNISEAGTYSSGTGLFENNHWKKTI**

4 M05 100.0% 99.7% **AHNVHIGSNTAIAAKCGIAGSTKIGKNCILAGACGVAGHLSIADNVTLTGMSMVTKNISEAGTYSSGTGLFENNHWKKTI**

5 M13 72.8% 99.6% **AHNVHIGSNTAIAAKCGIAGSTKIGKNCILAGACGVAGHLSIADNVTLTGMSMVTKNISEAGTYSSGTGLFENNHWKKTI**

6 M16 100.0% 99.7% **AHNVHIGSNTAIAAKCGIAGSTKIGKNCILAGACGVAGHLSIADNVTLTGMSMVTKNISEAGTYSSGTGLFENNHWKKTI**

7 M17 100.0% 99.7% **AHNVHIGSNTAIAAKCGIAGSTKIGKNCILAGACGVAGHLSIADNVTLTGMSMVTKNISEAGTYSSGTGLFENNHWKKTI**

8 M20 100.0% 99.7% **AHNVHIGSNTAIAAKCGIAGSTKIGKNCILAGACGVAGHLSIADNVTLTGMSMVTKNISEAGTYSSGTGLFENNHWKKTI**

9 MS14413 100.0% 99.7% **AHNVHIGSNTAIAAKCGIAGSTKIGKNCILAGACGVAGHLSIADNVTLTGMSMVTKNISEAGTYSSGTGLFENNHWKKTI**

10 SQ093 100.0% 99.7% **AHNVHIGSNTAIAAKCGIAGSTKIGKNCILAGACGVAGHLSIADNVTLTGMSMVTKNISEAGTYSSGTGLFENNHWKKTI**

11 KAB3 100.0% 99.7% **AHNVHIGSNTAIAAKCGIAGSTKIGKNCILAGACGVAGHLSIADNVTLTGMSMVTKNISEAGTYSSGTGLFENNHWKKTI**

12 AC-40 100.0% 99.7% **AHNVHIGSNTAIAAKCGIAGSTKIGKNCILAGACGVAGHLSIADNVTLTGMSMVTKNISEAGTYSSGTGLFENNHWKKTI**

13 AC-45 100.0% 99.7% **AHNVHIGSNTAIAAKCGIAGSTKIGKNCILAGACGVAGHLSIADNVTLTGMSMVTKNISEAGTYSSGTGLFENNHWKKTI**

14 AC-23 100.0% 99.7% **AHNVHIGSNTAIAAKCGIAGSTKIGKNCILAGACGVAGHLSIADNVTLTGMSMVTKNISEAGTYSSGTGLFENNHWKKTI**

15 A21 100.0% 99.7% **AHNVHIGSNTAIAAKCGIAGSTKIGKNCILAGACGVAGHLSIADNVTLTGMSMVTKNISEAGTYSSGTGLFENNHWKKTI**

16 SK044 100.0% 99.7% **AHNVHIGSNTAIAAKCGIAGSTKIGKNCILAGACGVAGHLSIADNVTLTGMSMVTKNISEAGTYSSGTGLFENNHWKKTI**

17 SK011 100.0% 99.7% **AHNVHIGSNTAIAAKCGIAGSTKIGKNCILAGACGVAGHLSIADNVTLTGMSMVTKNISEAGTYSSGTGLFENNHWKKTI**

18 SK002 100.0% 99.7% **AHNVHIGSNTAIAAKCGIAGSTKIGKNCILAGACGVAGHLSIADNVTLTGMSMVTKNISEAGTYSSGTGLFENNHWKKTI**

19 PSU120 100.0% 99.7% **AHNVHIGSNTAIAAKCGIAGSTKIGKNCILAGACGVAGHLSIADNVTLTGMSMVTKNISEAGTYSSGTGLFENNHWKKTI**

20 KUSSH35 100.0% 99.7% **AHNVHIGSNTAIAAKCGIAGSTKIGKNCILAGACGVAGHLSIADNVTLTGMSMVTKNISEAGTYSSGTGLFENNHWKKTI**

21 KUFAR56 100.0% 99.4% **AHNVHIGSNTAIAAKCGIAGSTKIGKNCILAGACGVAGPLSIADNVTLTGMSMVTKNISEAGTYSSGTGLFENNHWKKTI**

22 KUSSH36 100.0% 99.7% **AHNVHIGSNTAIAAKCGIAGSTKIGKNCILAGACGVAGHLSIADNVTLTGMSMVTKNISEAGTYSSGTGLFENNHWKKTI**

23 130 100.0% 99.7% **AHNVHIGSNTAIAAKCGIAGSTKIGKNCILAGACGVAGHLSIADNVTLTGMSMVTKNISEAGTYSSGTGLFENNHWKKTI**

24 Ab34 100.0% 99.7% **AHNVHIGSNTAIAAKCGIAGSTKIGKNCILAGACGVAGHLSIADNVTLTGMSMVTKNISEAGTYSSGTGLFENNHWKKTI**

25 Ab36 100.0% 99.7% **AHNVHIGSNTAIAAKCGIAGSTKIGKNCILAGACGVAGHLSIADNVTLTGMSMVTKNISEAGTYSSGTGLFENNHWKKTI**

26 Ab40 100.0% 99.7% **AHNVHIGSNTAIAAKCGIAGSTKIGKNCILAGACGVAGHLSIADNVTLTGMSMVTKNISEAGTYSSGTGLFENNHWKKTI**

27 Ab15 100.0% 99.7% **AHNVHIGSNTAIAAKCGIAGSTKIGKNCILAGACGVAGHLSIADNVTLTGMSMVTKNISEAGTYSSGTGLFENNHWKKTI**

28 Ab65 100.0% 99.7% **AHNVHIGSNTAIAAKCGIAGSTKIGKNCILAGACGVAGHLSIADNVTLTGMSMVTKNISEAGTYSSGTGLFENNHWKKTI**

29 AC-14 100.0% 99.7% **AHNVHIGSNTAIAAKCGIAGSTKIGKNCILAGACGVAGHLSIADNVTLTGMSMVTKNISEAGTYSSGTGLFENNHWKKTI**

30 SUH-26-2 100.0% 99.7% **AHNVHIGSNTAIAAKCGIAGSTKIGKNCILAGACGVAGHLSIADNVTLTGMSMVTKNISEAGTYSSGTGLFENNHWKKTI**

31 SUH-11-2 100.0% 99.7% **AHNVHIGSNTAIAAKCGIAGSTKIGKNCILAGACGVAGHLSIADNVTLTGMSMVTKNISEAGTYSSGTGLFENNHWKKTI**

32 SUH-11-1 100.0% 99.7% **AHNVHIGSNTAIAAKCGIAGSTKIGKNCILAGACGVAGHLSIADNVTLTGMSMVTKNISEAGTYSSGTGLFENNHWKKTI**

33 KUSSH15 100.0% 99.7% **AHNVHIGSNTAIAAKCGIAGSTKIGKNCILAGACGVAGHLSIADNVTLTGMSMVTKNISEAGTYSSGTGLFENNHWKKTI**

34 KUSSH37 100.0% 99.7% **AHNVHIGSNTAIAAKCGIAGSTKIGKNCILAGACGVAGHLSIADNVTLTGMSMVTKNISEAGTYSSGTGLFENNHWKKTI**

35 Ab35 100.0% 99.7% **AHNVHIGSNTAIAAKCGIAGSTKIGKNCILAGACGVAGHLSIADNVTLTGMSMVTKNISEAGTYSSGTGLFENNHWKKTI**

36 Ab38 100.0% 99.7% **AHNVHIGSNTAIAAKCGIAGSTKIGKNCILAGACGVAGHLSIADNVTLTGMSMVTKNISEAGTYSSGTGLFENNHWKKTI**

37 Ab41 100.0% 99.7% **AHNVHIGSNTAIAAKCGIAGSTKIGKNCILAGACGVAGHLSIADNVTLTGMSMVTKNISEAGTYSSGTGLFENNHWKKTI**

38 Ab64 100.0% 99.7% **AHNVHIGSNTAIAAKCGIAGSTKIGKNCILAGACGVAGHLSIADNVTLTGMSMVTKNISEAGTYSSGTGLFENNHWKKTI**

39 AB22 100.0% 99.7% **AHNVHIGSNTAIAAKCGIAGSTKIGKNCILAGACGVAGHLSIADNVTLTGMSMVTKNISEAGTYSSGTGLFENNHWKKTI**

cov pid **321**  **. . : ]** **356**

1 ATCC19606 100.0% 100.0% **VRLRQLADVPLTQITKRLDHIQAQIESLESTFNLRK**

2 M01 100.0% 99.7% **VRLRQLADVPLTQITKRLDHIQAQIESLESTFNLRK**

3 M04 100.0% 99.7% **VRLRQLADVPLTQITKRLDHIQAQIESLESTFNLRK**

4 M05 100.0% 99.7% **VRLRQLADVPLTQITKRLDHIQAQIESLESTFNLRK**

5 M13 72.8% 99.6% **VRLRQLADVPLTQITKRLDHIQAQIESLESTFNLRK**

6 M16 100.0% 99.7% **VRLRQLADVPLTQITKRLDHIQAQIESLESTFNLRK**

7 M17 100.0% 99.7% **VRLRQLADVPLTQITKRLDHIQAQIESLESTFNLRK**

8 M20 100.0% 99.7% **VRLRQLADVPLTQITKRLDHIQAQIESLESTFNLRK**

9 MS14413 100.0% 99.7% **VRLRQLADVPLTQITKRLDHIQAQIESLESTFNLRK**

10 SQ093 100.0% 99.7% **VRLRQLADVPLTQITKRLDHIQAQIESLESTFNLRK**

11 KAB3 100.0% 99.7% **VRLRQLADVPLTQITKRLDHIQAQIESLESTFNLRK**

12 AC-40 100.0% 99.7% **VRLRQLADVPLTQITKRLDHIQAQIESLESTFNLRK**

13 AC-45 100.0% 99.7% **VRLRQLADVPLTQITKRLDHIQAQIESLESTFNLRK**

14 AC-23 100.0% 99.7% **VRLRQLADVPLTQITKRLDHIQAQIESLESTFNLRK**

15 A21 100.0% 99.7% **VRLRQLADVPLTQITKRLDHIQAQIESLESTFNLRK**

16 SK044 100.0% 99.7% **VRLRQLADVPLTQITKRLDHIQAQIESLESTFNLRK**

17 SK011 100.0% 99.7% **VRLRQLADVPLTQITKRLDHIQAQIESLESTFNLRK**

18 SK002 100.0% 99.7% **VRLRQLADVPLTQITKRLDHIQAQIESLESTFNLRK**

19 PSU120 100.0% 99.7% **VRLRQLADVPLTQITKRLDHIQAQIESLESTFNLRK**

20 KUSSH35 100.0% 99.7% **VRLRQLADVPLTQITKRLDHIQAQIESLESTFNLRK**

21 KUFAR56 100.0% 99.4% **VRLRQLADVPLTQITKRLDHIQAQIESLESTFNLRK**

22 KUSSH36 100.0% 99.7% **VRLRQLADVPLTQITKRLDHIQAQIESLESTFNLRK**

23 130 100.0% 99.7% **VRLRQLADVPLTQITKRLDHIQAQIESLESTFNLRK**

24 Ab34 100.0% 99.7% **VRLRQLADVPLTQITKRLDHIQAQIESLESTFNLRK**

25 Ab36 100.0% 99.7% **VRLRQLADVPLTQITKRLDHIQAQIESLESTFNLRK**

26 Ab40 100.0% 99.7% **VRLRQLADVPLTQITKRLDHIQAQIESLESTFNLRK**

27 Ab15 100.0% 99.7% **VRLRQLADVPLTQITKRLDHIQAQIESLESTFNLRK**

28 Ab65 100.0% 99.7% **VRLRQLADVPLTQITKRLDHIQAQIESLESTFNLRK**

29 AC-14 100.0% 99.7% **VRLRQLADVPLTQITKRLDHIQAQIESLESTFNLRK**

30 SUH-26-2 100.0% 99.7% **VRLRQLADVPLTQITKRLDHIQAQIESLESTFNLRK**

31 SUH-11-2 100.0% 99.7% **VRLRQLADVPLTQITKRLDHIQAQIESLESTFNLRK**

32 SUH-11-1 100.0% 99.7% **VRLRQLADVPLTQITKRLDHIQAQIESLESTFNLRK**

33 KUSSH15 100.0% 99.7% **VRLRQLADVPLTQITKRLDHIQAQIESLESTFNLRK**

34 KUSSH37 100.0% 99.7% **VRLRQLADVPLTQITKRLDHIQAQIESLESTFNLRK**

35 Ab35 100.0% 99.7% **VRLRQLADVPLTQITKRLDHIQAQIESLESTFNLRK**

36 Ab38 100.0% 99.7% **VRLRQLADVPLTQITKRLDHIQAQIESLESTFNLRK**

37 Ab41 100.0% 99.7% **VRLRQLADVPLTQITKRLDHIQAQIESLESTFNLRK**

38 Ab64 100.0% 99.7% **VRLRQLADVPLTQITKRLDHIQAQIESLESTFNLRK**

39 AB22 100.0% 99.7% **VRLRQLADVPLTQITKRLDHIQAQIESLESTFNLRK**

**Supplementary Figure 17: Multiple sequence alignment (MSA) of the predicted amino acid sequence of LpxD carried by ST2^Pas^ and ST570^Pas^ (GC2) isolates and close genomes retrieved from the BV-BRC database compared to the respective gene in *A. baumannii* ATCC 19606.** MSA was created by the A multiple alignment viewer MView hosted by the EMBL-EBI; cov, coverage; pid, percent identity.

cov pid  **1** **[ . . . . : . . .** **80**

1 ATCC19606 100.0% 100.0% **MKVQQYRLDELAHLVKGELIGEGSLQFSNLASLENAEVNHLTFVNGEKHLDQAKVSRAGAYIVTAALKEHLPEKDNFIIV**

2 M03 100.0% 99.4% **MKVQQYRLDELAHLVKGELIGEGSLQFSNLASLENAEVNHLTFVNGEKHLDQAKVSRAGAYIITAALKEHLPEKDNFIIV**

3 M14 100.0% 99.4% **MKVQQYRLDELAHLVKGELIGEGSLQFSNLASLENAEVNHLTFVNGEKHLDQAKVSRAGAYIITAALKEHLPEKDNFIIV**

4 AB_1649-8 100.0% 99.4% **MKVQQYRLDELAHLVKGELIGEGSLQFSNLASLENAEVNHLTFVNGEKHLDQAKVSRAGAYIITAALKEHLPEKDNFIIV**

5 AB_1650-8 100.0% 99.4% **MKVQQYRLDELAHLVKGELIGEGSLQFSNLASLENAEVNHLTFVNGEKHLDQAKVSRAGAYIITAALKEHLPEKDNFIIV**

6 UV_1036 100.0% 99.4% **MKVQQYRLDELAHLVKGELIGEGSLQFSNLASLENAEVNHLTFVNGEKHLDQAKVSRAGAYIITAALKEHLPEKDNFIIV**

7 259_an 100.0% 99.4% **MKVQQYRLDELAHLVKGELIGEGSLQFSNLASLENAEVNHLTFVNGEKHLDQAKVSRAGAYIITAALKEHLPEKDNFIIV**

8 276_ax 100.0% 99.4% **MKVQQYRLDELAHLVKGELIGEGSLQFSNLASLENAEVNHLTFVNGEKHLDQAKVSRAGAYIITAALKEHLPEKDNFIIV**

9 PT061 100.0% 99.4% **MKVQQYRLDELAHLVKGELIGEGSLQFSNLASLENAEVNHLTFVNGEKHLDQAKVSRAGAYIITAALKEHLPEKDNFIIV**

10 PT003 100.0% 99.4% **MKVQQYRLDELAHLVKGELIGEGSLQFSNLASLENAEVNHLTFVNGEKHLDQAKVSRAGAYIITAALKEHLPEKDNFIIV**

11 PSU068 100.0% 99.4% **MKVQQYRLDELAHLVKGELIGEGSLQFSNLASLENAEVNHLTFVNGEKHLDQAKVSRAGAYIITAALKEHLPEKDNFIIV**

12 PSU073 100.0% 99.4% **MKVQQYRLDELAHLVKGELIGEGSLQFSNLASLENAEVNHLTFVNGEKHLDQAKVSRAGAYIITAALKEHLPEKDNFIIV**

13 CCBH26501 100.0% 98.9% **MKLKQYRLDELAHLVKGELIGEGSLQFSNLASLENAEVNHLTFVNGEKHLDQAKVSRAGAYIITAALKEHLPEKDNFIIV**

14 AB363 100.0% 99.4% **MKVQQYRLDELAHLVKGELIGEGSLQFSNLASLENAEVNHLTFVNGEKHLDQAKVSRAGAYIITAALKEHLPEKDNFIIV**

15 KUSSH08 100.0% 99.4% **MKVQQYRLDELAHLVKGELIGEGSLQFSNLASLENAEVNHLTFVNGEKHLDQAKVSRAGAYIITAALKEHLPEKDNFIIV**

16 KUSSH14 100.0% 99.4% **MKVQQYRLDELAHLVKGELIGEGSLQFSNLASLENAEVNHLTFVNGEKHLDQAKVSRAGAYIITAALKEHLPEKDNFIIV**

17 TUMA 100.0% 99.4% **MKVQQYRLDELAHLVKGELIGEGSLQFSNLASLENAEVNHLTFVNGEKHLDQAKVSRAGAYIITAALKEHLPEKDNFIIV**

18 4300STDY7045886 100.0% 99.4% **MKVQQYRLDELAHLVKGELIGEGSLQFSNLASLENAEVNHLTFVNGEKHLDQAKVSRAGAYIITAALKEHLPEKDNFIIV**

19 Aci00866 100.0% 99.4% **MKVQQYRLDELAHLVKGELIGEGSLQFSNLASLENAEVNHLTFVNGEKHLDQAKVSRAGAYIITAALKEHLPEKDNFIIV**

20 Aci00860 100.0% 99.4% **MKVQQYRLDELAHLVKGELIGEGSLQFSNLASLENAEVNHLTFVNGEKHLDQAKVSRAGAYIITAALKEHLPEKDNFIIV**

21 Aci00848 100.0% 99.4% **MKVQQYRLDELAHLVKGELIGEGSLQFSNLASLENAEVNHLTFVNGEKHLDQAKVSRAGAYIITAALKEHLPEKDNFIIV**

22 MRSN351524 100.0% 99.4% **MKVQQYRLDELAHLVKGELIGEGSLQFSNLASLENAEVNHLTFVNGEKHLDQAKVSRAGAYIITAALKEHLPEKDNFIIV**

cov pid  **81**  **. 1 . . . . : .** **160**

1 ATCC19606 100.0% 100.0% **DNPYLAFAILTHVFDKKISSTGIESTAQIHPSAVISETAYIGHYVVIGENCVVGDNTVIQSHTKLDDNVEVGKDCFIDSH**

2 M03 100.0% 99.4% **DNPYLAFAILTHVFDKKISSTGIESTAQIHPSAVISETAYIGHYVVIGENCVVGDNTVIQSHTKLDDNVEVGKDCFIDSH**

3 M14 100.0% 99.4% **DNPYLAFAILTHVFDKKISSTGIESTAQIHPSAVISETAYIGHYVVIGENCVVGDNTVIQSHTKLDDNVEVGKDCFIDSH**

4 AB_1649-8 100.0% 99.4% **DNPYLAFAILTHVFDKKISSTGIESTAQIHPSAVISETAYIGHYVVIGENCVVGDNTVIQSHTKLDDNVEVGKDCFIDSH**

5 AB_1650-8 100.0% 99.4% **DNPYLAFAILTHVFDKKISSTGIESTAQIHPSAVISETAYIGHYVVIGENCVVGDNTVIQSHTKLDDNVEVGKDCFIDSH**

6 UV_1036 100.0% 99.4% **DNPYLAFAILTHVFDKKISSTGIESTAQIHPSAVISETAYIGHYVVIGENCVVGDNTVIQSHTKLDDNVEVGKDCFIDSH**

7 259_an 100.0% 99.4% **DNPYLAFAILTHVFDKKISSTGIESTAQIHPSAVISETAYIGHYVVIGENCVVGDNTVIQSHTKLDDNVEVGKDCFIDSH**

8 276_ax 100.0% 99.4% **DNPYLAFAILTHVFDKKISSTGIESTAQIHPSAVISETAYIGHYVVIGENCVVGDNTVIQSHTKLDDNVEVGKDCFIDSH**

9 PT061 100.0% 99.4% **DNPYLAFAILTHVFDKKISSTGIESTAQIHPSAVISETAYIGHYVVIGENCVVGDNTVIQSHTKLDDNVEVGKDCFIDSH**

10 PT003 100.0% 99.4% **DNPYLAFAILTHVFDKKISSTGIESTAQIHPSAVISETAYIGHYVVIGENCVVGDNTVIQSHTKLDDNVEVGKDCFIDSH**

11 PSU068 100.0% 99.4% **DNPYLAFAILTHVFDKKISSTGIESTAQIHPSAVISETAYIGHYVVIGENCVVGDNTVIQSHTKLDDNVEVGKDCFIDSH**

12 PSU073 100.0% 99.4% **DNPYLAFAILTHVFDKKISSTGIESTAQIHPSAVISETAYIGHYVVIGENCVVGDNTVIQSHTKLDDNVEVGKDCFIDSH**

13 CCBH26501 100.0% 98.9% **DNPYLAFAILTHVFDKKISSTGIESTAQIHPSAVISETAYIGHYVVIGENCVVGDNTVIQSHTKLDDNVEVGKDCFIDSH**

14 AB363 100.0% 99.4% **DNPYLAFAILTHVFDKKISSTGIESTAQIHPSAVISETAYIGHYVVIGENCVVGDNTVIQSHTKLDDNVEVGKDCFIDSH**

15 KUSSH08 100.0% 99.4% **DNPYLAFAILTHVFDKKISSTGIESTAQIHPSAVISETAYIGHYVVIGENCVVGDNTVIQSHTKLDDNVEVGKDCFIDSH**

16 KUSSH14 100.0% 99.4% **DNPYLAFAILTHVFDKKISSTGIESTAQIHPSAVISETAYIGHYVVIGENCVVGDNTVIQSHTKLDDNVEVGKDCFIDSH**

17 TUMA 100.0% 99.4% **DNPYLAFAILTHVFDKKISSTGIESTAQIHPSAVISETAYIGHYVVIGENCVVGDNTVIQSHTKLDDNVEVGKDCFIDSH**

18 4300STDY7045886 100.0% 99.4% **DNPYLAFAILTHVFDKKISSTGIESTAQIHPSAVISETAYIGHYVVIGENCVVGDNTVIQSHTKLDDNVEVGKDCFIDSH**

19 Aci00866 100.0% 99.4% **DNPYLAFAILTHVFDKKISSTGIESTAQIHPSAVISETAYIGHYVVIGENCVVGDNTVIQSHTKLDDNVEVGKDCFIDSH**

20 Aci00860 100.0% 99.4% **DNPYLAFAILTHVFDKKISSTGIESTAQIHPSAVISETAYIGHYVVIGENCVVGDNTVIQSHTKLDDNVEVGKDCFIDSH**

21 Aci00848 100.0% 99.4% **DNPYLAFAILTHVFDKKISSTGIESTAQIHPSAVISETAYIGHYVVIGENCVVGDNTVIQSHTKLDDNVEVGKDCFIDSH**

22 MRSN351524 100.0% 99.4% **DNPYLAFAILTHVFDKKISSTGIESTAQIHPSAVISETAYIGHYVVIGENCVVGDNTVIQSHTKLDDNVEVGKDCFIDSH**

cov pid **161**  **. . . 2 . . . .** **240**

1 ATCC19606 100.0% 100.0% **VTITGGSKLRDRVRIHSSTVIGGEGFGFAPYQGKWHRIAQLGSVLIGNDVRIGSNCSIDRGALDNTILEDGVIIDNLVQI**

2 M03 100.0% 99.4% **VTITGSSKLRDRVRIHSSTVIGGEGFGFAPYQGKWHRIAQLGSVLIGNDVRIGSNCSIDRGALDNTILEDGVIIDNLVQI**

3 M14 100.0% 99.4% **VTITGSSKLRDRVRIHSSTVIGGEGFGFAPYQGKWHRIAQLGSVLIGNDVRIGSNCSIDRGALDNTILEDGVIIDNLVQI**

4 AB_1649-8 100.0% 99.4% **VTITGSSKLRDRVRIHSSTVIGGEGFGFAPYQGKWHRIAQLGSVLIGNDVRIGSNCSIDRGALDNTILEDGVIIDNLVQI**

5 AB_1650-8 100.0% 99.4% **VTITGSSKLRDRVRIHSSTVIGGEGFGFAPYQGKWHRIAQLGSVLIGNDVRIGSNCSIDRGALDNTILEDGVIIDNLVQI**

6 UV_1036 100.0% 99.4% **VTITGSSKLRDRVRIHSSTVIGGEGFGFAPYQGKWHRIAQLGSVLIGNDVRIGSNCSIDRGALDNTILEDGVIIDNLVQI**

7 259_an 100.0% 99.4% **VTITGSSKLRDRVRIHSSTVIGGEGFGFAPYQGKWHRIAQLGSVLIGNDVRIGSNCSIDRGALDNTILEDGVIIDNLVQI**

8 276_ax 100.0% 99.4% **VTITGSSKLRDRVRIHSSTVIGGEGFGFAPYQGKWHRIAQLGSVLIGNDVRIGSNCSIDRGALDNTILEDGVIIDNLVQI**

9 PT061 100.0% 99.4% **VTITGSSKLRDRVRIHSSTVIGGEGFGFAPYQGKWHRIAQLGSVLIGNDVRIGSNCSIDRGALDNTILEDGVIIDNLVQI**

10 PT003 100.0% 99.4% **VTITGSSKLRDRVRIHSSTVIGGEGFGFAPYQGKWHRIAQLGSVLIGNDVRIGSNCSIDRGALDNTILEDGVIIDNLVQI**

11 PSU068 100.0% 99.4% **VTITGSSKLRDRVRIHSSTVIGGEGFGFAPYQGKWHRIAQLGSVLIGNDVRIGSNCSIDRGALDNTILEDGVIIDNLVQI**

12 PSU073 100.0% 99.4% **VTITGSSKLRDRVRIHSSTVIGGEGFGFAPYQGKWHRIAQLGSVLIGNDVRIGSNCSIDRGALDNTILEDGVIIDNLVQI**

13 CCBH26501 100.0% 98.9% **VTITGSSKLRDRVRIHSSTVIGGEGFGFAPYQGKWHRIAQLGSVLIGNDVRIGSNCSIDRGALDNTILEDGVIIDNLVQI**

14 AB363 100.0% 99.4% **VTITGSSKLRDRVRIHSSTVIGGEGFGFAPYQGKWHRIAQLGSVLIGNDVRIGSNCSIDRGALDNTILEDGVIIDNLVQI**

15 KUSSH08 100.0% 99.4% **VTITGSSKLRDRVRIHSSTVIGGEGFGFAPYQGKWHRIAQLGSVLIGNDVRIGSNCSIDRGALDNTILEDGVIIDNLVQI**

16 KUSSH14 100.0% 99.4% **VTITGSSKLRDRVRIHSSTVIGGEGFGFAPYQGKWHRIAQLGSVLIGNDVRIGSNCSIDRGALDNTILEDGVIIDNLVQI**

17 TUMA 100.0% 99.4% **VTITGSSKLRDRVRIHSSTVIGGEGFGFAPYQGKWHRIAQLGSVLIGNDVRIGSNCSIDRGALDNTILEDGVIIDNLVQI**

18 4300STDY7045886 100.0% 99.4% **VTITGSSKLRDRVRIHSSTVIGGEGFGFAPYQGKWHRIAQLGSVLIGNDVRIGSNCSIDRGALDNTILEDGVIIDNLVQI**

19 Aci00866 100.0% 99.4% **VTITGSSKLRDRVRIHSSTVIGGEGFGFAPYQGKWHRIAQLGSVLIGNDVRIGSNCSIDRGALDNTILEDGVIIDNLVQI**

20 Aci00860 100.0% 99.4% **VTITGSSKLRDRVRIHSSTVIGGEGFGFAPYQGKWHRIAQLGSVLIGNDVRIGSNCSIDRGALDNTILEDGVIIDNLVQI**

21 Aci00848 100.0% 99.4% **VTITGSSKLRDRVRIHSSTVIGGEGFGFAPYQGKWHRIAQLGSVLIGNDVRIGSNCSIDRGALDNTILEDGVIIDNLVQI**

22 MRSN351524 100.0% 99.4% **VTITGSSKLRDRVRIHSSTVIGGEGFGFAPYQGKWHRIAQLGSVLIGNDVRIGSNCSIDRGALDNTILEDGVIIDNLVQI**

cov pid **241**  **: . . . . 3 . .** **320**

1 ATCC19606 100.0% 100.0% **AHNVHIGSNTAIAAKCGIAGSTKIGKNCILAGACGVAGHLSIADNVTLTGMSMVTKNISEAGTYSSGTGLFENNHWKKTI**

2 M03 100.0% 99.4% **AHNVHIGSNTAIAAKCGIAGSTKIGKNCILAGACGVAGHLSIADNVTLTGMSMVTKNISEAGTYSSGTGLFENNHWKKTI**

3 M14 100.0% 99.4% **AHNVHIGSNTAIAAKCGIAGSTKIGKNCILAGACGVAGHLSIADNVTLTGMSMVTKNISEAGTYSSGTGLFENNHWKKTI**

4 AB_1649-8 100.0% 99.4% **AHNVHIGSNTAIAAKCGIAGSTKIGKNCILAGACGVAGHLSIADNVTLTGMSMVTKNISEAGTYSSGTGLFENNHWKKTI**

5 AB_1650-8 100.0% 99.4% **AHNVHIGSNTAIAAKCGIAGSTKIGKNCILAGACGVAGHLSIADNVTLTGMSMVTKNISEAGTYSSGTGLFENNHWKKTI**

6 UV_1036 100.0% 99.4% **AHNVHIGSNTAIAAKCGIAGSTKIGKNCILAGACGVAGHLSIADNVTLTGMSMVTKNISEAGTYSSGTGLFENNHWKKTI**

7 259_an 100.0% 99.4% **AHNVHIGSNTAIAAKCGIAGSTKIGKNCILAGACGVAGHLSIADNVTLTGMSMVTKNISEAGTYSSGTGLFENNHWKKTI**

8 276_ax 100.0% 99.4% **AHNVHIGSNTAIAAKCGIAGSTKIGKNCILAGACGVAGHLSIADNVTLTGMSMVTKNISEAGTYSSGTGLFENNHWKKTI**

9 PT061 100.0% 99.4% **AHNVHIGSNTAIAAKCGIAGSTKIGKNCILAGACGVAGHLSIADNVTLTGMSMVTKNISEAGTYSSGTGLFENNHWKKTI**

10 PT003 100.0% 99.4% **AHNVHIGSNTAIAAKCGIAGSTKIGKNCILAGACGVAGHLSIADNVTLTGMSMVTKNISEAGTYSSGTGLFENNHWKKTI**

11 PSU068 100.0% 99.4% **AHNVHIGSNTAIAAKCGIAGSTKIGKNCILAGACGVAGHLSIADNVTLTGMSMVTKNISEAGTYSSGTGLFENNHWKKTI**

12 PSU073 100.0% 99.4% **AHNVHIGSNTAIAAKCGIAGSTKIGKNCILAGACGVAGHLSIADNVTLTGMSMVTKNISEAGTYSSGTGLFENNHWKKTI**

13 CCBH26501 100.0% 98.9% **AHNVHIGSNTAIAAKCGIAGSTKIGKNCILAGACGVAGHLSIADNVTLTGMSMVTKNISEAGTYSSGTGLFENNHWKKTI**

14 AB363 100.0% 99.4% **AHNVHIGSNTAIAAKCGIAGSTKIGKNCILAGACGVAGHLSIADNVTLTGMSMVTKNISEAGTYSSGTGLFENNHWKKTI**

15 KUSSH08 100.0% 99.4% **AHNVHIGSNTAIAAKCGIAGSTKIGKNCILAGACGVAGHLSIADNVTLTGMSMVTKNISEAGTYSSGTGLFENNHWKKTI**

16 KUSSH14 100.0% 99.4% **AHNVHIGSNTAIAAKCGIAGSTKIGKNCILAGACGVAGHLSIADNVTLTGMSMVTKNISEAGTYSSGTGLFENNHWKKTI**

17 TUMA 100.0% 99.4% **AHNVHIGSNTAIAAKCGIAGSTKIGKNCILAGACGVAGHLSIADNVTLTGMSMVTKNISEAGTYSSGTGLFENNHWKKTI**

18 4300STDY7045886 100.0% 99.4% **AHNVHIGSNTAIAAKCGIAGSTKIGKNCILAGACGVAGHLSIADNVTLTGMSMVTKNISEAGTYSSGTGLFENNHWKKTI**

19 Aci00866 100.0% 99.4% **AHNVHIGSNTAIAAKCGIAGSTKIGKNCILAGACGVAGHLSIADNVTLTGMSMVTKNISEAGTYSSGTGLFENNHWKKTI**

20 Aci00860 100.0% 99.4% **AHNVHIGSNTAIAAKCGIAGSTKIGKNCILAGACGVAGHLSIADNVTLTGMSMVTKNISEAGTYSSGTGLFENNHWKKTI**

21 Aci00848 100.0% 99.4% **AHNVHIGSNTAIAAKCGIAGSTKIGKNCILAGACGVAGHLSIADNVTLTGMSMVTKNISEAGTYSSGTGLFENNHWKKTI**

22 MRSN351524 100.0% 99.4% **AHNVHIGSNTAIAAKCGIAGSTKIGKNCILAGACGVAGHLSIADNVTLTGMSMVTKNISEAGTYSSGTGLFENNHWKKTI**

cov pid **321**  **. . : ]** **356**

1 ATCC19606 100.0% 100.0% **VRLRQLADVPLTQITKRLDHIQAQIESLESTFNLRK**

2 M03 100.0% 99.4% **VRLRQLADVPLTQITKRLDHIQAQIESLESTFNLRK**

3 M14 100.0% 99.4% **VRLRQLADVPLTQITKRLDHIQAQIESLESTFNLRK**

4 AB_1649-8 100.0% 99.4% **VRLRQLADVPLTQITKRLDHIQAQIESLESTFNLRK**

5 AB_1650-8 100.0% 99.4% **VRLRQLADVPLTQITKRLDHIQAQIESLESTFNLRK**

6 UV_1036 100.0% 99.4% **VRLRQLADVPLTQITKRLDHIQAQIESLESTFNLRK**

7 259_an 100.0% 99.4% **VRLRQLADVPLTQITKRLDHIQAQIESLESTFNLRK**

8 276_ax 100.0% 99.4% **VRLRQLADVPLTQITKRLDHIQAQIESLESTFNLRK**

9 PT061 100.0% 99.4% **VRLRQLADVPLTQITKRLDHIQAQIESLESTFNLRK**

10 PT003 100.0% 99.4% **VRLRQLADVPLTQITKRLDHIQAQIESLESTFNLRK**

11 PSU068 100.0% 99.4% **VRLRQLADVPLTQITKRLDHIQAQIESLESTFNLRK**

12 PSU073 100.0% 99.4% **VRLRQLADVPLTQITKRLDHIQAQIESLESTFNLRK**

13 CCBH26501 100.0% 98.9% **VRLRQLADVPLTQITKRLDHIQAQIESLESTFNLRK**

14 AB363 100.0% 99.4% **VRLRQLADVPLTQITKRLDHIQAQIESLESTFNLRK**

15 KUSSH08 100.0% 99.4% **VRLRQLADVPLTQITKRLDHIQAQIESLESTFNLRK**

16 KUSSH14 100.0% 99.4% **VRLRQLADVPLTQITKRLDHIQAQIESLESTFNLRK**

17 TUMA 100.0% 99.4% **VRLRQLADVPLTQITKRLDHIQAQIESLESTFNLRK**

18 4300STDY7045886 100.0% 99.4% **VRLRQLADVPLTQITKRLDHIQAQIESLESTFNLRK**

19 Aci00866 100.0% 99.4% **VRLRQLADVPLTQITKRLDHIQAQIESLESTFNLRK**

20 Aci00860 100.0% 99.4% **VRLRQLADVPLTQITKRLDHIQAQIESLESTFNLRK**

21 Aci00848 100.0% 99.4% **VRLRQLADVPLTQITKRLDHIQAQIESLESTFNLRK**

22 MRSN351524 100.0% 99.4% **VRLRQLADVPLTQITKRLDHIQAQIESLESTFNLRK**

**Supplementary Figure 18: Multiple sequence alignment (MSA) of the predicted amino acid sequence of LpxD carried by ST113^Pas^ (GC7) isolates and close genomes retrieved from the BV-BRC database compared to the respective gene in *A. baumannii* ATCC 19606.** MSA was created by the A multiple alignment viewer MView hosted by the EMBL-EBI; cov, coverage; pid, percent identity.

cov pid  **1** **[ . . . . : . . .** **80**

1 ATCC19606 100.0% 100.0% **MKVQQYRLDELAHLVKGELIGEGSLQFSNLASLENAEVNHLTFVNGEKHLDQAKVSRAGAYIVTAALKEHLPEKDNFIIV**

2 M10 68.8% 100.0% **--------------------------------------------------------------------------------**

3 A5 100.0% 99.7% **MKVQQYRLDELAHLVKGELIGEGSLQFSNLASLENAEVNHLTFVNGEKHLDQAKVSRAGAYIITAALKEHLPEKDNFIIV**

4 Aci00709 100.0% 99.7% **MKVQQYRLDELAHLVKGELIGEGSLQFSNLASLENAEVNHLTFVNGEKHLDQAKVSRAGAYIITAALKEHLPEKDNFIIV**

5 Aci00711 100.0% 99.7% **MKVQQYRLDELAHLVKGELIGEGSLQFSNLASLENAEVNHLTFVNGEKHLDQAKVSRAGAYIITAALKEHLPEKDNFIIV**

6 KAB15 100.0% 99.7% **MKVQQYRLDELAHLVKGELIGEGSLQFSNLASLENAEVNHLTFVNGEKHLDQAKVSRAGAYIITAALKEHLPEKDNFIIV**

7 M19 100.0% 99.7% **MKVQQYRLDELAHLVKGELIGEGSLQFSNLASLENAEVNHLTFVNGEKHLDQAKVSRAGAYIITAALKEHLPEKDNFIIV**

8 PT022 100.0% 99.7% **MKVQQYRLDELAHLVKGELIGEGSLQFSNLASLENAEVNHLTFVNGEKHLDQAKVSRAGAYIITAALKEHLPEKDNFIIV**

9 PSU091 100.0% 99.7% **MKVQQYRLDELAHLVKGELIGEGSLQFSNLASLENAEVNHLTFVNGEKHLDQAKVSRAGAYIITAALKEHLPEKDNFIIV**

10 151 100.0% 99.7% **MKVQQYRLDELAHLVKGELIGEGSLQFSNLASLENAEVNHLTFVNGEKHLDQAKVSRAGAYIITAALKEHLPEKDNFIIV**

11 GML-KP48-AB-TR 100.0% 99.7% **MKVQQYRLDELAHLVKGELIGEGSLQFSNLASLENAEVNHLTFVNGEKHLDQAKVSRAGAYIITAALKEHLPEKDNFIIV**

12 198 100.0% 99.7% **MKVQQYRLDELAHLVKGELIGEGSLQFSNLASLENAEVNHLTFVNGEKHLDQAKVSRAGAYIITAALKEHLPEKDNFIIV**

13 4300STDY7045706 100.0% 99.7% **MKVQQYRLDELAHLVKGELIGEGSLQFSNLASLENAEVNHLTFVNGEKHLDQAKVSRAGAYIITAALKEHLPEKDNFIIV**

14 4300STDY7045763 100.0% 99.7% **MKVQQYRLDELAHLVKGELIGEGSLQFSNLASLENAEVNHLTFVNGEKHLDQAKVSRAGAYIITAALKEHLPEKDNFIIV**

15 4300STDY7045798 100.0% 99.7% **MKVQQYRLDELAHLVKGELIGEGSLQFSNLASLENAEVNHLTFVNGEKHLDQAKVSRAGAYIITAALKEHLPEKDNFIIV**

16 4300STDY7045799 100.0% 99.7% **MKVQQYRLDELAHLVKGELIGEGSLQFSNLASLENAEVNHLTFVNGEKHLDQAKVSRAGAYIITAALKEHLPEKDNFIIV**

17 4300STDY7045808 100.0% 99.7% **MKVQQYRLDELAHLVKGELIGEGSLQFSNLASLENAEVNHLTFVNGEKHLDQAKVSRAGAYIITAALKEHLPEKDNFIIV**

18 4300STDY7045806 100.0% 99.7% **MKVQQYRLDELAHLVKGELIGEGSLQFSNLASLENAEVNHLTFVNGEKHLDQAKVSRAGAYIITAALKEHLPEKDNFIIV**

19 4300STDY7045811 100.0% 99.7% **MKVQQYRLDELAHLVKGELIGEGSLQFSNLASLENAEVNHLTFVNGEKHLDQAKVSRAGAYIITAALKEHLPEKDNFIIV**

20 4300STDY7045829 100.0% 99.7% **MKVQQYRLDELAHLVKGELIGEGSLQFSNLASLENAEVNHLTFVNGEKHLDQAKVSRAGAYIITAALKEHLPEKDNFIIV**

21 4300STDY7045870 100.0% 99.7% **MKVQQYRLDELAHLVKGELIGEGSLQFSNLASLENAEVNHLTFVNGEKHLDQAKVSRAGAYIITAALKEHLPEKDNFIIV**

22 4300STDY7045866 100.0% 99.7% **MKVQQYRLDELAHLVKGELIGEGSLQFSNLASLENAEVNHLTFVNGEKHLDQAKVSRAGAYIITAALKEHLPEKDNFIIV**

23 4300STDY6542380 100.0% 99.7% **MKVQQYRLDELAHLVKGELIGEGSLQFSNLASLENAEVNHLTFVNGEKHLDQAKVSRAGAYIITAALKEHLPEKDNFIIV**

cov pid  **81**  **. 1 . . . . : .** **160**

1 ATCC19606 100.0% 100.0% **DNPYLAFAILTHVFDKKISSTGIESTAQIHPSAVISETAYIGHYVVIGENCVVGDNTVIQSHTKLDDNVEVGKDCFIDSH**

2 M10 68.8% 100.0% **-------------------------------SAVISETAYIGHYVVIGENCVVGDNTVIQSHTKLDDNVEVGKDCFIDSH**

3 A5 100.0% 99.7% **DNPYLAFAILTHVFDKKISSTGIESTAQIHPSAVISETAYIGHYVVIGENCVVGDNTVIQSHTKLDDNVEVGKDCFIDSH**

4 Aci00709 100.0% 99.7% **DNPYLAFAILTHVFDKKISSTGIESTAQIHPSAVISETAYIGHYVVIGENCVVGDNTVIQSHTKLDDNVEVGKDCFIDSH**

5 Aci00711 100.0% 99.7% **DNPYLAFAILTHVFDKKISSTGIESTAQIHPSAVISETAYIGHYVVIGENCVVGDNTVIQSHTKLDDNVEVGKDCFIDSH**

6 KAB15 100.0% 99.7% **DNPYLAFAILTHVFDKKISSTGIESTAQIHPSAVISETAYIGHYVVIGENCVVGDNTVIQSHTKLDDNVEVGKDCFIDSH**

7 M19 100.0% 99.7% **DNPYLAFAILTHVFDKKISSTGIESTAQIHPSAVISETAYIGHYVVIGENCVVGDNTVIQSHTKLDDNVEVGKDCFIDSH**

8 PT022 100.0% 99.7% **DNPYLAFAILTHVFDKKISSTGIESTAQIHPSAVISETAYIGHYVVIGENCVVGDNTVIQSHTKLDDNVEVGKDCFIDSH**

9 PSU091 100.0% 99.7% **DNPYLAFAILTHVFDKKISSTGIESTAQIHPSAVISETAYIGHYVVIGENCVVGDNTVIQSHTKLDDNVEVGKDCFIDSH**

10 151 100.0% 99.7% **DNPYLAFAILTHVFDKKISSTGIESTAQIHPSAVISETAYIGHYVVIGENCVVGDNTVIQSHTKLDDNVEVGKDCFIDSH**

11 GML-KP48-AB-TR 100.0% 99.7% **DNPYLAFAILTHVFDKKISSTGIESTAQIHPSAVISETAYIGHYVVIGENCVVGDNTVIQSHTKLDDNVEVGKDCFIDSH**

12 198 100.0% 99.7% **DNPYLAFAILTHVFDKKISSTGIESTAQIHPSAVISETAYIGHYVVIGENCVVGDNTVIQSHTKLDDNVEVGKDCFIDSH**

13 4300STDY7045706 100.0% 99.7% **DNPYLAFAILTHVFDKKISSTGIESTAQIHPSAVISETAYIGHYVVIGENCVVGDNTVIQSHTKLDDNVEVGKDCFIDSH**

14 4300STDY7045763 100.0% 99.7% **DNPYLAFAILTHVFDKKISSTGIESTAQIHPSAVISETAYIGHYVVIGENCVVGDNTVIQSHTKLDDNVEVGKDCFIDSH**

15 4300STDY7045798 100.0% 99.7% **DNPYLAFAILTHVFDKKISSTGIESTAQIHPSAVISETAYIGHYVVIGENCVVGDNTVIQSHTKLDDNVEVGKDCFIDSH**

16 4300STDY7045799 100.0% 99.7% **DNPYLAFAILTHVFDKKISSTGIESTAQIHPSAVISETAYIGHYVVIGENCVVGDNTVIQSHTKLDDNVEVGKDCFIDSH**

17 4300STDY7045808 100.0% 99.7% **DNPYLAFAILTHVFDKKISSTGIESTAQIHPSAVISETAYIGHYVVIGENCVVGDNTVIQSHTKLDDNVEVGKDCFIDSH**

18 4300STDY7045806 100.0% 99.7% **DNPYLAFAILTHVFDKKISSTGIESTAQIHPSAVISETAYIGHYVVIGENCVVGDNTVIQSHTKLDDNVEVGKDCFIDSH**

19 4300STDY7045811 100.0% 99.7% **DNPYLAFAILTHVFDKKISSTGIESTAQIHPSAVISETAYIGHYVVIGENCVVGDNTVIQSHTKLDDNVEVGKDCFIDSH**

20 4300STDY7045829 100.0% 99.7% **DNPYLAFAILTHVFDKKISSTGIESTAQIHPSAVISETAYIGHYVVIGENCVVGDNTVIQSHTKLDDNVEVGKDCFIDSH**

21 4300STDY7045870 100.0% 99.7% **DNPYLAFAILTHVFDKKISSTGIESTAQIHPSAVISETAYIGHYVVIGENCVVGDNTVIQSHTKLDDNVEVGKDCFIDSH**

22 4300STDY7045866 100.0% 99.7% **DNPYLAFAILTHVFDKKISSTGIESTAQIHPSAVISETAYIGHYVVIGENCVVGDNTVIQSHTKLDDNVEVGKDCFIDSH**

23 4300STDY6542380 100.0% 99.7% **DNPYLAFAILTHVFDKKISSTGIESTAQIHPSAVISETAYIGHYVVIGENCVVGDNTVIQSHTKLDDNVEVGKDCFIDSH**

cov pid **161**  **. . . 2 . . . .** **240**

1 ATCC19606 100.0% 100.0% **VTITGGSKLRDRVRIHSSTVIGGEGFGFAPYQGKWHRIAQLGSVLIGNDVRIGSNCSIDRGALDNTILEDGVIIDNLVQI**

2 M10 68.8% 100.0% **VTITGGSKLRDRVRIHSSTVIGGEGFGFAPYQGKWHRIAQLGSVLIGNDVRIGSNCSIDRGALDNTILEDGVIIDNLVQI**

3 A5 100.0% 99.7% **VTITGGSKLRDRVRIHSSTVIGGEGFGFAPYQGKWHRIAQLGSVLIGNDVRIGSNCSIDRGALDNTILEDGVIIDNLVQI**

4 Aci00709 100.0% 99.7% **VTITGGSKLRDRVRIHSSTVIGGEGFGFAPYQGKWHRIAQLGSVLIGNDVRIGSNCSIDRGALDNTILEDGVIIDNLVQI**

5 Aci00711 100.0% 99.7% **VTITGGSKLRDRVRIHSSTVIGGEGFGFAPYQGKWHRIAQLGSVLIGNDVRIGSNCSIDRGALDNTILEDGVIIDNLVQI**

6 KAB15 100.0% 99.7% **VTITGGSKLRDRVRIHSSTVIGGEGFGFAPYQGKWHRIAQLGSVLIGNDVRIGSNCSIDRGALDNTILEDGVIIDNLVQI**

7 M19 100.0% 99.7% **VTITGGSKLRDRVRIHSSTVIGGEGFGFAPYQGKWHRIAQLGSVLIGNDVRIGSNCSIDRGALDNTILEDGVIIDNLVQI**

8 PT022 100.0% 99.7% **VTITGGSKLRDRVRIHSSTVIGGEGFGFAPYQGKWHRIAQLGSVLIGNDVRIGSNCSIDRGALDNTILEDGVIIDNLVQI**

9 PSU091 100.0% 99.7% **VTITGGSKLRDRVRIHSSTVIGGEGFGFAPYQGKWHRIAQLGSVLIGNDVRIGSNCSIDRGALDNTILEDGVIIDNLVQI**

10 151 100.0% 99.7% **VTITGGSKLRDRVRIHSSTVIGGEGFGFAPYQGKWHRIAQLGSVLIGNDVRIGSNCSIDRGALDNTILEDGVIIDNLVQI**

11 GML-KP48-AB-TR 100.0% 99.7% **VTITGGSKLRDRVRIHSSTVIGGEGFGFAPYQGKWHRIAQLGSVLIGNDVRIGSNCSIDRGALDNTILEDGVIIDNLVQI**

12 198 100.0% 99.7% **VTITGGSKLRDRVRIHSSTVIGGEGFGFAPYQGKWHRIAQLGSVLIGNDVRIGSNCSIDRGALDNTILEDGVIIDNLVQI**

13 4300STDY7045706 100.0% 99.7% **VTITGGSKLRDRVRIHSSTVIGGEGFGFAPYQGKWHRIAQLGSVLIGNDVRIGSNCSIDRGALDNTILEDGVIIDNLVQI**

14 4300STDY7045763 100.0% 99.7% **VTITGGSKLRDRVRIHSSTVIGGEGFGFAPYQGKWHRIAQLGSVLIGNDVRIGSNCSIDRGALDNTILEDGVIIDNLVQI**

15 4300STDY7045798 100.0% 99.7% **VTITGGSKLRDRVRIHSSTVIGGEGFGFAPYQGKWHRIAQLGSVLIGNDVRIGSNCSIDRGALDNTILEDGVIIDNLVQI**

16 4300STDY7045799 100.0% 99.7% **VTITGGSKLRDRVRIHSSTVIGGEGFGFAPYQGKWHRIAQLGSVLIGNDVRIGSNCSIDRGALDNTILEDGVIIDNLVQI**

17 4300STDY7045808 100.0% 99.7% **VTITGGSKLRDRVRIHSSTVIGGEGFGFAPYQGKWHRIAQLGSVLIGNDVRIGSNCSIDRGALDNTILEDGVIIDNLVQI**

18 4300STDY7045806 100.0% 99.7% **VTITGGSKLRDRVRIHSSTVIGGEGFGFAPYQGKWHRIAQLGSVLIGNDVRIGSNCSIDRGALDNTILEDGVIIDNLVQI**

19 4300STDY7045811 100.0% 99.7% **VTITGGSKLRDRVRIHSSTVIGGEGFGFAPYQGKWHRIAQLGSVLIGNDVRIGSNCSIDRGALDNTILEDGVIIDNLVQI**

20 4300STDY7045829 100.0% 99.7% **VTITGGSKLRDRVRIHSSTVIGGEGFGFAPYQGKWHRIAQLGSVLIGNDVRIGSNCSIDRGALDNTILEDGVIIDNLVQI**

21 4300STDY7045870 100.0% 99.7% **VTITGGSKLRDRVRIHSSTVIGGEGFGFAPYQGKWHRIAQLGSVLIGNDVRIGSNCSIDRGALDNTILEDGVIIDNLVQI**

22 4300STDY7045866 100.0% 99.7% **VTITGGSKLRDRVRIHSSTVIGGEGFGFAPYQGKWHRIAQLGSVLIGNDVRIGSNCSIDRGALDNTILEDGVIIDNLVQI**

23 4300STDY6542380 100.0% 99.7% **VTITGGSKLRDRVRIHSSTVIGGEGFGFAPYQGKWHRIAQLGSVLIGNDVRIGSNCSIDRGALDNTILEDGVIIDNLVQI**

cov pid **241**  **: . . . . 3 . .** **320**

1 ATCC19606 100.0% 100.0% **AHNVHIGSNTAIAAKCGIAGSTKIGKNCILAGACGVAGHLSIADNVTLTGMSMVTKNISEAGTYSSGTGLFENNHWKKTI**

2 M10 68.8% 100.0% **AHNVHIGSNTAIAAKCGIAGSTKIGKNCILAGACGVAGHLSIADNVTLTGMSMVTKNISEAGTYSSGTGLFENNHWKKTI**

3 A5 100.0% 99.7% **AHNVHIGSNTAIAAKCGIAGSTKIGKNCILAGACGVAGHLSIADNVTLTGMSMVTKNISEAGTYSSGTGLFENNHWKKTI**

4 Aci00709 100.0% 99.7% **AHNVHIGSNTAIAAKCGIAGSTKIGKNCILAGACGVAGHLSIADNVTLTGMSMVTKNISEAGTYSSGTGLFENNHWKKTI**

5 Aci00711 100.0% 99.7% **AHNVHIGSNTAIAAKCGIAGSTKIGKNCILAGACGVAGHLSIADNVTLTGMSMVTKNISEAGTYSSGTGLFENNHWKKTI**

6 KAB15 100.0% 99.7% **AHNVHIGSNTAIAAKCGIAGSTKIGKNCILAGACGVAGHLSIADNVTLTGMSMVTKNISEAGTYSSGTGLFENNHWKKTI**

7 M19 100.0% 99.7% **AHNVHIGSNTAIAAKCGIAGSTKIGKNCILAGACGVAGHLSIADNVTLTGMSMVTKNISEAGTYSSGTGLFENNHWKKTI**

8 PT022 100.0% 99.7% **AHNVHIGSNTAIAAKCGIAGSTKIGKNCILAGACGVAGHLSIADNVTLTGMSMVTKNISEAGTYSSGTGLFENNHWKKTI**

9 PSU091 100.0% 99.7% **AHNVHIGSNTAIAAKCGIAGSTKIGKNCILAGACGVAGHLSIADNVTLTGMSMVTKNISEAGTYSSGTGLFENNHWKKTI**

10 151 100.0% 99.7% **AHNVHIGSNTAIAAKCGIAGSTKIGKNCILAGACGVAGHLSIADNVTLTGMSMVTKNISEAGTYSSGTGLFENNHWKKTI**

11 GML-KP48-AB-TR 100.0% 99.7% **AHNVHIGSNTAIAAKCGIAGSTKIGKNCILAGACGVAGHLSIADNVTLTGMSMVTKNISEAGTYSSGTGLFENNHWKKTI**

12 198 100.0% 99.7% **AHNVHIGSNTAIAAKCGIAGSTKIGKNCILAGACGVAGHLSIADNVTLTGMSMVTKNISEAGTYSSGTGLFENNHWKKTI**

13 4300STDY7045706 100.0% 99.7% **AHNVHIGSNTAIAAKCGIAGSTKIGKNCILAGACGVAGHLSIADNVTLTGMSMVTKNISEAGTYSSGTGLFENNHWKKTI**

14 4300STDY7045763 100.0% 99.7% **AHNVHIGSNTAIAAKCGIAGSTKIGKNCILAGACGVAGHLSIADNVTLTGMSMVTKNISEAGTYSSGTGLFENNHWKKTI**

15 4300STDY7045798 100.0% 99.7% **AHNVHIGSNTAIAAKCGIAGSTKIGKNCILAGACGVAGHLSIADNVTLTGMSMVTKNISEAGTYSSGTGLFENNHWKKTI**

16 4300STDY7045799 100.0% 99.7% **AHNVHIGSNTAIAAKCGIAGSTKIGKNCILAGACGVAGHLSIADNVTLTGMSMVTKNISEAGTYSSGTGLFENNHWKKTI**

17 4300STDY7045808 100.0% 99.7% **AHNVHIGSNTAIAAKCGIAGSTKIGKNCILAGACGVAGHLSIADNVTLTGMSMVTKNISEAGTYSSGTGLFENNHWKKTI**

18 4300STDY7045806 100.0% 99.7% **AHNVHIGSNTAIAAKCGIAGSTKIGKNCILAGACGVAGHLSIADNVTLTGMSMVTKNISEAGTYSSGTGLFENNHWKKTI**

19 4300STDY7045811 100.0% 99.7% **AHNVHIGSNTAIAAKCGIAGSTKIGKNCILAGACGVAGHLSIADNVTLTGMSMVTKNISEAGTYSSGTGLFENNHWKKTI**

20 4300STDY7045829 100.0% 99.7% **AHNVHIGSNTAIAAKCGIAGSTKIGKNCILAGACGVAGHLSIADNVTLTGMSMVTKNISEAGTYSSGTGLFENNHWKKTI**

21 4300STDY7045870 100.0% 99.7% **AHNVHIGSNTAIAAKCGIAGSTKIGKNCILAGACGVAGHLSIADNVTLTGMSMVTKNISEAGTYSSGTGLFENNHWKKTI**

22 4300STDY7045866 100.0% 99.7% **AHNVHIGSNTAIAAKCGIAGSTKIGKNCILAGACGVAGHLSIADNVTLTGMSMVTKNISEAGTYSSGTGLFENNHWKKTI**

23 4300STDY6542380 100.0% 99.7% **AHNVHIGSNTAIAAKCGIAGSTKIGKNCILAGACGVAGHLSIADNVTLTGMSMVTKNISEAGTYSSGTGLFENNHWKKTI**

cov pid **321**  **. . : ]** **356**

1 ATCC19606 100.0% 100.0% **VRLRQLADVPLTQITKRLDHIQAQIESLESTFNLRK**

2 M10 68.8% 100.0% **VRLRQLADVPLTQITKRLDHIQAQIESLESTFNLRK**

3 A5 100.0% 99.7% **VRLRQLADVPLTQITKRLDHIQAQIESLESTFNLRK**

4 Aci00709 100.0% 99.7% **VRLRQLADVPLTQITKRLDHIQAQIESLESTFNLRK**

5 Aci00711 100.0% 99.7% **VRLRQLADVPLTQITKRLDHIQAQIESLESTFNLRK**

6 KAB15 100.0% 99.7% **VRLRQLADVPLTQITKRLDHIQAQIESLESTFNLRK**

7 M19 100.0% 99.7% **VRLRQLADVPLTQITKRLDHIQAQIESLESTFNLRK**

8 PT022 100.0% 99.7% **VRLRQLADVPLTQITKRLDHIQAQIESLESTFNLRK**

9 PSU091 100.0% 99.7% **VRLRQLADVPLTQITKRLDHIQAQIESLESTFNLRK**

10 151 100.0% 99.7% **VRLRQLADVPLTQITKRLDHIQAQIESLESTFNLRK**

11 GML-KP48-AB-TR 100.0% 99.7% **VRLRQLADVPLTQITKRLDHIQAQIESLESTFNLRK**

12 198 100.0% 99.7% **VRLRQLADVPLTQITKRLDHIQAQIESLESTFNLRK**

13 4300STDY7045706 100.0% 99.7% **VRLRQLADVPLTQITKRLDHIQAQIESLESTFNLRK**

14 4300STDY7045763 100.0% 99.7% **VRLRQLADVPLTQITKRLDHIQAQIESLESTFNLRK**

15 4300STDY7045798 100.0% 99.7% **VRLRQLADVPLTQITKRLDHIQAQIESLESTFNLRK**

16 4300STDY7045799 100.0% 99.7% **VRLRQLADVPLTQITKRLDHIQAQIESLESTFNLRK**

17 4300STDY7045808 100.0% 99.7% **VRLRQLADVPLTQITKRLDHIQAQIESLESTFNLRK**

18 4300STDY7045806 100.0% 99.7% **VRLRQLADVPLTQITKRLDHIQAQIESLESTFNLRK**

19 4300STDY7045811 100.0% 99.7% **VRLRQLADVPLTQITKRLDHIQAQIESLESTFNLRK**

20 4300STDY7045829 100.0% 99.7% **VRLRQLADVPLTQITKRLDHIQAQIESLESTFNLRK**

21 4300STDY7045870 100.0% 99.7% **VRLRQLADVPLTQITKRLDHIQAQIESLESTFNLRK**

22 4300STDY7045866 100.0% 99.7% **VRLRQLADVPLTQITKRLDHIQAQIESLESTFNLRK**

23 4300STDY6542380 100.0% 99.7% **VRLRQLADVPLTQITKRLDHIQAQIESLESTFNLRK**

**Supplementary Figure 19: Multiple sequence alignment (MSA) of the predicted amino acid sequence of LpxD carried by ST164^Pas^ the phylogenetically related isolate M10 and close genomes retrieved from the BV-BRC database compared to the respective gene in *A. baumannii* ATCC 19606.** MSA was created by the A multiple alignment viewer MView hosted by the EMBL-EBI; cov, coverage; pid, percent identity.

**Supplementary Table 1: Data of the genomes retrieved from BV-BRC database for mutation analysis**

| BV-BRC ID | Strain | ST^Pas^ | BioProject | BioSample | GenBank | Collection Year | Isolation Country | Colistin Susceptibility | Reference |
| --- | --- | --- | --- | --- | --- | --- | --- | --- | --- |
| 470.11568 | MS14413 | 2 | PRJNA631347 | SAMN14859482 | CP054302 | 2016 | Australia | R | (Roberts et al., 2021) |
| 470.12191 | ATCC 19606 | 52 | PRJNA645521 | SAMN15507635 | CP059040 | NA | NA | S | (Zhu et al., 2020) |
| 470.12436 | SQ093 | 2 | PRJNA676010 | SAMN16807097 | JADQAF000000000 | 2013 | China | NA | - |
| 470.13048 | KAB3 | 2 | PRJNA636771 | SAMN15083386 | JABTWS000000000 | 2017 | Kenya | ND | (Musila et al., 2021) |
| 470.13786 | M17 | 2 | PRJNA690827 | SAMN17266007 | JAESHE000000000 | 2020 | Egypt | S | (Hamed et al., 2022) |
| 470.13790 | M04 | 2 | PRJNA690827 | SAMN17265994 | JAESHP000000000 | 2020 | Egypt | S |  |
| 470.13793 | M05 | 2 | PRJNA690827 | SAMN17265995 | JAESHO000000000 | 2020 | Egypt | S |  |
| 470.13791 | M20 | 2 | PRJNA690827 | SAMN17266010 | JAESHB000000000 | 2020 | Egypt | S |  |
| 470.13795 | M16 | 2 | PRJNA690827 | SAMN17266006 | JAESHF000000000 | 2020 | Egypt | S |  |
| 470.13798 | M01 | 2 | PRJNA690827 | SAMN17265991 | JAESHS000000000 | 2020 | Egypt | S |  |
| 470.15481 | AC-40 | 2 | PRJNA628907 | SAMN14753157 | JABETG000000000 | 2018 | Sudan | S | (Al-Hassan et al., 2021) |
| 470.15487 | AC-14 | 2 | PRJNA628907 | SAMN14753147 | JABESW000000000 | 2018 | Sudan | S |  |
| 470.15498 | AC-45 | 2 | PRJNA628907 | SAMN14753158 | JABETH000000000 | 2018 | Sudan | S |  |
| 470.15501 | AC-23 | 2 | PRJNA628907 | SAMN14753151 | JABETA000000000 | 2018 | Sudan | S |  |
| 470.15514 | SUH-26-2 | 2 | PRJNA628907 | SAMN14753120 | JABESF000000000 | 2017 | Sudan | S |  |
| 470.15519 | SUH-11-2 | 2 | PRJNA628907 | SAMN14753131 | JABERW000000000 | 2017 | Sudan | S |  |
| 470.15521 | SUH-11-1 | 2 | PRJNA628907 | SAMN14753130 | JABERV000000000 | 2017 | Sudan | S |  |
| 470.17376 | A21 | 2 | PRJNA809652 | SAMN26197010 | JAKUMX000000000 | 2012 | China | S | (Dong et al., 2022) |
| 470.17485 | SK044 | 2 | PRJNA752484 | SAMN20599345 | JAIGTP000000000 | 2019 | Thailand | ND | (Chukamnerd et al., 2022) |
| 470.17505 | SK011 | 2 | PRJNA752484 | SAMN20599326 | JAIGUI000000000 | 2019 | Thailand | ND |  |
| 470.17512 | SK002 | 2 | PRJNA752484 | SAMN20599323 | JAIGUL000000000 | 2019 | Thailand | ND |  |
| 470.17568 | PSU120 | 2 | PRJNA752484 | SAMN20599262 | JAIGWU000000000 | 2019 | Thailand | ND |  |
| 470.4905 | KUSSH35 | 2 | PRJNA380997 | SAMN06690271 | NEPN00000000 | 2015 | Kuwait | ND | (Nasser et al., 2018) |
| 470.4907 | KUFAR56 | 2 | PRJNA380997 | SAMN06690282 | NEPC00000000 | 2015 | Kuwait | ND |  |
| 470.4920 | KUSSH15 | 2 | PRJNA380997 | SAMN06690262 | NEPW00000000 | 2014 | Kuwait | ND |  |
| 470.4925 | KUSSH37 | 2 | PRJNA380997 | SAMN06690273 | NEPL00000000 | 2015 | Kuwait | ND |  |
| 470.4926 | KUSSH36 | 2 | PRJNA380997 | SAMN06690272 | NEPM00000000 | 2015 | Kuwait | ND |  |
| 470.7338 | 130 | 2 | PRJNA278886 | SAMN04339712 | NA | 2015 | USA | ND | BV-BRC |
| 470.8444 | Ab34 | 2 | PRJNA541408 | SAMN11584666 | VALV00000000 | 2013 | China | S | (Zhang et al., 2021) |
| 470.8446 | Ab35 | 2 | PRJNA541408 | SAMN11584667 | VALW00000000 | 2013 | China | S |  |
| 470.8447 | Ab36 | 2 | PRJNA541408 | SAMN11584668 | VALX00000000 | 2013 | China | S |  |
| 470.8449 | Ab38 | 2 | PRJNA541408 | SAMN11584670 | VALZ00000000 | 2013 | China | S |  |
| 470.8450 | Ab40 | 2 | PRJNA541408 | SAMN11584671 | VAMA00000000 | 2013 | China | S |  |
| 470.8451 | Ab41 | 2 | PRJNA541408 | SAMN11584672 | VAMB00000000 | 2013 | China | S |  |
| 470.8469 | Ab15 | 2 | PRJNA541386 | SAMN11584449 | VAMT00000000 | 2013 | China | S |  |
| 470.8494 | Ab65 | 2 | PRJNA541822 | SAMN11603659 | VAOL00000000 | 2014 | China | S |  |
| 470.8495 | Ab64 | 2 | PRJNA541822 | SAMN11603658 | VAOM00000000 | 2014 | China | S |  |
| 470.9363 | AB22 | 2 | PRJNA573295 | SAMN12869267 | WBIZ00000000 | 2012 | Malaysia | NA | - |
| 470.10191 | SRR3222490 | 19 | NA | NA | NA | NA | USA | ND | BV-BRC |
| 470.10394 | SRR3227013 | 19 | NA | NA | NA | NA | USA | ND | BV-BRC |
| 470.10442 | SRR3228488 | 19 | NA | NA | NA | NA | USA | ND | BV-BRC |
| 470.10485 | SRR3228565 | 19 | NA | NA | NA | NA | USA | ND | BV-BRC |
| 470.12102 | A18 | 19 | PRJNA659545 | SAMN15915108 | JACSTP000000000 | 2015 | Egypt | ND | (Abouelfetouh et al., 2022) |
| 470.13787 | M15 | 19 | PRJNA690827 | SAMN17266005 | JAESHG000000000 | 2020 | Egypt | S | (Hamed et al., 2022) |
| 470.13788 | M12 | 19 | PRJNA690827 | SAMN17266002 | JAESHJ000000000 | 2020 | Egypt | S |  |
| 470.13801 | M06 | ND | PRJNA690827 | SAMN17265996 | JAESHN000000000 | 2020 | Egypt | S |  |
| 470.13802 | M09 | ND | PRJNA690827 | SAMN17265999 | JAESHM000000000 | 2020 | Egypt | S |  |
| 470.6781 | MRSN7133 | 19 | PRJNA300270 | SAMN04546722 | AAYNHK000000000 | NA | NA | NA | - |
| 470.7619 | MRSN7130 | 19 | PRJNA300270 | SAMN04555199 | AAYMYI000000000 | 2004 | USA | NA | - |
| 470.7665 | MRSN7202 | 19 | PRJNA300270 | SAMN04555511 | AAYMVF000000000 | 2003 | USA | NA | - |
| 470.7682 | MRSN7224 | 19 | PRJNA300270 | SAMN04555300 | AAYMWN000000000 | 2003 | USA | NA | - |
| 903901.3 | OIFC074 | 19 | PRJNA53401 | SAMN00761203 | AMDE00000000 | 2003 | Germany | ND | BV-BRC |
| 470.13058 | 15953 | 85 | PRJNA701275 | SAMN17860400 | JAFKQS000000000 | 2018 | Nigeria | NA | - |
| 470.13368 | 15946 | 85 | PRJNA701275 | SAMN17860394 | JAFKDG000000000 | 2017 | Nigeria | NA | - |
| 470.13418 | Survcare112 | 85 | PRJNA692829 | SAMN17372031 | JAFHVR000000000 | 2018 | Germany | NA | - |
| 470.13796 | M11 | 85 | PRJNA690827 | SAMN17266001 | JAESHK000000000 | 2020 | Egypt | S | (Hamed et al., 2022) |
| 470.13797 | M18 | 85 | PRJNA690827 | SAMN17266008 | JAESHD000000000 | 2020 | Egypt | S |  |
| 470.13800 | M02 | 85 | PRJNA690827 | SAMN17265992 | JAESHR000000000 | 2020 | Egypt | S |  |
| 470.14058 | Cl300 | 85 | PRJNA607827 | SAMN21240079 | CP082952,CP082953 | 2015 | Lebanon | S | (Mann et al., 2022) |
| 470.14590 | 17A1955 | 85 | PRJNA766794 | SAMN21876342 | JAIWUA000000000 | 2017 | France | NA | - |
| 470.1507 | ACMH-6200 | 85 | PRJNA294951 | SAMN04088953 | LKMA00000000 | 2012 | Lebanon | S | (Rafei et al., 2014) |
| 470.1508 | ACMH-6201 | 85 | PRJNA294952 | SAMN04089926 | LKMB00000000 | 2012 | Lebanon | S |  |
| 470.15460 | MIN-015 | 85 | PRJNA769286 | SAMN22108556 | JAJBHK000000000 | 2016 | Pakistan | NA | - |
| 470.15668 | 17A1955 | 85 | PRJNA766794 | SAMN21876342 | JAIWUA000000000 | 2017 | France | NA | - |
| 470.16140 | AB177-VUB | 85 | PRJNA798866 | SAMN25131649 | CP091361 | NA | Belgium | S | (Valcek et al., 2022) |
| 470.17396 | P116A | 85 | PRJNA823741 | SAMN27356705 | JALJVB000000000 | 2021 | Ghana | NA | - |
| 470.17952 | 26 | 85 | PRJNA835871 | SAMN28119815 | JAMCCB000000000 | 2014-2015 | Ghana | NA | - |
| 470.2844 | AR_0037 | 85 | PRJNA292904 | SAMN04014878 | MPBX00000000 | NA | NA | S | NCBI |
| 470.2845 | AR_0033 | 85 | PRJNA292904 | SAMN04014874 | MPCA00000000 | NA | NA | S | NCBI |
| 470.3103 | AB-A | 85 | PRJNA317645 | SAMN04589238 | LWSM00000000 | 2014 | Ethiopia | NA | - |
| 470.3104 | AB-C | 85 | PRJNA317645 | SAMN04622656 | LWSO00000000 | 2014 | Ethiopia | NA | - |
| 470.3105 | AB-B | 85 | PRJNA317645 | SAMN04589239 | LWSN00000000 | 2013 | Ethiopia | NA | - |
| 470.3532 | AE27M | 85 | PRJEB15065 | SAMEA103954683 | FWWO00000000 | NA | NA | NA | - |
| 470.3808 | AE3M | 85 | PRJEB15065 | SAMEA103920782 | FWFB00000000 | NA | NA | NA | - |
| 470.4275 | MBL_M1 | 85 | PRJNA377228 | SAMN06459018 | MWTR00000000 | 2013 | Tunisia | NA | - |
| 470.4279 | MBL_M6 | 85 | PRJNA377388 | SAMN06461248 | MWTW00000000 | 2015 | Tunisia | NA | - |
| 470.4283 | MBL_M10 | 85 | PRJNA377417 | SAMN06461382 | MWUA00000000 | 2015 | Tunisia | NA | - |
| 470.4284 | MBL_M9 | 85 | PRJNA377413 | SAMN06461369 | MWTZ00000000 | 2015 | Tunisia | NA | - |
| 470.5046 | R11 | 85 | PRJNA475868 | SAMN09404818 | QKWE00000000 | 2017 | France | NA | - |
| 470.8354 | Ab-NDM-1 | 85 | PRJNA449628 | SAMN08915454 | QBBY00000000 | 2017 | Spain | R | (Fernandez-Cuenca et al., 2020) |
| 470.9114 | MRSN15574 | 85 | PRJNA545079 | SAMN12087653 | VHGP00000000 | 2012 | Germany | S | (Galac et al., 2020) |
| 1221249.3 | AB_1649-8 | 113 | PRJNA172660 | SAMN02471286 | AMHF00000000 | NA | NA | NA | - |
| 1221250.3 | AB_1650-8 | 113 | PRJNA172661 | SAMN02471224 | AMHG00000000 | NA | NA | NA | - |
| 470.13792 | M14 | 113 | PRJNA690827 | SAMN17266004 | JAESHH000000000 | 2020 | Egypt | S | (Hamed et al., 2022) |
| 470.13799 | M03 | 113 | PRJNA690827 | SAMN17265993 | JAESHQ000000000 | 2020 | Egypt | S |  |
| 470.17530 | PT061 | 113 | PRJNA752484 | SAMN20599312 | JAIGUW000000000 | 2019 | Thailand | ND | (Chukamnerd et al., 2022) |
| 470.17551 | PT003 | 113 | PRJNA752484 | SAMN20599277 | JAIGWF000000000 | 2019 | Thailand | ND |  |
| 470.17596 | PSU068 | 113 | PRJNA752484 | SAMN20599232 | JAIGXY000000000 | 2019 | Thailand | ND |  |
| 470.17597 | PSU073 | 113 | PRJNA752484 | SAMN20599234 | JAIGXW000000000 | 2019 | Thailand | ND |  |
| 470.18044 | CCBH26501 | 113 | PRJNA677881 | SAMN27549388 | JAMKEG000000000 | 2019-03-01 | Brazil | ND | (Silveira et al., 2021) |
| 470.3781 | AB363 | 113 | PRJNA320195 | SAMN04924787 | LYNM00000000 | 2013 | Saudi Arabia | NA | - |
| 470.4915 | KUSSH08 | 113 | PRJNA380997 | SAMN06687317 | NEQB00000000 | 2015-04-19 | Kuwait | ND | (Nasser et al., 2018) |
| 470.4919 | KUSSH14 | 113 | PRJNA380997 | SAMN06690261 | NEPX00000000 | 2014-10-23 | Kuwait | ND |  |
| 470.5048 | TUMA | 113 | PRJNA475868 | SAMN09404816 | QKWG00000000 | 2017-05 | France | NA | - |
| 470.5676 | 4300STDY7045886 | 113 | PRJEB19226 | SAMEA104305385 | UFOF00000000 | 2016 | Thailand | NA | - |
| 470.8559 | Aci00866 | 113 | PRJEB31555 | SAMEA5396106 | VAGJ00000000 | 2013-10-06 | Germany | S | (Eigenbrod et al., 2019) |
| 470.8561 | Aci00860 | 113 | PRJEB31555 | SAMEA5396102 | VAGF00000000 | 2013-05-02 | Germany | S |  |
| 470.8567 | Aci00848 | 113 | PRJEB31555 | SAMEA5396095 | VAFY00000000 | 2013-10-11 | Germany | S |  |
| 470.9061 | MRSN351524 | 113 | PRJNA545079 | SAMN12087685 | VHEW00000000 | 2011 | Germany | S | (Galac et al., 2020) |
| 470.12231 | A5 | 164 | PRJNA669537 | SAMN16513546 | JADIWM000000000 | 2018 | South Africa | NA | - |
| 470.12906 | Aci00709 | 164 | PRJEB40828 | SAMEA7451185 | CAJHEZ000000000 | 2014 | Germany | NA | - |
| 470.12917 | Aci00711 | 164 | PRJEB40828 | SAMEA7451187 | CAJHEX000000000 | 2014 | Germany | NA | - |
| 470.13045 | KAB15 | 164 | PRJNA636771 | SAMN15083390 | JABTWM000000000 | 2018 | Kenya | ND | (Musila et al., 2021) |
| 470.13794 | M19 | 164 | PRJNA690827 | SAMN17266009 | JAESHC000000000 | 2020 | Egypt | R | (Hamed et al., 2022) |
|  | M10 | ND | PRJNA690827 | SAMN17266000 | JAESHL000000000 | 2020 | Egypt | S |  |
| 470.17545 | PT022 | 164 | PRJNA752484 | SAMN20599288 | JAIGVU000000000 | 2019 | Thailand | ND | (Chukamnerd et al., 2022) |
| 470.17585 | PSU091 | 164 | PRJNA752484 | SAMN20599246 | JAIGXK000000000 | 2019 | Thailand | ND |  |
| 470.4247 | 151 | 164 | PRJNA411997 | SAMN07692765 | NXHM00000000 | 2015 | Ghana | S | (Agyepong et al., 2019) |
| 470.5038 | GML-KP48-AB-TR | 164 | PRJNA471991 | SAMN09223253 | QHHF00000000 | 2017 | Turkey | NA | - |
| 470.5408 | 198 | 164 | PRJNA411997 | SAMN09768096 | QTKC00000000 | 2015 | Ghana | S | (Agyepong et al., 2019) |
| 470.5497 | 4300STDY7045706 | 164 | PRJEB19226 | SAMEA104305202 | UFIQ00000000 | 2016 | Thailand | NA | - |
| 470.5553 | 4300STDY7045763 | 164 | PRJEB19226 | SAMEA104305259 | UFKG00000000 | 2016 | Thailand | NA | - |
| 470.5583 | 4300STDY7045798 | 164 | PRJEB19226 | SAMEA104305293 | UFLR00000000 | 2016 | Thailand | NA | - |
| 470.5610 | 4300STDY7045799 | 164 | PRJEB19226 | SAMEA104305294 | UFLU00000000 | 2016 | Thailand | NA | - |
| 470.5615 | 4300STDY7045808 | 164 | PRJEB19226 | SAMEA104305303 | UFMH00000000 | 2016 | Thailand | NA | - |
| 470.5627 | 4300STDY7045806 | 164 | PRJEB19226 | SAMEA104305301 | UFMD00000000 | 2016 | Thailand | NA | - |
| 470.5629 | 4300STDY7045811 | 164 | PRJEB19226 | SAMEA104305306 | UFMJ00000000 | 2016 | Thailand | NA | - |
| 470.5640 | 4300STDY7045829 | 164 | PRJEB19226 | SAMEA104305324 | UFMP00000000 | 2016 | Thailand | NA | - |
| 470.5677 | 4300STDY7045870 | 164 | PRJEB19226 | SAMEA104305364 | UFOE00000000 | 2016 | Thailand | NA | - |
| 470.5678 | 4300STDY7045866 | 164 | PRJEB19226 | SAMEA104305361 | UFON00000000 | 2016 | Thailand | NA | - |
| 470.16286 | UV_1036 | 25 | NA | NA | NA | 2003-11-12 | Vietnam | S | BV-BRC |
| 470.16335 | 276_ax | 25 | NA | NA | NA | 2005-11-07 | Vietnam | S | BV-BRC |
| 470.16330 | 259_an | 25 | NA | NA | NA | 2005-10-10 | Vietnam | S | BV-BRC |
| 470.15687 | 15A1042 | 315 | PRJNA766794 | SAMN21876320 | JAIWUW000000000 | 2015 | France | NA | - |

S, susceptible; R, resistant; ND, susceptibility to colistin was not determined in the relevant study; NA, colistin susceptibility was not available in the databases and relevant studies could not be found.

**Supplementary Figure 20: P-values of Tukey's multiple comparisons test of ZP values of the groups of isolates that belonged to different GCs.** P-values <0.05 were considered as statistically significant.

**Supplementary Figure 21: P-values of Tukey's multiple comparisons test of ZP values of all isolates.** Yellow highlighted P-values correspond to comparisons including isolates that belonged to the same STs/GCs. P-values <0.05 were considered as statistically significant.

References:

Abouelfetouh, A., et al. (2022). Diversity of carbapenem-resistant Acinetobacter baumannii and bacteriophage-mediated spread of the Oxa23 carbapenemase. *Microb Genom* 8. doi: <https://doi.org/10.1099/mgen.0.000752>

Agyepong, N., et al. (2019). Whole-Genome Sequences of Two Multidrug-Resistant Acinetobacter baumannii Strains Isolated from Patients with Urinary Tract Infection in Ghana. *Microbiol Resour Announc* 8. doi: <https://doi.org/10.1128/MRA.00270-19>

Al-Hassan, L., et al. (2021). Molecular Epidemiology of Carbapenem-Resistant Acinetobacter baumannii From Khartoum State, Sudan. *Front Microbiol* 12**,** 628736. doi: <https://doi.org/10.3389/fmicb.2021.628736>

Chukamnerd, A., et al. (2022). Whole-genome analysis of carbapenem-resistant Acinetobacter baumannii from clinical isolates in Southern Thailand. *Comput Struct Biotechnol J* 20**,** 545-558. doi: <https://doi.org/10.1016/j.csbj.2021.12.038>

Dong, J.F., et al. (2022). Comparative genomics analysis of Acinetobacter baumannii multi-drug resistant and drug sensitive strains in China. *Microb Pathog* 165**,** 105492. doi: <https://doi.org/10.1016/j.micpath.2022.105492>

Eigenbrod, T., et al. (2019). Molecular characterization of carbapenem-resistant Acinetobacter baumannii using WGS revealed missed transmission events in Germany from 2012–15. *Journal of Antimicrobial Chemotherapy* 74**,** 3473-3480. doi: <https://doi.org/10.1093/jac/dkz360> %J Journal of Antimicrobial Chemotherapy

Fernandez-Cuenca, F., et al. (2020). First identification of bla(NDM-1) carbapenemase in bla(OXA-94)-producing Acinetobacter baumannii ST85 in Spain. *Enferm Infecc Microbiol Clin (Engl Ed)* 38**,** 11-15. doi: <https://doi.org/10.1016/j.eimc.2019.03.008>

Galac, M.R., et al. (2020). A Diverse Panel of Clinical Acinetobacter baumannii for Research and Development. *Antimicrob Agents Chemother* 64. doi: <https://doi.org/10.1128/AAC.00840-20>

Hamed, S.M., et al. (2022). Genetic Configuration of Genomic Resistance Islands in Acinetobacter baumannii Clinical Isolates From Egypt. *Front Microbiol* 13**,** 878912. doi: <https://doi.org/10.3389/fmicb.2022.878912>

Mann, R., et al. (2022). Variants of Tn6924, a Novel Tn7 Family Transposon Carrying the bla(NDM) Metallo-beta-Lactamase and 14 Copies of the aphA6 Amikacin Resistance Genes Found in Acinetobacter baumannii. *Microbiol Spectr* 10**,** e0174521. doi: <https://doi.org/10.1128/spectrum.01745-21>

Musila, L., et al. (2021). Detection of diverse carbapenem and multidrug resistance genes and high-risk strain types among carbapenem non-susceptible clinical isolates of target gram-negative bacteria in Kenya. *PLoS One* 16**,** e0246937. doi: <https://doi.org/10.1371/journal.pone.0246937>

Nasser, K., et al. (2018). Draft Genome Sequences of Six Multidrug-Resistant Clinical Strains of Acinetobacter baumannii, Isolated at Two Major Hospitals in Kuwait. *Genome Announc* 6. doi: <https://doi.org/10.1128/genomeA.00264-18>

Rafei, R., et al. (2014). First report of blaNDM-1-producing Acinetobacter baumannii isolated in Lebanon from civilians wounded during the Syrian war. *Int J Infect Dis* 21**,** 21-23. doi: <https://doi.org/10.1016/j.ijid.2014.01.004>

Roberts, L.W., et al. (2021). Genomic surveillance, characterization and intervention of a polymicrobial multidrug-resistant outbreak in critical care. *Microb Genom* 7. doi: <https://doi.org/10.1099/mgen.0.000530>

Silveira, M.C., et al. (2021). Genetic Basis of Antimicrobial Resistant Gram-Negative Bacteria Isolated From Bloodstream in Brazil. *Front Med (Lausanne)* 8**,** 635206. doi: <https://doi.org/10.3389/fmed.2021.635206>

Valcek, A., et al. (2022). Genomic Analysis of a Strain Collection Containing Multidrug-, Extensively Drug-, Pandrug-, and Carbapenem-Resistant Modern Clinical Isolates of Acinetobacter baumannii. *Antimicrob Agents Chemother* 66**,** e0089222. doi: <https://doi.org/10.1128/aac.00892-22>

Zhang, X., et al. (2021). Molecular Epidemiology and Clone Transmission of Carbapenem-Resistant Acinetobacter baumannii in ICU Rooms. *Front Cell Infect Microbiol* 11**,** 633817. doi: <https://doi.org/10.3389/fcimb.2021.633817>

Zhu, Y., et al. (2020). Complete genome sequence and genome-scale metabolic modelling of Acinetobacter baumannii type strain ATCC 19606. *International Journal of Medical Microbiology* 310**,** 151412. doi: <https://doi.org/https://doi.org/10.1016/j.ijmm.2020.151412>
